# Intervention effectiveness in reducing the clustering of non-communicable disease risk factors in the workplace: A quasi-experimental study

**DOI:** 10.1371/journal.pone.0317460

**Published:** 2025-02-06

**Authors:** Nahla Guesmi, Sihem Ben Fredj, Nawel Zammit, Rim Ghammam, Imed Harrabi, Firas Chouikha, Maher Maoua, Jihen Maatoug, Hassen Ghannem

**Affiliations:** 1 Department of Epidemiology, “LR19SP03”, University Hospital Farhat Hached, Sousse, Tunisia; 2 Faculty of Medicine of Sousse, University of Sousse, Sousse, Tunisia; 3 Department of Occupational Medicine, “LR19SP03”, University Hospital Farhat Hached, Sousse, Tunisia; National Trauma Research Institute, AUSTRALIA

## Abstract

Understanding the clustering patterns of non-communicable disease risk factors is important to address chronic diseases effectively, thus minimizing their onset and enhancing overall health. This study aimed to assess the feasibility and efficacy of a three-year workplace intervention in decreasing clustering of non-communicable disease risk factors in employees. A quasi-experimental study, including six companies, was conducted in the governorate of Sousse between 2010 and 2014. It involved an intervention group (Sousse-Jawhara and Sousse-Erriadh) and a control group (M’saken). The sample of participants in both groups was representative. Actions promoting physical activity, healthy diet, and smoking cessation in the workplace were included in this intervention. The participants’ socio-demographic characteristics and data concerning the risk factors were collected through interviews using a pretested questionnaire. The clustering of tobacco use, physical inactivity, unhealthy diet, obesity, and high blood pressure were examined pre- and post-intervention. In the intervention group, the mean risk factors per employee decreased significantly from 1.99 ± 1.00 to 1.81 ± 1.05 (p < 10^−3^). A minor non-significant increase, from 1.72 ± 0.97 to 1.78 ± 1.11, was noted in the control group. In the intervention group, the prevalence of two risk factor clusters dropped significantly from 40% to 34.4% pre- and post-intervention (p = 0.014). However, a non-significant decline was noted in the control group. Combinations such as obesity/hypertension and unhealthy diet/physical inactivity tended to aggregate in both groups. Overall, the intervention program showed significant protective effects in reducing the co-occurrence of multiple risk factors in the intervention group, with an adjusted OR of 0.81; CI95% [0.68–0.97]. Along with the existing literature, the present study confirmed the feasibility and effectiveness of health promotion programs in reducing non-communicable disease risk factors and their clustering. Integrating this intervention program into a national health policy could potentially generalize its positive impact.

## Introduction

Non-communicable diseases (NCDs) are a field of public health involving a very broad range of health issue categories, such as cardiovascular diseases, cancer, mental disorders, diabetes etc. [1]. The prevalence of NCDs, previously called “diseases of affluence”, has sharply increased worldwide in the last decades [2]. This rise is attributed to the economic progress, urban development, industrialization, and globalization that have led to changes in individual’s lifestyle worldwide [3] NCDs have a major impact on people’s individual lives and public health costs [4,5]. Regarding people’s individual lives, they can lead to disability, which affects the quality of life and the ability to work. They can also lead to premature death [6]. As for public health costs, they represent a major burden on healthcare systems as they often require ongoing care and treatment, accounting for a large proportion of global healthcare spending [7]. Health initiatives should therefore focus on the prevention and management of NCDs to improve health outcomes and alleviate their economic impact, which is considered a challenging task mainly in low- and middle-income Countries (LMICs) where health systems are marked by fragmented services [8]. The risk of premature death by NCDs is actually the highest in LMICs [9]. In Tunisia, NCDs are responsible for 86% of deaths in 2016 (49% of deaths are caused by cardiovascular diseases) and 16% of premature deaths in the age group 30–70 years in the same year [10]. Overall, five main risk factors can be identified, including tobacco and alcohol consumption, physical inactivity, unhealthy diet, hypertension, and obesity [6]. According to the World Health Organization (WHO), Tunisia is the first Arab country in consuming tobacco, with 50% of males being smokers [11]. In 2001, a study was conducted by the Tunisian National Institute of Public Health on a representative sample of the Tunisian population, involving 8576 subjects aged 15 years or more revealed that the prevalence of overweight is 12.2% (6.1% in males and 18.3% in females p < 10^−2^) [12]. A key approach to address NCDs, especially in LMICs with limited clinical capacity, is to prevent them through policies that reduce unhealthy behaviors and the risk factors [13]. Yet, many policymakers are unsure about where to focus their efforts. These risk factors can often co-occur [14,15]. It has been demonstrated that the risk factors have synergistic effects and that their combination is more harmful to health than their cumulative individual effects [16,17]. Consequently, the WHO has recently recommended an approach to chronic disease prevention focusing on how to address multiple modifiable risk factors [18]. Furthermore, the WHO has proposed the workplace as a priority environment for health promotion in the 21st century [19]. Indeed, the workplace directly affects the workers’ physical, psychological, economic, and social well-being, which in turn affect the health of their families, communities, and societies [20]. It provides an ideal framework and infrastructure to support health promotion for a broad audience [21]. The Chronic Disease Prevention Research Center (CDPRC) in Sousse initiated a large-scale quasi-experimental workplace study in 2009 as a community-based research being a part of non-communicable disease (NCD) prevention programs. The aim of the present study was to investigate the effectiveness of an early intervention on the NCDs risk factor cluster and to examine the predictors of the cumulative NCD risk factors in a Tunisian workforce.

## Methods

### Study design and setting

A quasi-experimental study involving two groups was carried out in two areas in Sousse, Tunisia, between 2010 and 2014. The intervention group included workplaces located in Sousse-Jawhara and Sousse-Erriadh. The workplaces in the control group were located in Sousse- M’saken. The estimated distance between Sousse Erriadh-Jawhara and M’saken is about 20 km. The distance was considered to avoid contamination bias.

### Study sample

The sample size calculation was based on the type 1 error of α =  5%, the type 2 error of β =  20%, and the change in the prevalence of various risk factors (smoking, unhealthy diet, lack of physical activity, obesity, arterial hypertension) [22] of 6% between pre- and post- intervention. The intervention group included three companies (Epi d’Or, TEXMED, UATS), selected by convenience, located in Sousse. The control group included three companies (STIP, AAF, FITLEC) from M’saken, and it was relatively similar to the intervention group in terms of size and gender composition. The estimated distance between Sousse Erriadh-Jawhara and M’saken is about 20 km. The distance was considered to avoid contamination bias. All the employees in the selected companies were included in data collection. The pre- and post- intervention assessments concerned both groups. Before the start of the intervention program, 1775 employees were included in the study, with 914 in the intervention group and 861 in the control group.

### Data collection and measurements

For data collection, a pretested and standardized questionnaire administered for the participants by interview with trained medical doctors at the worksite was used. Data regarding the sociodemographic characteristics, professional characteristics, eating habits, physical activity habits, and tobacco use habits were collected. The same questionnaire was administered by interview at the pre- and post- intervention assessments. Biometric data, including height and weight were also collected. The weight was measured to the nearest 0.1 kg using a portable electronic scale. The height in standing position was measured in participants with bare feet to the nearest 0.5 cm. Blood pressure was measured in a sitting position twice at rest using an arm electronic sphygmomanometer. The same questionnaire was administered by interview at the pre- and post- intervention assessments [20]. Data collection took two months during both the pre-assessment and post-assessment periods.

### Intervention

The “Together in Health” project was launched in 2009, in Sousse, Tunisia, by the Chronic Disease Prevention Research Centre in collaboration with the Department of Occupational Health at the University Hospital Farhat Hached of Sousse and the Grouping of Occupational Medicine of Sousse. A multidisciplinary work team of doctors, psychologists, nutritionists, and graphic designers have been working to produce guides and interventional tools adapted to each stakeholder. This project was implemented in three different settings: workplaces, households, and schools and aimed to reduce major risk factors for chronic diseases at the community level. The intervention actions were discussed with the different partners according to their feasibility in the context of the city of Sousse. In this study, focus was put specifically on workplace settings. The intervention strategies were based on employee engagement [23] and a social contextual framework for multi-level behavioral influence [24]. Accordingly, individuals’ behaviors and attitudes were influenced by the environments [25]. Focus was therefore put on three main points. The first involved effective communication to motivate managers and other stakeholders to participate in promoting healthy lifestyles in the workplace [26]. The second was based on creating a work environment that supports healthy eating, physical activity, and tobacco control [21]. The third point consisted in raising the workers’ awareness of healthy lifestyles and the risk factors for cardiovascular diseases. Therefore, to highlight the importance of the intervention, a strategic plan comprising different levels of action targeting four groups of stakeholders was prepared. Firstly, the occupational health team was asked to create a tailored training program for doctors working on the implementation of a healthy lifestyle, organize brainstorming meetings to negotiate realistic and operational changes to be made to the working environment, and produce a newsletter containing updated recommendations for the doctors concerned. Secondly, employers were encouraged to motivate their employees to adopt a healthier lifestyle by offering rewards, providing healthy food in canteens, taking environmental measures, etc. Thirdly, occupational physicians were involved by organizing seminars and workshops, providing consultations on smoking cessation in the workplace, organizing regular screening for non-communicable diseases and the related risk factors, distributing teaching and educational materials, etc. Fourthly, motivated employees were designated as Leaders and they were asked to adopt a healthier lifestyle, to encourage those around them to follow in their footsteps and to create sports clubs and sports tournaments between different departments, etc. The intervention guide in the workplace settings included the promotion of smoking cessation. Smoking was prohibited in the common spaces in the companies. In addition, smoking rooms were provided for smokers. To promote healthy eating, healthy food was provided in the company cafeterias after evaluating the needs of the kitchen staff and the characteristics of the customers’ consumption. With regard to physical activities, free exercise activities were organized for employees and alternative transportation, such as walking, riding a bike, was encouraged. See S1 File.

### Definitions of variables

The WHO defines physical activity as any bodily movement produced by skeletal muscles that requires energy expenditure. The recommended level of physical activity was used in the present study as defined by the WHO. Physical inactivity is defined when adults aged 18–64 years do less than 150 minutes [1] of moderate intensity aerobic physical activity throughout the week, or they do less than 75 minutes of vigorous intensity aerobic physical activity throughout the week, or an equivalent combination of both moderate and vigorous intensity activities [27]. Physical activity was investigated using the International Physical Activity Questionnaire (IPAQ) [28]. An unhealthy dietary behavior is defined as a diet containing less than five servings of fruits and vegetables per day [29]. Regarding the definition of smokers, the participants were asked the following question, “Do you currently smoke any tobacco products, such as cigarettes, cigars, or pipes?” Those who responded “yes” were defined as smokers. The socioeconomic status (SES) was based on the asset index developed by the World Bank [30] to define overweight and obesity, Body Mass Index (BMI) in kg/m2 was calculated by the ratio of weight to the square of the height and the international BMI cutoff values were used according to the WHO. For adults, the WHO defines overweight as a BMI greater than or equal to 25, and obesity as a BMI greater than or equal to 30 [31]. Blood pressure in a sitting position at rest was measured twice by medical doctors using an adjustable cuff electronic sphygmomanometer (OMRON HEALTHCARE EOROPE B.V.Kruisweg 577-2132 NA Hoofddorp, The Netherlands, M3 Intellisense (HEM-7051-E)). The first measurement was taken after 20 min of rest and the second one after completing the questionnaire (15 min). The mean value of the 2 measurements was used to calculate systolic and diastolic blood pressure and to define high blood pressure on the day of the measurement. Adults were considered to have hypertension if they had systolic blood pressure of 140 mm Hg or more and/or diastolic blood pressure of 90 mm Hg or more on the day of the visit [32].

### Data analyses

Statistical analysis was performed using the SPSS 20.0 software for windows. Continuous variables were presented as Mean (standard deviations) and categorical variables as percentages (%). Chi square test was used to compare percentages and Student t test to compare means in independent groups. The level of statistical significance was set at p < 0.05.

The baseline characteristics of the study population were evaluated for the intervention group and the control group separately in pre- and post-assessment. The prevalence of lifestyle risk factors was investigated in the study population. The observed frequency of single risk factors coded as a binary variable was described (presence =  1; absence =  0). The prevalence of multiple risk factors was estimated from the sum of risk factors, being ranked from 0 to 5 (0 =  no risk factor, 5 =  all risk factors), based on the distribution observed in the sample at pre- and post-assessment in both groups. First, the clustering of the five lifestyle risk factors was examined. The clustering of two or more risk factors was studied based on ratios of the observed and expected prevalence of one, two, three, four, and five simultaneously occurring risk habits. The expected proportion was calculated by multiplying the individual probabilities of each risk factor based on its occurrence in the study population for the intervention group and the control group separately in pre-and post-assessment. The difference between the observed and the expected proportion (O/E) was calculated. The aggregation was defined as existing when the observed combination (O) exceeds the expected prevalence factors (E) of the combination. If the result of the ratio of observed-to-expected (O/E) was greater than 1, it indicated the existence of aggregation between the risk factors. Secondly, the prevalence odds ratio (POR) was used to calculate the clustering of two risk factors, regardless of the exposure to the other three risk factors. The POR represents the estimation of the relative odds of a risk factor in relation to another risk factor, which is calculated using the following equation:


$$\frac{N_{11}\times N_{00}}{N_{10}\times N_{01}}.$$

Where $N_{11}$ is the number of individuals exposed to two risk factors, $N_{00}$ is the number of individuals without any risk factor, $N_{10}$ is the number of respondents showing only one risk factor, and $N_{01}$ representing those showing the other risk factors. For instance, a POR of 1.5 indicated that subjects exhibiting one risk factor (e.g., physical inactivity) were 1.5 times more likely to show the other risk factor (e.g., unhealthy diet) compared to the subjects not exposed to the risk factor (e.g., physical inactivity). If 1 was not included in the 95% confidence interval, the two risk factors cluster. We evaluated if the clustering of the risk factors was differential between the intervention group and the control group in pre-and post-assessment. The final analysis was multivariate. For the selection of explanatory variables, univariate analysis was applied to examine the association between each independent variable and the clustered risk factors. Then, Stepwise multinomial logistic regression analysis was used to seek the independent effects of the variables with significant univariate associations or with p-value ≤ 0.2. These variables were included as potential confounding variables. The adjusted odds ratios (OR) and 95% confidence intervals were calculated. The three-level variable, i.e., clusters of the risk factors, was set as the dependent variable in the regression model. The model estimated the probability of having a certain number of lifestyle risk factors in a respondent compared to the reference group having no or one risk factor, with p <  0.05 being adopted for all the analyses as the threshold for statistical significance. All the analyses were conducted for the intervention group and the control group separately at pre-and post-assessment.

### Ethical considerations

The protocol, data collection forms, and questionnaire received written approval from the ethics and research committee at University Hospital Farhat Hached, Sousse, IORG 007439. Authorizations from the Ministry of Health, the governor of Sousse, and the Occupational Medicine Group in Sousse were obtained. Informed written consent was obtained from the participants before the beginning of the intervention program which included interactive educational actions having no damage to the participants’ integrity.

At the end of the program and after the completion of post-intervention assessment, the same health education program was started for the control group as a delayed intervention.

## Results

### Characteristics of the study population

The overall response rate at the start of the intervention was 74.6%. After the intervention, the global response rate was 71.9%. The sociodemographic characteristics of the study population are presented in Table 1.

**Table 1:** The socio-demographic characteristics of the employees in the intervention and control groups pre- and post-intervention (Sousse, Tunisia. 2009–2014).

Characteristics	Intervention group			Control group		
	Pre	Post	p	Pre	Post	p
**Mean Age** (SD)	32.25 (8.11)	33.86 (8.10)	**<10** ^**−3**^	35.40 (8.79)	38.90 (8.77)	**<10** ^**−3**^
**Age group** n (%)						
[12–29]	384 (42.5)	373 (34.0)	**0.001**	242 (28.7)	158 (15.6)	**<10** ^**−3**^
[30–39]	357 (39.5)	489 (44.5)		284 (33.7)	387 (38.1)	
[40–49]	117 (13.0)	178 (16.2)		283 (33.6)	339 (33.4)	
[50–67]	45 (5.0)	58 (5.3)		33 (3.9)	131 (12.9)	
**Gender** n (%)						
Male	591 (64.7)	719 (65.5)	0.70	508(59.0)	623 (61.4)	0.29
Female	323 (35.3)	379 (34.5)		353 (41.0)	392 (38.6)	
**Marital status** n (%)						
Single	438 (48.5)	387 (35.3)	**<10** ^**−3**^	238 (33.5)	252 (25.1)	**<10** ^**−3**^
Divorced or widower	18 (2.0)	11 (1.0)		9 (1.1)	16 (1.6)	
Married	447 (49.5)	698 (63.7)		553 (65.4)	741 (73.4)	
**Educational level** n (%)						
Illiterate or Primary	290 (32.2)	316 (28.8)	0.37	100 (12.0)	144 (14.2)	0.13
College or Secondary	447 (49.6)	592 (54.0)		646 (77.6)	764 (75.3)	
University	165 (18.2)	189 (17.2)		87 (10.4)	106 (10.5)	
**Occupation** n (%)						
Blue collar	669 (74.6)	818 (75.3)	0.69	698 (84.9)	760 (80.6)	**0.016**
White collar	228 (25.4)	269 (24.7)		124 (15.1)	183 (19.4)	

Table 2 shows the observed and expected prevalence of the 32 possible combinations of the five chronic disease risk factors. At baseline, in the intervention group, the expected prevalence of the five risk factors was 0.4%; however, the observed prevalence of the five risk factors was 0.7% at pre-assessment, representing a 75% increase from expectation (O/E =  1.75). This ratio increased after the assessment in both the intervention and control groups. With regard to the simultaneity of the four risk factors, the combination “lack of exercise, unhealthy diet, obesity, hypertension” was relevant and it was 300% higher than expected for independent factors (O/E =  3) in the intervention group. However, it fell slightly in hindsight. As for the simultaneous occurrence of three risk factors, the most noteworthy was the combinations “Physical inactivity + obesity + high blood pressure” (O/E =  2.17) and “smoking + obesity + high blood pressure” (O/E =  2). The first cluster rose to 3.5 after the intervention while the second one fell to 1.66. In the control group, the two clusters increased. In the aggregation of two risk factors, obesity and hypertension were the highest in the intervention and control groups (O/E =  2), and they both decreased in the intervention and control groups after assessment.

**Table 2:** Observed and expected prevalence of NCDs risk factors and their clusters at pre-and post-assessment in the intervention and control groups among employees, Tunisia (2009–2014).

	S	PI	UD	O	HBP	Intervention group						Control group					
						Pre			Post			Pre			Post		
						O (%)	E (%)	O/E	O (%)	E (%)	O/E	O (%)	E (%)	O/E	O (%)	E (%)	O/E
**0**	–	–	–	–	–	5.9	5.6	1.05	10.3	8.4	1.27	9.5	8.8	1.07	12.5	9.9	1.26
**1**	+	–	–	–	–	4.7	3.7	1.27	8.7	5.0	1.74	5.4	4.1	1.31	5.9	4.4	1.34
	–	+	–	–	–	14.0	14.4	0.97	11.2	13.7	0.82	16.5	19.5	0.84	11.9	13.2	0.90
	–	–	+	–	–	4.3	6.2	0.69	4.8	7.7	0.62	5.9	5.7	1.03	4.4	6.0	0.73
	–	–	–	+	–	0.8	1.0	0.80	2.9	2.1	1.38	2.8	2.1	1.33	3.8	3.7	1.03
	–	–	–	–	+	0.9	1.2	0.75	1.4	1.4	1.00	1.4	1.6	0.87	2.7	3.5	0.77
**2**	+	+	–	–	–	8.6	9.3	0.92	6.6	8.2	0.80	10.5	9.0	1.16	4.7	5.8	0.81
	+	–	+	–	–	5.1	4.0	1.27	3.8	4.6	0.82	0.9	2.6	0.35	2.1	2.7	0.78
	+	–	–	+	–	0.3	0.6	0.50	0.2	1.3	0.15	0.8	1.0	0.80	1.2	1.6	0.75
	+	–	–	–	+	0.9	0.8	1.12	0.7	0.8	0.87	0.7	0.7	1.00	2.5	1.5	1.67
	–	+	+	–	–	17.0	16.0	1.06	14.9	12.7	1.17	16.1	5.7	2.82	10.7	8.0	1.34
	–	+	–	+	–	3.0	2.5	1.20	4.0	3.6	1.11	3.2	4.8	0.67	4.8	5.0	0.96
	–	+	–	–	+	2.6	3.1	0.83	1.5	2.3	0.65	2.3	3.4	0.68	2.1	4.6	0.45
	–	–	+	+	–	0.3	1.1	0.27	0.7	2.0	0.35	1.1	1.4	0.78	1.6	2.3	0.69
	–	–	+	–	+	1.4	1.4	1.00	1.0	1.3	0.77	0.7	1.0	0.70	1.3	2.1	0.62
	–	–	–	+	+	0.4	0.2	2.00	0.6	0.4	1.50	0.8	0.4	2.00	1.9	1.3	1.46
**3**	+	+	+	–	–	11.5	10.2	1.13	10.2	7.6	1.34	4.7	5.8	2.00	4	3.5	1.14
	–	+	+	+	–	2.6	2.8	0.93	3.9	3.3	1.18	3.3	3.0	1.10	3.3	3.0	1.10
	–	–	+	+	+	0.3	0.2	1.50	0.4	0.3	1.33	0.4	0.2	0.20	0.3	0.8	0.37
	+	–	+	–	+	0.6	0.9	0.67	0.4	0.8	0.50	0.4	0.5	0.80	1.0	0.9	1.11
	+	–	–	+	+	0.2	0.1	2.00	0.7	0.2	3.50	0.1	0.2	0.50	1.0	0.5	2.00
	–	+	–	+	+	1.3	0.6	2.17	1.0	0.6	1.66	1.2	0.8	1.50	3.6	1.7	2.12
	+	–	+	+	–	0.6	0.7	0.86	0.7	1.2	0.58	0.4	0.6	0.67	0.1	1.0	0.10
	+	+	–	+	–	0.8	1.6	0.50	0.7	2.1	0.33	2.9	2.2	1.32	0.8	2.2	0.36
	–	+	+	–	+	2.5	3.5	0.71	1.9	2.1	0.90	1.9	2.2	0.86	2.1	2.8	0.75
	+	+	–	–	+	2.0	2.0	1.00	0.4	1.3	0.30	1.5	1.6	0.94	1.7	2.0	0.85
**4**	–	+	+	+	+	1.8	0.6	3.00	1.0	0.5	2.00	0.5	0.5	1.00	1.6	1.1	1.45
	+	–	+	+	+	0.2	0.1	2.00	0.1	0.2	0.50	0.0	0.1	0.00	0.3	0.3	1.00
	+	+	–	+	+	0.1	0.3	0.33	0.5	0.3	1.66	1.1	0.4	2.75	1.1	0.8	1.37
	+	+	+	–	+	2.2	2.3	0.96	1.0	1.2	0.83	0.8	5.8	0.14	1.9	1.2	1.58
	+	+	+	+	–	1.0	1.8	0.55	1.5	2.0	0.75	0.7	1.4	0.50	0.9	1.3	0.69
**5**	+	+	+	+	+	0.7	0.4	1.75	0.7	0.3	2.33	0.1	0.2	0.50	0.8	0.5	1.60

This is the Table 1 legend.

a S: Smoking.

b PI: Physical Inactivity.

c UD: Unhealthy Diet.

d Ob: Obesity.

e HBP: High Blood Pressure.

Table 3 presents the prevalence and the prevalence odds ratios (POR) of combinations of two risk factors regardless of the exposure to the other three risk factors in the intervention group and the control group at pre-and post-assessment. At baseline, obesity was clustered with high blood pressure among employees in both the intervention group and the control group. The calculated PORs, 2.67 (CI95%:1.72–4.09) and 2.02 (CI95%:1.31–3.12), were the highest, indicating that the presence of these behaviors significantly increased the occurrence of the other behaviors by 2.67 and 2.02, in the intervention group and the control group respectively. However, smoking was inversely clustered with obesity and lack of physical activity at pre- and post-assessment. Indeed, non-smoking employees were more likely to be obese and physically inactive. The post-assessment evaluation indicated that the clustering of obesity and hypertension increased from POR = 2.67 (CI95%:1.72–4.09) to POR = 3.01 (CI95%:2.04–4.39) at pre-and post-intervention, respectively, in the intervention group. The same trend was observed for the clustering of unhealthy diet and physical inactivity, increasing from POR = 1.29 (CI95%:0.96–1.74) to POR = 2.78 (CI95%:2.15–3.60), at pre-and post-intervention, respectively.

**Table 3:** Prevalence (P) and Prevalence Odds Ratios (PORs) of Simultaneous Occurrence of Two NCDs Risk Factors at pre-and post-assessment in the intervention and control groups in Sousse, Tunisia (2009–2014).

	Intervention group				Control group			
	Pre-assessment		Post-assessment		Pre-assessment		Post-assessment	
	P %	POR (CI_95%_)	P %	POR (CI_95%_)	P %	POR (CI_95%_)	P %	POR (CI_95%_)
**Obesity and hypertension**	4.9	2.67 (1.72–4.09)	5.5	3.01 (2.04–4.39)	4.6	2.02 (1.31–3.12)	10.7	2.41 (1.78–3.26)
**Obesity and smoking**	4.1	0.53 (0.34–0.81)	5.6	0.55 (0.39–0.76)	6.4	1.03 (0.70–1.52)	6.4	0.63 (0.46–0.86)
**Obesity and unhealthy diet**	8.0	0.99 (0.68–1.44)	9.5	0.92 (0.68–1.24)	6.8	0.76 (0.53–1.10)	9.1	0.51 (0.38–0.68)
**Obesity and physical inactivity**	11.7	1.42 (0.92–2.20)	14.0	1.34 (0.98–1.83)	13.4	0.94 (0.65–1.36)	17.3	1.39 (1.04–1.85)
**Hypertension and smoking**	6.8	0.88 (0.62–1.26)	4.9	0.83 (0.58–1.19)	5.1	1.21 (0.80–1.85)	10.3	1.72 (1.28–2.32)
**Hypertension and unhealthy diet**	9.6	0.98 (0.69–1.38)	7.0	1.07 (0.76–1.51)	5.2	0.80 (0.53–1.19)	9.7	0.94 (0.70–1.26)
**Hypertension and physical inactivity**	12.7	0.94 (0.64–1.37)	8.6	0.93 (0.65–1.31)	10.7	1.09 (0.72–1.65)	14.8	0.99 (0.74–1.31)
**Smoking and physical inactivity**	26.5	0.68 (0.50–0.92)	22.0	0.78 (0.61–1.01)	22.8	1.19 (0.86–1.66)	16.2	0.81 (0.62–1.07)
**Smoking and unhealthy diet**	21.8	1.18 (0.90–1.55)	18.4	1.09 (0.85–1.39)	8.2	0.44 (0.31–0.61)	11.4	0.99 (0.75–1.31)
**Unhealthy diet and physical inactivity**	39.3	1.29 (0.96–1.74)	35.4	2.78 (2.15–3.60)	29.2	1.69 (1.23–2.32)	26.0	2.35 (1.79–3.10)

The changes in the prevalence of risk factors clusters for chronic diseases in relation to the socio-demographic characteristics are presented in Table 4. For the entire sample, the prevalence of having the aggregation of two risk factors decreased significantly from 40.1% at pre-assessment to 34.5% (p = 0.001) at post-assessment in the intervention group. However, the prevalence of no and one risk factor increased from 30.8% to 39.7% (p = 0.001). In the control group, the prevalence of having two risk factors slightly decreased from 37.6% at pre-assessment to 33.4% at post-assessment, and the prevalence of having more than three risk factors increased from 20.7% to 25.2% (p = 0.2). After stratification according to age, the prevalence of having the two-risk-factors cluster and more than three risk factors decreased in the two age groups involving participants over 30 years and those less than 30 years. This decrease was more pronounced in the employees aged more than 30 years. It was, from 37.0% and 44.9%, respectively for the two-risk-factors cluster and more than three risk factors, respectively at pre-assessment to 31.3% and 26.9%, respectively at post-assessment (p ≤ 10^−3^). In the control group, the rate of the two-risk-factors cluster decreased in the two age groups, but it was not a significant decrease. Regarding gender, a sharp decrease, from 37.8% to 30.2% (p ≤ 10^−3^), was noted in the two-risk-factors cluster, from 37.8% to 30.2% (p ≤ 10^−3^) in the intervention group. For the female participants, a non-significant decrease was noted for the two-risk-factors cluster. In the control group, the prevalence of having the aggregation of two risk factors decreased, but not significantly. After stratification based on education, the prevalence of having more than three risk factors decreased in all the categories in the intervention group. However, this decrease was significant only among the employees who did not finish their primary school and the rate dropped from 31.2% at pre-assessment to 21.1% at post-assessment (p ≤ 10^−3^). Yet, in the control group, the prevalence of having more than three risk factors increased significantly, in the employees having an intermediate or secondary school level. It was from 19.4% to 25.8% (p = 0.02) at pre- and post-assessment, respectively. In the intervention group, the prevalence of having the two-risk-factors cluster and more than three risk factors significantly decreased among married employees. This decrease was from 39% at pre-assessment to 33.2% at post-assessment (p = 0.005). In the control group, the rate decreased among employees with and without partners, but not significantly. With regard to the type of work, the prevalence of the two-risk-factors cluster and more than three risk factors significantly decreased in blue collars in both the intervention and control groups. The decrease was from 41.5% at pre-assessment to 35.1% at post-assessment and from 28.2% at pre-assessment to 25.3% at post-assessment. Stratification according to occupational seniority showed a decrease in the prevalence of the two-risk-factors cluster and more than three risk factors in all the categories in the intervention group. The decrease was significant only in employees having a job experience between three and ten years. In the control group, no significant changes were noted in the prevalence of the two-risk-factors cluster and more than three risk factors with regard to occupational seniority. Concerning perceived health, a significant decrease was noted in the prevalence of the two-risk-factors cluster among employees who rated their health as good or very good. The decrease was from 41.3% at pre-assessment to 29% at post-assessment (p < 10^−3^). However, the decrease was more pronounced in the prevalence of more than three risk factors among employees who reported intermediate health status; it was; from 34.8% at pre-assessment to 26.1% at post-assessment (p = 0.01). In the control group, no significant changes were noted in the prevalence of the two-risk-factors cluster and more than three risk factors with respect to perceived health status. In the intervention group, the prevalence of the two-risk-factors cluster significantly decreased in the participants believing in health improvement; it was from 40.7% at pre-assessment to 34.4% at post-assessment (p < 10^−3^). No significant changes were noted in the control group. In the intervention group, the prevalence of the two-risk-factors cluster significantly decreased only in the participants who were not diagnosed with at least one chronic disease, including cardiovascular diseases, diabetes, colorectal cancer, breast cancer, lung cancer, and ear-nose-throat cancer; it was; from 41% at pre-assessment to 35.3% at post- assessment (p = 0.001). In the control group, the decrease was not significant. In the intervention group, the prevalence of the two-risk-factors cluster decreased in both employees with and without health insurance. The change was significant only among employees with insurance; it was from 38.1% at pre-assessment to 34.7% at post-assessment (p = 0.002). In addition, a non-significant increase was noted in the prevalence of more than three risk factors among employees without insurance; it was from 24.3% at pre-assessment to 25.6% at post-assessment.

**Table 4:** Evolution of the prevalence of risk factors clusters for NCDs before and after the intervention according to the employees’ socio-demographic characteristics in the intervention and control groups in Sousse, Tunisia (2009–2014).

Variable		Intervention group							Control group						
		Pre n (%)			Post n (%)				Pre n (%)			Post n (%)			
		0–1	2	≥3	0–1	2	≥3	p	0–1	2	≥3	0–1	2	≥3	p
**Total**		255 (30.8)	332 (40.1)	241 (29.1)	416 (39.7)	362 (34.5)	271 (25.8)	**0.001**	297 (41.8)	267 (37.6)	147 (20.7)	392 (41.4)	316 (33.4)	239 (25.2)	0.20
**Gender**	Male	150 (27.1)	208 (37.6)	195 (35.3)	261 (38.0)	207 (30.2)	218 (31.8)	**<10** ^**−**3^	165 (38.4)	149 (34.7)	116 (27.0)	212 (36.0)	193 (32.8)	184 (31.2)	0.33
	Female	105 (38.2)	124 (45.1)	46 (16.7)	155 (42.7)	155 (42.7)	53 (14.6)	0.48	132 (47.0)	118 (42.0)	31 (11.0)	180 (50.3)	123 (34.4)	55 (15.4)	0.08
**Age (years)**	≤30	229 (33.1)	284 (41.0)	179 (25.9)	332 (39.2)	299 (35.3)	217 (25.6)	**0.027**	195 (42.5)	178 (38.8)	86 (18.7)	241 (44.4)	180 (33.1)	122 (22.5)	0.13
	>30	23 (18.1)	47 (37.0)	57 (44.9)	84 (41.8)	63 (31.3)	54 (26.9)	**<10** ^**−**3^	95 (39.1)	87 (35.8)	61 (25.1)	151 (37.4)	136 (33.7)	117 (29.0)	0.56
**Education level**	Illiterate/Primary school	66 (24.8)	117 (44.0)	83 (31.2)	142 (46.9)	97 (32.0)	64 (21.1)	**<10** ^**−**3^	26 (32.1)	32 (39.5)	23 (28.4)	60 (42.9)	50 (35.7)	30 (21.4)	0.25
	Intermediate/Secondary school	139 (34.2)	165 (40.6)	102 (25.1)	216 (38.3)	201 (35.6)	147 (26.1)	0.25	235 (43.4)	201 (37.2)	105 (19.4)	293 (41.5)	231 (32.7)	182 (25.8)	0.02
	University/master’s degree	46 (31.3)	47 (32.0)	54 (36.7)	58 (32.0)	64 (35.4)	59 (32.6)	0.70	32 (43.8)	24 (32.9)	17 (23.3)	39 (38.6)	35 (34.7)	27 (26.7)	0.77
**Marital status**	Single/ Widower/divorced	131 (31.3)	174 (41.5)	114 (27.2)	150 (39.3)	141 (36.9)	91 (23.8)	0.06	109 (44.1)	95 (38.5)	43 (17.4)	110 (45.1)	82 (33.6)	52 (21.3)	0.20
	Married	120 (30.0)	156 (39.0)	124 (31.0)	265 (39.8)	221 (33.2)	179 (26.9)	**0.005**	183 (40.0)	171 (37.4)	103 (22.5)	279 (40.0)	233 (33.4)	186 (26.6)	0.27
**Occupation**	White collars	65 (32.7)	69 (34.7)	65 (32.7)	95 (38.8)	81 (33.1)	69 (28.2)	0.37	40 (40.4)	28 (38.3)	31 (31.3)	69 (41.8)	48 (29.1)	48 (29.1)	0.93
	Blue collars	187 (30.3)	256 (41.5)	174 (28.2)	315 (39.6)	279 (35.1)	201 (25.3)	**0.001**	250 (43.0)	222 (38.2)	109 (18.8)	283 (40.0)	241 (34.1)	183 (25.9)	0.009
**Occupational seniority (year)**	≤2	98 (29.3)	145 (43.4)	91 (27.2)	75 (34.7)	72 (33.3)	69 (31.9)	0.06	76 (44.7)	69 (40.6)	25 (14.7)	34 (37.8)	34 (37.8)	22 (24.4)	0.14
	3–10	103 (31.8)	119 (36.7)	102 (31.5)	206 (40.2)	177 (34.6)	129 (25.2)	**0.03**	71 (40.3)	67 (38.1)	38 (21.6)	98 (48.8)	61 (30.3)	42 (20.9)	0.20
	>10	52 (33.3)	62 (39.7)	42 (26.9)	135 (42.1)	113 (35.2)	73 (22.7)	0.18	139 (41.6)	117 (35.0)	78 (23.4)	260 (39.6)	221 (33.7)	175 (26.7)	0.52

Table 5 presents the changes in chronic disease clusters in the prevalence of risk factors among the employees“according to health characteristics.

**Table 5:** Evolution of the prevalence of risk factors clusters for NCDs before and after the intervention according to the employees’ health characteristics in the intervention and control groups in Sousse, Tunisia (2009–2014).

Variable		Intervention group							Control group						
		Pre-assessment n (%)			Post-assessment n (%)				Pre-assessment n (%)			Post-assessment n (%)			
		0–1	2	≥3	0–1	2	≥3	p	0–1	2	≥3	0–1	2	≥3	p
**Self-Perceived health**	Very bad/Bad	18 (34.0)	23 (43.4)	12 (22.6)	42 (40.0)	39 (37.1)	24 (22.9)	0.70	12 (37.5)	9 (28.1)	11 (34.4)	23 (32.9)	19 (27.1)	28 (40.0)	0.84
	Intermediate	80 (26.8)	115 (38.5)	104 (34.8)	158 (35.6)	170 (38.3)	116 (26.1)	**0.01**	78 (40.6)	70 (36.5)	44 (22.9)	146 (39.2)	131 (35.2)	95 (25.5)	0.79
	Good/Very good	134 (31.1)	178 (41.3)	119 (27.6)	184 (42.3)	126 (29.0)	125 (28.7)	**<10** ^**−**3^	180 (41.4)	168 (38.6)	87 (20.0)	205 (44.6)	149 (32.4)	106 (23.0)	0.14
	Excellent	22 (61.1)	9 (25.0)	5 (13.9)	28 (47.5)	27 (45.8)	4 (6.8)	0.10	24 (55.8)	16 (37.2)	3 (7.0)	18 (43.9)	15 (36.6)	8 (19.5)	0.21
**Believe in health improvement**	Disagree	24 (30.4)	30 (38.0)	25 (31.6)	11 (34.4)	11 (34.4)	10 (31.3)	0.90	10 (31.3)	17 (53.1)	5 (15.6)	17 (35.4)	14 (29.2)	17 (35.4)	0.06
	Agree	225 (30.5)	300 (40.7)	213 (28.9)	402 (39.8)	347 (34.4)	261 (25.8)	**<10** ^**−**3^	280 (42.4)	242 (36.6)	139 (21.0)	368 (41.6)	296 (33.4)	221 (25.0)	0.16
**Chronic diseases** *	No	240 (31.8)	309 (41.0)	205 (27.2)	391 (40.4)	342 (35.3)	236 (24.4)	**0.001**	281 (42.8)	247 (37.7)	128 (19.5)	359 (43.4)	283 (34.2)	186 (22.5)	0.25
	Yes	15 (20.3)	23 (31.1)	36 (48.6)	25 (31.3)	20 (25.0)	35 (43.8)	0.28	16 (29.1)	20 (36.4)	19 (34.5)	33 (27.7)	33 (27.7)	53 (44.5)	0.39
**Health insurance**	Yes	195 (31.0)	240 (38.1)	195 (31.0)	380 (39.5)	333 (34.7)	248 (25.8)	**0.002**	232 (41.5)	203 (36.3)	124 (22.2	361 (40.6)	299 (33.6)	229 (25.8)	0.27
	No	58 (31.4)	82 (44.3)	45 (24.3)	32 (39.0)	29 (35.4)	21 (25.6)	0.34	61 (42.7)	60 (42.0)	22 (15.4)	27 (54.0)	14 (28.0)	9 (18.0)	0.21

*The included chronic diseases were as follows: cardiovascular diseases, diabetes, colorectal cancer, breast cancer, lung cancer, and ear-nose-throat cancer.

Table 6 presents the changes in the prevalence of risk factors clusters for chronic diseases with respect to health characteristics. In the intervention group, multiple logistic regression analyses revealed that males were more likely to have a risk score of three or more (OR = 2.38, p < 10^**−**3^) than females. The employees aged more than 30 years were more likely to have a risk score of three or more versus zero or one (OR = 1.39, p = 0.019) than the younger employees. The same was noted in the employees with less than two years of work experience compared to those having more than 10 years of work experience (OR = 1.71, p = 0.006) and those diagnosed with at least one chronic disease (OR = 2.44, p < 10^−3^). The employees who reported an intermediate perceived health were considerably more likely to have a risk score of three or more versus zero or one risk factor (OR = 5.16, p = p < 10^−3^) than those who reported excellent perceived health. Overall, the odds ratios for the comparisons of two versus zero or one risk factor were lower compared to those of three or four versus zero or one risk factor. The odds ratios for the two factor comparisons revealed that the employees working for less than two years were more likely to have two risk factors versus zero or one risk factor than those working for over 10 years (OR = 1.53, p = 0.015). The employees who reported intermediate perceived health were slightly more likely to have a risk score of two versus zero or one risk factor than those who reported an excellent perceived health (OR = 1.70, p = 0.026). The intervention showed a significant protective effect in the intervention group in reducing the likelihood of the multiple risk factors occurring with OR = 0.70 (p = 0.018) and OR = 0.74 (p = 0.002) for risk scores two and three or more, respectively. In the control group, the intervention had no significant effect in the stepwise method for the two-risk factor aggregation (OR = 0.87, p = 0.255). Furthermore, the time factor increased the likelihood of the risk score three or more, but not significantly (OR = 1.12, p = 0.378).

**Table 6:** Ordinal regression analysis of multiple risk factors among employees in the intervention and control groups in Sousse, Tunisia, 2009–2014, with 0 or 1 risk factor as the reference category.

Variables	Intervention group		Control group	
	OR (CI_95%_)	P	OR (CI_95%_)	P
**Intervention effect**	0.81 (0.68–0.97)	0.025	1.01 (0.84–1.21)	0.937
**Gender**	1.62 (1.35–1.96)	<10^−3^	1.96 (1.62–2.37)	<10^−3^
**Age (years) > 30** (≤30)	1.28 (1.05–1.55)	0.016	–	–
**Occupational seniority (year)** (>10)				
≤2	1.51 (1.15–1.98)	0.003	–	–
3–10	1.25 (0.99–1.57)	0.051	–	–
Chronic diseases* **yes** (no)	2.10 (1.52–2.90)	<10^−3^	2.05 (1.52–2.77)	<10^−3^
**Perceived health** (excellent)				
Very bad/Bad	2.07 (1.37–3.13)	0.008	2.45 (1.41–4.26)	0.001
Intermediate	2.59 (1.7–3.93)	<10^−3^	1.67 (1.07–2.60)	0.023
Good/Very good	1.95 (1.19–3.20)	0.001	1.39 (0.90–2.14)	0.131

*The included chronic diseases were as follows: cardiovascular diseases, diabetes, colorectal cancer, breast cancer, lung cancer, and ear-nose-throat cancer.

## Discussion

One of the main findings in the present study was the decrease in the rate of employees having two or more risk factors in the intervention group and the increase in the rate of employees with two or more risk factors in the control group. These results clearly illustrate the need to adopt a new paradigm of research to have greater effect on public health with reduced health care costs. A growing body of literature has outlined several attempts to modify multiple lifestyle-related risk factors on a population scale, and it has demonstrated the feasibility and potential effectiveness of this approach [33]. In addition, multiple unhealthy behaviors are often present simultaneously. It is therefore important to study the clustering of lifestyle risk factors because of the possible synergistic effects on health. Indeed, knowing the clustering tendency of the risk factors and targeting change in multiple health behaviors are the key approaches to an effective prevention of chronic diseases [34,35]. There is some evidence that combinations of lifestyle risk factors are more detrimental to people’s health than what can be expected from the added individual effects alone [23,31], suggesting that the health effects of lifestyle risk factors are multiplicative rather than cumulative. The fact that multiple risk factors are the norm in the adult population strongly argues in favor of multiple-behavior interventions rather than single-behavior interventions [24,32]. Compared to single-behavior interventions, multiple-behavior interventions may not only have a much greater impact on public health [35], but they may also be more effective and efficient in achieving these goals [26]. Additionally, the workplace presents a suitable environment to prevent multiple risk factors, and this could be advantageous for both employees and employers [9].

When changes in the number of NCD risk factors are adjusted according to the employees’ characteristics, improvements in the adherence to the five recommendations were found to increase, particularly among male participants, those over 30 years, and among workers. Thus, the determining factors of the employees’ response to the intervention were questioned. This trend could be partly explained by the perception of being at risk among older employees and the close association between the adherence rate and the type of job. According to Hanlon et al. [36], the perception of being at risk improves the responsiveness to such workplace interventions. Thus, it would be logical to target those who perceive themselves to need health changes, and who worry about the information given to them. The positive effect among males rather than females prompts us to wonder about the mechanisms responsible for the disproportionate change according to gender. Further research is needed to identify this pattern. Indeed, the POR values from the cluster analysis indicated that unhealthy diet was associated with physical inactivity and that obesity was associated with hypertension, independently of the exposure to other risk factors, both pre- and post-assessment in the intervention and control groups. However, the combination “physical inactivity, unhealthy diet, obesity, high blood pressure” was 300% higher than what would be expected if the factors were independent (O/E = 3) at baseline, and it slightly decreased at post-assessment. However, this combination increased significantly in the control group. Although the improvement in the clusters of NCD risk factors is unlikely to be statistically significant, we believe that the results reported in the present study are important because this workplace health promotion program was able to identify the employees at risk and to provide access to health promotion activities to improve health. These features imply the need for deeper structural changes of environmental dietary and physical activity to tackle the obesogenic and hypertensive environment. Indeed, combining environmental interventions in the worksites [37] with a multi-sectoral approach would be the cornerstone of an efficient and cost-effective action for the prevention of NCD risk factors. Food industry may play a key role in addressing NCDs pattern through food reformulation, consumer information, responsible marketing, and accepting public private partnership [38]. The North Karelia Project, raising the slogan of ‘Health in All Policies’ [39], is a benchmarking example of a successful multi-sectoral prevention project of non-communicable diseases. It evolved from a demonstration project to a national policy, involving health and other services, schools, non-governmental organizations (NGOs), innovative media campaigns, local media, supermarkets, food industry, agriculture, etc. Indeed, a strategy to sustain an economic environment that can offer citizens a better quality of life is an absolute need since many interventions addressing poverty and development proved to have great potential on the risk and prevalence of NCDs [40]. Our attempts to make structural and environmental changes were limited by the political instability following the political and social events of 2010–2011 [41]. The fight against non-communicable diseases and their risk factors was not one of the priorities and challenges taken by the authorities and policy makers at the Ministry of Health and other sectors [42].

### Implications for clustering of risk factors for NCDs

This study suggests a novel approach that considers the interplay of risk variables in analyzing the impact of interventions on a variety of health behaviors rather than examining the effectiveness of intervention programs by reducing individual risk factors. The clusters or patterns of harmful behaviors in adult populations and the associated socio-demographic characteristics have been highlighted in numerous earlier studies about a variety of health behaviors [43]. However, the influence of intervention studies on these clusters and the compliance with healthy lifestyles and health standards have received less attention in the public health literature [44].

### Predictors for change in the clustering of NCD risk factors

The multinomial logistic model suggested five independent variables determining the clustering of risk habits in the intervention group which include male participants who were more than 30 years of age, having less than two years work experience, having lower self-perceived health, and having at least one chronic disease. Large study population from the 2003 Health Survey for England, Poortinga [16] reported that men belonging to lower social class households, who were single, and who were economically inactive are more likely to have a higher number of lifestyle risk factors. However, older age groups and homeowners are less likely to have a higher number of lifestyle risk factors. Pronk et al. [45] reported the prevalence and cluster patterns of multiple healthy lifestyle factors among a random sample from a large Midwestern health plan. The assessed lifestyle-related health factors were physical activity, nonsmoking, high-quality diet, healthy weight, and alcohol consumption. Only 10.8% of adults were found to meet all the five behavior-related factors. For adults, who were in the 50- to 64-year-old cohort (OR1.46, p0.05), having a college degree (OR1.65; p 0.05), and having no chronic disease (OR1.92; p 0.05) were associated with an increased likelihood to be in adherence to multiple healthy lifestyle factors. Raitakari et al. [43] studied the occurrence of common lifestyle risk factors, such as unhealthy diet, smoking, physical inactivity, and alcohol use in a cohort of 484 young adults aged between 18 and 24 years. The authors investigated the predictive factors for risk habit clustering, including male sex, aggressiveness, and past unemployment. Paying a lot of attention to health habits, higher education (being a student), good self-perceived health, and a high sense of responsibility seem to be protective factors against the clustering of risk habits. The 2007 China Chronic Disease and Risk Factor Surveillance provided data with regard to NCD risk factors in a nationally representative sample of 49 247 Chinese aged 15 to 69 years. The risk factors were as follows: tobacco use, excessive alcohol drinking, insufficient intake of vegetables and fruits, physical inactivity, and overweight or obesity. In their study, Yichong Li et al. [46] reported that 57% of a Chinese population have at least two lifestyle risk factors and the mean number of NCDs risk factors is 1.80 (95% CI: 1.78–1.83). Being a Chinese male of older age, living in rural areas, and having a lower education level and a lower yearly household income were found to increase the likelihood of having higher NCD risk factors. Although the study design used was a quasi-experimental one, as in several other international interventions [21,47], it is possible to affirm that the change and evolution of the risk factors was due to the intervention because the workplace is generally a stable environment. Indeed, the same participants were assessed at the start and at the end of the intervention. However, the results of the multinomial multilevel regression model demonstrated the advantage of the present intervention that was more intensive and interactive. It proved to have a significant protective effect in reducing the likelihood of the multiple risk factors occurrence in the intervention group, with ORa = 0.70 (C.I95%: [0.56–0.88]; p = 0.018) and ORa = 0.74 (C.I95%: [0.57–0.95]; p = 0.002) for the risk scores two and three or more, respectively. In the control group, the adjusted ORs were not significant, which demonstrated the effect of the present intervention. The results of the present study showed that the three-year workplace-based intervention was more effective than usual actions in reducing the co-occurrence and aggregation of multiple NCD risk factors. The project in its current form can therefore be recommended for implementation in the worksites while taking into consideration all the cultural and individual needs that affect the clustering pattern of multiple NCD risk factors. Furthermore, this study can be considered a leading one in terms of describing the clustering phenomenon in a representative sample that considers the interaction between the risk factors in order to investigate the effectiveness of the intervention on the cluster of NCD risk factors, and to explore the predictors of the risk factors associated with NCDs in a Tunisian working population. To the best of the authors’ knowledge, this research is one of the fewest studies using an intervention scheme in Tunisia and it is the first to offer multi-component interventions targeting multiple NCD risk factors in a workplace setting. Although the co-occurrence of these risk factors is associated with further increased risk of morbidity and mortality [48], the majority of health promotion interventions typically address them as separate entities [49,50]. Workplaces have the potential to create in-built social support, i.e., active collaboration of the employees in making sustainable changes to attain a healthy lifestyle [51], they can therefore reduce the degree of individual effort and motivation needed to make behavioral changes. Thus, the changes in lifestyle achieved at work are thought to be sustainable in the long term. Most lifestyle programs often focus on short-term behavioral change. Indeed, only a few studies have a follow-up assessment beyond one-year post-baseline. However, only longer-term behavioral lifestyle changes can have a lasting impact on individual health [52]. The existence of a comparison group was another strength in the present study. Indeed, comparing results with those of a control group would further strengthen the assumption that the intervention program was effective in reducing cardiovascular diseases risk [53]. Despite the innovative approach of this intervention program in Tunisia, this study has some limitations that need to be considered.

For feasibility and convenience, a quasi-experimental, non-randomized design was adopted with an added control group to strengthen the findings. Nevertheless, using a quasi-experimental nonequivalent control group design usually creates less compelling support for counterfactual interferences compared to a randomized controlled trial, which might limit the ability to illuminate causal inference [54]. Moreover, it does not provide certitude that the improvement in the targeted risk factors is the result of the intervention. Independent groups were compared at pre-and post-assessment and some socio-demographic characteristics were different. Individual-natural changes in NCD risk factors could therefore have been missed in the present approach. To control this statistically, a multivariate analysis was therefore conducted. In addition, the sample included in the present study was not truly population-based but rather a convenience sample. Although this may limit generalizability to the general population, the results reported in the present study would be broadly applicable to worksite intervention programs involving middle-aged professionals. However, the findings could not be generalized to all worksites in Sousse as employees outside the industrial sector were not considered. Moreover, reliance on self-report is another limitation, which might have led to social desirability bias. Self-assessment of the studied behaviors could also have led to recall bias. Although we attempted to mitigate potential measurement error by incorporating widely used standardized measures, it was most problematic for the assessment of physical activity and dietary intake, and it might have provided over or underestimates for some individuals. To manage this risk, standardization of data collection was performed by trained physician interviewers. Additionally, the present study could have been subject to selection bias related to the level of motivation with regard to willingness to adopt a healthier lifestyle similar to other healthy-lifestyle promotion programs. The individuals choosing to participate may have been motivated by factors unrelated to this specific program. Furthermore, certain environmental changes did not occur until the last part of the intervention period, which may have represented another limitation. Changes that were easy to make (workshops and educational sessions for promoting a healthy lifestyle) took place quickly and they were directly and entirely performed by the research staff. However, the interventions requiring employer resources (providing healthy food in the companies’ canteens and cafeterias) tended to be delayed, probably due to the cost and the level of administrative approval needed.

## Conclusion

With the limited clinical capacity, a critical approach for addressing NCDs is prevention through policies that reduce unhealthy behaviors and risk factors. The workplace is internationally recognized as a crucial area to tackle these health issues; however, it is often marginalized at the national level. The results in the present study together with the data reported in the literature demonstrate the feasibility and effectiveness of these health promotion programs in preventing cluster patterns. Generalization of these programs is possible by their integration into a national health policy. Health care systems alone are not effective in the fight against chronic diseases and their risk factors. Thus, a holistic approach based on scientific evidence supplemented by partnering across a variety of sectors is strongly needed. Strategies for the prevention and control of NCDs should be implemented at a multi-sectoral level. Stakeholders and policy makers should also be involved to undertake legislative, environmental, and structural changes in addition to community involvement. This could facilitate the collaboration of other sectors that are indirectly involved in health, such as transport, agriculture, education, trade, finance, and social affairs. Meanwhile, the Ministry of Health and the Ministry of Labor should collaborate to promote research on workers’ health needs, particularly by framing special research agendas, giving them priority in national research programs and grant schemes, and fostering practical and participatory research. Making a difference to workplace health requires that employers, government, and health-care professionals operate in harmony with each other. Finally, the civil society and the private sector should share responsibility for health and wellbeing through innovative partnerships, exchanging expertise and knowledge, and mobilizing resources.

## Supporting information

S1 Fig The study design of the workplaces based quasi-experimental study. (PDF)

S1 Appendix Questionnaire. (PDF)

S1 File The “Together in Health” Project for the prevention of chronic non-communicable diseases in Sousse, Tunisia: Description, results, and perspectives. (DOCX)
