# Supplementary material for: Intervention effectiveness in reducing the clustering of non-communicable disease risk factors in the workplace: A quasi-experimental study
Source: PLoS One. 2025 Feb 6;20(2):e0317460. doi: 10.1371/journal.pone.0317460 (PMC11801702; doi:10.1371/journal.pone.0317460)
Supplement: S1 Fig — (PDF) [file pone.0317460.s001.pdf]

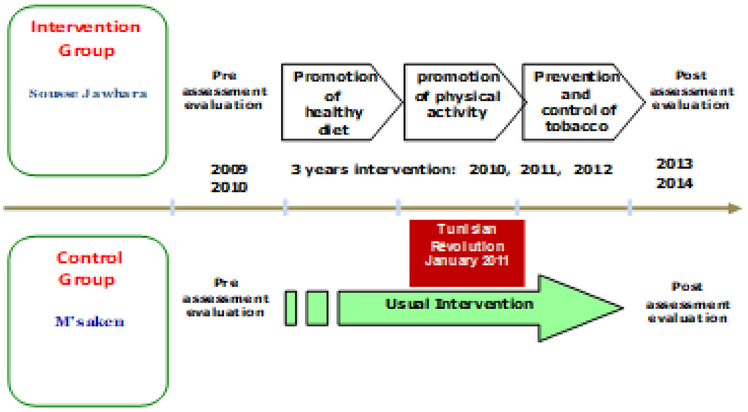

Figure 1: The study design of the workplace based quasi- experimental study: pre-assessment and post-assessment with a control group in the region of Sousse Tunisia, 2009 – 2014
