# Supplementary material for: Intervention effectiveness in reducing the clustering of non-communicable disease risk factors in the workplace: A quasi-experimental study
Source: PLoS One. 2025 Feb 6;20(2):e0317460. doi: 10.1371/journal.pone.0317460 (PMC11801702; doi:10.1371/journal.pone.0317460)
Supplement: S1 Appendix — (PDF) [file pone.0317460.s002.pdf]

استبيان في الوسط المهني

**خاص بالفريق الطبي للدراسة :**	
**المعتمدية:**...	**نوع الإستبيان:**...................
**المؤسسة:**...	**المعرف الوحيد:**...................
	**تاريخ الإستبيان:**...................

بعد التحية و السلام , إن هذا الاستبيان هو أداة لدراسة يقوم بها مركز البحوث للوقاية من الأمراض المزمنة بمستشفى فرحات حشاد بسوسة في إطار برنامج وقائي لتعزيز أنماط حياة صحية و سليمة لذا نرجو منكم الإجابة عنه علما و أن أجوبتكم ستعالج بالسرية المطلقة و المطلوب أن لا تكتبوا إسمكم على هذا الاستبيان و شكرا.

لا تكتب هنا، مساحة خاصة بالفريق الطبي.

1) ضع علامة أمام الاختيار الذي يصف عملك بدقة (أكثر من اختيار)

☐ مدير مؤسسة
☐ نائب مدير مؤسسة
☐ إطار (cadre)
☐ مدير مدرسة إعدادية
☐ أستاذ
☐ موظف إداري
☐ تقني / تقني سامي
☐ عامل
☐ قسم الموارد البشرية
☐ قسم الصحة و السلامة المهنية
☐ قسم الإنتاج
☐ مدير خدمة المطاعم
☐ إجابة أخرى

1 |__|

2) منذ متى تشتغل مع صاحب هذه المؤسسة ؟

☐ أقل من سنة
☐ ما بين السنة و السنتين
☐ ما بين 3 و 10 سنوات
☐ أكثر من 10 سنوات

2 |__|

3) هل تشجعك مؤسستك على أي من الإقتراحات التالية ؟

	نعم	لا	إذا نعم، هل تقدم لك جوائزا تشجيعية؟		إذا نعم، أذكر ماهي ؟
			نعم	لا	
الغذاء الصحي (خضر ، غلال، حبوب كاملة)					
الحفاظ على الوزن المثالي					
القيام بنشاط بدني منتظم					
الوقاية والإقلاع عن التدخين					

3 |__| 4 |__| 5 |__|
6 |__| 7 |__| 8 |__|
9 |__| 10 |__| 11 |__|
12 |__| 13 |__| 14 |__|

4) أي من برامج تعزيز و تطوير أنماط الحياة الصحية التالية يوجد بمؤسستك ؟

	نعم	إذا نعم ضع علامة إذا كنت تشارك في هذا البرنامج	لا	لا أعرف /لست متأكدا	
التغذية الصحية/دروس في التغذية					15 ⊔ 16 ⊔
اختبار وإبداء رأي في تذوق الخضر و الغلال					17 ⊔ 18 ⊔
تظاهرات اجتماعية تتضمن نشاطات متعلقة بالتغذية					19 ⊔ 20 ⊔
مباريات وألعاب متعلقة بالتغذية					21 ⊔ 22 ⊔
برنامج تغذية يُدَرّس في المنزل (مطوية، كتيب...)					23 ⊔ 24 ⊔
برنامج شخصي بخصوص إتباع حمية أو تغذية صحية					25 ⊔ 26 ⊔
نشاطات رياضية جماعية					27 ⊔ 28 ⊔
برنامج شخصي يقدم لك نصائح لممارسة النشاط البدني					29 ⊔ 30 ⊔
القيام بفحص طبي لتقييم حالتك الصحية و البحث عن عوامل إختطار بعض الأمراض					31 ⊔ 32 ⊔
دروس في الإقلاع عن التدخين					33 ⊔ 34 ⊔
برنامج شخصي يقدم لك نصائح في الوقاية والإقلاع عن التدخين					35 ⊔ 36 ⊔
إجابة أخرى أذكر ما هي					37 ⊔ 38 ⊔

39 ⊔

5) هل تتقاسم المعلومات التي تتلقاها في برنامج تطوير أنماط الحياة الصحية بمؤسستك مع عائلتك ؟

☐ نعم ☐ لم أكن حاضرا في هذه البرامج أو لم أتلق أي معلومات

☐ لا

40 ⊔

6) في أيام العمل، عادة أين تتناول فطور الصباح؟

☐ عادة لا أتناول فطور الصباح ☐أتناوله في المنزل أو آتي به من المنزل

☐ أشتريه من مغازة، pâtisserie, بائع متجول، مطعم أو مقهى ☐توفره لي المؤسسة أو أشتريه من مقر العمل(مشربة:Buvette)

41 ⊔

7) في أيام العمل، عادة أين تتناول طعام الغداء ؟

☐ عادة لا أتناول طعام الغداء ☐ أتناوله في المنزل أو آتي به من المنزل

☐ أشتريه من، مغازة، مطعم، بائع متجول،fast food ☐ توفره لي المؤسسة أو أشتريه من مقر العمل(مشربة، مطعم)

42 ⊔

8) هل يوجد في مقر عملك مشربة (Cafétéria) أو مطعم للموظفين؟

☐ نعم

☐ لا [مر إلى السؤال 10] ☐لا أعرف/ لست متأكدا [مر إلى السؤال 10]

9) أجب بنعم أو لا عن الأسئلة التالية بخصوص المشربة (Cafétéria, Buvette) أو المطعم الموجود في مقر عملك والذي تستعمله في أغلب الأوقات:

	نعم	لا	إذا لا، ضع علامة على الاقتراح الذي تظن أنه يجب أن يتوفر	لا أعرف/ لست متأكدا	
هل توفر[المشربة (Cafétéria) أو المطعم] خضر و غلال طازجة يوميا ؟					43 ﺎ 44 ﺎ
هل توفر[المشربة (Cafétéria) أو المطعم] خضر و غلال مطبوخة يوميا؟					45 ﺎ 46 ﺎ
هل توفر[المشربة (Cafétéria) أو المطعم] مشروبات صحية (عصير طازج، مشروبات خالية من السكر، حليب...) يوميا؟					47 ﺎ 48 ﺎ
هل تصنف[المشربة (Cafétéria) أو المطعم]المأكولات الصحية (قليلة الدهنيات، بدون سكر، صحية للقلب...) بواسطة علامات أو لاصقات؟					49 ﺎ 50 ﺎ
هل توفر[المشربة (Cafétéria) أو المطعم] علامات أو لاصقات تصنف القيمة الغذائية للأطعمة الموجودة بها (كمية الحريرات، كمية الدهنيات...)؟					51 ﺎ 52 ﺎ

10) كم مرة يقوم أي من زملائك بالاحتمالاتا التالية ؟

	أبدا	نادرا	أحيانا	عادة	دائما	
يشكر محاولتك إتباع نظام غذائي صحي						53 ﺎ
يشجعك لأكل الخضر أو الغلال						54 ﺎ
يأتيك بغذاء صحي للعمل لتجربه						55 ﺎ
يأتيك بخضر أو غلال للعمل لتجربها						56 ﺎ
يشجعك على تعاطي نشاط بدني						57 ﺎ
يشاركك في القيام بالنشاط البدني						58 ﺎ
ينهاك عن التدخين						59 ﺎ

11) إلى أي مدى توافق أو تعارض الاقتراحات التالية ؟

	أعترض كليا	أعترض إلى حد ما	لا أعترض و لا أوافق	أوافق إلى حد ما	أوافق كليا	
هل تقدر مؤسستك الموظفين الذين يتبعون أنماط الحياة الصحية						60 ﺎ
هل تهتم مؤسستك حقيقة بصحة موظفيها						61 ﺎ
من الواضح أن إدارتك تسعى إلى تحسين صحة موظفيها						62 ﺎ

12) ما هو عنوانك الكامل (بدون إسم) ؟ ....

.... 63 ﺎ

- النهاية -

شكرا للإجابة على هذا الاستبيان
