# Supplementary material for: Intervention effectiveness in reducing the clustering of non-communicable disease risk factors in the workplace: A quasi-experimental study
Source: PLoS One. 2025 Feb 6;20(2):e0317460. doi: 10.1371/journal.pone.0317460 (PMC11801702; doi:10.1371/journal.pone.0317460)
Supplement: S1 File — (DOCX) [file pone.0317460.s003.docx]

i


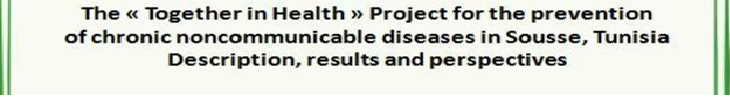


**Summary**

1

2

2

2

2

2

2

2

2

2

2

2

2

2

2

2

2

3

3

3

3

3

3

3

3

.

.

Preface ............................................................................................................1

Epidemiology of chronic noncommunicable diseases....................................3

.2. Brief overview of the problem's burden.........................................................5

.3. Epidemiology of cardiovascular diseases......................................................11

.3.1.

Mortality ...............................................................................................12

Morbidity ..............................................................................................12

Risk factors............................................................................................13

Hypertension.....................................................................................13

Smoking.............................................................................................17

Dyslipidemia......................................................................................19

Diabetes ............................................................................................20

Other risk factors ..............................................................................24

Obesity ..........................................................................................24

Physical inactivity..........................................................................27

Unhealthy diet ..............................................................................27

Excessive alcohol consumption.....................................................28

Social determinants and cardiovascular diseases.........................28

.3.2.

.3.3.

.3.3.1.

.3.3.2.

.3.3.3.

.3.3.4.

.3.3.5.

.3.3.5.1.

.3.3.5.2.

.3.3.5.3.

.3.3.5.4.

.3.3.5.5.

.

Description of the project « Together in Health »........................................29

.1. Introduction, justification of the project « Together in Health » .................29

.2. Intervention guide in school settings............................................................32

.2.1.

.2.2.

.2.3.

.2.4.

.2.5.

Actions for school principals:................................................................38

Actions for teachers:.............................................................................38

Actions for schoolchildren : ..................................................................40

Actions for parents :..............................................................................40

Actions school health team :.................................................................41

i

3

3

3

3

3

3

3

3

3

3

3

3

3

3

3

3

4

4

4

4

4

4

5

5

5

6

7

.3. Intervention guide in work places ................................................................41

.3.1.

.3.2.

.3.3.

.3.4.

Actions for occupational health teams:................................................45

Actions for employers:..........................................................................46

Actions for occupational physicians: ....................................................47

Actions for employees (leaders): ..........................................................48

.4. Intervention guide in community settings....................................................49

.4.1.

.4.2.

.4.3.

.4.4.

Actions for officers and regional directors: ..........................................50

Actions for Non governmental organisations NGOs:............................51

Actions for leaders:...............................................................................53

Actions for mass media:........................................................................53

.5. Intervention guide in health centres ............................................................55

.5.1.

.5.2.

.5.3.

.5.4.

.5.5.

The objectives of intervention in health centres:.................................55

Training programme of primary care physicians ..................................56

Actions for the regional direction of health: ........................................58

Actions for primary care physicians:.....................................................58

Actions for paramedical staff:...............................................................59

.

Methodology and Results of the project « Together in Health ».................60

.1. Methodology.................................................................................................60

.2. Results of the project « Together in Health »...............................................65

.2.1.

.2.2.

.2.3.

Results in school settings......................................................................65

Results in workplace settings................................................................79

Results in community settings ..............................................................93

.

Discussion, conclusion and perspectives ....................................................106

.1. Discussion....................................................................................................106

.2. Conclusion et perspectives: ........................................................................125

.

.

References ..................................................................................................128

Acknowledgement ......................................................................................147

ii

**1**

**.**

**Preface**

According to the World Health Organization (WHO), chronic diseases are

responsible for 36 million deaths in 2008. Chronic diseases known as

non-communicable diseases (NCDs) include mainly cardiovascular

diseases, cancers, diabetes and chronic respiratory diseases. The global

epidemic of NCDs were responsible for 63% of all deaths, including at

least one quarter before the age of 60. The NCD deaths are projected to

increase by 15% overall between 2010 and 2020 (to reach 44 million

deaths). Only 20% of these deaths occurred in high-income countries,

while 80% occurred in the low-income and middle-income countries.

Tunisia is currently undergoing an epidemiological transition

characterized by a decrease in mortality and birth rates, increase in life

expectancy and a decline in infectious diseases compared to chronic

noncommunicable diseases that are constantly increasing. This change

also affects the lifestyle of Tunisians who are adopting the new so-called

"

Western habits" (physical inactivity, changes in dietary habits and

leisure means...). Thus, Tunisia is now facing the pronounced emergence

of NCDs, hence the need to prepare an integrated prevention strategy

to address common risk factors and major determinants shared by

NCDs.

Most of what we know about the prevention of NCDs comes from the

experience of developed countries where many of these diseases have

been the object of successful interventions. Four preventable risk

factors: tobacco use, unhealthy diet, physical inactivity and harmful use

1

of alcohol play a dominant and often synergistic role in the development

of these NCDs. This means that integrated actions against these risk

factors implemented in the social context can lead to the reduction of

major chronic diseases. The available evidence supports the feasibility

and effectiveness of large-scale prevention in the population intended

to increase the proportion of people at low risk for these diseases. The

concept of integrated NCD prevention as an essential component of

existing health system with a focus on health promotion at the

population level could be the way that our country must take to

significantly reduce the future epidemic of these diseases.

The project "Together in Health" is an example of an integrated chronic

disease prevention programme in an intersectoral approach that goes

beyond the health care system. This manuscript aims to describe the

epidemiology of NCDs in Tunisia and around the world and to present

the project "Together in Health" and its results in the region of Sousse in

Tunisia.

2

**2**

**. Epidemiology of chronic noncommunicable diseases**

**.1. Definition, rational of reflection**

**2**

Having a comprehensive and integrated national policy for the

prevention and control of noncommunicable diseases (NCDs) is required

to address common risk factors and major determinants shared by NCDs

(which are cardiovascular disease, diabetes , cancers and chronic

respiratory diseases). Having a comprehensive policy is more

appropriate than to have separate policies for each NCD or even worse

for each individual risk factor or determinant of NCDs, particularly for

developing countries facing a shortage of resources (1).

Develop and implement policy for NCD prevention in a country like ours,

where the transition is compressed in time compared to what happened

in developed countries where the health system has taken the time

needed for adaptation, is a major challenge in itself (2). Most developing

countries have limited capacity to fight against NCDs (3), therefore in

these countries, there is very little experience and expertise in the

development of integrated community response to NCD prevention

programs but the challenge must be met in our country.

Moreover, most of what we know about the prevention of NCDs comes

from the experience of developed countries. For example, the

prediction of absolute cardiovascular risk, which is based on the

calculation of the Framingham risk score was developed in the United

States. It can not be directly applicable to all people in developing

3

countries; therefore, it must be calibrated and validated using new data

from these countries (4).

Fortunately, we have long known that in developed countries, many of

these diseases have benefited from successful interventions (5, 6). In

fact, there is evidence that a small number of risk factors are common

to NCDs. Four preventable risk factors: tobacco use, unhealthy diet,

physical inactivity and harmful use of alcohol play a dominant and often

synergistic role in the development of these chronic problems. This

means that integrated actions against these risk factors implemented in

the social context can lead to the reduction of major NCDs (7, 8). The

available evidence supports the feasibility and effectiveness of large-

scale prevention in the population intended to increase the proportion

of people at low risk of NCDs (9, 10). The concept of integrated NCD

prevention as an essential component of the existing health system with

a focus on health promotion at the population level could be the way

that our country must take to significantly reduce the future epidemic of

these diseases.

So we will emphasize in this monograph on the justification and the

need to launch community based programs to the control of NCDs in the

population.

The model to follow is based on concrete evidence and consists of an

integrated approach to NCD prevention essentially inspired from the

project in North Karelia (11) in Finland. This approach aims to assess the

best way to reduce major risk factors of NCDs through interventions in

4

schools, workplaces, communities and health centers through

multisectoral and integrated approach. This document will also highlight

the merits and limitations of this approach and attempts to identify the

challenges that may be encountered in the implementation of these

programs through the example of our project "Together in Health"

undertaken in the region of Sousse.

**2**

**.2. Brief overview of the problem's burden**

3

6 million people died in 2008 from NCDs mainly from cardiovascular

diseases, stroke, cancer and diabetes mellitus (12). The global epidemic

of NCDs was responsible for 63% of all deaths, including at least one

quarter before the age of 60. The NCD deaths are projected to increase

by 15% overall between 2010 and 2020 (to reach 44 million deaths).

Only 20% of these deaths occurred in high-income countries, while 80%

occurred in the low-income and middle-income countries. The expected

number of deaths from NCDs will increase from 35 million in 2005 to 41

million in 2015, at the same time, years of life lost adjusted for disability

(DALYs) will increase from 725 to 808 million (13) as shown in the

following table:

5

**Table I** : Projected global deaths and DALYs (Adjusted Life Years lost to

disability) of chronic conditions by age 2005-2015

Deaths (million)

005 2015

DALY (million)

2005 2015

220 219

305 349

101 125

2

0

3

6

≥

-29 years

0-59 years

0-69 years

70 years

1,7

1,5

8

7

7

8

20

35

24

41

99

116

all ages

725 808

It is also estimated that in 2030 the number of deaths related to

cardiovascular disease in the world is expected to reach 23 million, with

approximately 85% occurring in countries with low and middle income

(14). According to projections by the World Health Organization, over a

period of ten years (2006-2015), the largest increases in NCD deaths

occur in regions of Africa and the Middle East (12) . The total number of

people with diabetes is expected to increase in the Middle East from 20

million in 2000 to nearly 52.8 million in 2030 and this is the largest

relative increase (163%) of the number of people with diabetes

worldwide (15). Moreover, and according to the International Diabetes

6

Federation (16), in 2007, six of the ten countries with the highest

prevalence of diabetes in the world come from the Middle East mainly

from the Gulf countries.

Tunisia is currently undergoing an epidemiological transition

characterized by a decrease in mortality and birth rates, increase in life

expectancy and a decline in infectious diseases compared to chronic

noncommunicable diseases that are constantly increasing. This change

also affects the lifestyle of Tunisians who are adopting the new so-called

"

Western habits" (physical inactivity, changes in dietary habits and

leisure means...). Thus, Tunisia is now facing the pronounced emergence

of NCDs and because of their multiple determinants, they add to the

burden of disease that will be described in detail later on in the chapter

dedicated to the burden of NCDs in Tunisia. This burden results in an

increase in the economic and social health costs for both the state and

households.

This epidemiological transition raises fundamental questions about

either the selection of health policy, medical practice, dietary behaviors,

the necessary coordination with other sectors such as education, agri-

food and the environment, or the place and role granted to the

individual in order to empower the grip of his own health and diet while

acting on the environment to make easier the choices that protect

health.

A purely curative approach, even the most rational, can not therefore

solve the problem. Hence the importance of a strategy to integrate the

7

fight against non-communicable diseases in a coherent system giving

importance as well to treatment as prevention and health promotion.

This is what the WHO strategy for the fight against NCDs called to by all

member states in the framework of its 2008-2013 action plan

advocating the need for a multisectoral approach (17 ). One of the

major objectives of the WHO strategy for NCD prevention is to promote

interventions to reduce the main shared modifiable risk factors for

noncommunicable diseases: tobacco use, unhealthy diet, physical

inactivity and harmful use of alcohol. This population-based prevention

strategy is to:

**A- Fight against tobacco**

Consider implementing the following set of six cost-effective

interventions (MPOWER) inspired measures to reduce demand in the

WHO Convention Framework on Tobacco Control:

⮚

⮚

⮚

⮚

⮚

⮚

Monitor tobacco use and prevention policies;

Protect people against tobacco smoke;

Offer help to those who want to stop smoking;

Warn against the dangers of smoking;

Enforce the ban on advertising tobacco, promotion and sponsorship;

Raise taxes on tobacco.

**B- Promote a healthy diet**

Apply, without limitation, the measures recommended in the Global

Strategy on Diet, Physical Activity and Health to:

8

⮚

⮚

⮚

To promote and support exclusive breastfeeding for the first six

months of life and promote programs to ensure optimal feeding for

all infants and young children;

Develop a national policy and action plan on food and nutrition with

a focus on national priorities on nutrition, including the fight against

non-communicable diseases related to the nutrition;

To develop and implement guidelines for food and advocate more

healthy foods:

o by reducing the salt content;

o eliminating industrially produced trans fatty acids;

o reducing saturated fatty acids;

o limiting free sugars;

⮚

⮚

Providing consumers with accurate and balanced information to

enable them to make positive health choices;

Prepare and implement, as appropriate, and with all stakeholders, a

framework and / or devices to promote responsible marketing of

food and non-alcoholic beverages to children, in order to reduce

impact of foods high in saturated fats, trans-fatty acids, free sugars,

or salt.

9

**C- Promoting physical activity**

Apply, without limitation, the measures recommended in the Global

Strategy on Diet, Physical Activity and Health to:

⮚

⮚

⮚

to develop and implement national guidelines on physical activity in

favor for health;

implement programs in schools in accordance with the WHO

initiative for health promotion in schools;

ensure that the physical environment promotes active and safe

travel and create spaces for recreational activities in the following

ways:

o ensuring that walking, cycling and other forms of physical

activity are accessible and safe;

o introducing transport policies that promote active and safe

methods of travel to get to school or work, such as walking or

cycling;

o improving sports and recreational facilities;

o increasing the number of safe places available for active

games.

1

0

**2**

**.3. Epidemiology of cardiovascular diseases**

Cardiovascular diseases (CVD) are responsible alone of over 17 million

deaths in 2008 (18), 3 million occurring prematurely before the age of

6

0 years. They are the leading cause of death in developed countries

(19, 20) despite the decline observed over the last 30 years (21, 22).

Their risk factors are well known in most industrialized countries (23, 24)

where effective prevention programs have been established (25, 26).

Very few studies have investigated the extension of the risk factors of

CVD in developing countries (27, 28) which are increasingly affected by

this new flaw.

While communicable diseases are not completely controlled in these

countries, health systems are likely to face a new pathology, that is

chronic and increasingly expensive.

With socio-economic development and health infrastructure, Tunisia is

in the midst of epidemiological transition (29). This transition is

characterized by a decrease in mortality rates, increased life expectancy,

changes in causes of death in favor of chronic non-communicable

diseases, especially cardiovascular disease.

In Tunisia, chronic diseases are responsible of 80% of deaths in 2002

according to the WHO report about the impact of these diseases in the

country (30).

1

1

CVD are considered in our country as a national priority both in research

and in terms of intervention programs. In this context, the need for

scientifically valid and relevant information for decision-making at the

population level and sensitive enough to measure the impact of

interventions is acute.

**2**

**.3.1. Mortality**

Statistics on causes of death in Tunisia in 2009, revealed that MCV ranks

first with 28.9% of all registered deaths (31). In a study of trends in

mortality from ischemic heart disease in Tunisia between 1997 and 2009

(32), the mortality rate due to coronary disease increased by 11.8% for

men and 23.8% for women, resulting in 680 additional coronary deaths

in 2009 compared to the baseline in 1997 and after adjustment for

concurrent changes in population. These increases were explained by a

9

8% deterioration of the cardiovascular risk profile (hypertension and

hypercholesterolemia).

**2**

**.3.2. Morbidity**

In a study that looked at the reasons for hospitalization, in six cardiology

departmets at the District of Tunis during two periods ten years apart

1

992-2002 (33), of 9499 hospitalized patients, the percentage of

ischemic heart disease rose from 39.2% of men and 15.5% of women in

992, to 58.9% and 38.2% respectively in 2002, while rheumatic heart

disease passed from 11.8% in men and 25 3% in women to 4.4% and

1.7% respectively.

1

1

1

2

**2**

**.3.3. Risk factors**

CVD are not just diseases caused by impairment of an organ and the

malfunction of a system; they also reflect individual behavior which in

turn is highly influenced by social class.

The world health report 2002 (8) found that risk factors for the major

cardiovascular diseases and responsible for much of the morbidity and

mortality were hypertension, smoking, hypercholesterolemia, low

consumption of fruits and vegetables, overweight and obesity and

physical inactivity.

**2**

**.3.3.1. Hypertension**

Hypertension is currently recognized as a public health problem

worldwide due to its frequency and risk of cardiovascular disease

attached to it. It would be responsible for approximately 7.5 million

deaths annually or about 12.8% of all deaths worldwide according to

statistics from 2009/2010 (34, 35).

The problem of high blood pressure is a public health priority in Tunisia

by its high frequency, severity of complications and the excessive cost of

its management (monitoring, exploration and drugs). A summary of the

various epidemiological data on population studies is presented in Table

II.

The first survey in this area was that of Gharbi et al (36) in 1988 - 1990.

It focused on a representative sample of urban and rural population of

the Good Cape region (692 men and women aged 35-50 years).

1

3

Hypertension was defined at that time by a systolic blood pressure (SBP)

160 mm Hg and / or diastolic blood pressure (DBP) ≥ 95 mm Hg or

≥

already known and treated. In urban areas, the prevalence of

hypertension was 12, 8% with 11.6% for men and 13.8% among women.

In rural areas, the figures were 8.8%, 4% and 13.3%.

The first survey in the central region of Tunisia was that of Ghannem et

al (37) conducted in 1990 among 555 adults aged 20 and older (60%

women and 40% men), living in the town of Kalaa Kebira (semi-urban

Tunisian Sahel region). Hypertension was defined as a SBP ≥ 140 mmHg

and / or DBP ≥ 90 mm Hg. The prevalence of hypertension was 24.5%

and was slightly higher among women (25.5%) than men (23%) with no

statistically significant difference.

The same team undertook in 1997 an epidemiological survey of a

representative sample of adults aged 20 and older, living in urban areas

in the city of Sousse (n = 957) (38). Hypertension was defined as a SBP ≥

1

40 mmHg and / or DBP ≥ 90 mm Hg. Prevalence of hypertension was

2

8.9%. This prevalence increased with age. There was no significant

difference between men (30%) and women (28.4%).

The team of Ben Romdhane (39) conducted two surveys in five years

intervals, 1996 - 1997 and 2000 - 2001, with two representative samples

of the population of Ariana as part of the monitoring system of CVD. In

total 7608 adults aged 35-70 years were observed: 5771 adults aged 35-

6

4 years have been studied in 1997; the second survey, meanwhile, took

place in 2001 and involved a sample of 1837 adults aged 40-70 years.

1

4

Was considered hypertensive, anyone with an SBP ≥ 140 mmHg and / or

DBP> 90 mm Hg or those claiming to be treated for hypertension and

having a balanced blood pressure. In 1997, 39.6% of men and 49.7% of

women aged 40-64 years of age were hypertensive. In the second

survey, the percentages were 35.9% for men and 46.2% among women,

but the difference was not statistically significant between the two

populations. However, for the two surveys, the prevalence was

consistently higher in women for all age groups and the difference was

-

3

statistically significant (p <10 ). For both sexes, the prevalence was

relatively low before the age of 50 years and increased significantly after

this age of 50.

Ben Romdhane et al (40) also conducted a national study published in

2

011 on a sample of 8007 adults aged 35-70 years. The overall

prevalence of hypertension was 30.6% (33.5% among women and 27.3%

men). Only 38.8% of hypertensive patients knew their diagnosis. Of

these, 84.8% were treated and 24.1% were balanced.

Other studies have been performed in older populations. Laouani et al

(41) found a prevalence of 69.3% among adults aged 60 and over,

Hammami et al (42) showed a prevalence of 52% in a population of

adults aged 65 and over.

1

5

**Table II: The various studies of hypertension in adults in Tunisia**

**Global**

**Prevalence %**

**12, 8**

**Males**

**%**

**Females**

**%**

**Author**

**Study**

**Population**

**Date**

CVD risk factors FRCV

in urban and rural

population of Good

cape

692 males and

females aged 35-

50 yearss

**11,6**

***13,8***

1988

-

(Urban)

(Urban)

*(Urban)*

Gharbi

(36)

**8,8**

**4**

***13,3***

**HBP >160/95**

**mmHg**

1990

(Rural)

(Rural)

*(Rural)*

CVD risk factors in the

commune of Kalaa

Kebira

Ghannem

(37)

555 adults aged

1990

**24,4**

**28,8**

**23**

**30**

**25,5**

***28,4***

***49,7***

20 years and over

Ghannem

(38)

CVD risk factors in

urban city of Sousse

CVD surveillance:

prevalence of risk

factors

957 adults aged

1

997

20 years and over

Ben

1996

-

5

1

771 adults aged

5- 64 years

Romdhane

(39)

____

____

**39,6**

3

1997

2000

-

Ben

CVD surveillance:

ischemic heart disease

and risk factors

TAHINA projet :

Hypertension among

adults in Tunisia,

national study

837 adults aged

40- 70 years

Romdhane

(39)

**35,9**

**27,3**

***46,2***

2001

Ben

Romdhane

(40)

8007 adults qged

35- 70 years

2011

**30,6**

**33,5**

CVD risk factors in

primary care centres

of the district of

Sousse

Laouani

(41)

600 adults aged

2

000

**69,3**

**52**

**37**

**45**

**63**

60 years and over

Prevalence of

Hammami

(42)

598 adults aged

hypertension among

old persons

2011

**55,5**

65 years and over

1

6


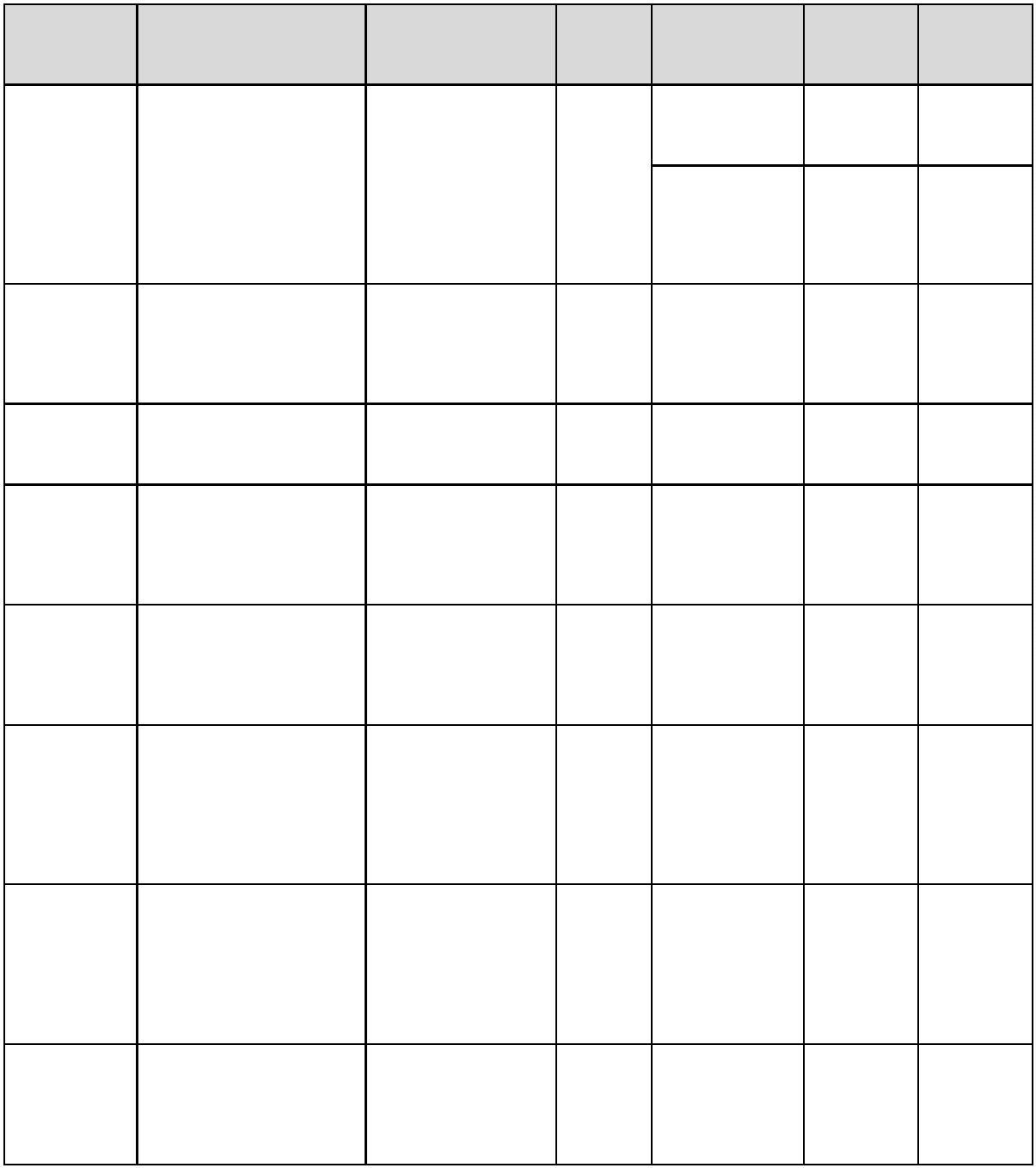


**2**

**.3.3.2. Smoking**

A real public health problem worldwide, the tobacco epidemic killed in

the 20^th^ century, almost 100 million people worldwide, with an increase

of that number to about one billion for the current century (43, 44 ). If

current trends continue, by 2030 tobacco will become the leading cause

of preventable morbidity and mortality worldwide, responsible for more

than 8 million deaths per year (43). The epidemiological data on

smoking among adults in Tunisia are shown in Table III

Among the older surveys on smoking in the general population, a cross-

sectional study was conducted by Ghannem et al (37) in 1990 in a semi-

urban community of Tunisia’s Sahel among 555 persons aged 20 years

and over. The proportion of smokers was 66.5% in men and 0.6% among

women.

Another cross-sectional study was conducted in 1996 of a nationally

representative sample of 5696 subjects aged 25 and over (45). Tobacco

use was reported by 30.4% of the respondents. Smoking was reported

by 55.6% of men and 5.2% of women. Among men, the proportion of

tobacco users diminished with age, in parallel with an increase in

dropout rates. Among women, the proportion of smoking peaked in the

age group 35-54 years. In contrast, the proportion of consuming

traditional tobacco increased with age in both sexes, from 2.4% in men

2

5-34 years to 20.4% among those over 55 years and 0,1% to 14.3% in

women for the same age groups. Smoking was more prevalent in rural

1

7

areas in men with the lowest levels of education and belonging to the

lower social classes.

In urban areas, Ghannem et al (38) noted in the 1997 survey in Sousse,

that smoking was found in 21% of cases. At all ages, men were

significantly more often smokers (61%) than women (4%) (p<0,001).

Among men, the proportion of heavy smokers (more than 20 cigarettes

per day) was 23% of all smokers, and that of light smokers (fewer than

1

0 cigarettes per day) was 31%.

Ben Romdhane H. et al (39) reported that, in men, the percentage of

smokers passed from 51.2% to 46.9%; the non - smokers from 37.3% to

2

5.1% and the ex - smokers from 11.5% to 28% (p <0.001). Among

women, the percentage remained stable at respectively 5.2% and 5.9%

for those who smoke, 94.1% and 92.9% for those who do not smoke and

0

.7% and 1.2% of those who stopped smoking. In both surveys, the

differences between men and women were statistically significant (p

0.001).

<

The National Family and Population office conducted a study in 2001 in

7000 households for people aged over 12 years (46). The overall

prevalence of smoking was 24% with 45.4% for men and 3.8% among

women.

1

8

**Table III : The various studies of smoking among adults in Tunisia**

**Global**

**Males**

**%**

**Females**

**%**

**Author**

**Study**

**Population**

**Date**

**Prevalence %**

CVD risk factors in

the commune of

Kalaa Kebira

555 adults

aged 20 years

and over

Ghannem

(37)

1990

**26,8**

**30,4**

**21**

**66,5**

**55,6**

***0,6***

***5,2***

Smoking in Tunisia :

Behaviours and

knowledges

5696 adults

aged 25 years

and over

Fakhfakh

(45)

1996

1997

9

57 adults

Ghannem

(38)

CVD risk factors in

aged 20 years

and over

**61**

***4***

urban city of Sousse

Ben

Romdhane

(39)

CVD surveillance:

prevalence of risk

factors

5771 adults

aged 35- 64

years

1996

-

____

**51,2**

***5,2***

1997

CVD surveillance:

ischemic heart

disease and risk

factors

Ben

Romdhane

(39)

1837 adults

aged 40- 70

years

2000

-

**46,9**

____

***5,9***

2001

CVD risk factors in

primary care centres

of the district of

Sousse

6

00 adults

**13,3**

Laouani

(41)

***24,5***

aged 60 years

and over

2000

____

(tabagis

me actif)

*(neffa)*

The National

Family and

Population

office (46)

7000

Tunisian study of

family health

households;

persons aged

over 12 years

2

001

**24**

**45,4**

**3,8**

**2**

**.3.3.3. Dyslipidemia**

Hypercholesterolemia is a major risk factor for cardiovascular disease

and is responsible for about a third of deaths from ischemic heart

disease (34). Globally, high cholesterol would be responsible for

approximately 2.3 million deaths annually (18, 34). In 2008, the overall

1

9


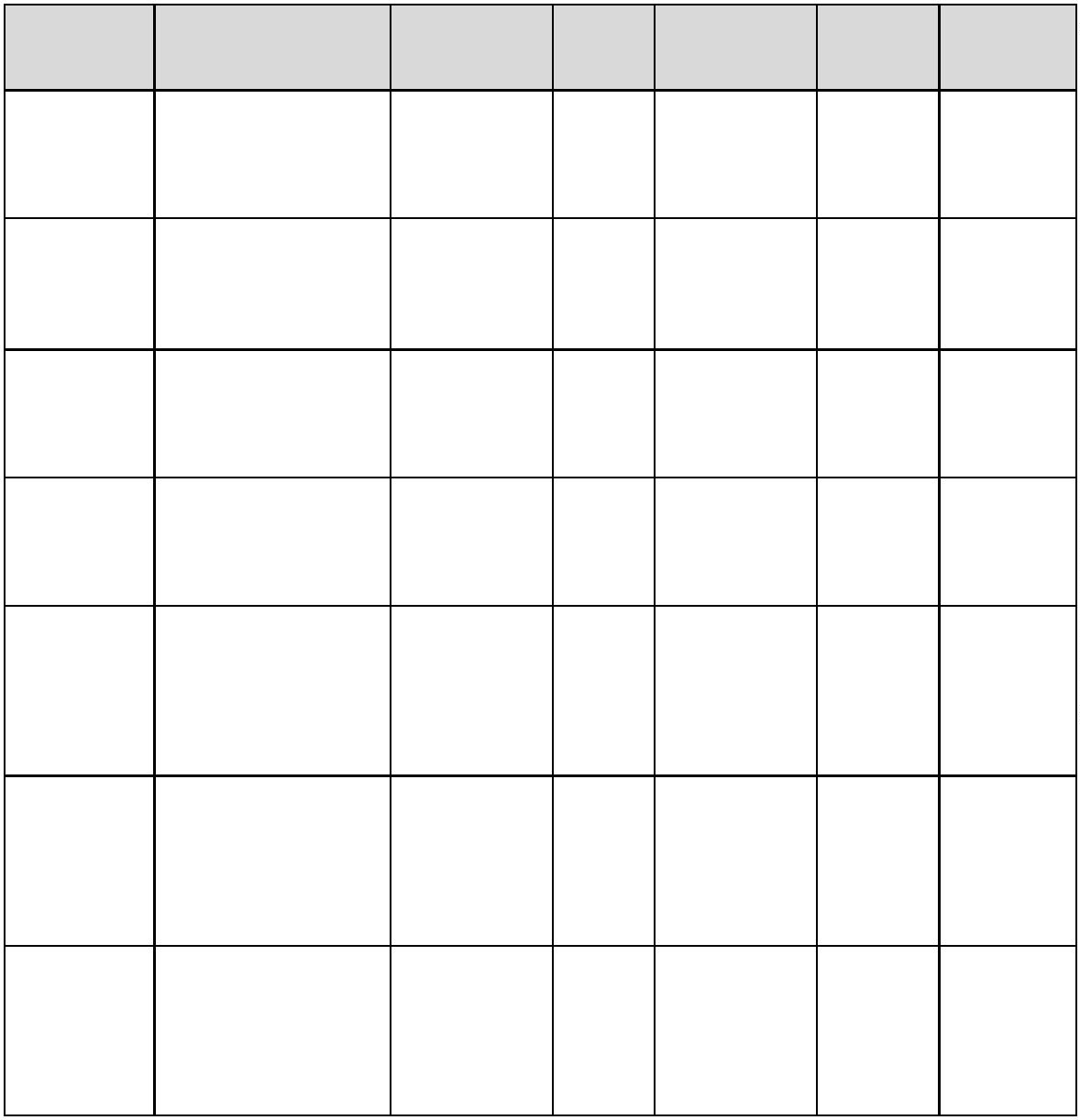


prevalence of high cholesterol in adults - defined as a total cholesterol

level ≥ 6.2 mmol / l (≥240 mg / dl) - was 9.7% (8.5% for men and 10.7%

for women) (35).

In Tunisia, in the study of cardiovascular risk factors in semi-urban areas

in Sousse in 1990 (37), hypercholesterolemia was relatively low in the

general population with about 12%. In the city of Ariana (38) during the

first survey (1997), 12% of men and 17.4% of women had cholesterol

levels ≥ 6.2 mmol/l; 20.9% and 22.2% respectively had borderline

cholesterol levels, between 5.2 and 6.2 mmol/l. In the second survey

(2001), the percentage of those with high cholesterol was 8.7% in men

and 18.2% among women, but the difference was not statistically

significant between the two surveys. During the first survey, 16.6% of

men and 12.3% of women had triglycerides ≥ 2.3 mmol/l. These

percentages were 18.9% and 13.8% for the second survey, but the

differences were not statistically significant between the two surveys. A

2

009 study on the prevalence of dyslipidemia in Tunisia has found a

prevalence of hypercholesterolemia of 8.4% in the adult population (47)

with 21% of hypertriglyceridemia.

**2**

**.3.3.4. Diabetes**

Diabetes mellitus is a common cosmopolitan health problem with a

rapid growth representing a real pandemic according to the

International Diabetes Federation IDF (48). The number of diabetics in

the world is estimated at 366 million in 2011 and is expected to reach

5

52 million in 2030 according to IDF prediction. Among the countries in

2

0

the top 10 of the highest diabetes prevalence, there is 6 countries of the

Arab Middle East region affected (48) (Kuwait, Lebanon, Qatar, Saudi

Arabia, Bahrain and the United Arab Emirates).

In Tunisia, the oldest diabetes study ever conducted was realized in

1

977 and concerned a representative sample of the governorate of

Tunis (49). The total number of people investigated during that survey

was 9712 subjects composed of 45% men and 55% women. The average

age was respectively 24.8 and 26 years. The overall prevalence of

diabetes was 2.3%, half of which was old known diabetic.

In 1990, the prevalence of diabetes (fasting glucose ≥ 7.8 mmol / l) in

the commune of Kalaa Kebira (37) was 6.4%. It was virtually the same in

both sexes.

In the 1997 survey (38) at the city of Sousse, the prevalence of diabetes

increased with age and was present in 10% of the study population.

Regardless of age, women were significantly more often affected than

men with 11% and 7% respectively (p = 0.04).

In both surveys in the region of Ariana (39), the prevalence of diabetes

(fasting glucose ≥ 7.8 mmol / l) was 14.5% for the general population,

1

3% of men and 15% of women. The difference between men and

women was not statistically significant except for the age group 40-49

years for which the prevalence was higher among women. The

prevalence increased significantly starting from 50 years but had not

changed statistically significantly between the two periods.

Gharbi et al (50) conducted a study in the governorate of Nabeul in 692

adults aged 35-50 years. They found an overall prevalence of 7.2%, two-

2

1

thirds were aware of their diabetes diagnosis. This prevalence was

different in urban and rural areas. Indeed, it was respectively 9.3% and

1

0.4% for men and women in urban areas and 2% and 4.5% in rural

areas respectively. In 2007, Bouguerra et al (51) published a study of

3

9

8

729 adults with whom they have found a prevalence of diabetes of

.9% (9.5% in men and 10.1% women). The age adjusted prevalence was

.5% (7.3% for men and 9.6% among women). Among those aged 60

years and over, the prevalence was 23% (41). In a recent study (52) on a

national sample of 7,700 aged 35-70 years in 2005 people, the

prevalence of diabetes was 15.1% (14.1% among women and 16.1%

among men ) with differences between urban and rural areas and

between regions of Tunisia. The epidemiological data on diabetes

among adults in Tunisia are shown in Table IV

2

2

**Table IV : The various studies of diabetes among adults in Tunisia**

**Global**

**Males**

**%**

**Females**

**%**

**Author**

**Study**

**Population**

**Date**

**Prevalence %**

Prevalence of

diabetes in the

Ben Khalifa

(49)

9712 adults

1977

**2,3**

**6,4**

____

**6,4**

**7**

*____*

***6,4***

***11***

gouvernorate of Tunis

CVD risk factors in the

commune of Kalaa

Kebira

555 adults aged

20 years and

over

Ghannem

(37)

1990

1997

9

57 adults aged

Ghannem

(38)

CVD risk factors in

2

0 years and

over

**10**

urban city of Sousse

Ben

Romdhane

(39)

CVD surveillance:

prevalence of risk

factors

5771 adults

aged 35- 64

years

1996

-

**14,5**

**13**

***15***

1997

CVD surveillance:

ischemic heart

disease and risk

factors

Ben

Romdhane

(39)

1837 adults

aged 40- 70

years

2000

-

**15,2**

**15,7**

***14,9***

2001

Prevalence of

diabetes among

adukts in Nabeul

Diabetes study in

Tunisia

**9,3**

**10,4**

Gharbi

(50)

692 adults aged

35- 50 years

2002

2007

**7,2**

**9,9**

**urbain**

**2 rural**

**urbain**

**4,5 rural**

Bouguerra

(51)

3

729 adults

**9,5**

**10,1**

CVD risk factors in

primary care centres

of the district of

Sousse

6

00 adults aged

Laouani

(41)

6

0 years and

over

2000

2005

**23**

**20,4**

**25**

Ben

Romdhane

(52)

7700 adults

aged 35- 70

years

Prevalence of

**15,1%**

**16,1%**

**14,1%**

diabetes in Tunisia

2

3


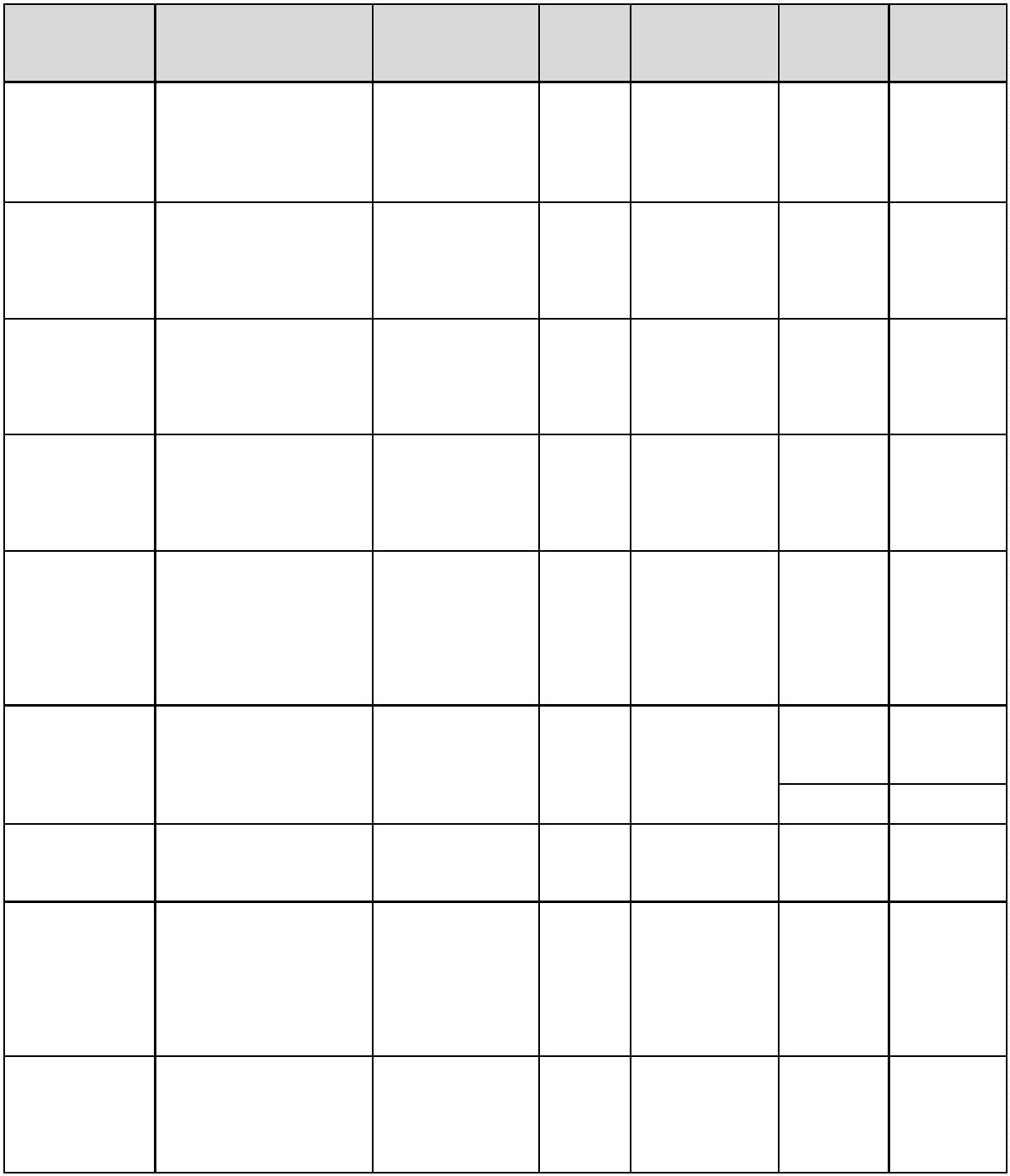


**2**

**.3.3.5. Other risk factors**

**.3.3.5.1. Obesity**

**2**

Obesity is a growing public health problem in both developed and

developing countries (35). It is a cardiovascular risk factor predisposing

to glucose intolerance and diabetes and responsible for an increase of

blood pressure and dyslipidemia (35).

Worldwide, at least 2.8 million people die each year from conditions

related to overweight or obesity (35). In 2008, 34% of adults aged over

2

2

9

0 were overweight with a Body Mass Index BMI (equal or greater than

5 kg/m^2^ : 33.6% of men and 35% of women. For the same year 2008,

,8% of men and 13.8% of women were obese (BMI equal or greater

than 30 kg/m^2^ I), compared with 4.8% for men and 7.9% for women in

1

980 (35).

In Tunisia, several epidemiological studies have been devoted to

overweight and obesity. Epidemiological data on adult obesity in Tunisia

are shown in Table V

In the study of the semi urban area of Kalaa Kebira (37), the prevalence

of obesity (BMI ≥ 30) was 20.7%. The prevalence of overweight

(27≤BMI<30) was 18.4%. Moreover, the prevalence of obesity was twice

as high in women than in men for all ages. This difference was

statistically significant.

In the survey conducted in 1997 in the city of Sousse (38), obesity (BMI ≥

3

0) was found in 28% of cases. It was significantly more common among

2

4

-

3

women (34%) than men (12)

(p<10 ). Android or abdominal obesity

was found in 36% of cases. Its prevalence increased with age and was

significantly higher, regardless of age except for those over 70 years, for

-

3

men (50%) than women (30%) (p < 10 ).

Abdominal obesity was estimated in adults aged 35 to 70 years in the

city of Tunis to 69% among women and 21% among men with a

significant difference (53). The prevalence of metabolic syndrome in this

population was 31.2% (37.3% among women and 23.9% men). Another

study involving 2927 adults aged over 20 years found 9% of men with a

waist circumference greater than 102 cm and 33% of women with a

waist circumference greater than 88 cm (54). Another study in Tunisia

on a sample of 8576 people aged over 15 years (55) reported an overall

prevalence of obesity of 12.2% with a prevalence of 18.3% among

women. In a study of obesity among women aged 20-59 years (56), we

found a prevalence of obesity of 22.6%.

Huge inequalities in the prevalence of obesity according to gender were

found in a Tunisian study in 2005 on a random cluster sample of 2379

men and 2964 women aged between 35 and 70 years. The prevalence of

obesity was higher among women (37%) than men (13.3%) and in urban

areas, but it was lower in subjects with a higher grade level of education

(57).

2

5

**Tableau V: The various studies of diabetes among adults in Tunisia**

**Global**

**Author**

**Study**

**Population**

**Date**

**Males**

**%**

**Females**

**%**

**Prevalence %**

**1**

**8,4**

CVD risk factors in

the commune of

Kalaa Kebira

____

*____*

Ghannem

(37)

555 adults aged

(27 ≤ BMI < 30)

**20,7**

1990

20 years and over

**12**

***26***

(BMI ≥ 30)

1

6 (obesity

in urban

area)

*35,2*

CVD risk factors

FRCV in urban and

rural population

of Good cape

6

92 males and

(obesity in

urban area)

*26,3*

Gharbi

(36)

females aged 35-

50 years

1996

1997

5,4

(obesity in

rural area)

**12**

(obesity in

rural area)

CVD risk factors in

urban city of

Sousse

**28**

Ghannem

(38)

957 adults aged

***34***

(BMI ≥ 30)

(BMI ≥ 30)

20 years and over

(BMI ≥ 30)

**3**

**7,5**

____

*____*

CVD risk factors in

primary care

(27 ≤ BMI < 30)

Laouani

(41)

600 adults aged

2

000

***13,1***

***32,6***

*(27 ≤ BMI <*

*30)*

centres of the

60 years and over

***24,2***

*(27 ≤ BMI*

district of Sousse

(BMI ≥ 30)

*<*

*30)*

Prevalence of

obesity and

overweight

8576 persons

aged 15 years

and over

Kamoun

(55)

Obesity

12,2

2001

6,1

18,3

2

2,6

-

-

-

-

Prevalence of

obesity among

women

BMI ≥30

29,2

Beltaifa

(56)

1849 women

2009

2005

aged 20-59 years

waist

size>88cm

2

379 men and

964 women

aged 35-70 years

El Ati

(57)

Obesity and

gendre

2

25,4%

13,3%

33%

2

6


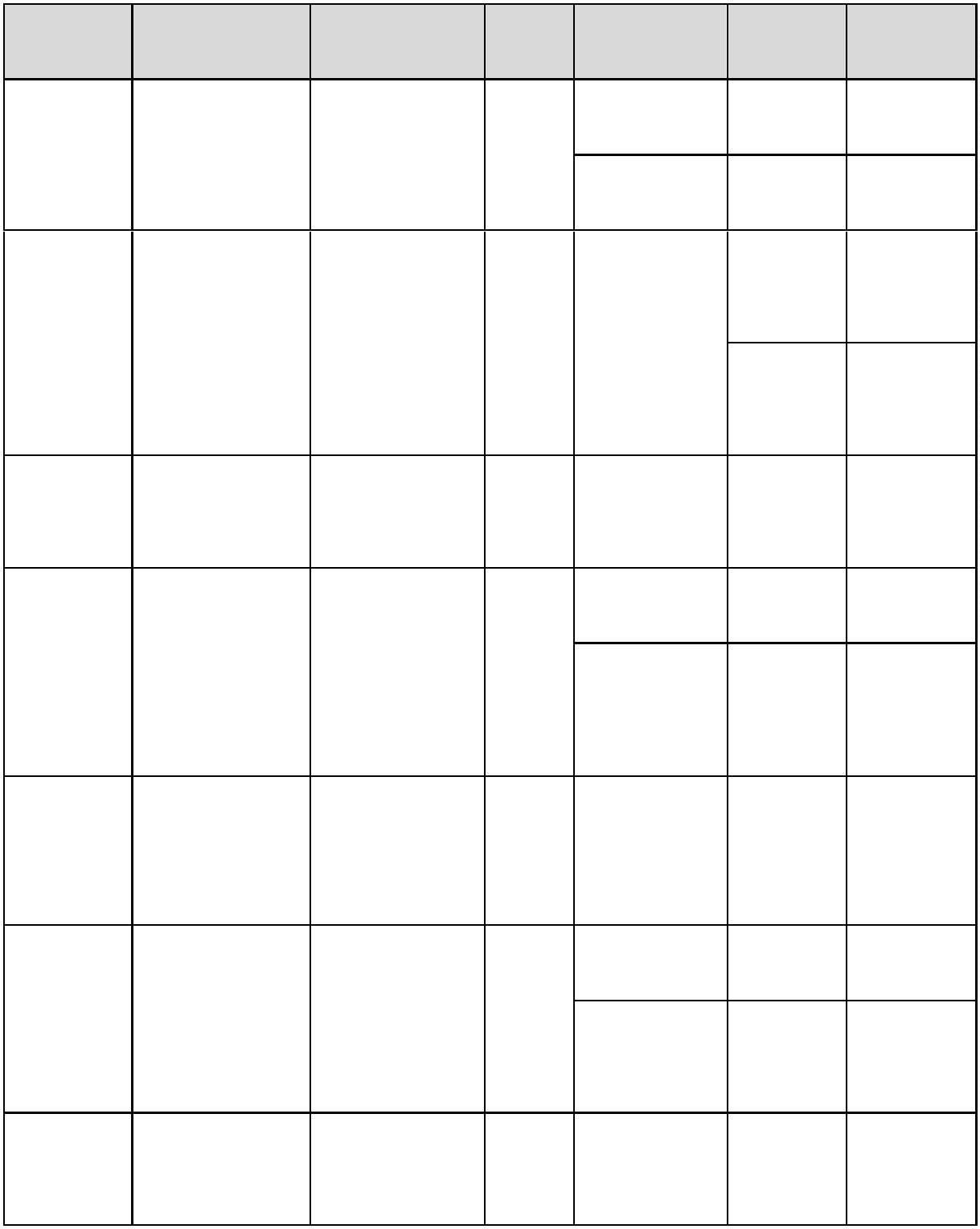


**2**

**.3.3.5.2.**

Insufficient physical activity can be defined as making less than 5 times

0 minutes of moderate activity per week, or less than 3 times 20

**Physical inactivity**

3

minutes of vigorous activity per week, or equivalent. Insufficient

physical activity is the fourth leading risk factor for mortality,

responsible globally of about 3.2 million deaths (18, 34). Very few

epidemiological studies have focused on the study of sedentary or

physical activity in Tunisia because of the difficulty of quantifying these

concepts validly. In a study in 2002-2003 (58) describing the variability

of the data on the prevalence of physical inactivity in 51 countries from

different WHO regions and for different age groups, it was reported for

Tunisia a prevalence of physical inactivity of 11.5% in men and 18.9%

among women in a sample of 4332 people aged 18-69 years. Physical

activity was assessed using the short form of the International Physical

Activity Questionnaire (IPAQ), in which people were asked to indicate

the number of days and duration of severe or moderate activities and

walking activities that they have taken during the last week.

**2**

**.3.3.5.3.**

**Unhealthy diet**

There are a considerable number of evidence regarding the involvement

and role of nutrition in the development of atherosclerosis in general

and in particular cardiovascular disease (34, 35). High dietary intake of

saturated fats, cholesterol, trans fats, salt and low dietary intake of fruit,

vegetables and fish are associated with cardiovascular risk (34, 35).

Obesity appears when there is an imbalance between energy intake in

the diet and energy expenditure. Regular physical activity can help

2

7

prevent obesity by increasing energy expenditure. Approximately 16

million of deaths worldwide (1.0%) and 1.7 million of disability adjusted

life years DALYs (2.8%) are attributable to low fruit and vegetable

consumption (34 ).

**2**

**.3.3.5.4.**

**Excessive alcohol consumption**

Harmful use of alcohol is a risk factor for numerous adverse health and

social outcomes, including hypertension, acute myocardial infarction,

cardiomyopathy, cardiac arrhythmia, liver cirrhosis and other

consequences. Excessive and harmful alcohol consumption was

responsible for 2.5 million deaths (3.8%) in the world in 2004 (18.34).

Very little data are available on excessive alcohol consumption in

Tunisia.

**2**

**.3.3.5.5.**

**Social determinants and cardiovascular**

**diseases**

Social determinants such as social class, income distribution and the

level of education indirectly affect cardiovascular health and overall

health. These determinants play a role in the hierarchy of power and

access to resources and health system. They have a cardiovascular and

metabolic impact because of behavioral risk factors that they condition

(59).

In 2005, WHO convened the Commission on Social Determinants of

Health (60) to provide advice on how to reduce inequalities in health by

fighting against the unequal distribution of power and resources.

2

8

**3**

**. Description of the project « Together in Health »**

**3**

**.1. Introduction, justification of the project « Together in Health »**

Tunisia is currently undergoing an epidemiological transition

characterized by a decrease in mortality and birth rates, increased life

expectancy and a decline in infectious diseases in relation to chronic

diseases that are increasing constantly. This change also affects the

lifestyle of Tunisian who are adopting new Western habits (physical

inactivity, dietary changes and means of leisure ...).

The cardiovascular risk profile is extremely worrying in Tunisia both in

adults and in adolescents. The cardiovascular risk factors increasingly

appear early in life and tend to persist and to be stable over the time. A

cohort study among schoolchildren in the city of Sousse between 1999

and 2003 confirmed the stability of cardiovascular risk factors. Indeed,

the percentage of the stability of blood pressure was 25%, that of

obesity was 48.9% and cholesterol was 59%. These results attest to the

stability of lipids and different vascular risk parameters and impose to

undertake prevention policies targeted at a young age in order to

reduce the burden of morbidity and mortality of cardiovascular disease

in adulthood.

A double burden of disease, with the persistence of perinatal

disease results in greater economic and social cost of health care for

both the state and households. This double burden of disease raises

fundamental questions about either the selection of health policy,

medical practice, dietary behaviors, the necessary coordination with

2

9

other sectors, including education, the agro-food and environment, or

the place and role granted to individuals and their responsibility in the

handling of their own health and diet while acting on the environment

to make it easier the choices that protect health.

It is in this context that the Research Centre for Chronic Disease

Prevention in Sousse attempts to establish an intervention at the

community level to assess its feasibility and effectiveness in the

prevention of major risk factors for chronic disease.

The project, implemented in the city of Sousse aimed to reduce

major risk factors for chronic diseases at the community level: smoking,

unhealth diet and physical inactivity.

The intervention programme intereted the following settings:









School settings

Workplace settings

Health centre settings

Community settings

It is based not only on education through various niches,

including mass media, but also and hopefuly on structural changes in

the environment (fitness trails, bike paths, sports field, smoke free

environment...).

3

0

The center worked in collaboration with different project

partners to implement the program. This monograph proposes, for

information, to describe some of the intervention actions in the

different settings.

These actions were discussed with the partners according to

their feasibility in the context of the city of Sousse. Participants who

work in the field could of course propose further actions that they deem

beneficial.

A multidisciplinary team of teachers, doctors, psychologists,

nutritionists and graphic designers have been working for the center to

3

1


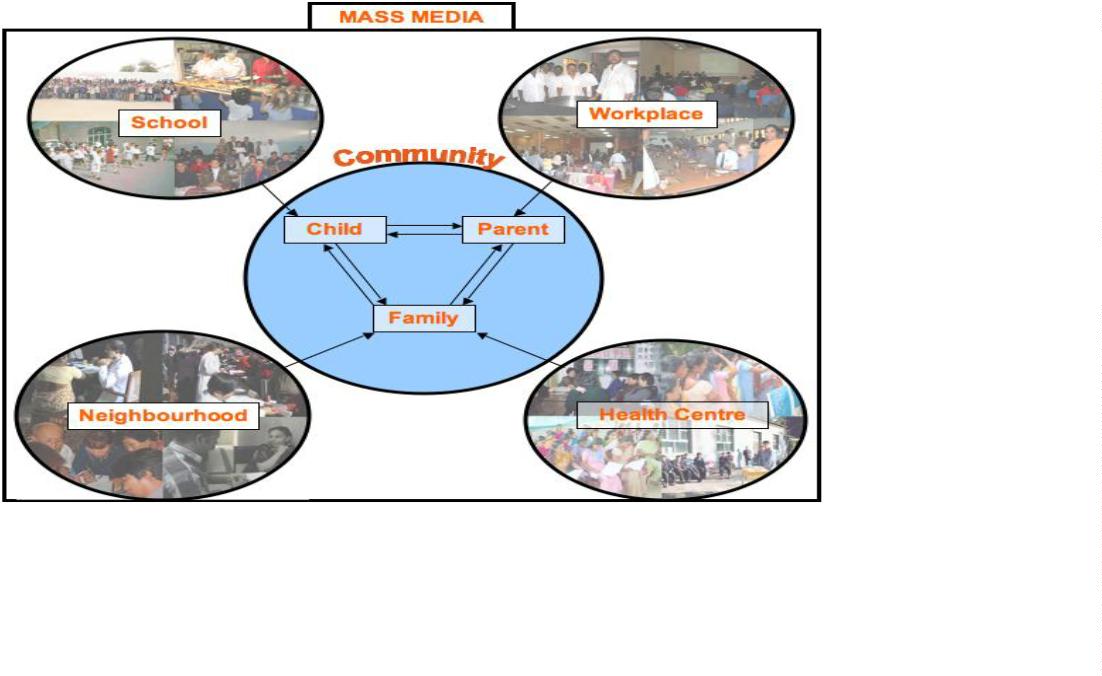

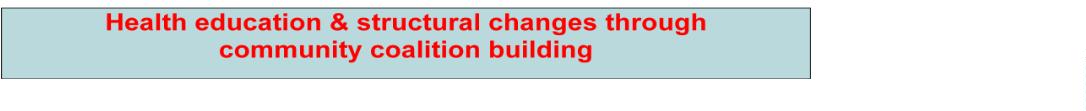


produce guides and intervention tools (such as pamphlets, posters, films

..) adapted to each stakeholder and each sector to serve for the

intervention activities.

.

**3**

**.2. Intervention guide in school settings**

Coordinated programs that involve the school, community and families

can be effective in promoting healthy behaviors, such as eating more

healthy and be more active.

Schools provide a key setting in which children observe and adopt

healthy lifestyles that will last a lifetime. Children spend many hours in

school during their growing years, and it is necessary to give them daily

opportunities to be physically active, eat healthy and develop their self-

esteem to maximize their growth and learning.

Schools can play a key role in promoting healthy lifestyles by following

tracks:

•

Provide schoolchildren with training in nutrition, physical activity,

smoking and body image and self-esteem to help them acquire the

knowledge and skills appropriate to their age as part of a health training

program offered from kindergarten to age 12.

•

Train teachers to focus on the principles and benefits of healthy

eating, physical activity and consequences of smoking and the social

skills that enable schoolchildren to avoid smoking. Enable teachers to

use active learning methods to invest and interest young students in the

school intervention program.

3

2

•

Involve parents in learning activities at home, provide training on the

three factors in the context of parental and school activities and

encourage parents to call for policies and services.

•

Working with public health agencies and voluntary organizations to

the introduction of large school and community programs.

Develop peer leadership and stimulate leadership for schoolchildren.

•

Schools can play an important role in health promotion for the following

reasons:

o All children and young people can be reached.

o Diet, physical activity and smoking are social behaviors

influenced by education, social support and educational

services.

o Training in schools can improve the lifestyles of young

people.

o Role models, such as teachers, instructors and group leaders

can profoundly influence children by example.

o Preventive services and other health services that may be

offered in schools allow for early detection of problems, refer

youth to appropriate resources and offer efficient services.

o All parents can be reached through schools.

The authors of experimental studies have found a number of effects of

education programs for health. It is common to see an increase of

knowledge, less common to see changes in attitudes and uncommon to

3

3

see behavior change. When program effects are observed, we find that

they disappear with time.

Hence the importance of sustaining our actions, begin step by step:

Knowledge then attitudes and behavior. It also requires that actions are

any community-wide and not just in schools.

The chronic disease prevention research centre of Sousse with its

partners, particularly the Regional Direction of Education and the

Regional Commissioner for Youth, Sports and Physical Education have

worked to build a program intervention to promote healthy lifestyles in

schools. The intervention began in the early school year 2010/2011 and

lasted 3 years.

The objective of this intervention is to promote healthy eating, physical

activity and tobacco control. To work on these three factors, the project

team organized training seminars for school doctors, teachers and

administrative staff to make them aware of the importance of the

intervention, how to approach children and information to be sent to

schoolchildren.

We started by developing leaders in each category such as teachers,

administrators, school physicians, schoolchildren and parents that will

have to pass the message through (Figure 1). We made these trainings

for each separate category because each has a special message to

deliver and a particular way of doing it.

Education of leaders about healthy lifestyles, communication and social

skills to allow the message to pass through to different social categories

3

4

namely schoolchildren, teachers, parents and administrative and

medical personnel.

All these categories fell into a cycle of behavior change to move from

the contemplation stage to the action stage and maintenance of a new

behavior. (Figure 2)

3

5

***Leaders***

-

-

-

-

-

Teachers

Administrative personnel

School health team

Scoolchildren

Parents

Increase awareness for the need for change, provide information on the possible

benefits of change

Motivate and encourage the individual to a specific plan or a personal approach

Assist the individual in the development of concrete and possible actions

Assist in providing feedback, problem solving, social support, reward and

reinforcement elements

Assist in the search for alternatives, avoid relapses and misconduct

***The others***

-

-

-

-

-

Teachers

Administrative personnel

School health team

Scoolchildren

Parents

3

6


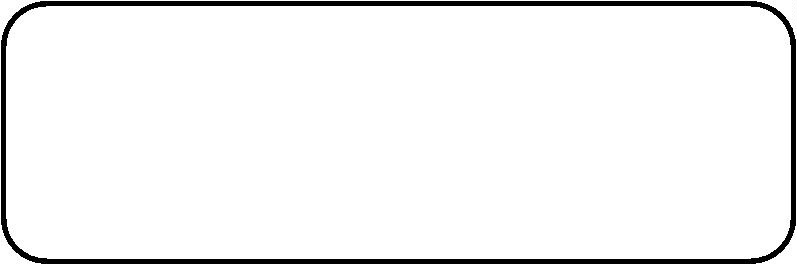

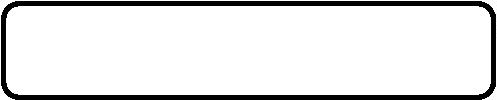

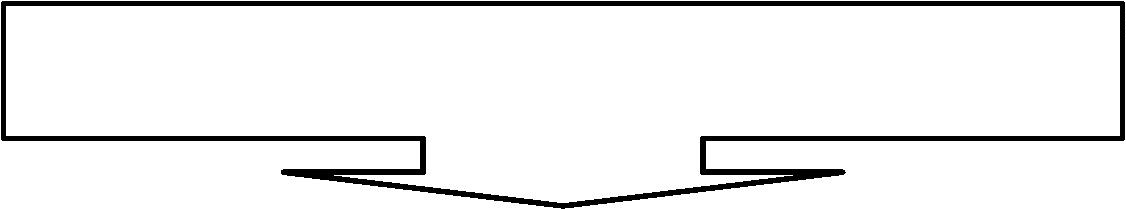

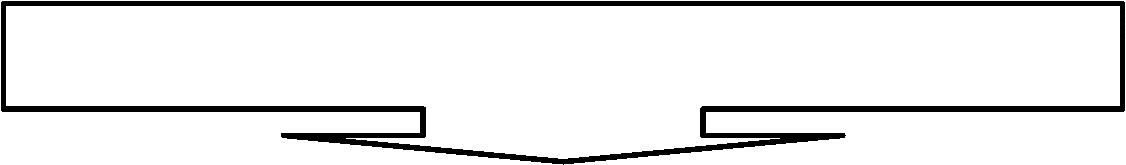

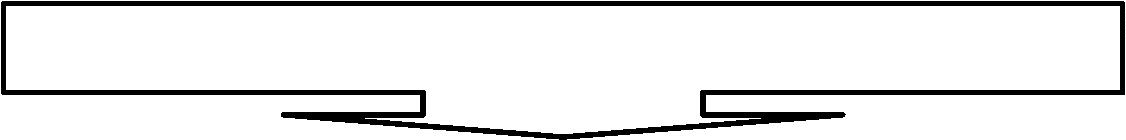

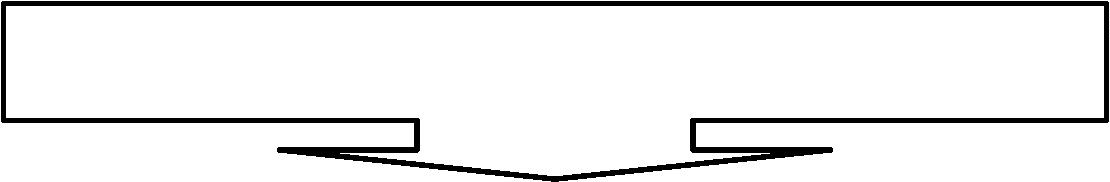

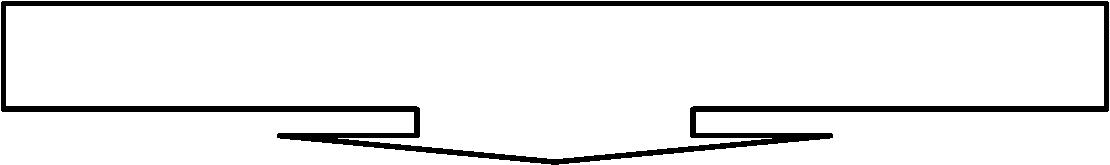

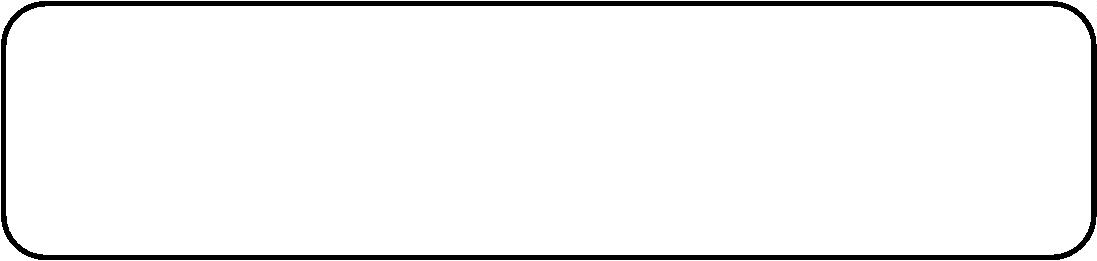

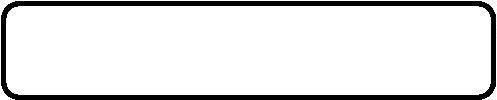


PRECONTEMPLATION

CONTEMPLATION

Teachers and

schoolchildren

Leaders in early

intervention

PREPARATION

MAINTENANCE

ACTION

**Figure 2 : stages of behavior change from leaders to other teachers and**

**students**

Follow-up meetings were scheduled at month 3, month 6 each year with

different groups to document the process and monitor progress and

difficulties encountered.

The project team proposed to support, train and assist different school

stakeholders in the following actions:

3

7


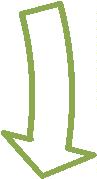

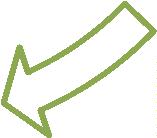

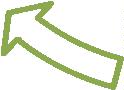

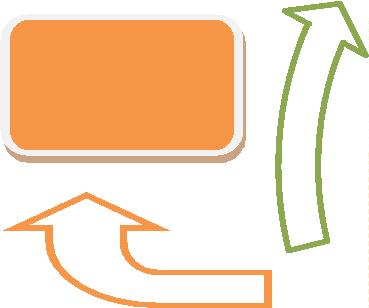

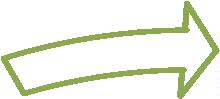

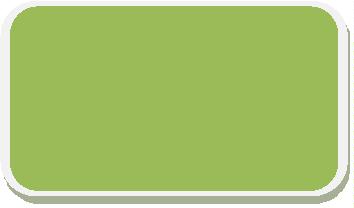

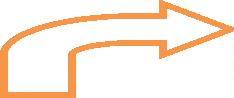


**3**

**.2.1. Actions for school principals:**

-

-

Examine the school environment (eg. Cafeterias, opportunities for

physical activity), develop plans and policies to create and maintain

a healthy school environment and monitor progress.

Participate in programs of nutrition, physical activity and anti

tobacco set up at school at the beginning by the project team.

Thereafter, once leaders are trained, they can maintain these

programs and develop others.

-

-

-

-

-

-

Encourage schoolchildren to participate in events on healthy

lifestyles: taking part in days of tobacco control, sporting events …

Encourage parents to engage in physical activity with their children

(meeting at mid semester and end of the year)

Animation of awareness days and workshops for schoolchildren and

their parents.

Ensure a healthy environment even outside of the school (small

merchants around the school, we can involve them in the program).

Ask teachers to talk about physical activity and active living, healthy

eating and anti tobacco actions in class.

Provide time for teachers and give them the opportunity to

participate in training sessions that focus on healthy lifestyles.

**3**

**.2.2. Actions for teachers:**

-

Build a trust relationship with their schoolchildren, taking into

account their emotional world.

3

8

-

-

Ensure that schoolchildren hear and see the same messages on

nutrition, physical activity and tobacco control.

Incorporate healthy nutrition, physical activity and tobacco control

to other materials, such as using community trails for a field trip

science, write essays in literary materials on different ways to do a

physical activity .

-

Encourage physical activity during physical education sessions, leave

the choice to the schoolchildren of the discipline they prefer to

practice.

-

-

-

Rewarding academic and social efforts of schoolchildren by

providing opportunities for physical activity.

Encourage and congratulate the schoolchildren who are members of

a sports club.

Organize fitness class that combines discipline, but mostly fun and

unwinding after class or during peak hours for schoolchildren and

teachers.

-

-

Lead by example in their feeding behavior, vis-à-vis physical activity

and smoking. Get involved and participate in the school program.

The staff room should be 100% non smoking, as throughout the

whole school, promote physical exercises during breaks and a

healthy diet.

-

Ask The local media to cooperate to deal with school events and

promote healthy eating.

3

9

-

-

Provide opportunities for schoolchildren to explore nutrition,

physical activity, smoking, positive body image and positive self-

esteem with their families, giving them activities to take home.

Assist schoolchildren set goals to improve their behavior, rewarding

new behaviors and celebrate successes.

**3**

**.2.3. Actions for schoolchildren :**

-

-

-

Encourage students to participate as leaders in the project.

Involve students in the program by adopting their ideas.

Encourage students to enroll in a fitness class offered by the school

after class and invite their friends to follow their examples.

Develop socially appropriate behavior by leaders: Do not smoke, eat

dairy products or fruit as a snack, do physical activity at breaks.

Develop students' skills in terms of decision making and the

selection and preparation of food.

-

-

-

-

Encourage students to demonstrate leadership and to handle health

problems.

Set an example of healthy eating, active living and tobacco control

and encourage others to do the same.

**3**

**.2.4. Actions for parents:**

-

Ensure that their child's school offers a program to promote healthy

lifestyles, otherwise make the suggestion to do it for teachers and

administration.

4

0

-

-

Involve parents in learning activities at home, provide training on

nutrition in the context of parental and school activities and

encourage parents to call for policies and services.

Organize meetings and mailings of information for parents

**3**

**.2.5. Actions school health team:**

-

-

Developing the idea and train managers of health promotion in

schools

Teaching teachers to teach nutrition, use of active learning methods

and recognize eating disorders and neglect.

-

-

Ensuring that nutritionists offer on-site services to teachers.

Treating eating disorders in the context of peer support programs

All these actions aim to promote healthy lifestyles in schools with the

collaboration of different stakeholders. These ideas could be developed

by the participants themselves with details to suit the conditions of each

establishment. The project team provides necessary guides for each

category and for each factor mentioned: diet, physical activity and

tobacco control.

**3**

**.3. Intervention guide in work places**

The objectives of intervention in work places were :

4

1







Develop a compelling communication to motivate executives

and other stakeholders to be involved in creating an

environment that promotes and encourages healthy lifestyles.

Encourage community labor to value healthy lifestyles and

develop work environments conducive to healthy eating,

physical activity and tobacco control.

Improve knowledge of workers on healthy lifestyles and risk

factors for cardiovascular disease.

The conditions of success for the development of a program of healthy

lifestyles in the workplace are:



Obtain a formal and clear commitment from the management of

the company for the promotion of healthy lifestyles

Work as a team;







Get the support of all;

Promote activities by various means and in a sustained manner

The three important components of a healthy work environment

elements are: the physical environment, safety and health at work;

hygien and lifestyles; culture of the workplace and a positive

environment.

It is not surprising that leading companies addressed the three elements

of well-being at work on a variety of fronts, ranging from ergonomics to

the feeling of employee control over their work. Most companies

surveyed have implemented detailed security plans, health and welfare

at work to make informed decisions about programs, implementation,

4

2

participant observation, monitoring and evaluation. The main

conclusion was: health in the workplace, it pays.

Occupational health is primordial. The more a person is healthy, the less

he is absent from work and the more he expresses a sense of belonging

towards the organization. He also is more likely to be happy and to

enjoy a good quality of life, both in his professional and personal life.

Each investment in a program of healthy lifestyle generates productivity

gains in firms within 5 years after launch.

Thus, the promotion of healthy lifestyles in the workplace is profitable:

**For employees**

**For the enterprise**

Quality of life at work

Image of entreprise

Relations at work







**To**





Quality of life

**improve**

Stress management

**To**



Psychological well beeing



Sens of belonging

**increase**



Premature mortality risk

Risk of suffering from



certain diseases (mainly

cardiovascular disease,

diabetes, hypertension and

hypercholesterolemia)





Absenteeism

Staff turnover

**To reduce**

4

3


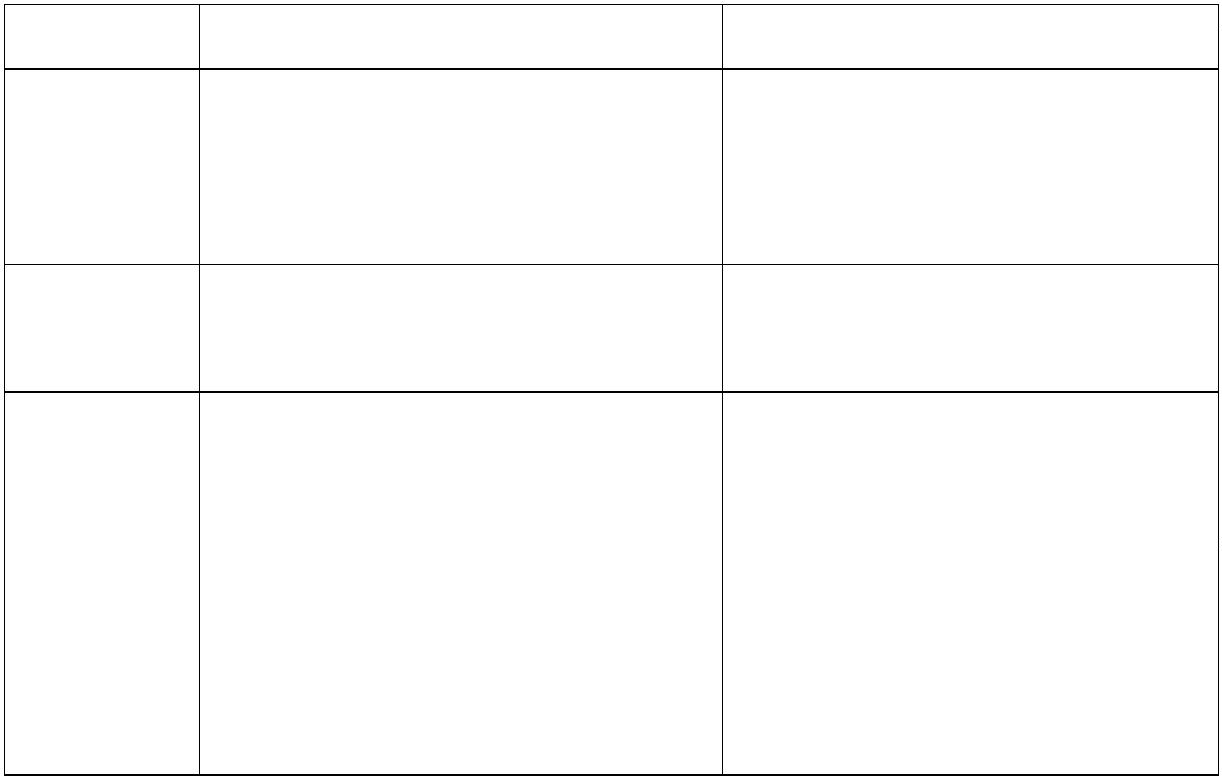


It is for these reasons that the proposed project of prevention of chronic

diseases has included the workplace in education actions on healthy

lifestyles. It is for the interest of employees as well as business leaders.

The chronic disease prevention research centre of Sousse with its

partners, particularly the Department of occupational health at the

University Hospital Farhat Hached of Sousse and the grouping of

Occupational Medicine of Sousse have introduced an intervention

program to promote of healthy lifestyles in the workplace. The

intervention began in September 2010 and lasted three years.

The objective of this intervention was to promote healthy eating,

physical activity and anti tobacco fight in the workplace. To work on

these three factors, the project team in collaboration with the grouping

of Occupational Medicine of Sousse has organized training seminars for

physicians, business managers and employees to make them aware of

importance of intervention for employees but also for business

productivity. The team developed a proposal for a program of training

and intervention that was discussed and approved by the partners and

stakeholders.

The project team proposed to support, train and assist the various

stakeholders in the workplace in the following actions:

4

4

**3**

**.3.1. Actions for occupational health teams:**

-

Develop a compelling communication to motivate executives and

other stakeholders to be involved in creating an environment that

promotes and encourages healthy lifestyles. The workplace offers

several ways to get the attention of employees. It is the

responsibility of the entreprise to determine the best means of

communication, innovate and diversify messages. It is important to

disseminate these messages regularly to arouse and sustain the

interest of most employees.

-

Provide

a

training program for doctors working on the

implementation of a healthy lifestyles program in workplace and

health education in the hiring and supervision visits.

Offer entrepreneurs an intervention program on healthy lifestyles in

the workplace.

-

-

-

Negotiate changes in the work environment that are realistic and

operational

Develop a newsletter for health officials in the workplace. This

newsletter will be sent regularly to companies in the program. One

can find credible and documented articles on lifestyles such as stress

reduction, smoking cessation and maintaining a healthy weight,

nutrition information from registered dietitians to prevent heart

disease, recommendations for physical activity to protect the heart

health.

4

5

-

Involve supervisors, managers and union leaders in the program

promotion "Advertising". Use of multiple communication and

repeated promotions for program "advertising".

**3**

**.3.2. Actions for employers:**

-

-

Helping employers encourage their employees to use a non-

motorized mode of transport for round trip between home and

work, such as walking or cycling if distances permit.

Implement an intervention-based reward if the employee joins the

program and shows an improvement (stops smoking, becomes less

sedentary, loses weight ...), the administration is committed to

reward him according to the means and interests of the company.

Prohibit smoking in the company while ensuring proper smoking

rooms for smokers.

-

-

Providing healthy food in canteens and cafeterias of companies,

identifying the needs of the kitchen staff and the consumption

profile of the clientele.

-

-

-

Provide signage at point of purchase and make available posters,

leaflets available in cafeterias and projection of educational videos.

Ensure individual actions (advocacy, self - assessment of dietary

habits, skill development to change behavior, taste panel ...).

Undertake environmental actions (setting up a committee of

employees, improving the availability and accessibility of healthy

food, posters, table signage at point of service, mascot, etc ...).

4

6

-

-

Stimulate the spirit of mutual support and respect.

Show the organizational commitment to the well-being of its

members or employees.

-

-

Provide training, conferences, demonstrations and special events

that promote the exchange of experiences and inform the

participants on various aspects of healthy lifestyles.

Organize a special event like a sneaker day, a bike ride, a hike, an

open house in a sports center, etc. This type of event also provides

an opportunity for discussion between management and staff.

**3**

**.3.3. Actions for occupational physicians:**

-

Conduct seminars and workshops for employees on healthy

lifestyles.

-

-

Provide smoking cessation consultation in the entreprise.

Conduct regular screening for chronic diseases and risk factors

among all employees while relying on screening algorithms and

treatment.

-

-

Identify barriers in the enterprise that could harm a good diet, the

practice of physical activity, anti tobacco fight and the achievement

of program objectives. Discuss barriers with the administration

project leaders and among employees and try to find practical and

feasible solutions.

Distribution of information materials and disseminating messages.

4

7

**3**

**.3.4. Actions for employees (leaders):**

-

Give environmental activities to an employee committee who should

conduct an analysis of their environment, find solutions and

implement them.

-

-

Improve the knowledge of colleagues on healthy lifestyles.

Promote alternative modes of transport to the car (on foot, by

bicycle)

-

-

Implement a walking club in the company.

Adopt healthy behaviors during breaks and encourage colleagues to

participate.

-

Organize sports tournaments between different departments of the

company or different companies.

-

-

Promote good behavior during breaks and meetings.

Encourage colleagues to adhere to healthy lifestyles programs

promotions introduced by the administration.

-

-

-

-

Form groups to make physical activity (sports, walking faster ...)

Encourage colleagues to bring healthy meals for lunch.

Encourage colleagues to quit smoking and consult for medical help.

Carry out a survey to better understand the interests and needs of

employees for physical activity, providing healthy food and having a

separate space for smokers.

4

8

**3**

**.4. Intervention guide in community settings**

Given the limited success of previous attempts to improve health

through education on lifestyles, the focus of the intervention has shifted

from individual to more holistic approaches that consider health as a

social or community issue, to approaches that address the social

processes that change individual behavior. We now know it is not

enough to put the full responsibility to the person change. Such an

approach blames the victim instead of addressing the social conditions

that cause the harmful behavior. In fact, social or community

interventions can add resources to the set of strategies a person could

have to do the change and improve his health.

It is usual to recognize the limit of the current individual intervention

models for health problem prevention, considering communities as

complex systems, dynamic and adaptive that we can change in order to

influence the healthy lifestyle.

Community initiatives to change the relationship between the individual

and the environment are very promising. It is multidisciplinary

approaches comprising a solid component focused on citizen

participation.

Citizens must interact in their community while addressing certain

special problems and helping their colleagues. As the studied problem

becomes more complex, the level of action becomes more complex

4

9

because people will need more resources, as it is the case of chronic

diseases. In the community we can target the folowing actions:

**3**

**.4.1. Actions for officers and regional directors:**

-

-

-

Increase the availability, accessibility and the safe use of sports

facilities and services offered in the community.

Develop facilities to support regular physical activity outside of

school hours and working practice.

Identify unsafe areas (accidents, injuries) for young people (parks,

recreation centers, etc.) and make the necessary corrections.

Develop green spaces and playgrounds.

-

-

Encourage active transport (walking, cycling) creating security

arrangements (safe routes, road signs, walking trails, bike paths,

police surveillance).

-

Establish and ensure compliance with policies, regulations and

procedures for the promotion of healthy lifestyles:

•

•

Development of fitness trails, bike paths.

Programming and competitive rates for group activities and

sport leisure.

•

Development of neighborhood where services are within

walking or cycling.

•

Design of urbanization based walking and cycling (identified

intersections, pedestrian streets, etc.).

5

0

•

Measures of traffic calming (limit of the speed limit, stop lights,

reducing the width of some streets, etc.).

•

Access to bike track

**3**

**.4.2. Actions for Non governmental organisations NGOs:**

-

Strengthen stakeholder knowledge about the risks of smoking

and the importance of adopting and maintaining healthy

lifestyles in the physical, psychological, intellectual and social

development of young people and the prevention of chronic

diseases;



information on activities, resources, tools available in the

community.



incentives (adjusted prices) for the participation of low-

income citizens in sports activities.

-

-

-

Continue the citizens mobilization and all community

organizations around the optimal development of young people.

Develop a common vision of promoting healthy lifestyle

approach.

Consolidate and develop local and regional collaborative action

plans (community, school, family, youth) to promote the

adoption and maintenance of healthy lifestyles and safe

5

1

behavior (healthy eating, physically active life , tobacco

abstinence)

-

-

-

Continue the pooling of resources, services, programs, activities

already in place in the territory (geographical and sociological).

Integrate advocacy and information for a healthy lifestyle in

activities for youth and their families.

Provide citizens with a diverse range of physical activities and

sport (individual or group) to help them reach the target of at

least 30 minutes of moderate intensity physical activity of (brisk

walking, swimming, etc. ) to high intensity (running, sporting

event, etc.) daily.

-

-

Encourage the development of skills to identify and resist social

influences favorable to smoking.

Support the engagement of individuals in the fight against

smoking by promoting the relationship with non-governmental

organizations fighting against smoking.

Among the participatory evaluation methods we can include the

seasonal calendar of events that these NGOs can use to educate the

community about the importance of chronic disease prevention:











February 4: World fight against cancer day

March 14: World hypertension day

April 6: World physical ativity day.

May 31: World no tobacco day

November 14: World diabetes Day

5

2

**3**

**.4.3. Actions for leaders:**

-

Give environmental activities to a committee of citizens who

should do an analysis of their environment, find solutions and

implement them.

-

-

Improve knowledge of citizens on healthy lifestyles.

Promote alternative modes of transport to the car (on foot, by

bicycle).

-

-

-

-

-

Value the social aspect of sport.

Adopt healthy behaviors and encourage citizens to do it.

Organize sports tournaments between different neighborhoods.

Develop good eating habits at parties and social gatherings.

Encourage citizens to adhere to healthy lifestyles promotion

programs introduced by the municipality.

-

Form groups to make physical activity (sports, fast walking ...).

**3**

**.4.4. Actions for mass media:**

-

Media campaigns against smoking emphasizing the risks of active

and passive smoking (spot TV, radio, magazine articles,

newspapers, ...)

-

-

Frequently raising awareness about the health risks of a fatty

food, sweet and rich in salt.

Organisation of social groups and committees for the promotion

of healthy lifestyle using new information technology (facebook,

twitter ...).

5

3

-

-

-

The use of various media operations, including television, radio,

press, Internet, cinema, on billboards and in the new electronic

media.

Appropriate public relations, including media outreach to the

problems associated with smoking and lack of physical activity to

generate wider media coverage of these issues.

An outreach to various community organizations, such as

schools, religious institutions and civic organizations, and

mobilization; and products such as t-shirts, pins, brochures and

posters that can invite the audience to participate in or attend

certain events, encourage discussion and having a long lasting

presence. These products can play a major role in a campaign of

public awareness if they are carriers of strategic messages and

are widely distributed.

-

-

The broadcast campaigns in the mass media should be

ubiquitous: advertisements must be seen and heard often

enough to generate a change in beliefs, attitudes and behaviors.

Awareness related to a campaign, advertisements or a message

is not enough.

The campaign should include a wide variety of targeted

messages and constantly renewed to motivate different groups

of people trying to quit smoking, to do physical activity and eat a

healthy diet.

5

4

-

A campaign should reflect lessons learned internationally in

identifying the most effective messages and developing creative

strategies. It must be organized and implemented in full

independence, away from the influence of the tobacco industry

and the food and beverage industry.

**3**

**.5. Intervention guide in health centres**

**3**

**.5.1. The objectives of intervention in health centres:**

The objectifs are:

-

Encourage leaders and health professionals and other stakeholders

to be involved in creating an environment that promotes and

encourages healthy lifestyles.

-

-

Improve the knowledge of health professionals about healthy

lifestyles and risk factors for cardiovascular disease.

Encourage health centers to value healthy lifestyles and develop

work environments conducive to healthy eating, physical activity

and tobacco control.

-

To promote the quality of care for chronic diseases (hypertension,

diabetes) in primary and tertiary level of care.

The intervention to promote healthy lifestyle can not be considered

without the involvement of the health sector, seen as a key area of

action in parallel to community actions.

5

5

The chronic disease prevention research centre of Sousse with its

partners, particularly the Departments of Cardiology, Endocrinology,

Pediatrics of the University Hospital Farhat Hached of Sousse and the

regional service of primary health care of Sousse have implemented an

intervention program to promote healthy lifestyles in primary care. The

intervention began in September 2010 and lasted three years. The

objective of this intervention was to promote healthy eating, physical

activity and tobacco control in primary care. To work on these three

factors, the project team hold meetings with partners, as well as training

seminars for primary care physicians, paramedical staff as well as inter-

regional seminars to raise awareness of the importance of intervention

for health professionals.

**3**

**.5.2. Training programme of primary care physicians**

The Department of Epidemiology of the University Hospital Farhat

Hached of Sousse was responsible for the following actions:







Presentation of updated epidemiological data on the

extent of NCDs, cardiovascular diseases and their risk

factors.

Presentation of the results of the pre assessment of

chronic disease risk factors in the districts of Sousse and

Msaken

Presentation of importance of the intervention program

in primary care.

5

6

The Departments of Cardiology, Endocrinology, Pediatrics and

Occupational health of the University Hospital Farhat Hached of Sousse

were responsible of the following actions:



Introduction to international recommendations for the

adoption of healthy lifestyles and encouraging the

medical and paramedical staff to adopt good habits and

be the example to follow.





Presentation of the value of screening for cardiovascular

risk factors (the importance of taking weight, height,

waist circumference, blood pressure and calculate BMI)

Management of patients with cardiovascular risk

according to validated recommendations.





Health education and compliance to treatment

Prevention of obesity and overweight in children.

After the presentation sessions of the intervention, physicians were

required to:







discuss and propose various alternatives for intervention

on promoting healthy lifestyle in primary care.

Share their experience on the feasibility of such an

initiative.

Raise any obstacles and propose appropriate solutions to

provide.

5

7

**3**

**.5.3. Actions for the regional direction of health:**

-

-

Develop a compelling communication to motivate executives

and other stakeholders to be involved in creating an

environment that promotes and encourages healthy lifestyles.

Provide a training program for health professionals on the

implementation of a program of healthy lifestyles in hospitals

and primary care.

-

-

-

Propose to Regional Directors an intervention on healthy

lifestyles in primary care.

Establish, with other organizations and other sectors, a network

aimed at removing obstacles for health promotion.

Design guides for primary care physicians and paramedics

containing algorithms for management of at risk patients.

**3**

**.5.4. Actions for primary care physicians:**

-

-

-

Adhere to the healthy lifestyles program.

Participate in training seminars organized by the project team.

Know the magnitude of chronic diseases and the frequency of

complications such as myocardial infarction, stroke.

Screen adequately for chronic diseases in primary care.

Raise awareness among at-risk patients on the importance of

proper hygiene life

-

-

5

8

-

Educate and inform patients about the importance of promoting

healthy eating and smoking cessation and practice of regular

physical activity.

-

-

-

Get the lifestyle management to all patients and particularly

those at risk.

Design practical guides for hypertensive or diabetic patients on

healthy eating, physical activity and smoking cessation.

Alternate individual and group health education sessions for

patients with cardiovascular risk.

**3**

**.5.5. Actions for paramedical staff:**

-

-

-

To raise awareness about the constantly changing factors, which

determine the emergence of NCDs and cardiovascular disease.

Attend training seminars on healthy lifestyles promotion and

encourage active participation (communication, price ...)

Communicate the concepts taught to patients and encourage

their awareness on the preventive health component.

5

9

**4**

**. Methodology and Results of the project « Together in Health »**

**.1. Methodology**

**.1.1. Study design**

**4**

**4**

To evaluate the effectiveness of an intervention program at the

community level for the prevention of risk factors for noncommunicable

diseases (NCDs) in the region of Sousse in Tunisia, a quasi-experimental

intervention study was conducted in two districts in the governorate of

Sousse. The first distrit community that has served for the intervention

group was represented by delegations of Sousse Jawhara and Sousse

Erriadh. The district community of control was located in the delegation

Msaken. We have included various settings to the evaluation of risk

factors for NCDs in the two groups namely the schools, the workplaces

and the communities. The study design (see below) shows that we have

carried out an evaluation before intervention (pre assessment) in 2009-

2

010. It focused on the knowledge, attitudes and behaviors of

participants in relation to the various studied risk factors that were

represented by diet, physical activity and smoking in the three

environments and for both groups. An intervention was made during 3

years in the three settings of the project intervention area. In the

control area, there's been no specific intervention program from the

usual or routine interventions. The evaluation of these parameters was

made at the end of the intervention (post assessment) in both groups in

2

013-2014.

6

0

**Study design: Quasi-experimental design**

**(Pre - Post assessment with a control group)**

Intervention

Group

**Sousse Jawhara**

**Pre**

**intervention**

**assessment**

**healthy**

**Diet**

**habits**

**promoting**

**physical**

**activit**

**smoking**

**cessation**

**& control**

**Post**

**intervention**

**Assessment**

**&**

**Riadh**

**Schools, workplaces,**

**health centres and**

**community**

**2013**

**2**

**2**

**009**

**010**

**3 ans d’intervention: 2010, 2011, 2012**

**2**

**014**

**Tunisian**

**Revolution**

**January 2011**

Control

Group

**Post**

**Post**

**intervention**

**Assessment**

**M’saken**

**intervention**

**assessment**

**Ususal Intervention**

**Schools, workplaces,**

**health centres and**

**community**

**4**

**.1.2. Study population**

Our study population consisted of a sample from the different settings

included in the study. These samples have helped us evaluate the

different NCDS risk factors but the intervention actions concerned the

entire population of these setting areas.

The sample size calculation in the three settings was based on a type 1

error of α = 5% , a type 2 error of β = 20% and a change in the

prevalence of various factors risk (smoking, poor diet, lack of physical

activity) of 6% between the pre and post intervention. For this, we

needed 4,000 college schoolchildren in intervention and control area,

6

1


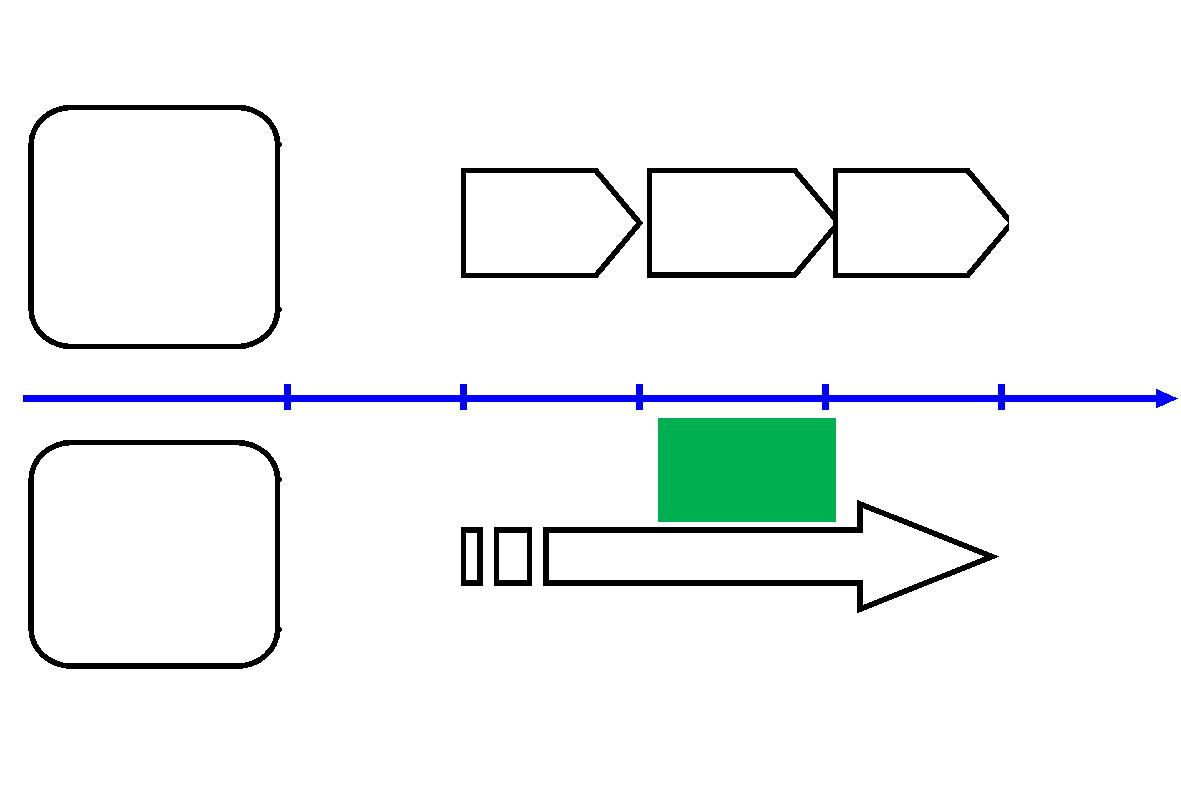


2

,000 adults in communities and 2,000 employees in professional

settings.

For school and community settings, the assessment before and after the

intervention concerned representative samples drawn, while for the

workplace setting assessment, we selected a convenience sample of

three enterprises in the intervention area and 3 enterprises in the

control area based on their numbers of employees and sex distribution.

Thus, in the schools, all colleges from delegations of Sousse Jawhara and

Sousse Erriadh (n = 8) formed the intervention group and all colleges

from the delegation Msaken (n = 7) formed the control group. A

stratified proportional sampling was used to select the schoolchildren

who participated in the data collection.

In the community, we have drawn 500 households in the intervention

area and 500 households in the control area, all adults aged 18 to 65

years found in these households were included in the data collection. In

the workplace, 3 enterprises were selected by convenience to be part of

the intervention group (Epi d'Or, TEXMED, UATS) and 3 enterprises

relatively similar in terms of size and gender composition in the control

group (STIP, AAF, FITLEC). All employees of the selected companies were

included in the data collection.

**4**

**.1.3. Data collection**

Three questionnaires designed for the "Community Intervention for

Health" project to which we previously participated were translated into

Arabic, pre-tested and used for data collection among participants in the

6

2

three settings: schools, work places and communites. They were

administred before and after the intervention. In schools, the

questionnaire was self-administered and distributed to schoolchildren in

their classes, in the presence of pre-trained doctors to ensure the

standardization of administration. The administration of the

questionnaires was conducted by interview in the workplaces and

neighborhoods.

The questionnaires have enabled us to collect the following information

in three areas:

•

•

•

•

Socio demographic characteristics

Knowledge, attitudes and smoking behavior

Knowledge, attitudes and eating behaviors

Knowledge, attitudes and physical activity behaviors

We also collected biometric data such as height and weight. The weight

was measured to the nearest 0.1 kg using a portable electronic scale.

The height, in standing position was measured in participants with bare

feet to the nearest 0.5 cm. Blood pressure was measured only for adult

participants in the household and workplace twice at rest using an arm

electronic sphygmomanometer.

**4**

**.1.4. Variables definition**

Definition of overweight and obesity: Body Mass Index (BMI) in kg / m^2^

was calculated by the ratio of weight to the square of the height. To

define overweight and obesity among schoolchildren, we used the Cole

BMI threshold values by age and sex (61).

6

3

Definition of hypertension: an average of the two measurements

greater than or equal to 140 mmHg for systolic blood pressure and / or

9

0 mmHg for diastolic blood pressure (62).

Definition of smoking: In adults, participants were asked the question:

"Do you currently smoke tobacco products such as cigarettes, cigars, or

chicha?". Smokers were participants who answered yes to this question.

Among adolescents were considered smokers, schoolchildren who

smoked at least one cigarette a month prior to the study (63).

The recommended level of physical activity was used as defined by

WHO for children and adults (64).

For healthy diet, the notion of eating at least 5 servings of fruits and

vegetables per day was used to assess participants' knowledge and their

eating habits.

**4**

**.1.5. statistical analysis**

Data were entered and analyzed using SPSS 17.0 software. The following

analysis procedures were carried out where their applications were

appropriate: The Student t test and the Chi-square test were used on

independent samples, respectively for comparison of means and inter-

group percentages. A statistical significance was set at 5% for the

different tests used.

6

4

**4**

**.1.6. Ethical considerations**

The study protocol, data collection forms, the questionnaire and the

manual methods of investigation were approved by the ethics

committee of the University Hospital Farhat Hached of Sousse.

We asked permissions to the Ministry of Health, the Governor of Sousse,

the Regional direction of education, school management and the group

of Occupational Medicine of Sousse. For schoolchildren, we got the

passive consent of their parents who were informed of the ongoing

project and its objectives and could refuse the participation of their

children and report it to the research team.

Adults in the workplaces and households participants signed a written

consent form prior to participation in data collection.

**4**

**.2. Results of the project « Together in Health »**

**.2.1. Results in school settings**

**.2.1.1. Sociodemographic characteristics:**

**4**

**4**

Before the beginning of the intervention program, 4003 schoolchildren

were included in the study: 1929 in the intervention group and 2074 in

the control group with a response rate of 94.6%. After the intervention,

4

2

275 schoolchildren participated in the evaluation with respectively

170 and 2105 in each group. The response rate at the end of the study

was 92.9% (Table VI).

6

5

Table VI: Schoolchildrens’ distribution and responses rates in both

intervention and control groups before and after the intervention

program.

Intervention group

n (response rate %)

1929 (93.1)

Control group

n (response rate %)

2074 (96.0)

Total

n (response rate %)

4003 (94.6)

Pre-assessement

Post-assessement

Colleges n=15

Colleges n=15

2170 (91.9)

2105 (93.9)

4275 (92.9)

Regarding the gender distribution in the two groups, there was not a

significant difference between participants before and after the

intervention in both intervention (p = 0.35) and control (p = 0.45)

groups. (Figure 3)

100%

80%

60%

40%

20%

4

5

9.8%

0.2%

51.3%

48.7%

52.3%

47.7%

5

4

3.5%

6.5%

Girls

Boys

0%

Pre-assessment Post assessment Pre-assessment Post assessment

Intervention Group Control Group

Figure 3: Schoolchildrens’ distribution by gender in both intervention

and control groups before and after the intervention.

6

6


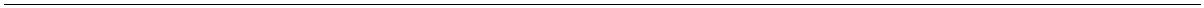

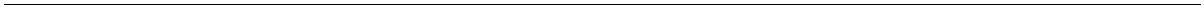

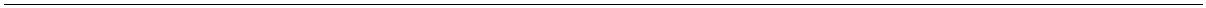

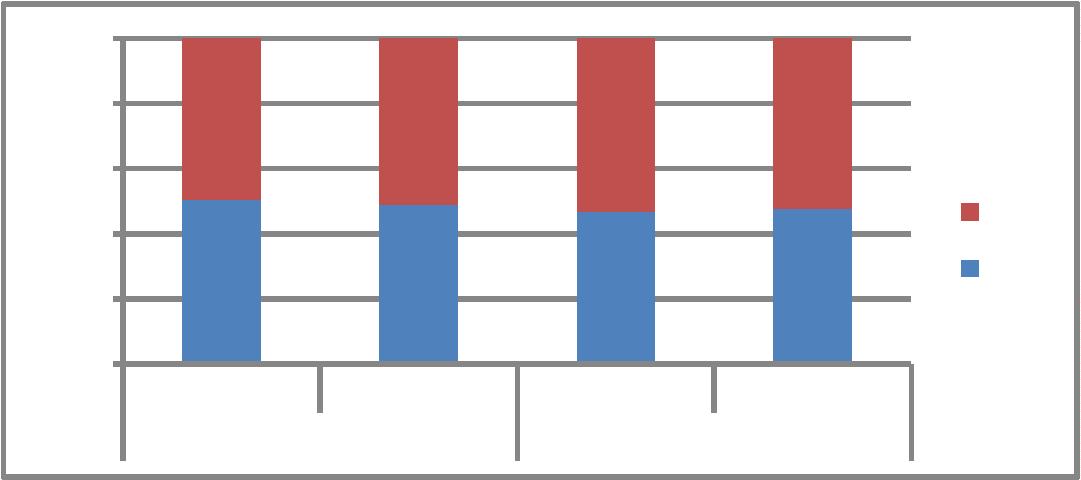


In terms of age; in the intervention group; there was no significant

difference between participants before and after the intervention

(p=0.52). However in control group, the composition has changed

significantly including more schoolchildren aged less than 14 years

(p<0.001) (Figure 4).

100

90

80

70

60

50

40

30

20

10

0

~~4~~

~~5~~

~~4.3%~~

~~5.7%~~

45.3%

54.7%

4

5

7.3%

2.7%

5

3.2%

6.8%

≥

14 years

<14 years

4

Pre-assessment Post assessment Pre-assessment Post assessment

Intervention Group Control Group

Figure 4: Schoolchildrens’ distribution according to the age in both

intervention and control groups before and after the intervention.

**4**

**.2.1.2. Knowldge and perceptions evolution:**

**.2.1.2.1. Evolution of tobacco use perceptions:**

**4**

In the intervention group, perceptions about smoking have been

improved but not significantly. While in the control group, there was a

6

7


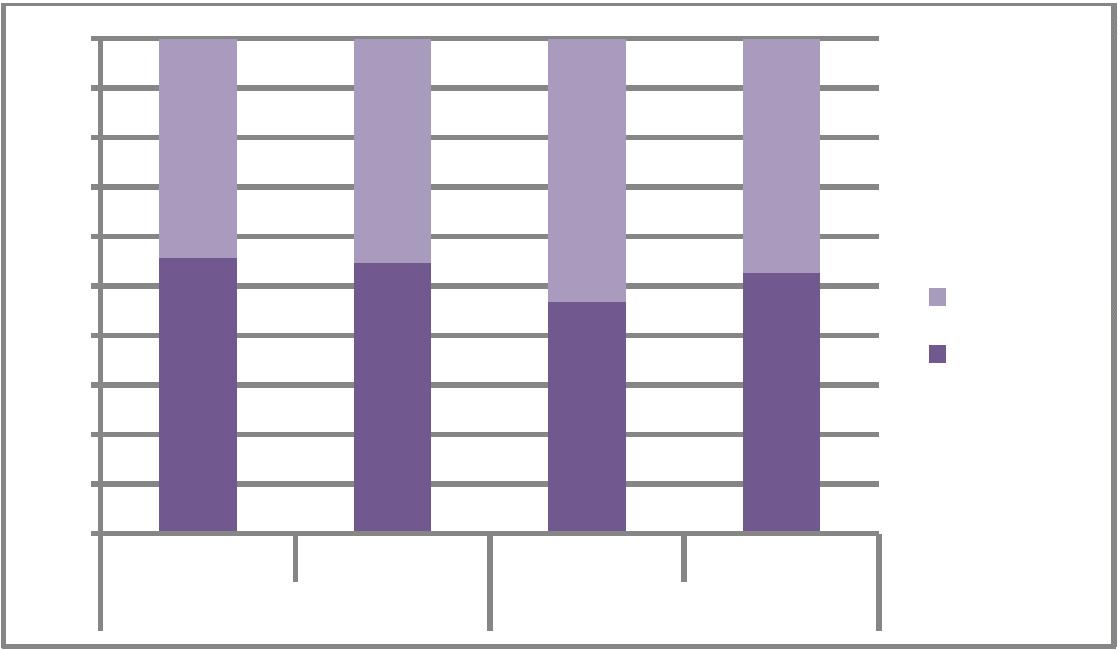


significant decline in evaluated perceptions. In fact, the proportion of

schoolchildren who agreed with a legislation banning smoking in public

places decreased from 98.5% to 93% (p <0.001) and the proportion of

those who thought that smoking did not make the boys more attractive

decreased significantly (Table VII).

Table VII: Evolution of schoolchildrens’ tobacco use perceptions before

and after the intervention in both intervention and control groups.

Intervention group

Pre assessement Post assessement

n(%)

Control group

Pre assessement Post assessement

p

p

n(%)

n(%)

n(%)

It is important to

ban tobacco use

in public places

Tobacco use does

not make boys

more attractive

Tobacco use does

not make girls

1

824

2023

2018

1886

0.60

<0.001

(94.9)

(94.6)

(98.5)

(93.0)

1

644

1790

1854

1684

0.26

0.98

<0.001

0.5

(85.8)

(87.0)

(90.2)

(86.3)

1

753

1887

1878

1822

(91.5)

(91.6)

(91.8)

(92.3)

more attractive

**4**

**.2.1.2.2.**

**Evolution of knowledge about physical**

**activity:**

We observed a non significant increase on knowledge about the

recommended level of physical activity (60 minutes per day) in the

intervention group from 45.3% to 46.4% (p= 0.48). However, in the

6

8


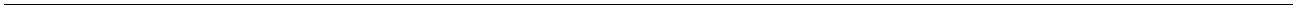

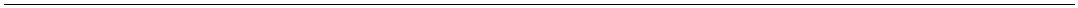

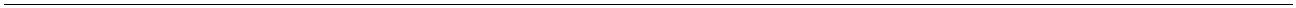

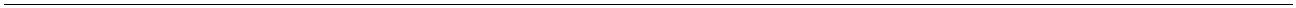

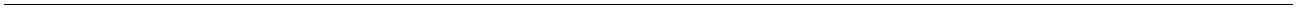

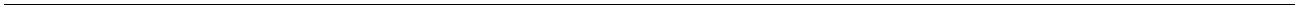


control group we observed a significant decrease from 49.6% to 44.3%

(p <0.001).

100

90

80

70

60

50

40

30

20

10

0

49.6%

4

6.4%

44.3%

4

5.3%

Pre-assessment

Post assessment

Pre-assessment

Post assessment

Intervention Group

Control Group

Figure 5: Evolution of schoolchildrens’ knowledge about the

recommended level of physical activity before and after the intervention

in both intervention and control groups.

**4**

**.2.1.2.3.**

**Evolution of knowledge on diet:**

Knowledge about the unhealthy eating habits has improved in the two

groups (Table VIII). Moreover, knowledge about health dietary benefits

has improved significantly in the intervention group. This improvement

was especially observed with the impact of sugar and fat consumption in

the intervention group contrary to the control group (Table IX).

6

9


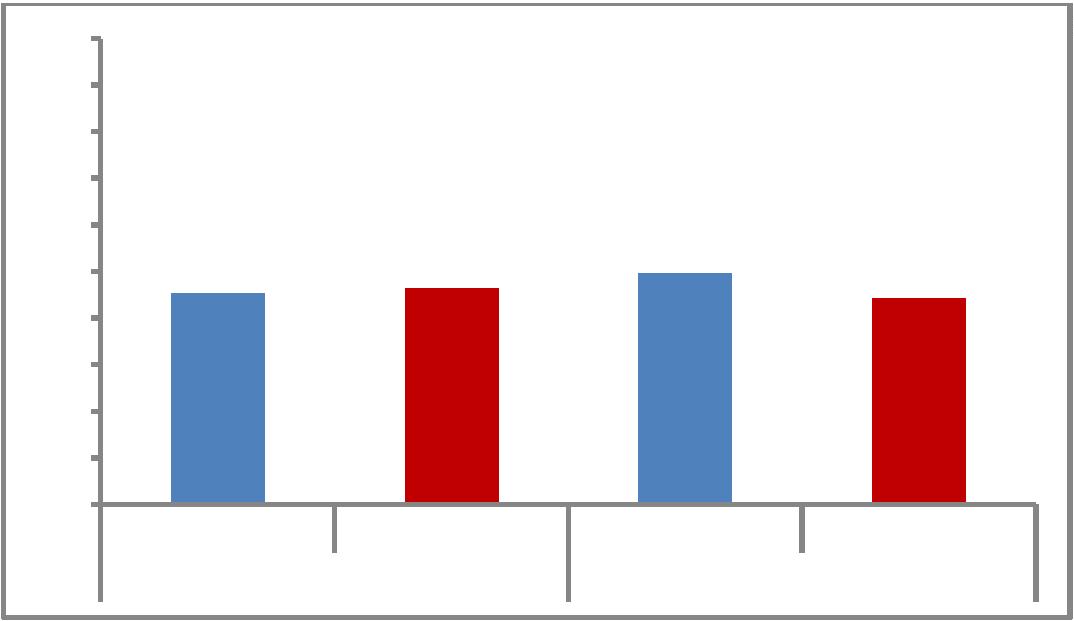


Table VIII: Evolution of schoolchildrens’ knowledge on diet before and

after the intervention in the two groups.

Intervention group

Pre assessement Post assessement

Control group

Post assessement

n(%)

Pre assessement

n(%)

p

p

n(%)

n(%)

It is recommended to

consume 5 portions

of fruits and

454

529

738

478

0

.453

<0.001

<0.001

(23.6)

(24.6)

(35.6)

(23.6)

vegetables daily

Unhealthy eating

habits can be harmful

344

686

457

675

<

0.001

(17.9)

(31.8)

(22.1)

(33.1)

Table IX: Evolution of schoolchildren’s knowledge about the benefits of

healthy diet before and after the intervention in the two groups.

Intervention group

Control group

Eating habits which can

have a significant

influence on health:

Increasing fruits and

vegetables consumption

Reducing sugar

consumption

Pre assessement

Post assessement

n(%)

Pre assessement

n(%)

Post assessement

n(%)

p

p

n(%)

1779

(86.2)

1313

1673

(82.6)

1567

1608

(83.8)

1597

1844

(85.8)

1467

0

.002

0.08

<0.001

0.001

0.001

<

<

0.001

0.001

(68.6)

1292

(74.9)

1532

(77.5)

1628

(72.8)

1389

Reducing fat

consumption

(67.5)

1206

(72.3)

1442

(79.0)

1386

(69.4)

1343

Reducing salt

0

.7

consumption

(63.1)

(68.0)

(67.4)

(66.9)

7

0


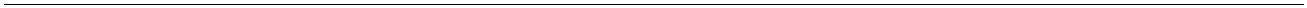

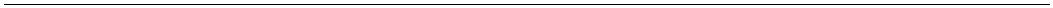

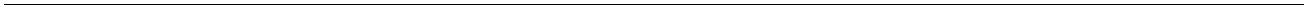

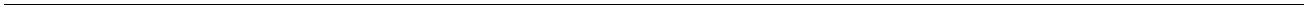

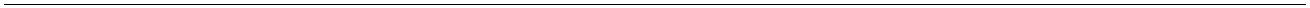

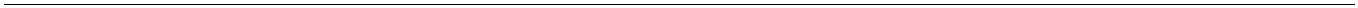

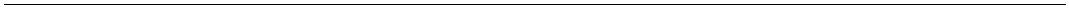

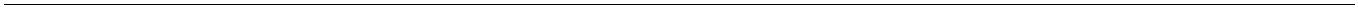

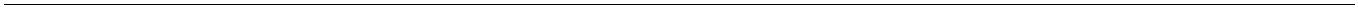

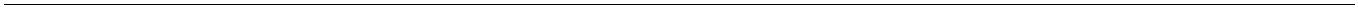

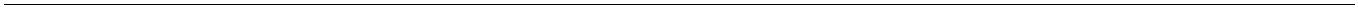

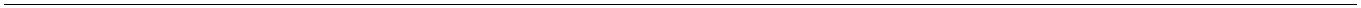


**4**

**.2.1.3. Evolution of lifestyle habits:**

**.2.1.3.1. Tobacco use evolution:**

**4**

There was a non significant decrease in tobacco use from 5.7% to 4.8%

(p= 0.2) in the intervention group. Whereas, in control group tobacco

use increased significantly from 7.5% to 9.2% (p= 0.048). (Figure 6)

1

0

9

8

7

6

5

4

3

2

1

0

9.2%

7

.5%

5

.7%

4

.8%

Pre-assessment

Post assessment

Pre-assessment

Post assessment

Intervention Group

Control Group

Figure 6 : Evolution of the tobacco use among schoolchildren before and

after the intervention in both intervention and control groups.

After adjustement for sex, in the intervention group, there was a non-

significant decline in tobacco use among both boys and girls. While, in

control group we observed a significant increase of tobacco use among

girls from 1.1% to 5.2% (p <0.001). We observed also a significant

increase in tobacco use from 3.3% to 5.9% among those under 14 years

of age in the same group (p = 0.006). (Table X)

7

1


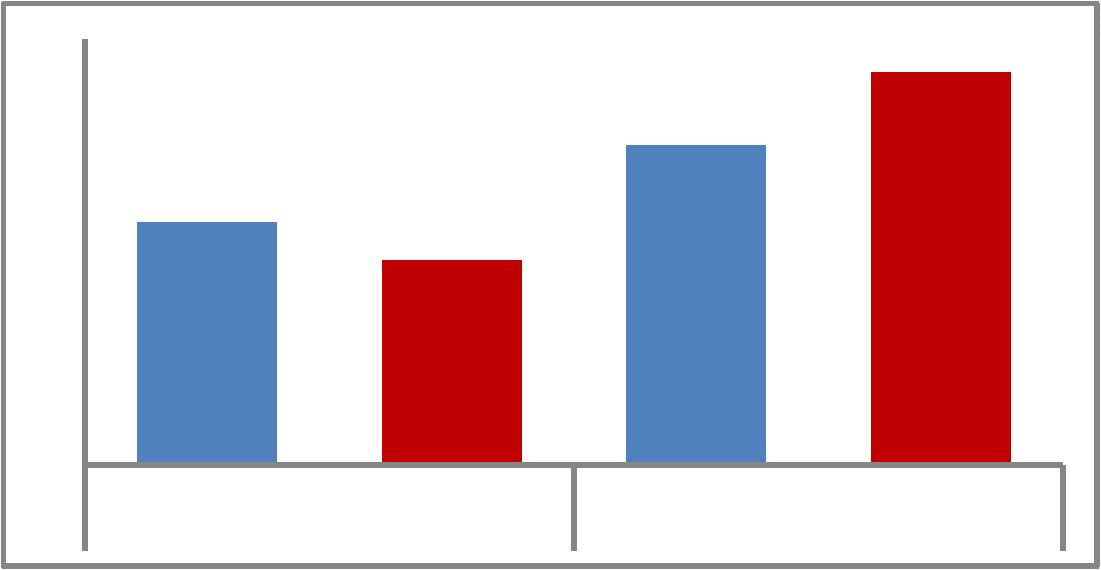


Table X : Evolution of tobacco use among schoolchildren according to

age and sex before and after the intervention in intervention and

control groups

Intervention group

Control group

Pre assessement

n (%)

Post assessement

n (%)

Pre assessement

n (%)

Post assessement

n (%)

p

p

Boys

Girls

85 (8.8)

25 (2.6)

81 (7.8)

23 (2.1)

0.4

0.4

143 (14.8)

12 (1.1)

136 (13.6)

57 (5.2)

0.4

<0.001

<

14

years

14

years

3

7 (3.4)

41 (3.5)

63 (6.5)

0.9

32 (3.3)

65 (5.9)

0.006

0.2

≥

7

3 (8.5)

0.09

123 (11.1)

127 (12.8)

**4**

**.2.1.3.2.**

**Practice of recommanded level of physical**

**activity evaluation:**

The practice of the recommended level of physical activity (60 minutes

per day) decreased significantly from 29.1% to 25.5% (p = 0.010) in the

intervention group and remain unchanged in control group (p = 0.887)

(Figure 7).

The decrease of the practice of the recommended level of physical

activity was significant for boys and for schoolchildren aged 14 or more

in the intervention group (Table XI).

7

2


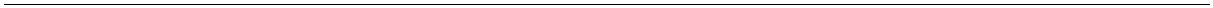

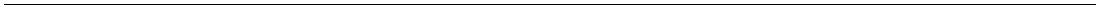

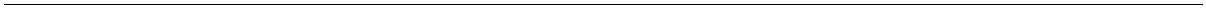

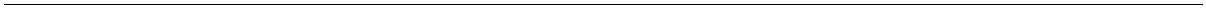

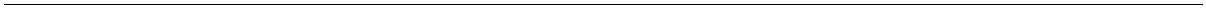

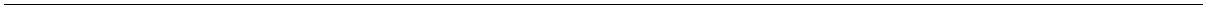

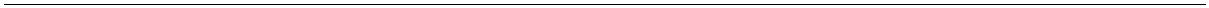


35

30

25

20

15

10

5

0

2

9.1%

2

5.5%

2

1.1%

21.2%

Pre-assessment

Post assessment

Pre-assessment

Post assessment

Intervention Group

Control Group

Figure 7: Evolution of practice of the recommended level of physical

activity before and after the intervention in the two groups.

Table XI: Evolution of the schoolchildrens practice of the recommanded

level of physical activity before and after the intervention in the two

groups.

Intervention group

Control group

Post assessement

Pre assessement

Post assessement

n(%)

Pre assessement

n(%)

p

p

n(%)

n(%)

Boys

Girls

431 (44.9)

123 (13.0)

406 (39.8)

126 (11.9)

0.02

0.40

355 (37.1)

79 (7.2)

343 (35.8)

82 (7.9)

0.6

0.5

<

14

years

14

years

3

2

20 (30.1)

34 (27.8)

326 (28.5)

206 (21.8)

0.400

0.003

203 (21.1)

231 (21.0)

229 (21.5)

195 (20.9)

0.80

0.96

≥

7

3


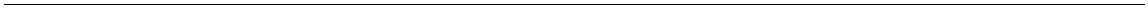

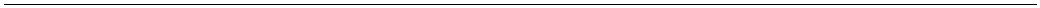

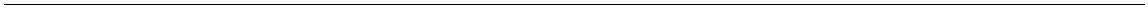

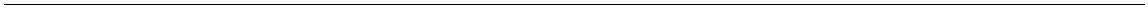

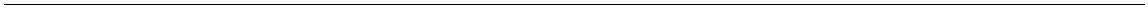

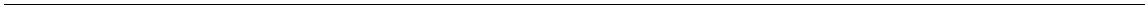

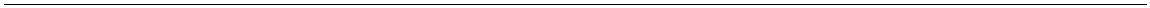

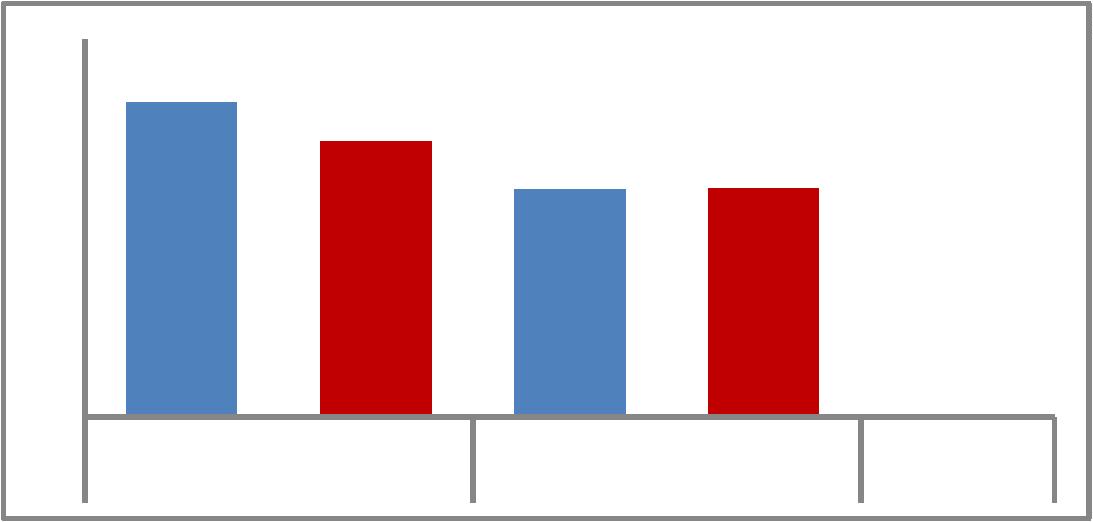


**4**

**.2.1.3.3.**

**Eating habits evolution:**

Concerning the consumption of five fruits and vegetables per day, there

was a significant improvement in the intervention group from 30% to

3

3

3.2% (p = 0.027) in opposition to a significant decrease from 40.2% to

5% (p = 0.001) in control group. (Figure 8)

50

40

30

20

10

0

4

0.2%

3

5%

3

3.2%

3

0%

Pre-assessment

Post assessment

Pre-assessment

Post assessment

Intervention Group

Control Group

Figure 8: Evolution of 5 fruits and vegetables consumption daily before

and after the intervention in the two groups.

When we adjusted by sex, improvement of the consumption of five

fruits and vegetables per day in the intervention group becomes non

significant contrary to a significant decrease in the control group for

both sexes (Table XI). Furthermore we observed a significant

improvement from 30.2% to 35.4% (p = 0.009) among schoolchildren

aged under 14 years in the intervention group, whereas there was a

significant decrease in those 14 years old or more in the control group.

(Table XII)

7

4


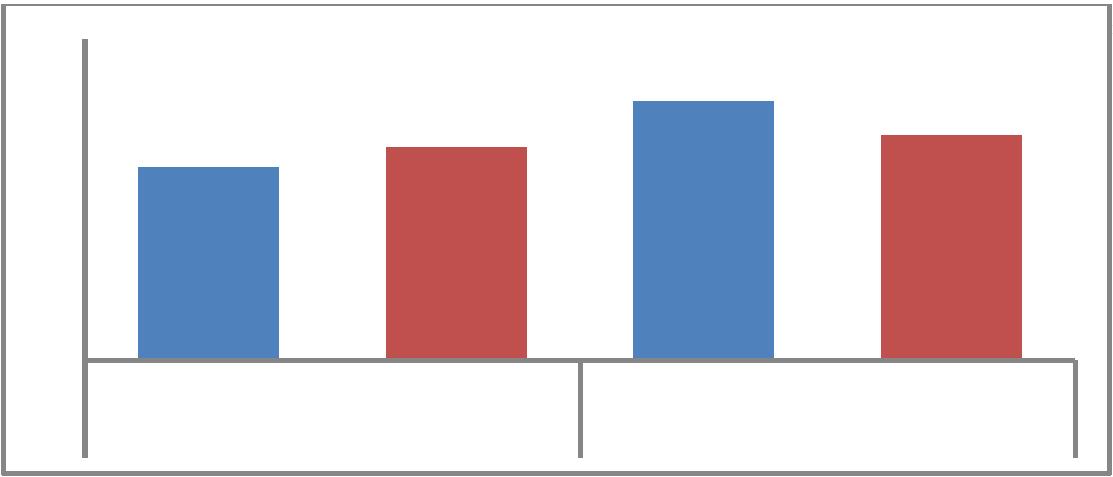


Table XII : Evolution of the consumption of 5 portions of fruits and

vegetables daily before and after the intervention in the two groups.

Intervention group

Control group

Pre assessement

Post assessement

n(%)

Pre assessement

n(%)

Post assessement

n(%)

p

p

n(%)

Boys

Girls

314 (33.3)

251 (26.6)

350 (37.1)

320 (30.0)

0.070

0.100

419 (44.0)

402 (36.9)

353 (37.2)

340 (32.9)

0.002

0.050

<

14

years

14

years

3

2

19 (30.2)

46 (29.7)

408 (35.4)

290 (30.6)

0.009

0.700

379 (39.5)

442 (40.9)

401 (37.9)

291 (31.6)

0.460

≥

<0.001

Focusing on family eating habits, we observed in the intervention group

a significant increase in parental provision of fruits and vegetables. We

observed also an increase in parental encouragement to consume more

fruits and vegetables in addition to an increase of healthy cooking

methods by parents.

On the other side, in the control group, the parental provision of fruits

and vegetables to children decreased significantly from 75.2% to 68.4%

(p <0.001) (Table XIII) despite their increased encouragement of fruits

and vegetables consumption.

7

5


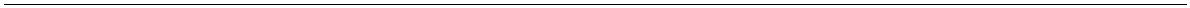

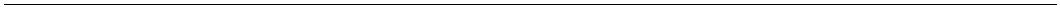

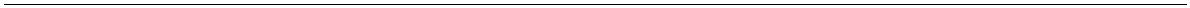

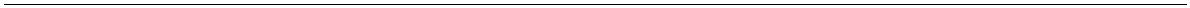

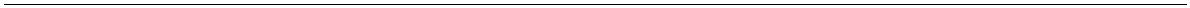

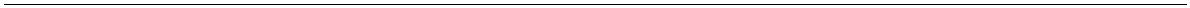

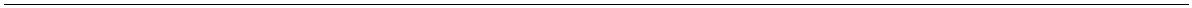


Table XIII: Family eating habits repartition among schoolchildren before

and after the intervention in the two groups.

Intervention group

Control group

Post assessement

At least 5 days a week ,

one of your parents :

Pre assessement

n(%)

Post assessement

n(%)

Pre assessement

n(%)

p

p

n(%)

Encourage you to

consume more fruits

and vegetables

1232 (64.0)

1101 (57.6)

541 (28.6)

1505 (69.8)

1447 (67.9)

<0.001

1014 (48.9)

1559 (75.2)

1272 (62.6)

<0.001

Provide you fruits and

vegetables in meals or

snacks

<0.001

1382 (68.4)

<0.001

Prepare for you grilled

or steamed or boiled

food

644 (30.5)

253 (11.9)

<0.001

0.100

406 (19.7)

213 (10.3)

521 (26.0)

234 (11.6)

0.200

0.200

Prepare for you fried

meals

2

56 (13.4)

**4**

**.2.1.3.4.**

**Evolution of excess weight:**

The mean of Body Mass Index (BMI) among schoolchildren in the

intervention group decreased significantly from 20.8 ± 4 kg / m^2^ to 20.3

±

4.3 kg / m^2^ (p <0.001). Whereas in control group, it increased

2

2

significantly from 20.0 ± 4.1 kg / m to 20.4 ± 4.2 kg / m (p = 0.004).

Otherwise, the excess of weight among schoolchildren decreased in the

intervention group from 27.6% to 26% against an increase in the control

group from 20.1% to 25.6%. (Figure 9)

7

6


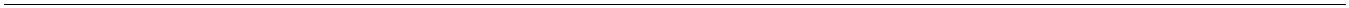

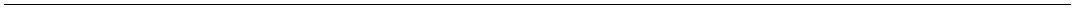

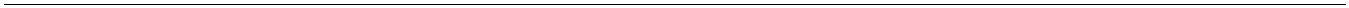

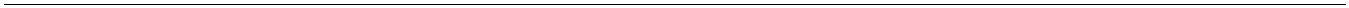

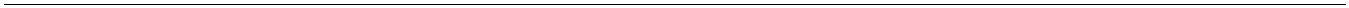

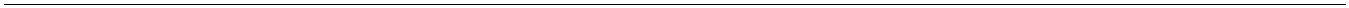

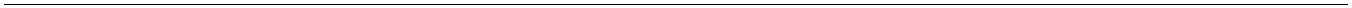


30%

25%

20%

15%

10%

27.6%

2

6.0%

2

5.6%

2

0.1%

5

%

%

0

Pre-assessment

Post assessment

Pre-assessment

Post assessment

Intervention Group (p=0.24)

Control Group (p<0.001)

Figure 9: Evolution of excess of weight before and after the intervention

in the two groups.

In the intervention group, proportion of boys with normal weight

increased significantly from 73.1% to 77.2% (p = 0.03) and proportion of

boys with overweight decreased significantly from 20.4% to 16.9% (p =

0

.04 ). When it comes to obesity, it did not decrease significantly in this

group.

While in the control group, the proportion of obese boys increased

significantly from 4.9% to 7.6% (p = 0.01). (Table XIV)

For girls, there was no significant change in terms of overweight in the

intervention group. However, in the control group the proportion of

girls in normal weight decreased significantly from 79.9% to 76.2% (p =

0

.03) and the proportion of obese girls increased significantly from 4.2%

to 6.3% (p= 0.03). (Table XIV)

7

7


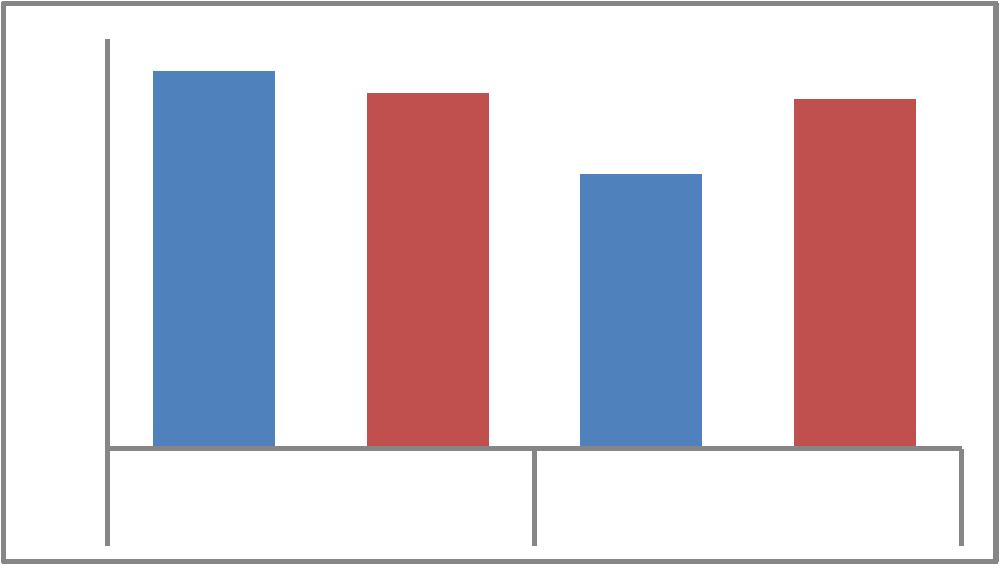


Table XIV : Weight status by sex among the schoolchildren before and

after the intervention in the two groups.

Boys

Girls

Pre assessement

n(%)

Post assessement

n(%)

Pre assessement

n(%)

Post assessement

n(%)

p

p

Normal weight

Overweight

707(73.1)

798 (77.2)

0.03

689 (71.8)

807 (74.0)

0.2

Intervention

group

197(20.4)

175(16.9)

0.04

199 (20.7)

207 (19.0)

0.3

Obese

63 (6.5)

61 (5.9)

0.57

0.20

72 (7.5)

77 (7.1)

0.7

Normal weight

772(80.1)

756 (77.8)

886 (79.9)

810 (76.2)

0.03

Control

group

Overweight

Obese

145 (15)

47 (4.9)

142 (14.6)

74 (7.6)

0.80

0.01

176 (15.9)

47 (4.2)

186 (17.5)

67 (6.3)

0.30

0.03

In the intervention group, the proportion of schoolchildren aged 14

years and older with overweight decreased significantly from 20.2% to

1

6.6% (p = 0.04). In the control group, the proportion of obesity

increased significantly for both age groups. (Table XV)

7

8


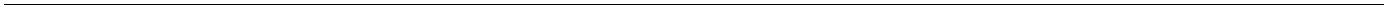

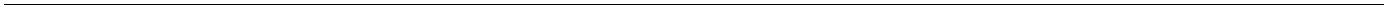

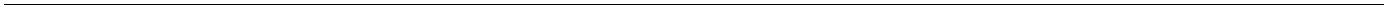

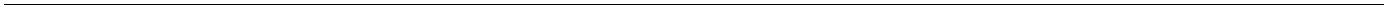

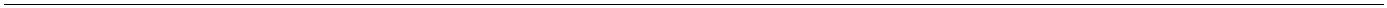


Table XV: Weight status by age among schoolchildren before and after

the intervention in the two groups.

<

14 years

≥14 years

Post assessement

Pre assessement

n(%)

Post assessement

n(%)

Pre assessement

n(%)

p

p

n(%)

Normal weight

Overweight

Obese

755 (70.3)

860 (73.8)

0.07

641(75.1)

745 (77.7)

0.20

Intervention

group

224 (20.9)

95 (8.8)

223 (19.1)

83 (7.1)

0.30

0.10

0.30

0.60

0.01

172 (20.2)

40 (4.7)

159 (16.6)

55 (5.7)

0.04

0.30

0.03

0.20

0.03

Normal weight

Overweight

Obese

756 (77.9)

164 (16.9)

50 (5.2)

826 (76.1)

175 (16.1)

85 (7.8)

902 (81.8)

157 (14.2)

44 (4.0)

740 (78.0)

153 (16.1)

56 (5.9)

Control

group

**4**

**.2.2. Results in workplace settings**

**.2.2.1. Description of the studied population:**

**4**

At the pre-assessment, we enrolled 914 employees of Sousse in the

intervention group and 861 in the control group. The response rate was

7

1

4.6%. At the post-assessment, the studied population was composed of

098 and 1015 employees in the intervention and control groups

respectively. The response rate was 71.9%.

7

9


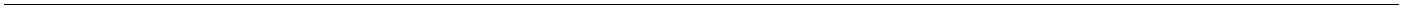

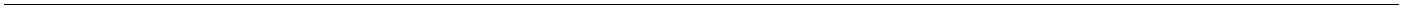

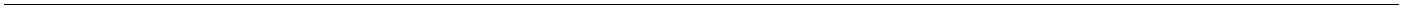

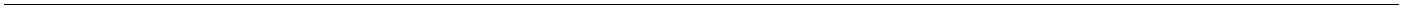

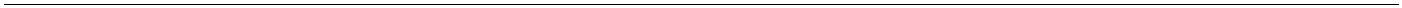


Table XVI: Evolution of the response rate of the two groups in

workplace

Intervention group

n (response rate %)

Control group

Total

n (response rate %)

n (response rate %)

Entreprises

n=6

Pre-assessment

Post-assessment

914 (76.7)

861 (72.5)

1775 (74.6)

2113 (71.9)

Entreprises

n=6

1098 (67.5)

1015 (77.5)

At the pre-assessment, the intervention group was composed of 64.7%

of men versus 65.5% at the post assessment. The control group was

composed of 59% of men at the pre-assessment versus 61.4% at the

post assessment. The differences were not significant in the two groups

(table XVII).

Table XVII: Distribution of the participants in the two groups at pre and

post assessment according to the sex

Intervention group

Post n (%)

Control group

Post n (%)

Sex

Men

Pre n (%)

p

Pre n (%)

508 (59.0)

353 (41.0)

861(100.0)

p

591 (64.7)

323 (35.3)

914 (100.0)

719 (65.5)

379 (34.5)

623 (61.4)

392 (38.6)

Women

Total

0.70

0.29

1098 (100.0)

1015 (100.0)

At the pre-assessment, the mean age of the intervention group

employees was 32.25 ± 8.11 years. It was 33.86 ± 8.10 years at the post

assessment (p<0.001).

8

0


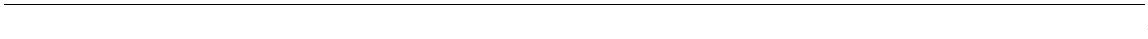

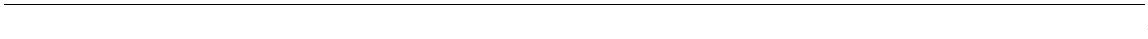

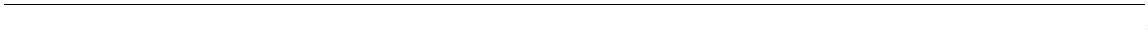

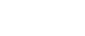

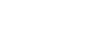

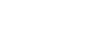

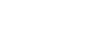

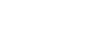

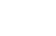


At the pre-assessment, the mean age of the control group employees

was 35.40 ± 8.79 years. It was 38.90 ± 8.77 years at the post assessment

(p<0.001).

The studied population was composed mostly of workers or technician

without significant difference at the pre-assessment and the post

assessment neither in the control group (p= 0.07) nor in the intervention

group (p= 0.26).

**9**

**1.8%**

**8**

**9.3%**

**8**

**5.6%**

**8**

**3.7%**

worker and technician

other

1

6.3%

14.4%

1

0.7%

8

.2%

pre

post

pre

post

Control group

Intervention group

Figure 10: Distribution of the participants in the intervention

program according to the profession in intervention and control

group

8

1

**4**

**.2.2.2. Evolution of the knowledge and perceptions on**

**non-communicable diseases risk factors before and**

**after intervention among the employees in**

**workplace of Sousse:**

**For eating habits:**

In the intervention group, the workers improved their knowledge on the

relation between the food they consumed and the risk of developing

non-communicable diseases. The observed improvement was significant

(p<0.001) for the relation between dietary habits and the risk to develop

cardio-vascular diseases, diabetes mellitus and cancers.

Likewise for the control group, we observed significant improvements of

the knowledge (Table XVIII).

Table XVIII: Evolution of the knowledge on the relation between eating

habits and the risk to develop non-communicable diseases of the

employees in the two groups

Intervention group

Control group

Post n (%)

Do you think that what you

are consuming can change

the risk?

Pre n (%)

Post n (%)

p

Pre n (%)

p

Cardio-vascular diseases

506 (56.2)

797 (72.7)

<0.001

464 (55.2)

715 (71.4)

<0.001

Diabetes mellitus

Cancers

593 (65.4)

337 (37.5)

862 (78.7)

675 (61.8)

<0.001

<0.001

542 (65.0)

313 (37.9)

787 (78.3)

626 (63.0)

<0.001

<0.001

8

2

The table XIX shows significant improvement of the perceptions among

the employees in the intervention group about the importance of some

eating habits for health (changing the methods of cooking, eating more

fiber and more fruits and vegetables, eating less sugar, salt and fat).

Table XIX: Evolution of the participants’ perceptions on the influence of

healthy diet on health before and after the intervention in both groups

It’s important for

Intervention group

Post n (%)

Control group

Post n (%)

health

Pre n (%)

p

Pre n (%)

p

Changing cooking

methods

7

40 (82.2)

961 (87.6)

988 (90.1)

0.001

728 (88.0)

855 (85.0)

871 (87.0)

0.06

Eating more fibre

777 (86.2)

834 (92.6)

0.008

745 (88.6)

0.30

Eating more fruits

and vegetables

1047 (95.6)

1034 (94.4)

0.004

779 (93.1)

745 (89.2)

900 (89.7)

896 (89.4)

0.01

0.89

Eating less sugar

Eating less fat

8

8

00 (88.9)

02 (89.3)

<0.001

1036 (94.4)

1021 (93.2)

<0.001

<0.001

758 (90.7)

745 (89.4)

899 (89.4)

890 (88.7)

0.35

0.63

Eating less salt

794 (88.2)

8

3

**For physical activity:**

In the intervention group, the employees improved significantly

(p<0.001) their knowledge on the benefits of practicing 30 minutes of

physical activity per day at least 5 days a week. Likewise for the control

group, we observed significant improvements of the knowledge (tableau

XX).

Table XX: Evolution of the participant’s knowledge on the benefits of the

practicing 30 minutes of physical activity per day at least for 5 days a

week in both groups

Intervention group

Control group

Post n (%)

practicing thirty minutes of

physical activity per day at

least for 5 days a week

Pre n (%)

Post n (%)

p

Pre n (%)

p

Reduce risk of diseases

661 (73.3)

966 (88.4)

978 (89.5)

<0.001

<0.001

626 (75.0)

658 (78.8)

878 (87.3)

886 (88.2)

<0.001

<0.001

Improve heart and lung

fitness

6

83 (75.8)

Gain muscle

Help losing weight

Prevent depression

Reduce stress

592 (65.9)

514 (57.5)

546 (60.9)

562 (62.6)

571 (63.2)

916 (83.9)

834 (76.5)

873 (79.9)

894 (81.8)

865 (79.1)

<0.001

<0.001

<0.001

<0.001

<0.001

606 (72.9)

532 (63.6)

520 (61.4)

526 (62.9)

554 (66.3)

822 (81.8)

779 (77.7)

782 (78.0)

799 (79.7)

800 (79.8)

<0.001

<0.001

< 0.001

<0.001

<0.001

Do better on job

8

4

**For tobacco use:**

The proportion of employees who recognized that cigarettes labeled

light aren’t less harmful than regular cigarettes increased significantly in

the intervention group (p<0.001). However, it decreased in control

group (p=0.13).

In control group, the improvement of knowledge that tobacco use is

addictive was 9.5% vesus 17% in intervention group.

The increase of the proportion of participants in control group who

think smoking of narguile as harmful for health was 8.8% versus 12.1%

in intervention group. These improvements were statistically significant

(p<0.001) in the two groups (figure 11).

9

3.6%

8

6.5%

87.2%

8.4% 81.1%

8

1.5%

7

7

1.5%

6

9.5%

The light cigarette smoking is not less

harmful

4

9.1%

4

4.1%

4

0.6%

Tobacco use can result in the addiction

The smoking of narguile is harmful

3

3.8%

pre

post

pre

post

Intervention group

Control group

Figure 11: Evolution of the knowledge about tobacco use among

employees in the two groups

8

5

**4**

**.2.2.3. Evolution of employees behaviors in workplace:**

**For eating habits:**

In the intervention group, the employees decreased 2.2% the habit of

adding salt on food (p=0.05). The employees in the control group

decreased this habit in 1.5% (p=0.67).

We assessed the habit of eating snacks among the employees

participating to the study. The intervention group increased

insignificantly the habit of choosing fried food (p=0.42). It increased

significantly the habit of choosing fruits or vegetables as a snack

(p=0.01) and decreased significantly the consumption of milk and dairy

products (p=0.02). The evolution in the control group consisted in an

increase of the consumption of fried food, a decrease of the

consumption of fruits or vegetables and a decrease in the consumption

of milk and dairy products. All these trends observed among the control

group were statistically significant (table XXI).

8

6

Table XXI: Evolution of snacks consumed by the participants in the two

groups

Intervention group

Control group

Post n (%)

Pre n (%)

178 (19.8)

Post n (%)

232 (21.3)

p

Pre n (%)

p

Choose fried foods

0.42

128 (15.2)

203(20.5)

208 (21.1)

0.003

Choose fruits and

vegetables

1

2

40 (15.6)

14 (23.8)

217 (19.9)

211 (19.4)

0.01

0.02

258 (30.6)

235 (27.8)

<0.001

<0.001

Choose milk and

dairy products

187 (18.9)

The intervention group improved significantly the behavior of

consuming at least five servings of fruits and vegetables daily (p=0.04)

whereas in the control group, the observed improvement was not

significant (p=0.57) (table XXII).

Table XXII: Evolution of the consumption of 5 fruits and vegetables daily

among the employees participating to the study in the two groups

Eating at least 5 servings of

fruits and vegetables daily

Intervention group

Control group

Post n (%)

Pre n (%)

Post n (%)

558 (52.1)

513 (47.9)

p

Pre n (%)

504 (60.9)

323 (39.1)

p

yes

no

421 (47.5)

466 (52.5)

0.04

613 (62.2)

372 (37.8)

0.57

8

7

When we adjust for sex, the habit of eating at least five fruits and

vegetables daily was not significantly improved among men and women

in the intervention group. While for the control group, men reduced

significantly (p = 0.001) this habit and women improved it significantly (p

<0.001).

After adjustment for the age, this habit was improved among

participants who were aged 40 years old or more in the intervention

group (p = 0.05) (table XXIII).

Table XXIII: Evolution of the consumption of 5 fruits and vegetables per

day among the employees participating to the study in the two groups

according to the sex and the age

Eating at least 5

portions of fruits and

vegetables per day

Men

Intervention group

Control group

Post n (%)

Pre n (%)

Post n (%)

p

Pre n (%)

p

281 (48.6)

140 (45.3)

346 (46.5)

69 (51.9)

375 (53.7)

183 (49.1)

430 (49.7)

128 (62.4)

365 (73.9)

139 (41.7)

287 (54.4)

207 (72.9)

395 (64.9)

218 (58.0)

318 (55.9)

295 (70.9)

0

0

0

0

.07

.33

.21

.05

0.001

<0.001

0.61

Women

≤

40 years

>40 years

0.57

**For physical activity:**

The employees participating to the intervention program improved

significantly their physical activity level in both groups (Table XXIV).

8

8

Table XXIV: Evolution of the behavior of practicing 30 minutes of

physical activity per day during 5 day per week at least in the two

groups

Practicing 30 minutes of

Intervention group

Post n (%)

Control group

Post n (%)

physical activity daily

Pre n (%)

p

Pre n (%)

262 (31.2)

577 (68.8)

p

during 5 days per week

yes

no

253 (28.3)

642 (71.7)

414 (37.9)

679 (62.1)

426 (42.9)

568 (57.1)

<

0.001

<0 .001

This improvement was significant for men and women in both groups.

After stratification by age, only employees above 40 years old in the

intervention group improved significantly their physical activity level

(p<0.001) (Table XXV). In the control group, the improvement remained

significant after adjustment for age and sex.

Table XXV: Evolution of the behavior of practicing 30 minutes of physical

activity per day during 5 day per week in the two groups adjusted for

sex and age

Intervention group

Control group

Post n (%)

Pre n (%)

Post n (%)

315(44.0)

p

Pre n (%)

p

Men

196 (33.8)

57 (18.1)

228 (30.2)

22 (16.8)

168 (33.4)

284 (46.6)

<

0.001

<0.001

Women

99 (26.3)

305 (34.6)

109 (51.7)

94 (28.0)

160 (29.8)

97 (33.7)

142(36.9)

254 (43.6)

172 (41.7)

0

0

.01

.06

0.01

<0.001

0.03

≤

40 years

>40 years

<

0.001

8

9

**For tobacco use:**

In the intervention group, the prevalence of tobacco use was 39.2% at

the pre-assessment and 37.5% at the post assessment (p=0.43). In the

control group, it was 31.7% at the pre-assessment and 30.6% at the post

assessment (p= 0.62). These trends were not significant (Table XXVI).

Table XXVI: Evolution in tobacco use prevalence in both groups

Are you a current

smoker?

Intervention group

Control group

Post n (%)

Pre n (%)

Post n (%)

410 (37.5)

p

Pre n (%)

p

yes

no

350 (39.2)

542 (60.8)

250 (31.7)

308 (30.6)

699 (69.4)

0

.43

0.62

683 (62.5)

539 (68.3)

After adjustment for sex and age (Table XXVII), the prevalence of

tobacco-use among men decreased from 59.1% to 56.7% in intervention

group (p=0.35) and it decreased from 52.2% to 49.4% in control group (p

=

0.38).

The prevalence of tobacco use among women was 1.3% at the pre-

assessment in the intervention group and decreased to 0.5% at the post

assessment. This improvement was not significant (p=0.42). It was 0.3%

in the control group without any improvement.

Age didn’t seem to have any impact on the evolution of tobacco-use

after the intervention.

9

0

Table XXVII: Evolution in tobacco use prevalence in both groups

adjusted for sex and age

Intervention group

Control group

Post n (%)

Pre n (%)

Post n (%)

408 (56.7)

p

Pre n (%)

p

Men

346 (59.1)

0.38

249 (52.2)

307 (49.4)

0.35

Women

4 (1.3)

2 (0.5)

0.42

0.99

0.21

1 (0.3)

1 (0.3)

-

≤

40 years

289 (38.7)

53 (39.3)

341 (38.7)

69 (32.7)

136 (27.0)

112 (41.0)

158 (26.9)

150 (35.7)

0.96

0.16

>

40 years

**4**

**.2.2.4. Evolution of other risk factors after the**

**intervention:**

The prevalence of obesity increased significantly both in the

intervention (p= 0.01) and the control groups (p <0.001). However, the

prevalence of overweight has not increased significantly in both groups

(Table XXVIII).

Table XXVIII: Evolution of the obesity and overweight in the two

groups

Intervention group

Control group

Post n (%)

Pre n (%)

Post n (%)

386 (35.3)

p

Pre n (%)

p

Overweight

Obesity

281 (31.4)

0.07

308 (37.5)

407 (40.6)

0.18

134 (15.0)

225 (20.6)

0.01

161 (19.6)

274 (27.3)

<0.001

9

1

The evolution of overweight adjusted for age and sex was not significant

in the intervention group. Whereas, we noticed a significant increase of

the overweight prevalence among women in the control group (Table

XXIX).

In the intervention group, obesity increased significantly among men as

well as women and only in participants aged less than 40 years (Table

XXIX). In the control group, obesity increased significantly among

women and in the two groups of age (Table XXIX).

Table XXIX: Evolution of the obesity and overweight in the two

groups adjusted for sex and age

Intervention group

Control group

Post n (%)

Pre n (%)

Post n (%)

264 (36.8)

p

Pre n (%)

p

Men

1

90 (32.5)

0.11

207 (43.1) 264 (42.7)

101 (29.6) 143 (37.1)

0.89

Women

9

1 (29.4)

122 (32.4)

301 (34.0)

85 (40.5)

0.39

0.07

0.79

0.02

0.02

0.001

0.68

0.03

0.23

Over- weight

≤

40 years

40 years

Men

2

25 (30.0)

174 (32.7)

211 (3.1)

>

5

2 (39.1)

1 (12.2)

3 (20.3)

3 (12.4)

9 (29.3)

133 (47.8) 196 (46.9)

107 (22.3) 162 (26.2)

0.80

7

120 (16.7)

105 (27.9)

159 (18.0)

66 (31.4)

0.13

Women

6

9

3

54 (15.8)

93 (17.5)

67 (24.1)

112 (29.1)

137 (23.4)

137 (32.8)

<0.001

0.01

Obesity

≤

40 years

>40 years

0.01

In the intervention group, the prevalence of hypertension decreased

significantly from 16.2% to 12.8% (p=0.03). However after adjustement

for sex and age, this improvement was only significant for participants

aged 40 years or less (p=0.04) (Table XXX).

9

2

In the control group, the proportion of hypertension increased

significantly from 13.3% to 23.3% (p<0.001). After adjustement, this

increase was significant according to sex and age (Table XXX).

Tableau XXX: Evolution of the hypertension in the two groups

adjusted for sex and age

Intervention group

Control group

Post n (%)

Hyper-tension

Pre n (%)

05 (17.9)

Post n (%)

103 (14.3)

p

Post n (%)

86 (17.1)

p

Men

1

0.08

184 (29.6)

<0.001

Women

4

1 (13.1)

8 (13.0)

6 (34.3)

37 (9.8)

85 (9.6)

55 (26.1)

0.17

0.04

0.10

26 (7.7)

56 (10.4)

56 (19.2)

52 (13.3)

98 (16.5)

138 (32.8)

0.01

0.003

<0.001

≤

40 years

9

>40 years

4

**4**

**.2.3. Results in community settings**

**.2.3.1. Description of the studied population:**

**4**

At the pre-assessment, our studied population was composed of 940

adults in the intervention group and 940 in the control group. At the

post assessment, the population was composed of respectively 1001

and 976 participants. The response rates were almost similar at the pre

and post assessment for the intervention group but we noticed a small

response rate decline in the control group at the post assessment.

9

3

Table XXXI: Evolution of the response rate of the two groups

Intervention group

n (response rate %)

control Group

Total

n (response rate %)

n (response rate %)

Pre-assessment

Post-assessment

940 (73.5)

940 (73.1)

976(62.5)

1880 (73.3)

1977 (67.9)

1001 (74.3)

The intervention group was composed of 56.8 % of women at the pre

assessment versus 55.8% at post assessment (p = 0.67). The control

group was composed of 71.2% of women at the pre assessment versus

6

5.7% at the end of the study (p = 0.01).

The table XXXII below shows the distribution of participants according to

the sex.

Table XXXII: Distribution of the adults participating to the program in the

two groups at the pre and the post assessment according to sex

Intervention group

Post n (%)

Control group

Post n (%)

Sex

Pre n (%)

406 (43.2)

534 (56.8)

p

Pre n (%)

271 (28.8)

669 (71.2)

p

Men

442 (44.2)

335 (34.3)

641 (65.7)

0

.67

0.01

women

559 (55.8)

The mean age of participants in the intervention group at the pre

assessment was 37.20 ± 13.22 years old. It was 39.25 ± 13.61 years after

(p = 0.001).

The mean age of participants in the control group at the pre-assessment

was 38.61 ± 13.73 years. It was 40.43 ± 13.96 years after (p = 0.004).

9

4

Figure 12 describes the employment status of participants according to

sex in each group at pre and post assessment.

**2**

**8.5%**

**3**

**6.3%**

**3.1%**

**34.8%**

**3**

**9.7%**

**2.5%**

**6**

**1.4%**

**6**

**6.3%**

**4.2%**

**7**

**5.9%**

**1.2%**

**7**

**9.6%**

**2.5%**

not working

working

**4**

**8.3%**

**3.3%**

**51.5%**

**5**

**5**

**2**

**1**

**6.8%**

**2**

student

**1**

**1**

**2**

**1.8% 10.7% 9.5% 13.7% 12.9%**

**7**

**.8% 8%**

men

women

Pre

Intervention group

men

women

men

women

Pre

men

women

Post

Post

Control group

Figure 12: Distribution of adults participating in the intervention

program in both groups by occupational status

**4**

**.2.3.2. Evolution of the knowledge and perceptions on**

**the risk factors of non-communicable diseases before**

**and after intervention among the participants:**

**For eating habits:**

The adults of Sousse participating in the intervention group improved

their knowledge about the relation between eating habits and the risk

of developing NCDs. This observed improvement was statistically

significant (<0.001) for all of the cardiovascular diseases, diabetes and

cancers. The same for was observed in the control group which

improved significantly this knowledge (Table XXXIII).

9

5

Table XXXIII: Evolution of the knowledge on the link between

alimentation and the risk to develop non-communicable diseases among

the adults participating in the two groups

Do you think that what you

are consuming can change

the risk?

Intervention group

Control group

Pre n (%)

Post n (%)

890 (88.9)

p

Pre n (%)

Post n (%)

p

Cardio-vascular diseases

707 (75.2)

<0.001

715 (76.1)

848 (86.9)

<0.001

Diabetes mellitus

Cancers

793 (84.4)

462 (49.1)

901 (90.0)

850 (84.9)

<0.001

<0.001

777 (82.7)

425 (45.2)

873 (89.4)

802 (82.2)

<0.001

<0.001

Other knowledges were identified among participants (Table XXXIV). In

the control group the proportion of adults recognizing the importance

of changing cooking methods declined significantly (p = 0.006). In the

intervention group, the proportion of adults recognizing the importance

of eating less salt increased significantly (p = 0.02).

9

6

Table XXXIV: Evolution of the participants’ perceptions on the influence

of healthy diet on health before and after the intervention in both

groups

Intervention group

Control group

Post n (%)

It’s important for health

Pre n (%)

97 (95.4)

877 (93.3)

08 (96.6)

Post n (%)

940 (93.9)

p

Pre n (%)

p

Changing cooking

methods

8

0.14

907 (96.5)

915 (93.8)

922 (94.5)

915 (93.8)

0.006

Eating more fibre

943 (94.2)

970 (96.9)

0.40

0.70

903 (96.1)

907 (96.5)

0.10

0.33

Eating more fruits and

vegetables

9

Eating less sugar

Eating less fat

Eating less salt

907 (96.5)

916 (97.4)

891 (94.8)

971 (97.0)

970 (96.9)

970 (96.9)

0.52

0.47

0.02

910 (96.8)

911 (97.0)

906 (96.4)

944 (96.7)

944 (96.7)

941 (96.4)

0.91

0.71

0.97

**For physical activity:**

Adults in the intervention group had significantly improved their

knowledge about all the benefits of practicing 30 minutes of physical

activity daily five days a week (p <0.001). For the control group, there

was a statistically significant improvement in the knowledge that doing

3

0 minutes of physical activity daily, five days a week help to reduce risk

of diseases (p= 0.03), gain muscle (p<0.001) and do better job (p

0.001).

<

The participants' knowledge’s of the benefits of practicing 30 minutes of

physical activity are detailed in the table XXXV below.

9

7

Table XXXV: Evolution of the participants’ knowledge on the benefits of

the practice of 30 minutes of physical activity per day at least 5 days a

week in both groups

practicing thirty minutes of

physical activity per day at least

for 5 days a week

Intervention group

Control group

Post

Pre n (%)

Pre n (%)

p

Pre n (%)

p

n (%)

Reduce risk of diseases

Improve heart and lung fitness

Gain muscle

867 (92.5)

869 (92.6)

812 (86.6)

823 (87.7)

861 (91.8)

855 (91.2)

844 (90.0)

976 (97.7)

973 (97.4)

963 (96.4)

938 (94.0)

969 (97.0)

965 (96.6)

969 (97.1)

<0.001

<0.001

<0.001

<0.001

<0.001

<0.001

<0.001

879 (93.7)

885 (94.2)

847 (90.2)

879 (93.6)

881 (93.8)

887 (94.5)

856 (91.2)

933 (95.9)

931 (95.7)

919 (94.5)

880 (90.4)

921 (94.7)

915 (94.0)

925 (95.1)

0.03

0.15

<0.001

0.01

Help losing weight

Prevent depression

Reduce stress

0.43

0.69

Do better on job

<0.001

**For tobacco use:**

In the intervention group, the proportion of adults who knew that

smoking can cause myocardial infarction (MI) increased significantly

from 88.5% to 91.5% (p = 0.03) but not in the control group. In the

control group the proportion of adults who believed that cigarettes

labeled light aren’t less harmful than regular cigarettes increased from

5

% to 13.2% (p <0.001). However, the proportion of those who thought

that the smoking of narguile is harmful decreased significantly in the

control group (p = 0.02) (Table XXXVI).

9

8

Table XXXVI: Evolution of the knowledge about tobacco use among

adults in the two groups

Intervention group

Control group

Post

Pre n (%)

Pre n (%)

p

Pre n (%)

p

n (%)

Tobacco use can result in cerebral

stroke

0.83

7

8

67 (81.6)

32 (88.5)

885 (88.5)

915 (91.5)

0.68

0.03

793 (84.4)

828 (88.1)

838 (85.9)

862 (88.4)

Tobacco use can result in myocardial

infarction (MI)

0.82

The light cigarette is not less harmful

The smoking of narguile is harmful

143 (15.2)

851 (90.5)

182 (18.2)

925 (92.7)

0.08

0.09

47 (5.0)

129 (13.2)

887 (91.0)

<0.001

0.02

881 (93.7)

**4**

**.2.3.3. Evolution of adult participants behaviors:**

**For eating habits:**

The intervention group decreased by 4.8% the habit of adding salt to

food, this improvement was statistically significant (p = 0.01) from

2

=

6.9% to 22.1%. The control group decreased by 4.1% the same habit (p

0.01) from 15.8% to 11.7%.

We assessed the habit of eating snacks among adults participating in the

study. The intervention group increased not significantly the habit of

choosing fried food. It decreased significantly the habit of choosing

fruits or vegetables (p = 0.04) and the habit of choosing milk and dairy

products (p <0.001). The changes in the control group consisted in a

decrease of fried food consumption, but also a decrease in the

9

9

consumption of fruits and vegetables as well as milk and dairy products

between meals. These trends among the control group were significant

(Table XXXVII).

Table XXXVII: Evolution of snacks consumed by the participants in the

intervention program in the two groups

Intervention group

Control group

Post

Pre n (%)

Pre n (%)

p

Pre n (%)

p

n (%)

Choose fried food

113 (12.1)

141 (14.2)

0.19

124 (13.2)

97 (10.0)

0.03

Choose fruits and vegetables

1

2

89 (20.3)

43 (26.1)

166 (16.7)

168 (16.9)

0.04

263 (28.0)

197 (21.0)

158 (16.2)

117 (12.0)

<0.001

<0.001

Choose milk and dairy

products

<0.001

The intervention group improved significantly (p <0.001) the habit of

eating at least five servings of fruits and vegetables per day from 39.4%

to 58.4%. The same for the control group, a significant improvement (p

<0.001) was observed from 51.4% to 67.9%.

This habit (eating at least 5 servings of fruit and vegetables per day) was

significantly improved in the two groups after adjusting for age and sex

(table XXXIII).

1

00

Table XXXIII: Evolution of the behavior of consuming 5 fruits and

vegetables daily in the two groups according to sex and age

Intervention group

Post n (%)

Control group

Post n (%)

Pre n (%)

p

Pre n (%)

p

Men

161 (39,8)

244 (55,6)

122 (45,0)

234 (69,9)

<

<

<

<

0.001

<0.001

Women

207 (39,1)

202 (37,5)

162 (41,6)

235 (60,6)

257 (51,6)

304 (66,2)

361 (54,0)

272 (51,0)

209 (51,9)

429 (66,9)

317 (64,2)

343 (71,6)

0.001

0.001

0.001

<0.001

<0.001

<0.001

≤

40 years

>

40 years

**For physical activity:**

The adults of Sousse participating to the study improved significantly

the behavior of doing recommended level of physical activity in the two

groups (Table XXXIX).

Furthermore, after adjusting by sex, this improvement was still

significant for men and women in the two groups. However, the

improvement among men (26.3%) was higher than that among women

(8.2%) in the intervention group (Table XXXX).

The same results were seen after adjusting by age, where we noticed

that the improvement is still significant in the two groups (Table XXXX).

1

01

Table XXXIX: Evolution of the behavior of practicing 30 minutes of

physical activity daily during 5 days a week among adults in the two

groups

Intervention group

Control group

Post n (%)

Pre n (%)

Post n (%)

400 (40.1)

p

Pre n (%)

p

yes

no

141 (15.1)

795 (84.9)

141 (15.0)

375 (38.5)

598 (61.5)

<

0.001

<0.001

597 (59.9)

799 (85.0)

Table XXXX: Evolution of the behavior of practicing 30 minutes of

physical activity daily during 5 days a week among the participants in

the two groups according to sex and age

Intervention group

Control group

Post n(%)

Pre n(%)

Post n(%)

195 (44.4)

p

Pre n(%)

62 (22.9)

p

Men

73 (18.1)

57 (18.1)

93 (17.2)

46 (11.8)

<0.001

145 (43.4)

<

<

<

<

0.001

Women

99 (26.3)

209 (39.1)

191 (41.3)

<0.001

<0.001

<0.001

79 (11.8)

76 (14.3)

64 (15.9)

230 (36.0)

200 (40.6)

175 (36.7)

0.001

0.001

0.001

≤

40 years

>

40 years

**For tobacco use:**

In the intervention group, the prevalence of tobacco use was 26.2% at

the pre-assessment and 23.2% after (p = 0.13). In the control group, the

prevalence increased significantly from 14.4% before the intervention to

1

8.3% after (p = 0.02) (Table XXXXI).

1

02

Table XXXXI: Evolution of the prevalence of tobacco use among the

participants

in the two groups

Intervention group

Pre n (%) Post n(%)

232 (23.2)

Control group

post n(%)

Are you a current smoker?

p

Pre n(%)

p

yes

no

242 (26.2)

683 (73.8)

135 (14.4)

178 (18.3)

797 (81.7)

0

.13

0.02

768 (76.8)

804 (85.6)

After adjusting for sex and age (Tableau XXXXII), the prevalence of

tobacco use among men decreased significantly (p=0.03) in the

intervention group from 52.9 % to 45.6 %.

Table XXXXII: Evolution of the prevalence of tobacco use among the

participants in the two groups according to sex and age

Intervention group

Control group

Post n (%)

Pre n (%)

Post n (%)

201(45.6)

p

Pre n (%)

127(46.9)

p

Men

213 (52.9)

169(50.4)

0

0

0

0

.03

0.38

Women

29 (5.6)

31(5.5)

122 (22.8)

110(23.7)

8(1.2)

9(1.4)

.99

.43

.18

0.73

0.07

0.09

≤

40 years

133 (24.9)

106 (27.6)

82(15.4)

52(12.9)

97(19.6)

81(16.9)

>40 years

**4**

**.2.3.4. Evolution of other NCD’s risk factors after the**

**intervention:**

The prevalence of obesity increased significantly in the control group

(p=0.04) (Table XXXXIII).

1

03

Table XXXXIII: Evolution of the obesity and overweight in the two groups

Intervention group

Control group

Post n(%)

Pre n(%)

Post n(%)

351 (35.4)

p

Pre n(%)

p

Overweight

Obesity

308 (33.8)

0.47

292 (31.6)

308 (31.9)

0

0

.88

243(26.7)

296 (29.8)

0.12

271 (29.3)

324 (33.5)

.04

After adjusting for sex and age (Table XXXXIV), the prevalence of

overweight increased significantly from 33.2% to 40.9% (p = 0.02)

among male participants in the intervention group.

Table XXXXIV: Evolution of the obesity and overweight in the two groups

according to the sex and the age

Intervention group

Control group

Post n (%)

Risk factor

Caracteris-tics

Pre n (%)

Post n (%)

179 (40.9)

p

Pre n (%)

91 (34.2)

p

Men

1

1

1

1

30 (33.2)

78 (34.2)

58 (30.0)

49 (39.2)

0.02

126 (38.1)

0.33

Women

172 (31.0)

168 (31.6)

183 (39.7)

78 (17.8)

0.26

0.57

0.88

0.72

0.07

0.33

0.64

201 (30.5)

154 (29.3)

137 (34.6)

53 (19.9)

182 (28.7)

161 (33.0)

146 (30.7)

72 (21.8)

0.56

0.20

0.40

0.58

0.06

0.20

0.71

Over-weight

≤

40 years

40 years

Men

>

6

6 (16.9)

Women

1

1

77 (34.0)

218 (39.4)

102 (19.2)

194 (42.1)

218 (33.1)

86 (16.4)

252 (39.7)

95 (19.5)

Obesity

≤

40 years

8

9 (16.9)

>40 years

54 (40.5)

183 (46.2)

228 (48.0)

The prevalence of hypertension decreased significantly (p = 0.04) in the

intervention group globally from 35.8% to 31.4%. In the control group,

this proportion increased from 29.3% to 30.3% without significant

1

04

difference (p = 0.62). In the intervention group, after adjustment for sex

and age (Table XXXXV), a significant decrease (p = 0.004) in the

prevalence of hypertension was observed for participants aged less than

4

0 years, it decreased from 22.6% to 15.7%. No significant change was

observed in the control group.

Table XXXXV: Evolution of the hypertension in the two groups according

to the sex and the age

Intervention group

Control group

Post n (%)

Hyper-tension

Pre n (%)

Post n (%)

164 (37.4)

p

Pre n (%)

81 (30.2)

p

Men

1

1

1

2

60 (41.6)

65 (31.5)

19 (22.6)

04 (54.0)

0.218

113 (33.7)

0.360

Women

147 (26.6)

83 (15.7)

228 (49.5)

0.079

0.004

0.193

193 (28.9)

73 (13.8)

197 (49.1)

183 (28.5)

73 (14.8)

221 (46.1)

0.877

0.646

0.376

≤

40 years

>40 years

1

05

**5**

**. Discussion, conclusion and perspectives**

**.1. Discussion**

**5**

Noncommunicable diseases (NCDs) are the leading causes of death

globally, killing more people each year than all other causes combined.

Available data show that nearly 80% of deaths from NCDs occur in low-

and middle-income (35). NCDs are mostly the result of unhealthy

behaviors such as poor diet, lack of physical activity, tobacco and

excessive alcohol consumption. Tunisia, which is undergoing an

epidemiological transition, faces the challenge of the fight against non-

communicable diseases and their risk factors (38).

Despite their rapid growth and inequitable distribution, much of the

health and social consequences caused each year by NCD-related deaths

could be avoided by well-known cost-effective and feasible

interventions (35). Thus, the prevention of noncommunicable diseases is

more effective if it is done by a combination of a population and

individual approach to the prevention of major risk factors (65). The

principle of actions at the community level is not only targeting the

community to bring about change in behavior. They also aim to enable

these communities to encourage them to act as an agent of change and

encourage them to use their own resources for action. The overall

strategy includes educating the community to be more informed of

existing risk factors. It also includes assistance to provide simple tools

and technologies to facilitate the choice of healthy lifestyles (66).

1

06

It is in this context that we proposed to evaluate the feasibility and

effectiveness of a community based prevention program for major risk

factors of noncommunicable diseases in the region of Sousse to possibly

generalize later in different region of Tunisia. This program consisted of

an intervention at the community level through a quasi-experimental

study design in three main settings namely the schools, the workplace

and the community. The intervention group was in the delegations of

Sousse Erriadh and Sousse Jawhara and the control group was in the

delegation of Msaken with similar social and economic conditions. A

representative sample in the three settings was used to assess the main

risk factors for noncommunicable diseases in both the intervention and

control group with an assessment before and after the intervention. The

choice of intervention and control areas took into account the distance

between the two groups to avoid any possible contamination but also

the fact that the control area should not be very far to ensure the

feasibility of data collection.

This is the first intervention at the community level done in Tunisia

through a quasi-experimental research design with a control group and

power based samples. However, the absence of randomization in this

case could be the cause of non comparability of the two groups

representing then a certain limit in this study. But more important than

the comparability between the two groups is, rather to be considered,

the comparability intra group before and after the intervention.

1

07

The intervention lasted three years. This time duration may seem

limited compared to other international studies, however we do not

have other studies with such an important time in Tunisia especially as

the conditions under which the intervention took place with the

occurrence of the Tunisian revolution in the middle of the project that

increased the efforts to involve new leaders and adapt to the new socio-

political conditions.

One limitation of this study is that the majority of the tested variables

was reported by the participants themselves. Self-evaluation of

participants for their diet, physical activity and smoking habits could be

biased especially when small changes in behavior were measured.

However, other parameters measured by trained interviewers after

standardization of data collection, have also been improved in the

intervention group such as weight and blood pressure.

The socio-political conditions after the revolution also made it that the

authorities and policy makers at the Ministry of Health and other

sectors (Ministry of Interior, education, finance, urban design...) had

other priorities and challenges than the fight against non-communicable

diseases and their risk factors. Thus, multi-sectoral and structural

measures that are most important in this area were very limited in our

program given the circumstances described above.

1

08

We noticed after all, an improvement of key risk factors in the

intervention group. However, this trend was also observed in the

control group for some variables. We can explain this improvement by

the fact that the control group continued to receive existing awareness

actions and may also have access to certain interventions disseminated

by the media. For obvious ethical considerations, we could not prevent

interventions that already exist in the two groups, but our results

demonstrate the superiority of our intervention that is more intensive

and interactive. On the other hand, contamination is also possible since

the interventions that we have done could reach the control group

because the population of Msaken is not far from the city of Sousse

where we hosted open spaces at large supermarkets for example and

we intervened through awareness programs in local radio that could be

heard in the area Msaken. "Hawthorne" effect is also possible because

the participants were interested in improving their health after being

interviewed.

Behavior change is achieved primarily by the mass media on the one

hand and interpersonal communication on the other. Influencing human

behavior through mass communication is a difficult and complex task

that can compete with existing interests such as the preference of

listeners or advertisments that can generate a lot of money.

Interpersonal communication channels (small group meetings, door to

door, visits, etc.) tend to be more useful in changing attitudes and

behaviors (67). In our program, open days were used to educate the

1

09

general population and provide educational and personalized messages,

so we resorted to widespread household distribution of educational

pamphlets. The use of mass media has been primarily through the local

radio stations as they were the most accessible and especially to

selectively target the intervention community.

To improve the effectiveness and feasibility of our program, several

society actors have been targeted in their respective sectors such as

children in schools and adults in the workplace. Shea et al (68, 69) in a

review of five programs for the prevention of cardiovascular disease

throughout the community have identified effective strategies for

community mobilization: social marketing, education program in middle

school and in the workplace, screening and referral of high-risk

individuals, the education of health professionals and changes in

physical environments. Thus, it is necessary to identify key stakeholders

for effective intervention such as policy makers, citizens, non-

governmental organizations, volunteers and other resources. As a result,

decisions that affect people's health concern not only health services or

"

health policy", but decisions in many policy areas have their influence

on these determinants of health. Indeed, health is strongly influenced

by lifestyle and environment, for example how people live, work, eat

and drink, travel, spend their leisure time, etc ... It is not only individual

choices, but they often have social, cultural, economic and

environmental determinants (70).

1

10

There are many examples of successful interventions at community level

for the prevention and control of noncommunicable diseases in

different parts of the world which include for example the Pawtucket

Heart Health program of the United States of America and the Isfahan

Healthy Heart program in Iran (71-76). Valuable lessons have been

learned from the implementation of projects in developed countries

(77) and evaluation of various community-based interventions (78).

The majority of these programs have targeted several areas as is the

case for our program with the aim of targeting different segments of

society and potentiate the effect of interventions in different sectors.

The school is a privileged area that can target a large number of young

people. The intervention implemented in Finland since 1978 in the

community has, in fact, included a school-based intervention "The North

Karelia Youth Project". This intervention lasted two years, it was

intensive for two colleges, less intensive for the rest of the area colleges

with two reference colleges. It was effective on eating habits, was able

to improve lipid parameters but failed to lower blood pressure. For

smoking habits, intervention could be effective long after. This was

explained by the social approach combined with the involvement of the

media and the community intervention conducted concomitantly (79-

8

1). Thus, to have a successful school program, it must be combined

with other community actions and have a fairly long period of

intervention; this was also observed in the program of Isfahan in Iran

(82, 83).

1

11

The "Child and Adolescent Trial for Cardiovascular Health" program

(CATCH) is one of the largest randomized controlled school trials

sponsored by the National Institutes of Health in the US. Between 1990

and 1994, the intervention program was implemented with a total of

5

106 students in the third year of diverse ethnic backgrounds and

educated in public schools. Students came from 96 schools (56 in the

intervention group and 40 in the control group) in four states (California,

Louisiana, Minnesota and Texas). The program focused on the school

environment and school programs for physical education, nutrition and

smoking. This program has also shown interest in the education of the

whole family. Evaluations were made with schoolchildren, teachers,

specialists in physical education and school canteens staff. At the end,

the intervention was able to change the fat content of school lunches,

increase moderate to vigorous physical activity and improve eating

habits and physical activity.

After a decline of 5 years follow up, the results of the CATCH

intervention were: maintaining the level of energy expenditure and time

spent in moderate intensity physical activity during physical education

classes in intervention groups versus a decline in response in control

groups as well as improved levels of intervention and interest in physical

education classes (84, 85).

Literature reviews on intervention programs in schools to promote

healthy lifestyles among students provides data for the development

1

12

and implementation of effective interventions (86-89). Indeed, the best

interventions are those that include a program on several components

such as intervention in classes, parental and the school involvement

through tasting programs and involvement of school cafeterias. These

studies also emphasize the importance of fruits and vegetables

accessibility (86-89), a suitable environment for physical activity (90, 91)

or restrictions on youth access to tobacco for an effective program (92,

9

3). Another important aspect in the effectiveness of an intervention is

the influence of parents in their behavior and their adherence to the

intervention (94-97).

In our program, the actions on the environment have been limited to

the confines of the colleges by improving access to healthy foods in

cafeterias with encouragement for schoolchildren who consumed foods

and improved anti tobacco law enforcement. However, we believe that

greater involvement of the school staff is necessary for the success of

this kind of program. We need to create a dynamic and a culture to

emphazise the importance of the fight against risk factors for chronic

diseases in several disciplines, not only in the natural sciences or

physical activity courses. It is also important to develop the spirit of

volunteerism or include more interactive sessions in the curriculum of

schoolchildren to target these risk factors. Indeed, teachers need

training and awareness to serve as role models. On the other hand, we

must improve the infrastructure of sport facilities in schools and ensure

1

13

the value of physical activity sessions. Parents were involved through

meetings and workshops conducted in colleges.

Parental involvement can be done in schools but also in the context of

interventions programs in occupational settings where adults spend

most of their time. The work environment has potentially important

effects on the behavior of workers' health and risk factors for several

diseases. Previous research has shown, for example, that stress at work,

long hours at work, social norms supporting tobacco consumption and

shift work contribute to increased smoking habit and are obstacles to

smoking cessation (98-100). Similarly, it is clear that the organization of

work has a role in the prevalence of obesity (101, 102). Therefore, the

WHO states that the importance of health promotion in the workplace is

increasingly recognized (103). Recent research has shown that effective

programs were those that offered multiple interventions on risk factors

combined with a combination of group and individual education (104).

These programs have been successful on both the health of employees

(104) but also with a decrease in the cost of health care needs (105).

Moreover, this benefit may be an argument to convince employers of

the benefits of these actions and make them relevant and acceptable

programs for employees. Indeed, effective interventions for health

promotion in the workplace depend in part on the interest and

willingness of employers to support these programs and employees to

participate (106). We also noticed this in our program since the

1

14

interventions were easier to build and more frequent in companies

where employers were more motivated and involved.

Various intervention actions have been made in different countries with

awareness, care of obesity in the workplace (107), smoking cessation

(107, 108), sometimes with incentives and bonuses (106) for those who

managed to lose weight or quit smoking. Other actions have targeted

businesses cafeterias by improving access to fruits and vegetables (109-

1

11). This promotion followed several channels ranging from

personalized education and through the development of employee skills

to achieve group interventions based on partnerships between

employers and employees the introduction of environmental change

and integrated and community programs (106).

Indeed, structural and environmental changes in the workplace can

improve and enhance the effect of the intervention to promote healthy

lifestyles (112). In our program, we tried in some companies to make

some changes such as banning smoking in the workplace trying to

create spaces for smokers. We also tried to educate businesses

managers to report refreshment foods high in sugar and fat and we

have encouraged them to sell fruit and vegetables. These actions were

limited and unfortunately it seems that it is also the case in many other

international interventions (112). However, there is substantial evidence

that policies that prohibit smoking in the workplace can reduce cigarette

consumption, increase attempts to quit smoking and reduce the overall

1

15

prevalence of smoking (113-115). The smoke-free workplaces were also

effective in reducing exposure to passive smoking (116, 117).

A systematic review published in 2005 (112) has included 13

randomized controlled trials of interventions with environmental

assessment of physical activity, diet and risk indicators for health. This

review concluded that intervention to promote health programs

affecting the environment in the workplace had a significant impact on

the promotion of healthy eating. Indeed, 10 of the 13 studies raised

studies have shown positive effects of certain strategies such as labeling

of food, tools (brochures and posters), broadening the availability of

healthy foods. On the other hand, the three studies that have acted on

the environment to promote physical activity have yielded inconclusive

results. This could be explained by a relatively low methodological

quality (encouraging the use of stairs, spaces to walk outside the

company).

A recent work of the National Institutes of Health in the United States

(107) published in 2010 detailed a quasi-experimental study that

included 227 workers in engines industry to assess the effectiveness of a

four months intervention to promote smoking cessation and weight

control. The intervention program consisted of five call visits handled by

trained telephone counselors using motivational interviewing

techniques. This study had a positive impact on smoking cessation; the

rate of smoking cessation was higher among participants compared to

1

16

those who did not participate. After completion of this four months

intervention, no overall improvement in the management of overweight

was observed. However, smokers who participated in the intervention

program and who stopped smoking gained an average of 2.7 kg.

Another review of the literature (118), published in 2010 included 31

randomized controlled trials with the goal of promoting physical activity,

and / or improving eating habits, weight control, lipid mass, blood

pressure, lipid and / or glucose. Intervention programs were diversified

(counseling, group education, exercises). This literature review has

shown the effectiveness of the intervention program on lifestyles in the

workplace on the control of lipid mass and weight control among people

with cardiovascular risk. Indeed, these at-risk populations appeared to

benefit most from interventions to promote healthy lifestyles. On the

other hand, for the other studied variables, the effectiveness of the

intervention was not proven as it was the case of blood pressure that

has not significantly improved in most studies as well for the

populations at high risk or the general population.

Guidelines for health promotion in the workplace suggest that these

programs should be given at different levels to facilitate sustainable

behavior change. At the individual level, a program should include

several educational strategies. At the organizational level, employers

and the various agencies such as the occupational health should

strengthen and encourage positive action on health (for example, by

1

17

providing healthy food options in the cafeteria). Finally, at the

community level, these programs of health promotion in the workplace

can be actively disseminated by employees to their families and social

networks (119, 120) and represent a major component of interventions

at the community level.

During the last two decades, we have noticed an increased interest in

interventions to promote health at the community level. This is due to

the fact that risk factors for noncommunicable diseases are determined

by individual behaviors that themselves depend largely on social and

environmental context. This has led many researchers and public health

professionals to implement actions and compains to change not only

individual behavior but also social norms, public policy and physical

environment (121, 122). These programs have so many advantages as

they target the population on a large scale and therefore have great

potential to improve the level of risk and morbidity in the general

population rather than approaches that target only a small proportion

of at high risk patients (123).

Among the most effective interventions, one can cite the example of

Finland (124), that started from the 70s in North Karelia in response to

local people's representatives petition for urgent and effective

assistance to reduce the burden of mortality due to coronary heart

disease in the region. In cooperation with the authorities and local and

national experts, as well as WHO, the North Karelia project was

1

18

formulated and implemented to carry out a comprehensive intervention

through community organizations with the action of the population

itself. Multisectoral actions were used, involving health and other

services, schools, NGOs, media campaigns, local media, supermarkets,

food industry, agriculture and legislation. After the initial period of the

project (1972-1977), the interventions were actively enforced

nationally. The main results of this integrated intervention program is

the decline in mortality from ischemic heart disease in people under 65

by 73% after 25 years of intervention. Risk factors for cardiovascular

disease have also decreased, such as smoking, cholesterol, blood

pressure with an increased use of vegetable oils rather than butter. The

key to the success of this program according to Pekka Puska (124) was

the organization of the community, the collaborative work with many

organizations and a strong participation. The intervention program was

flexible, based on continuous monitoring and feedback to take

advantage of opportunities that occur naturally. The intervention used

several strategies: from the use of innovative media, communication

activities and the systematic involvement of primary health care

(particularly GPs and public health nurses) to environmental change,

collaboration with the food industry and changes in policy and

legislation. The project worked closely with the national authorities; its

activities benefited and contributed to the national health policy. Over

the years, the project was expanded to include broader objectives of

integrated prevention of major noncommunicable diseases and health

promotion and the prevention of risks related to lifestyles from

1

19

childhood (11 ). It is on this principle that Finland has developed the

slogan "Health in all policies". In the Finnish view, the core of "health in

all policies is to examine the determinants of health that are primarily

controlled by the policies of sectors other than health. The desire is to

adapt the laws and regulations in the context of policy development at

all levels of governance in Europe (125).

The North Karelia program since served as a model based for

international interventions such as the Isfahan program in Iran that was

proven successful. The heart health program (Isfahan Healthy Heart

Program IHHP) is

a

quasi-experimental intervention with

a

comprehensive and multi-sectoral approach targeting the whole

population (n = 2,180,000) in three districts of central Iran. An

evaluation on samples before (2000 - 2001) and after (2007), was made

in the intervention and control area with adjustment for age, education

level and income area. Samples in 2000-2001 and 2007 included

respectively 6175 and 4719 participants in the intervention area, and

6

339 and 4853 in the control area. Multiple procedures were performed

for the promotion of a healthy diet, regular physical activity, tobacco

control and stress management. The prevalence of abdominal obesity,

hypertension, hypercholesterolemia, hypertriglyceridemia and high LDL-

C decreased significantly in the intervention area compared to the

control area in both sexes. However, the reduction of overweight and

obesity was significant only in women. No changes were significant in

the prevalence of diabetes mellitus (126). The beneficial effects of this

1

20

intervention in changing behavior and promoting healthy lifestyles has

also been demonstrated (127). The process evaluation of the

intervention (128, 129) allowed the researchers to suggest that the

activities of intersectoral action and community collaborations resulting

from the program have created synergies with existing national health

policies, although some of them have not been fully implemented (126).

The anti-smoking law is one example. It prohibits smoking in public

places and the sale of cigarettes to minors, but it was not fully

implemented. The IHHP program used these laws to facilitate certain

procedures. Another national health policy that existed was mandatory

attendance at classes for all young men and women who intend to get

married. In these classes, participants were educated on family planning

and reproductive health. An association that was working with the IHHP

project added a ten-minute training on the promotion of healthy

lifestyles in the course. The comprehensive multisectoral approach

effectively increased the level of intervention in which individuals in the

intervention area were exposed, which resulted in reductions in risk

behaviors that have in turn resulted in favorable changes in the

cardiovascular risk factors (126).

The Stanford project in three communities showed that individual

education for people at high risk associated with the mass media

campaign after one year of intervention was more effective in reducing

the consumption of saturated fats, smoking, mean serum cholesterol

1

21

and systolic blood pressure that that of a mass media campaign alone.

The latter was more effective than if there was no intervention (5).

Other prevention strategies have been conducted in various provinces

of Canada where they focused on the social determinants of health to

promote healthy eating and physical activity in oder to fight against

chronic diseases. One study examined provincial initiatives implemented

st

st

between 1 January 2006 and 1 September 2011 which were classified

interventions on lifestyle, structural and environmental actions. The

results indicate better skills in health promotion, partnerships,

infrastructure change. These interventions have been adapted to each

environment and selected according to the needs and characteristics of

each province were able to ensure the sustainability of these actions

with a lower cost (130, 131).

Thus, community-based prevention and control of cardiovascular

diseases began in Europe and the United States in early 1970. While

most of these programs have been conducted in developed countries,

the strong increase of the burden of noncommunicable diseases in

many developing countries has led to similar activities in these countries

too. Many of them were carried out in conjunction with the "Inter-

Health" WHO program (11), which began in 1986. Its aim was to

demonstrate how an integrated program could be implemented in all

populations parts of the world at all stages of the epidemiological

transition. Since then, a large amount of literature has presented its

findings and discussed their experiences. The results indicated that

1

22

these programs could be generalized, were profitable and could

influence health policy. In the 1980s, there has been a focus of

expanded cardiovascular diseases to noncommunicable diseases (NCDs),

mainly due to common risk factors programs. Currently, we must direct

attention to the developing countries, where the prevalence of

noncommunicable diseases is growing. Theory and experience show

that prevention programs for noncommunicable diseases at the

community level should be planned, implemented and evaluated

according to the principles and clear rules, working with all sectors of

the community, and maintain close contact with national authorities.

Given the burden they represent and globalization, there is a great need

for international collaboration. Practical networks with common but

adaptable to local cultures flexibly guidelines have proven to be very

useful (132).

A significant reduction in the burden of noncommunicable diseases is

possible through interventions at the level of the population, which are

cost effective and can even be generating income, as is the case with

tobacco and alcohol after increasing taxes, for example. But effective

interventions, such as measures of tobacco control and salt reduction,

are not implemented on a large scale due to insufficient political

commitment, as well as non-health sectors, lack of resources and the

limited involvement of key stakeholders. For example, less than 10% of

the world population is fully protected by one of the anti tobacco

1

23

measures contained in the WHO Framework Convention on Tobacco

Control (35).

Many interventions for prevention and control of noncommunicable

diseases exist. However, even in the richest countries, choices must be

made in favor of the highest priority interventions to be implemented

because the health resources are finite and in most countries very

limited. A number of criteria are to consider, including the burden of

disease, cost-effectiveness, equity, feasibility of implementation of

specific interventions and political considerations.

In preparation for the high-level meeting of the United Nations, WHO

has identified a set of evidence called "best buys" interventions that are

not only profitable but also very appropriate and feasible to implement

within the constraints of local health systems in low- and middle-income

countries (133). Of course, many other interventions are available to

reduce chronic diseases in the population or in individuals who can still

contribute to the fight against NCDs (134, 135). WHO has developed a

costing tool for countries to add or replace interventions based on

needs or national priorities (136).

To help countries to fight against this burden, WHO has developed in

May 2013 an action plan for the prevention and control of

noncommunicable diseases for the period 2013-2020 (137). The Global

Action Plan provides Member States and WHO international partners a

roadmap and policy options which, when implemented collectively

1

24

between 2013 and 2020, will contribute to work on nine global targets

for the fight against NCDs to achieve by 2025, a relative reduction of

2

5% of premature mortality by NCDs.

**5**

**.2. Conclusion et perspectives:**

The "Together in Health" project for the prevention of risk

factors for NCD is an example of a loco-regional initiative. This initiative

has shown that it is possible to design and implement activities to

promote health in various settings with the support of the community. It

also showed that such actions can have a positive impact on the risk

factors of NCDs, which could presage a decrease in their burden later

on. Such initiatives can only be beneficial if they became widespread in

oil stains manner within a structure organized by the government. The

results of the project "Together in Health" show that it is time to act.

Prevention of non-communicable diseases and health

promotion, a major public health challenge, go through a citizen

empowerment, community and socio-political commitment. The only

example in the fight against smoking helps confirm because this is not

the action of the health care system alone that will fight against this

flaw. Increasing the price of tobacco, smoking bans in public places,

legislation on advertising gradually contribute to a change in smoking

habits, provided that these procedures are properly applied. Public and

political authorities must continue the efforts already undertaken in

these many areas with the help of civil society through NGOs and health

1

25

professionals. Control programs should take advantage of already

existing laws to increase their chances of success.

Thus, an integrated approach to the prevention and

management of NCDs is needed at the national level. It should be

conducted by the government and benefit from the involvement of the

entire community. It is important to foster multidisciplinary

stakeholders but also to focus on collaborations and synergies between

different sectors related to health (NGO sectors, political, socio-

economic, medical and paramedical).

Another guarantee of success of multidisciplinary actions is the

understanding of the community ("community diagnosis") which will

ensure the proper coordination of all partners and their involvement

with the beneficiaries right from the start of the process. Finally, we

must encourage multiple sites of action and do everything possible to

help facilitate positive health choices (ensuring access to healthy food,

initiate health paths, provide affordable infrastructure for recreation

activities and physical activity, fight against passive smoking). This can

be facilitated by the combination of well-planned across a broad

population and actions involving primary health care, media actions,

voluntary organizations such as NGOs, the food industry and

supermarkets, workplaces, schools and local media...

1

26

Many activities have so far received only limited evaluation, they

are also often considered for the very short term. In addition, it is

especially evaluation of the effectiveness of actions while assessing the

efficiency of actions and methods used and their sustainability would be

equally relevant.

It remains important to address the prevention and health

promotion in a comprehensive and sustainable development. WHO

recommends to assess and monitor the burden imposed by NCDs and

their determinants for public health and incorporates the fight against

this burden in the relevant socio-economic policies of poverty reduction

strategies. It also provides support to the adoption of approaches

involving all departments, ensuring to take appropriate intersectoral

action on public health and focusing on social determinants of NCDs,

particularly with regard to the programs of equitable financing and

access to primary health care.

Finally, given the global burden of noncommunicable diseases

and the impact of globalization on modern lifestyles and health, it is

necessary to have an international collaboration. Practical networks

sharing common guidelines but adapted flexibly to local cultures have

proven to be very useful in this context.

1

27

**6**

**. References**

1

2

3

4

5

. Mendis S, Fuster V: National policies and strategies for

noncommunicable diseases. Nature Reviews Cardiology 2009; 6,

23–727

7

. Nishtar S: Prevention of non-communicable diseases in Pakistan:

an integrated partnership-based model. Health Research Policy

and Systems 2004, 2:7doi:10.1186/1478-4505-2-7

. Alwan A, MacLean D, Mandil A: Assessment of National Capacity

for Non Communicable Diseases Prevention and Control.

Geneva, Switzerland. World Health Organization 2001.

. Wu Y, Liu X, Li X, et al. Estimation of 10-year risk of fatal and

nonfatal ischemic cardiovascular diseases in Chinese adults

Circulation 2006; 114:2217-2225.

. Elder JP, McGraw SA, Abrams DB et al: Organizational and

community approaches to community-wide prevention of heart

disease: The first two years of the pawtucket heart health

program. Preventive Medicine, 1986, 15(2): 107–117

6

7

. Puska P, Salonen JT, Nissinen A et al : Change in risk factors for

coronary heart disease during 10 years of a community

intervention program (North Karelia Project). BMJ 1983; 287:

1

840 - 1844.

. Protocol and guidelines: Countrywide Integrated Non-

communicable Diseases Intervention (CINDI) Programme.

Copenhagen, WHO Regional Office for Europe 1996

1

28

8

9

1

. The world Health Report 2002. Reducing the risks, promoting

healthy life. WHO Geneva 2002.

. Rose G. The Strategy of Preventive Medicine. Oxford: Oxford

University Press, 1992.

0. Beaglehole R, Magnus P: The search for new risk factors for

coronary

heart

disease:

occupational

therapy

for

epidemiologists? Int J Epidemiol 2002; 31(6):1117-22

1

1. Puska P, Tuomilehto J, Nissinen A, Vartiainen E (eds): The North

Karelia Project: 20 years results and experiences, Helsinki,

Helsinky University Printing House 1995.

1

1

2. Alwan A: Global status report on noncommunicable diseases

2

010. World Health Organization 2011

3. [Strong K, Mathers C, Leeder S, Beaglehole R:](http://www.ncbi.nlm.nih.gov/entrez/query.fcgi?cmd=Retrieve&db=pubmed&dopt=Abstract&list_uids=16257345&query_hl=2&itool=pubmed_docsum) Preventing chronic

diseases: how many lives can we save. Lancet 2005;

3

66(9496):1578-82

1

1

4. Mathers CD, Loncar D. Projections of global mortality and burden

of disease from 2002 to 2030. PLoS Med 2006; 3:2011-2030

5. Wild S, Roglic G, Green A, Sicree R, King H[.: Global prevalence of](http://www.ncbi.nlm.nih.gov/pubmed/15111519?ordinalpos=57&itool=EntrezSystem2.PEntrez.Pubmed.Pubmed_ResultsPanel.Pubmed_DefaultReportPanel.Pubmed_RVDocSum)

[diabetes: estimates for the year 2000 and projections for 2030.](http://www.ncbi.nlm.nih.gov/pubmed/15111519?ordinalpos=57&itool=EntrezSystem2.PEntrez.Pubmed.Pubmed_ResultsPanel.Pubmed_DefaultReportPanel.Pubmed_RVDocSum)

Diabetes Care 2004; 27(5):1047-53.

1

1

6. Diabetes Atlas: International Diabetes Federation:

http://www.eatlas.idf.org/index1397.html

7. Plan d'action 2008-2013 pour la Stratégie mondiale de lutte

contre les maladies non transmissibles. Editions de

l’Organisation mondiale de la Santé 2010, Genève.

1

29

1

8. Global Atlas on cardiovascular disease prevention and control:

Published by the World Health Organization (Shanthi Mendis) in

collaboration with the World Heart Federation (Pekka Puska) and

the World Stroke Organization (Bo Norrving). Geneva 2011

1

2

9. Uemura K, Pisa Z. Trends in cardiovascular disease mortality in

industrialized countries since 1950. World Health Stat Q

1

988;41:155-78.

0. Thom TJ. International mortality from heart disease: rates and

trends.

Int J Epidemiol 1989;18 (suppl 1):520-29.

2

2

2

1. The advisory board ; the Victoria Declaration on Heart Health

International Heart. Health Conference. Victoria – Canada 1992.

2. Tuomiletho J. Decline in mortality from coronary heart disease in

North Karelia and other parts of Finland. BMJ 1986;293:1068-71.

3. Martin MJ, Hulley SB, Browner WS, Kuller LH, Wehtworth D.

Serum cholesterol, blood pressure and mortality: implication

from a cohort of 361 662 men. Lancet 1986;2:933-6.

2

2

2

4. Kannel W, Higgins M. Smoking and hypertension as predictors of

cardiovascular risk in population studies. J Hypertens 1990;8:3-8.

5. Farquhar JW, Wood PD, Breitrose H et al. Community education

for cardiovascular health. Lancet 1977;1:1192-5.

6. Puska P, Salonen JT, Nissinen A, et al. Change in risk factors for

coronary heart disease during 10 years of a community

intervention program (North Karelia Project). BMJ

1

983;287:1840-4.

1

30

2

2

7. Dodu SRA. Emergence of cardiovascular disease in developing

countries. Cardiology 1988;75:56-64.

8. Ghannem H, Limam K, Ben Abdelaziz A, Mtiraoui A, Hadj Fredj A,

et al. Facteurs de risque des maladies cardiovasculaires dans

une communauté semi-urbaine du Sahel Tunisien. Rev Epidém et

Santé Publ 1992;40:108-12.

2

9. Omran AR. The epidemiological transition: a theory of the

epidemiology of population change. Millbank Mem Fund Q

1

971;75:509-38.

3

3

0. The impact of chronic disease in Tunisia. Rapport de l’OMS;2002.

http://www.who.int/chp/chronic_disease_report/tunisia.pdf

1. Hajem S. Le système national d’information sur les causes de

décès : composante et principaux résultats pour l’année 2009.

Publication de l’Institut National de Santé Publique, Tunis, 2012.

2. [Saidi O,](http://www.ncbi.nlm.nih.gov/pubmed?term=Saidi%20O%5BAuthor%5D&cauthor=true&cauthor_uid=23658808) [Ben Mansour N,](http://www.ncbi.nlm.nih.gov/pubmed?term=Ben%20Mansour%20N%5BAuthor%5D&cauthor=true&cauthor_uid=23658808) [O'Flaherty M,](http://www.ncbi.nlm.nih.gov/pubmed?term=O'Flaherty%20M%5BAuthor%5D&cauthor=true&cauthor_uid=23658808) [Capewell S,](http://www.ncbi.nlm.nih.gov/pubmed?term=Capewell%20S%5BAuthor%5D&cauthor=true&cauthor_uid=23658808) [Critchley JA,](http://www.ncbi.nlm.nih.gov/pubmed?term=Critchley%20JA%5BAuthor%5D&cauthor=true&cauthor_uid=23658808)

[Ben Romdhane H.:](http://www.ncbi.nlm.nih.gov/pubmed?term=Ben%20Romdhane%20H%5BAuthor%5D&cauthor=true&cauthor_uid=23658808) Analyzing recent coronary heart disease

mortality trends in Tunisia between 1997 and 2009. [PLoS One.](http://www.ncbi.nlm.nih.gov/pubmed/23658808)

3

3

3

2

013 May 3;8(5):e63202. doi: 10.1371/journal.pone.0063202.

3. Ben Romdhane H, Haouala H, Belhani A, Drissa H, Kafsi N, et al.

La transition épidémiologique: ses déterminants et son impact

sur les systèmes de santé à travers l’analyse de la tendance des

maladies cardiovasculaires en Tunisie. Tun Med 2005;83 1-7.

4. World Health Organization. Global health risks: Mortality and

burden of disease attributable to selected major risks. Geneva,

WHO, 2009.

1

31

3

3

5. World Health Organization. Global status report on

noncommunicable diseases 2010. Geneva, WHO, 2010.

6. Gharbi M, Belhani A, Aouidet A, Ben Rayana Ch, Achour A, et al.

Niveau des facteurs de risque cardio - vasculaire dans la

population urbaine et rurale du Cap -Bon : Tunisie. Rev Epidém

et Santé Publ 1996;44:125-32.

3

7. Ghannem H, Maarouf R, Tabka A, Hadj Fredj A, et al. La Triade

obésité, hypertension et troubles de la glycorégulation dans une

population semi-urbaine du Sahel Tunisien. Diabetes Metab

(Paris) 1993;19:310-4.

3

3

8. Ghannem H. The challenge of preventing cardiovascular disease

in Tunisia. Prev Chronic Dis 2006; 3(1): A13.

9. Ben Romdhane H, Skhiri H, Bougatef S, Gharbi D, Ben Alaya N,

Achour N. Surveillance des facteurs de risque des maladies

cardiovasculaires en Tunisie: méthodologie et principaux

résultats. Tun Med 2005;83 (spéc 5):8-13.

4

4

0. [Ben Romdhane H,](http://www.ncbi.nlm.nih.gov/pubmed?term=%22Ben%20Romdhane%20H%22%5BAuthor%5D) [Ben Ali S,](http://www.ncbi.nlm.nih.gov/pubmed?term=%22Ben%20Ali%20S%22%5BAuthor%5D) [Skhiri H](http://www.ncbi.nlm.nih.gov/pubmed?term=%22Skhiri%20H%22%5BAuthor%5D) et [al. Hypertension among](http://www.ncbi.nlm.nih.gov/pubmed/22129519)

[Tunisian adults: results of the TAHINA project.](http://www.ncbi.nlm.nih.gov/pubmed/22129519) [Hypertens Res](http://www.ncbi.nlm.nih.gov/pubmed)

2

011 Dec 1. doi: 10.1038/hr.2011.198.

1. Laouani KC, Hmouda H, Ben Naceur MH, Ghannem H, Toumi S, et

al. Hypertension artérielle du sujet de plus de 60 ans: enquête

épidémiologique dans la région de Sousse (Tunisie). Tun Med

2

004; 82(4):1001-5.

1

32

4

2. [Hammami S,](http://www.ncbi.nlm.nih.gov/pubmed?term=%22Hammami%20S%22%5BAuthor%5D) [Mehri S,](http://www.ncbi.nlm.nih.gov/pubmed?term=%22Mehri%20S%22%5BAuthor%5D) [Hajem S](http://www.ncbi.nlm.nih.gov/pubmed?term=%22Hajem%20S%22%5BAuthor%5D) et [al. Awareness, treatment and](http://www.ncbi.nlm.nih.gov/pubmed/22044442)

[control of hypertension among the elderly living in their home in](http://www.ncbi.nlm.nih.gov/pubmed/22044442)

[Tunisia.](http://www.ncbi.nlm.nih.gov/pubmed/22044442) [BMC Cardiovasc Disord](http://www.ncbi.nlm.nih.gov/pubmed) 2011 Nov 1;11(1):65. [Epub

ahead of print]

4

4

3. WHO, 2008; WHO report on the global tobacco epidemic, 2008:

the MPOWER package. Geneva:

4. Peto and Lopez, 2001 Future worldwide health effects of current

smoking patterns. In: Koop CE, Pearson CE, Schwarz MR, eds.

Critical issues in global health. San Francisco, Wiley (Hossey-

Bass) 2001:154-161.

4

4

4

5. Fakhfakh R, Hsairi M, Maalej M, Achour N, Nacef T. Tabagisme

en Tunisie : comportements et connaissances. Bulletin de

l’Organisation mondiale de la Santé 2002;7:60-6.

6. Office National de la famille et de la population. Etude tunisienne

de la santé de la famille. Rapport principal. Tunis:Publications de

l’ONFP, 2001 : 130-2.

7. [Essais O,](http://www.ncbi.nlm.nih.gov/pubmed?term=%22Essais%20O%22%5BAuthor%5D) [Jabrane J,](http://www.ncbi.nlm.nih.gov/pubmed?term=%22Jabrane%20J%22%5BAuthor%5D) [Bouguerra R](http://www.ncbi.nlm.nih.gov/pubmed?term=%22Bouguerra%20R%22%5BAuthor%5D) et al. [Distribution and](http://www.ncbi.nlm.nih.gov/pubmed/20180352)

[prevalence of dyslipidemia in Tunisia: results of Tunisian National](http://www.ncbi.nlm.nih.gov/pubmed/20180352)

[Nutrition Survey.](http://www.ncbi.nlm.nih.gov/pubmed/20180352) [Tunis Med](http://www.ncbi.nlm.nih.gov/pubmed) 2009;87(8):505-10.

[4](http://www.idf.org/diabetesatlas/5e/fr/le-fardeau-mondial?language=fr)

4

[8. http://www.idf.org/diabetesatlas/5e/fr/le-fardeau-](http://www.idf.org/diabetesatlas/5e/fr/le-fardeau-mondial?language=fr)

[mondial?language=fr](http://www.idf.org/diabetesatlas/5e/fr/le-fardeau-mondial?language=fr)

9. Henry-Amar M, Papoz L, Ben Khalifa F, Khaled K, Eschwege E,

Ben Ayed H. Prevalence of diabetes in a random sample from the

gouvernorat of Tunis. Rev Epidém et Santé Publ 1981;29(1):1-13.

1

33

5

0. [Gharbi M,](http://www.ncbi.nlm.nih.gov/pubmed?term=%22Gharbi%20M%22%5BAuthor%5D) [Akrout M,](http://www.ncbi.nlm.nih.gov/pubmed?term=%22Akrout%20M%22%5BAuthor%5D) [Zouari B.](http://www.ncbi.nlm.nih.gov/pubmed?term=%22Zouari%20B%22%5BAuthor%5D) Prevalence and risk factors of non-

insulin-dependent diabetes mellitus in the rural and urban

population of Tunisia. [Rev Epidemiol Sante Publique](http://www.ncbi.nlm.nih.gov/pubmed) 2002;

5

0(4):349-55

5

5

5

1. [Bouguerra R,](http://www.ncbi.nlm.nih.gov/pubmed?term=%22Bouguerra%20R%22%5BAuthor%5D) [Alberti H,](http://www.ncbi.nlm.nih.gov/pubmed?term=%22Alberti%20H%22%5BAuthor%5D) [Salem LB](http://www.ncbi.nlm.nih.gov/pubmed?term=%22Salem%20LB%22%5BAuthor%5D) et al. [The global diabetes](http://www.ncbi.nlm.nih.gov/pubmed/16900086)

[pandemic: the Tunisian experience.](http://www.ncbi.nlm.nih.gov/pubmed/16900086) [Eur J Clin Nutr](http://www.ncbi.nlm.nih.gov/pubmed) 2007;

6

1(2):160-5.

2. Ben Romdhane H et al: Prevalence of diabetes in Northern

African countries: the case of Tunisia BMC Public Health. 2014

Jan 28;14:86. doi: 10.1186/1471-2458-14-86.

3. Allal-Elasmi M, Haj Taieb S, Hsairi M et al. [The metabolic](file://pubmed/20202880)

[syndrome: prevalence, main characteristics and association with](file://pubmed/20202880)

[socio-economic status in adults living in Great Tunis.](file://pubmed/20202880) [Diabetes](file:///M:/AppData/Local/Microsoft/AppData/Local/Microsoft/Windows/Temporary%20Internet%20Files/Content.IE5/0L3XHGGP/user/AppData/Local/Microsoft/Windows/HP/Documents/MNT%20Tunisie/obÃÂ©sitÃÂ©.htm)

[Metab](file:///M:/AppData/Local/Microsoft/AppData/Local/Microsoft/Windows/Temporary%20Internet%20Files/Content.IE5/0L3XHGGP/user/AppData/Local/Microsoft/Windows/HP/Documents/MNT%20Tunisie/obÃÂ©sitÃÂ©.htm) 2010 Jun;36(3):204-8.

5

5

5

4. Bouguerra R, Ben Salem L, Alberti H et al. [Prevalence of](file://pubmed/16799397)

[metabolic abnormalities in the Tunisian adults: a population](file://pubmed/16799397)

[based study.](file://pubmed/16799397) [Diabetes Metab](file:///M:/AppData/Local/Microsoft/AppData/Local/Microsoft/Windows/Temporary%20Internet%20Files/Content.IE5/0L3XHGGP/user/AppData/Local/Microsoft/Windows/HP/Documents/MNT%20Tunisie/obÃÂ©sitÃÂ©.htm) 2006;32(3):215-21.

5. Kamoun M, Hajem S, Imen S, Achour N, Slimane H[. Prevalence of](file://pubmed/19472725)

[obesity and overweight in Tunisia on 2001.](file://pubmed/19472725) [Tunis Med](file:///M:/AppData/Local/Microsoft/AppData/Local/Microsoft/Windows/Temporary%20Internet%20Files/Content.IE5/0L3XHGGP/user/AppData/Local/Microsoft/Windows/HP/Documents/MNT%20Tunisie/obÃÂ©sitÃÂ©.htm) 2008

Jul;86(7):649-52.

6. Beltaïfa L, Traissac P, El Ati J, Lefèvre P, Romdhane HB, Delpeuch

F. [Prevalence of obesity and associated socioeconomic factors](file://pubmed/19037895)

[among Tunisian women from different living environments.](file://pubmed/19037895) [Obes](file:///M:/AppData/Local/Microsoft/AppData/Local/Microsoft/Windows/Temporary%20Internet%20Files/Content.IE5/0L3XHGGP/user/AppData/Local/Microsoft/Windows/HP/Documents/MNT%20Tunisie/obÃÂ©sitÃÂ©.htm)

[Rev](file:///M:/AppData/Local/Microsoft/AppData/Local/Microsoft/Windows/Temporary%20Internet%20Files/Content.IE5/0L3XHGGP/user/AppData/Local/Microsoft/Windows/HP/Documents/MNT%20Tunisie/obÃÂ©sitÃÂ©.htm) 2009 Mar;10(2):145-53.

1

34

5

7. [El Ati J,](http://www.ncbi.nlm.nih.gov/pubmed?term=El%20Ati%20J%5BAuthor%5D&cauthor=true&cauthor_uid=23118943) [Traissac P,](http://www.ncbi.nlm.nih.gov/pubmed?term=Traissac%20P%5BAuthor%5D&cauthor=true&cauthor_uid=23118943) [Delpeuch F,](http://www.ncbi.nlm.nih.gov/pubmed?term=Delpeuch%20F%5BAuthor%5D&cauthor=true&cauthor_uid=23118943) [Aounallah-Skhiri H,](http://www.ncbi.nlm.nih.gov/pubmed?term=Aounallah-Skhiri%20H%5BAuthor%5D&cauthor=true&cauthor_uid=23118943) [Béji C,](http://www.ncbi.nlm.nih.gov/pubmed?term=B%C3%A9ji%20C%5BAuthor%5D&cauthor=true&cauthor_uid=23118943)

[Eymard-Duvernay S,](http://www.ncbi.nlm.nih.gov/pubmed?term=Eymard-Duvernay%20S%5BAuthor%5D&cauthor=true&cauthor_uid=23118943) [Bougatef S,](http://www.ncbi.nlm.nih.gov/pubmed?term=Bougatef%20S%5BAuthor%5D&cauthor=true&cauthor_uid=23118943) [Kolsteren P,](http://www.ncbi.nlm.nih.gov/pubmed?term=Kolsteren%20P%5BAuthor%5D&cauthor=true&cauthor_uid=23118943) [Maire B,](http://www.ncbi.nlm.nih.gov/pubmed?term=Maire%20B%5BAuthor%5D&cauthor=true&cauthor_uid=23118943) [Ben](http://www.ncbi.nlm.nih.gov/pubmed?term=Ben%20Romdhane%20H%5BAuthor%5D&cauthor=true&cauthor_uid=23118943)

[Romdhane H.](http://www.ncbi.nlm.nih.gov/pubmed?term=Ben%20Romdhane%20H%5BAuthor%5D&cauthor=true&cauthor_uid=23118943) : Gender obesity inequities are huge but differ

greatly according to environment and socio-economics in a

North African setting: a national cross-sectional study in Tunisia.

PLoS One. 2012;7(10):e48153. doi: 10.1371/ journal.pone.0048153.

Epub 2012 Oct 31

5

8. [Guthold R,](http://www.ncbi.nlm.nih.gov/pubmed?term=Guthold%20R%5BAuthor%5D&cauthor=true&cauthor_uid=18471584) [Ono T,](http://www.ncbi.nlm.nih.gov/pubmed?term=Ono%20T%5BAuthor%5D&cauthor=true&cauthor_uid=18471584) [Strong KL,](http://www.ncbi.nlm.nih.gov/pubmed?term=Strong%20KL%5BAuthor%5D&cauthor=true&cauthor_uid=18471584) [Chatterji S,](http://www.ncbi.nlm.nih.gov/pubmed?term=Chatterji%20S%5BAuthor%5D&cauthor=true&cauthor_uid=18471584) [Morabia A.:](http://www.ncbi.nlm.nih.gov/pubmed?term=Morabia%20A%5BAuthor%5D&cauthor=true&cauthor_uid=18471584) Worldwide

variability in physical inactivity a 51-country survey. Am J Prev

Med 2008;34(6):486–494

5

6

9. World Health Organization. Equity, social determinants and

public health programmes. Geneva, WHO, 2010.

0. World Health Organization. Closing the gap in generation: Health

equality through action on the social determinants of health.

Final Report of the Commission on Social Determinants of

Health. Geneva, WHO, 2008

6

6

1. Cole TJ, Bellizzi MC, Flegal KM, Dietz WH. Establishing a standard

definition for child overweight and obesity worldwide:

international survey. BMJ 2000; 320: 1-6.

2. Chobanian AV, Bakris GL, Black HR, et al. The seventh report of

the Joint National Committee on Prevention, Detection,

Evaluation, and Treatment of High Blood Pressure: the JNC 7

Report. JAMA 2003;289:2560-2572.

1

35

6

6

3. Warren CW, Jones NR, Eriksen MP, Asma S. Patterns of global

tobacco use in young people and implications for future chronic

disease burden in adults. Lancet 2006; 367: 749–53.

4. WHO. Global recommendation on physical activity for health.

2

011. http://www.who.int/dietphysicalactivity/physical-activity-

recommendations-5-17years.pdf?ua=1

http://www.who.int/dietphysicalactivity/physical-activity-

recommendations-18-64years.pdf

6

6

5. Lewis B, Rose G. Prevention of coronary heart disease:

putting theory into practice. J R Coll Physicians Lond 1991; 25(1) :

2

1–26.

6. Baker E A, Brownson C A. Defining characteristics of community-

based health promotion programs. [J Public Health Manag Pract](http://www.ncbi.nlm.nih.gov/pubmed/?term=Defining+characteristics+of+community-based+health+promotion+programs.)

1

998; 4(2): 1–9.

6

6

7. Rogers E M. Diffusion of Innovation, 4th edition. New York : Free

Press;1995

8. Shea S, Basch C E. A review of five major community-based

cardiovascular disease prevention programs. Part I: Rationale,

design, and theoretical framework. Am J Health Promot 1990 Jan-

Feb;4(3):203-13.

6

9. [Shea S,](http://www.ncbi.nlm.nih.gov/pubmed?term=Shea%20S%5BAuthor%5D&cauthor=true&cauthor_uid=10106505) [Basch CE.](http://www.ncbi.nlm.nih.gov/pubmed?term=Basch%20CE%5BAuthor%5D&cauthor=true&cauthor_uid=10106505) A review of five major community-based

cardiovascular disease prevention programs. Part II: Intervention

strategies, evaluation method and results. [Am J Health](http://www.ncbi.nlm.nih.gov/pubmed/10106505)

[Promot](http://www.ncbi.nlm.nih.gov/pubmed/10106505) 1990 Mar-Apr;4(4):279-87.

1

36

7

7

0. World Health Organization. Social Determinants of Health. The

solid facts. 2nd edition. Wilkinson R, Marmot M, editors.

Geneva,WHO, 2003.

1. [Stern MP,](http://www.ncbi.nlm.nih.gov/pubmed?term=Stern%20MP%5BAuthor%5D&cauthor=true&cauthor_uid=975479) [Farquhar JW,](http://www.ncbi.nlm.nih.gov/pubmed?term=Farquhar%20JW%5BAuthor%5D&cauthor=true&cauthor_uid=975479) [McCoby N,](http://www.ncbi.nlm.nih.gov/pubmed?term=McCoby%20N%5BAuthor%5D&cauthor=true&cauthor_uid=975479) [Russell SH.](http://www.ncbi.nlm.nih.gov/pubmed?term=Russell%20SH%5BAuthor%5D&cauthor=true&cauthor_uid=975479) Result of a two-

year health education campaign on dietary behavior. The

Stanford

Three

Community

Study.

[Circulation](http://www.ncbi.nlm.nih.gov/pubmed/?term=Result+of+a+two-year+health+education+campaign+on+dietary+behavior.+Circulation) 1976

Nov;54(5):826-33

7

7

7

2. [Fortmann SP,](http://www.ncbi.nlm.nih.gov/pubmed?term=Fortmann%20SP%5BAuthor%5D&cauthor=true&cauthor_uid=7293934) [Williams PT,](http://www.ncbi.nlm.nih.gov/pubmed?term=Williams%20PT%5BAuthor%5D&cauthor=true&cauthor_uid=7293934) [Hulley SB,](http://www.ncbi.nlm.nih.gov/pubmed?term=Hulley%20SB%5BAuthor%5D&cauthor=true&cauthor_uid=7293934) [Haskell WL,](http://www.ncbi.nlm.nih.gov/pubmed?term=Haskell%20WL%5BAuthor%5D&cauthor=true&cauthor_uid=7293934) [Farquhar JW.](http://www.ncbi.nlm.nih.gov/pubmed?term=Farquhar%20JW%5BAuthor%5D&cauthor=true&cauthor_uid=7293934)

Effect of health education on dietary behavior: the Stanford 3

community study. [Am J Clin Nutr](http://www.ncbi.nlm.nih.gov/pubmed/?term=Effect+of+health+education+on+dietary+behavior%3A+the+Stanford+3+community+study) 1981 Oct;34(10):2030-8.

3. [Farquhar JW,](http://www.ncbi.nlm.nih.gov/pubmed?term=Farquhar%20JW%5BAuthor%5D&cauthor=true&cauthor_uid=4014215) [Fortmann SP,](http://www.ncbi.nlm.nih.gov/pubmed?term=Fortmann%20SP%5BAuthor%5D&cauthor=true&cauthor_uid=4014215) [Maccoby N,](http://www.ncbi.nlm.nih.gov/pubmed?term=Maccoby%20N%5BAuthor%5D&cauthor=true&cauthor_uid=4014215) [Haskell WL,](http://www.ncbi.nlm.nih.gov/pubmed?term=Haskell%20WL%5BAuthor%5D&cauthor=true&cauthor_uid=4014215) [Williams](http://www.ncbi.nlm.nih.gov/pubmed?term=Williams%20PT%5BAuthor%5D&cauthor=true&cauthor_uid=4014215)

[PT,](http://www.ncbi.nlm.nih.gov/pubmed?term=Williams%20PT%5BAuthor%5D&cauthor=true&cauthor_uid=4014215) [Flora JA](http://www.ncbi.nlm.nih.gov/pubmed?term=Flora%20JA%5BAuthor%5D&cauthor=true&cauthor_uid=4014215) et al. The Stanford five-city project: design and

methods. [Am J Epidemiol](http://www.ncbi.nlm.nih.gov/pubmed/4014215) 1985;122(2):323-34.

4. Mittelmark M B, Luepker R V, Jacobs D R, Bracht N F, Carlaw R

W, Crow R S et al. Community-wide prevention and

cardiovascular disease: education strategies of the Minnesota

Heart Health Program. [Prev Med](http://www.ncbi.nlm.nih.gov/pubmed/3714655) 1986 Jan;15(1):1-17.

7

7

5. [Lefebvre RC,](http://www.ncbi.nlm.nih.gov/pubmed?term=Lefebvre%20RC%5BAuthor%5D&cauthor=true&cauthor_uid=3227200) [Lasater TM,](http://www.ncbi.nlm.nih.gov/pubmed?term=Lasater%20TM%5BAuthor%5D&cauthor=true&cauthor_uid=3227200) [Assaf AR,](http://www.ncbi.nlm.nih.gov/pubmed?term=Assaf%20AR%5BAuthor%5D&cauthor=true&cauthor_uid=3227200) [Carleton RA.](http://www.ncbi.nlm.nih.gov/pubmed?term=Carleton%20RA%5BAuthor%5D&cauthor=true&cauthor_uid=3227200) Pawtucket Heart

Health Program: the process of stimulating community change.

[Scand J Prim Health Care Suppl](http://www.ncbi.nlm.nih.gov/pubmed/3227200) 1988;1:31-7.

6. [Baghaei A,](http://www.ncbi.nlm.nih.gov/pubmed?term=Baghaei%20A%5BAuthor%5D&cauthor=true&cauthor_uid=22371716) [Sarrafzadegan N,](http://www.ncbi.nlm.nih.gov/pubmed?term=Sarrafzadegan%20N%5BAuthor%5D&cauthor=true&cauthor_uid=22371716) [Rabiei K,](http://www.ncbi.nlm.nih.gov/pubmed?term=Rabiei%20K%5BAuthor%5D&cauthor=true&cauthor_uid=22371716) [Gharipour M,](http://www.ncbi.nlm.nih.gov/pubmed?term=Gharipour%20M%5BAuthor%5D&cauthor=true&cauthor_uid=22371716) [Tavasoli](http://www.ncbi.nlm.nih.gov/pubmed?term=Tavasoli%20AA%5BAuthor%5D&cauthor=true&cauthor_uid=22371716)

[AA,](http://www.ncbi.nlm.nih.gov/pubmed?term=Tavasoli%20AA%5BAuthor%5D&cauthor=true&cauthor_uid=22371716) [Shirani S](http://www.ncbi.nlm.nih.gov/pubmed?term=Shirani%20S%5BAuthor%5D&cauthor=true&cauthor_uid=22371716) et al. How effective are strategies for non

communicable disease prevention and control in a high risk

population in a developing country? Isfahan Healthy Heart

Programme[. Arch Med Sci](http://www.ncbi.nlm.nih.gov/pubmed/?term=How+effective+are+strategies+for+non+communicable+disease+prevention+and+control+in+a+high+risk+population+in+a+developing+country) 2010 Mar 1;6(1):24-31.

1

37

7

7

7. [Brownson RC,](http://www.ncbi.nlm.nih.gov/pubmed?term=Brownson%20RC%5BAuthor%5D&cauthor=true&cauthor_uid=10186735) [Riley P,](http://www.ncbi.nlm.nih.gov/pubmed?term=Riley%20P%5BAuthor%5D&cauthor=true&cauthor_uid=10186735) [Bruce TA.](http://www.ncbi.nlm.nih.gov/pubmed?term=Bruce%20TA%5BAuthor%5D&cauthor=true&cauthor_uid=10186735) Demonstration projects in

community-based prevention[. J Public Health Manag Pract](http://www.ncbi.nlm.nih.gov/pubmed/?term=79.%09Brownson%2C+R.+C.%2C+Riley%2C+P.+and+Bruce%2C+T.+A.++Demonstration+projects+in+community-based+prevention.+Journal+of+Public+Health+Management+Practice%2C+4%2C+66%E2%80%9377.) 1998

Mar;4(2):66-77.

8. Sellers D E, Crawford S L, Bullock K, Mckinlay J B. Understanding

the variability in the effectiveness of community heart health

program: a meta-analysis. [Soc Sci Med](http://www.ncbi.nlm.nih.gov/pubmed/?term=Understanding+the+variability+in+the+effectiveness+of+community+heart+health+program%3A+a+meta-analysis) 1997 May;44(9):1325-39.

7

8

9. [Vartiainen E,](http://www.ncbi.nlm.nih.gov/pubmed?term=Vartiainen%20E%5BAuthor%5D&cauthor=true&cauthor_uid=9584038) [Paavola M,](http://www.ncbi.nlm.nih.gov/pubmed?term=Paavola%20M%5BAuthor%5D&cauthor=true&cauthor_uid=9584038) [McAlister A,](http://www.ncbi.nlm.nih.gov/pubmed?term=McAlister%20A%5BAuthor%5D&cauthor=true&cauthor_uid=9584038) [Puska P.](http://www.ncbi.nlm.nih.gov/pubmed?term=Puska%20P%5BAuthor%5D&cauthor=true&cauthor_uid=9584038) Fifteen-year follow

up of smoking prevention effects in the North Karelia youth project.

Am J Public Health 1998 Jan;88(1):81-5.

0. [Vartiainen E,](http://www.ncbi.nlm.nih.gov/pubmed?term=Vartiainen%20E%5BAuthor%5D&cauthor=true&cauthor_uid=6981903) [Puska P,](http://www.ncbi.nlm.nih.gov/pubmed?term=Puska%20P%5BAuthor%5D&cauthor=true&cauthor_uid=6981903) [Pallonen U,](http://www.ncbi.nlm.nih.gov/pubmed?term=Pallonen%20U%5BAuthor%5D&cauthor=true&cauthor_uid=6981903) [Pöyhiä P.](http://www.ncbi.nlm.nih.gov/pubmed?term=P%C3%B6yhi%C3%A4%20P%5BAuthor%5D&cauthor=true&cauthor_uid=6981903) Effects of two years'

educational intervention on dietary habits, serum cholesterol

and blood pressure among 13 to 15 year old adolescents.

The North Karelia youth project. [Acta Cardiol](http://www.ncbi.nlm.nih.gov/pubmed/6981903) 1982;37(3):199

1. [Vartiainen E,](http://www.ncbi.nlm.nih.gov/pubmed?term=Vartiainen%20E%5BAuthor%5D&cauthor=true&cauthor_uid=19959603) [Laatikainen T,](http://www.ncbi.nlm.nih.gov/pubmed?term=Laatikainen%20T%5BAuthor%5D&cauthor=true&cauthor_uid=19959603) [Peltonen M,](http://www.ncbi.nlm.nih.gov/pubmed?term=Peltonen%20M%5BAuthor%5D&cauthor=true&cauthor_uid=19959603) [Juolevi A,](http://www.ncbi.nlm.nih.gov/pubmed?term=Juolevi%20A%5BAuthor%5D&cauthor=true&cauthor_uid=19959603) Männistö

S, Sundvall J et al. Thirty-five year trends in cardiovascular risk

factors in Finland. Int J Epidemiol 2010 Apr;39(2):504-18.

2. [Kelishadi R,](http://www.ncbi.nlm.nih.gov/pubmed?term=Kelishadi%20R%5BAuthor%5D&cauthor=true&cauthor_uid=15351543) [Pour MH,](http://www.ncbi.nlm.nih.gov/pubmed?term=Pour%20MH%5BAuthor%5D&cauthor=true&cauthor_uid=15351543) [Zadegan NS,](http://www.ncbi.nlm.nih.gov/pubmed?term=Zadegan%20NS%5BAuthor%5D&cauthor=true&cauthor_uid=15351543) [Kahbazi M,](http://www.ncbi.nlm.nih.gov/pubmed?term=Kahbazi%20M%5BAuthor%5D&cauthor=true&cauthor_uid=15351543) [Sadry G,](http://www.ncbi.nlm.nih.gov/pubmed?term=Sadry%20G%5BAuthor%5D&cauthor=true&cauthor_uid=15351543) [Amani A](http://www.ncbi.nlm.nih.gov/pubmed?term=Amani%20A%5BAuthor%5D&cauthor=true&cauthor_uid=15351543)

et al. Dietary fat intake and lipid profiles of Iranian adolescents:

Isfahan Healthy Heart Program-Heart Health Promotion from

Childhood[. Prev Med](http://www.ncbi.nlm.nih.gov/pubmed/15351543) 2004 Oct;39(4):760-6

8

8

8

3. [Mohammadifard N,](http://www.ncbi.nlm.nih.gov/pubmed?term=Mohammadifard%20N%5BAuthor%5D&cauthor=true&cauthor_uid=23696765) [Sarrafzadegan N,](http://www.ncbi.nlm.nih.gov/pubmed?term=Sarrafzadegan%20N%5BAuthor%5D&cauthor=true&cauthor_uid=23696765) [Ghassemi GR,](http://www.ncbi.nlm.nih.gov/pubmed?term=Ghassemi%20GR%5BAuthor%5D&cauthor=true&cauthor_uid=23696765) [Nouri](http://www.ncbi.nlm.nih.gov/pubmed?term=Nouri%20F%5BAuthor%5D&cauthor=true&cauthor_uid=23696765)

[F,](http://www.ncbi.nlm.nih.gov/pubmed?term=Nouri%20F%5BAuthor%5D&cauthor=true&cauthor_uid=23696765) Pashmi R. Alteration in unhealthy nutrition behaviors in

adolescents through community intervention: Isfahan Healthy

HeartProgram. ARYA Atheroscler 2013 Jan;9(1):89-97

1

38

8

8

4. [McKenzie TL,](http://www.ncbi.nlm.nih.gov/pubmed?term=McKenzie%20TL%5BAuthor%5D&cauthor=true&cauthor_uid=8818066) [Nader PR,](http://www.ncbi.nlm.nih.gov/pubmed?term=Nader%20PR%5BAuthor%5D&cauthor=true&cauthor_uid=8818066) [Strikmiller PK,](http://www.ncbi.nlm.nih.gov/pubmed?term=Strikmiller%20PK%5BAuthor%5D&cauthor=true&cauthor_uid=8818066) [Yang M,](http://www.ncbi.nlm.nih.gov/pubmed?term=Yang%20M%5BAuthor%5D&cauthor=true&cauthor_uid=8818066) [Stone EJ,](http://www.ncbi.nlm.nih.gov/pubmed?term=Stone%20EJ%5BAuthor%5D&cauthor=true&cauthor_uid=8818066) [Perry CL](http://www.ncbi.nlm.nih.gov/pubmed?term=Perry%20CL%5BAuthor%5D&cauthor=true&cauthor_uid=8818066)

et al. School physical education: effect of the Child and

Adolescent Trial for Cardiovascular Health. [Prev Med](http://www.ncbi.nlm.nih.gov/pubmed/8818066) 1996 Jul-

Aug;25(4):423-31

5. [Nader PR1,](http://www.ncbi.nlm.nih.gov/pubmed?term=Nader%20PR%5BAuthor%5D&cauthor=true&cauthor_uid=10401802) [Stone EJ,](http://www.ncbi.nlm.nih.gov/pubmed?term=Stone%20EJ%5BAuthor%5D&cauthor=true&cauthor_uid=10401802) [Lytle LA,](http://www.ncbi.nlm.nih.gov/pubmed?term=Lytle%20LA%5BAuthor%5D&cauthor=true&cauthor_uid=10401802) [Perry CL,](http://www.ncbi.nlm.nih.gov/pubmed?term=Perry%20CL%5BAuthor%5D&cauthor=true&cauthor_uid=10401802) [Osganian SK,](http://www.ncbi.nlm.nih.gov/pubmed?term=Osganian%20SK%5BAuthor%5D&cauthor=true&cauthor_uid=10401802) [Kelder S](http://www.ncbi.nlm.nih.gov/pubmed?term=Kelder%20S%5BAuthor%5D&cauthor=true&cauthor_uid=10401802) et

al. Three-year maintenance of improved diet and physical

activity: the CATCH cohort. Child and Adolescent Trial for

Cardiovascular Health. [Arch Pediatr Adolesc Med](http://www.ncbi.nlm.nih.gov/pubmed/10401802) 1999

Jul;153(7):695-704.

8

8

6. [Hoelscher DM,](http://www.ncbi.nlm.nih.gov/pubmed?term=Hoelscher%20DM%5BAuthor%5D&cauthor=true&cauthor_uid=11902389) [Evans A,](http://www.ncbi.nlm.nih.gov/pubmed?term=Evans%20A%5BAuthor%5D&cauthor=true&cauthor_uid=11902389) [Parcel GS,](http://www.ncbi.nlm.nih.gov/pubmed?term=Parcel%20GS%5BAuthor%5D&cauthor=true&cauthor_uid=11902389) [Kelder SH.](http://www.ncbi.nlm.nih.gov/pubmed?term=Kelder%20SH%5BAuthor%5D&cauthor=true&cauthor_uid=11902389) Designing effective

nutrition interventions for adolescents. [J Am Diet Assoc](http://www.ncbi.nlm.nih.gov/pubmed/?term=89.%09Hoelscher+DM%2C+Evans+A%2C+Parcel+GS%2C+et+al.+Designing+effective+nutrition+interventions+for+adolescents.+J+Am+Diet+Assoc+2002%3B102%3AS52%E2%80%93S63.) 2002

Mar;102(3 Suppl):S52-63.

7. [van der Horst K,](http://www.ncbi.nlm.nih.gov/pubmed?term=van%20der%20Horst%20K%5BAuthor%5D&cauthor=true&cauthor_uid=16861362) [Oenema A,](http://www.ncbi.nlm.nih.gov/pubmed?term=Oenema%20A%5BAuthor%5D&cauthor=true&cauthor_uid=16861362) [Ferreira I,](http://www.ncbi.nlm.nih.gov/pubmed?term=Ferreira%20I%5BAuthor%5D&cauthor=true&cauthor_uid=16861362) [Wendel-Vos W,](http://www.ncbi.nlm.nih.gov/pubmed?term=Wendel-Vos%20W%5BAuthor%5D&cauthor=true&cauthor_uid=16861362) [Giskes](http://www.ncbi.nlm.nih.gov/pubmed?term=Giskes%20K%5BAuthor%5D&cauthor=true&cauthor_uid=16861362)

[K,](http://www.ncbi.nlm.nih.gov/pubmed?term=Giskes%20K%5BAuthor%5D&cauthor=true&cauthor_uid=16861362) [van Lenthe F](http://www.ncbi.nlm.nih.gov/pubmed?term=van%20Lenthe%20F%5BAuthor%5D&cauthor=true&cauthor_uid=16861362) et al. A review of environmental correlates of

obesity-related dietary behaviors in youth[. Health Educ Res](http://www.ncbi.nlm.nih.gov/pubmed/?term=90.%09Van+der+Horst+K%2C+Oenema+A%2C+Ferreira+I%2C+et+al.+A+review+of+environmental+correlates+of+obesity-related+dietary+behaviors+in+youth.+Health+Educ+Res+2007+%3B22%3A203%E2%80%93226.) 2007

Apr;22(2):203-26.

8

8

8. Contento I, Balch G I, Bronner Y L, Lytle L A, Maloney S K, Olson C

M et al . The effectiveness of nutrition education and

implications for nutrition education policy, programs and

research: a review of research. J Nutr Educ 1995 ;27:279–418.

9. [Knai C,](http://www.ncbi.nlm.nih.gov/pubmed?term=Knai%20C%5BAuthor%5D&cauthor=true&cauthor_uid=16375956) [Pomerleau J,](http://www.ncbi.nlm.nih.gov/pubmed?term=Pomerleau%20J%5BAuthor%5D&cauthor=true&cauthor_uid=16375956) [Lock K,](http://www.ncbi.nlm.nih.gov/pubmed?term=Lock%20K%5BAuthor%5D&cauthor=true&cauthor_uid=16375956) [McKee M.](http://www.ncbi.nlm.nih.gov/pubmed?term=McKee%20M%5BAuthor%5D&cauthor=true&cauthor_uid=16375956) Getting children to eat

more fruit and vegetables: a systematic review. [Prev Med](http://www.ncbi.nlm.nih.gov/pubmed/?term=92.%09Knai+C%2C+Pomerleau+J%2C+Lock+K%2C+et+al.+Getting+children+to+eat+more+fruit+and+vegetables%3A+a+systematic+review.+Prev+Med+(2006)+42%3A85%E2%80%9395.) 2006

Feb;42(2):85-95.

1

39

9

9

0. [Ferreira I,](http://www.ncbi.nlm.nih.gov/pubmed?term=Ferreira%20I%5BAuthor%5D&cauthor=true&cauthor_uid=17300279) [van der Horst K,](http://www.ncbi.nlm.nih.gov/pubmed?term=van%20der%20Horst%20K%5BAuthor%5D&cauthor=true&cauthor_uid=17300279) [Wendel-Vos W,](http://www.ncbi.nlm.nih.gov/pubmed?term=Wendel-Vos%20W%5BAuthor%5D&cauthor=true&cauthor_uid=17300279) [Kremers S,](http://www.ncbi.nlm.nih.gov/pubmed?term=Kremers%20S%5BAuthor%5D&cauthor=true&cauthor_uid=17300279) [van Lenthe](http://www.ncbi.nlm.nih.gov/pubmed?term=van%20Lenthe%20FJ%5BAuthor%5D&cauthor=true&cauthor_uid=17300279)

[FJ,](http://www.ncbi.nlm.nih.gov/pubmed?term=van%20Lenthe%20FJ%5BAuthor%5D&cauthor=true&cauthor_uid=17300279) [Brug J.](http://www.ncbi.nlm.nih.gov/pubmed?term=Brug%20J%5BAuthor%5D&cauthor=true&cauthor_uid=17300279) Environmental correlates of physical activity in

youth—A review and updat[e. Obes Rev](http://www.ncbi.nlm.nih.gov/pubmed/?term=Environmental+correlates+of+physical+activity+in+youth%E2%80%94A+review+and+update) 2007 Mar;8(2):129-54.

1. [Evenson KR1,](http://www.ncbi.nlm.nih.gov/pubmed?term=Evenson%20KR%5BAuthor%5D&cauthor=true&cauthor_uid=16972999) [Birnbaum AS,](http://www.ncbi.nlm.nih.gov/pubmed?term=Birnbaum%20AS%5BAuthor%5D&cauthor=true&cauthor_uid=16972999) [Bedimo-Rung AL,](http://www.ncbi.nlm.nih.gov/pubmed?term=Bedimo-Rung%20AL%5BAuthor%5D&cauthor=true&cauthor_uid=16972999) [Sallis JF,](http://www.ncbi.nlm.nih.gov/pubmed?term=Sallis%20JF%5BAuthor%5D&cauthor=true&cauthor_uid=16972999) [Voorhees](http://www.ncbi.nlm.nih.gov/pubmed?term=Voorhees%20CC%5BAuthor%5D&cauthor=true&cauthor_uid=16972999)

[CC,](http://www.ncbi.nlm.nih.gov/pubmed?term=Voorhees%20CC%5BAuthor%5D&cauthor=true&cauthor_uid=16972999) [Ring K](http://www.ncbi.nlm.nih.gov/pubmed?term=Ring%20K%5BAuthor%5D&cauthor=true&cauthor_uid=16972999) et al. Girls perceptions of physical environmental

factors and transportation: reliability and association with

physical activity and active transport to schoo[l. Int J Behav Nutr](http://www.ncbi.nlm.nih.gov/pubmed/?term=Girls+perceptions+of+physical+environmental+factors+and+transportation%3A+reliability+and+association+with+physical+activity+and+active+transport+to+school.)

[Phys Act](http://www.ncbi.nlm.nih.gov/pubmed/?term=Girls+perceptions+of+physical+environmental+factors+and+transportation%3A+reliability+and+association+with+physical+activity+and+active+transport+to+school.) 2006 Sep 14;3:28.

9

2. U.S. Dept. of Health and Human Services. Reducing tobacco use:

A report of the Surgeon General. Washington, D.C.: U.S.Dept. of

Health and Human Services, Public Health Service, Centers for

Disease Control and Prevention, National Center for Chronic

Disease Prevention and Health Promotion, Office on Smoking

and Health. 2000.

9

9

3. Institute of Medicine (US) Committee on Preventing Nicotine

Addiction in Children and Youths. Growing up Tobacco Free:

Preventing Nicotine Addiction in Children and Youths. [Lynch](http://www.ncbi.nlm.nih.gov/pubmed?term=Lynch%20BS%5BEditor%5D)

[BS,](http://www.ncbi.nlm.nih.gov/pubmed?term=Lynch%20BS%5BEditor%5D) [Bonnie RJ,](http://www.ncbi.nlm.nih.gov/pubmed?term=Bonnie%20RJ%5BEditor%5D) editors.Washington (DC): National Academies

Press (US); 1994.

4. [Rasmussen M,](http://www.ncbi.nlm.nih.gov/pubmed?term=Rasmussen%20M%5BAuthor%5D&cauthor=true&cauthor_uid=16904006) [Krolner R,](http://www.ncbi.nlm.nih.gov/pubmed?term=Kr%C3%B8lner%20R%5BAuthor%5D&cauthor=true&cauthor_uid=16904006) [Klepp KI,](http://www.ncbi.nlm.nih.gov/pubmed?term=Klepp%20KI%5BAuthor%5D&cauthor=true&cauthor_uid=16904006) [Lytle L,](http://www.ncbi.nlm.nih.gov/pubmed?term=Lytle%20L%5BAuthor%5D&cauthor=true&cauthor_uid=16904006) [Brug J,](http://www.ncbi.nlm.nih.gov/pubmed?term=Brug%20J%5BAuthor%5D&cauthor=true&cauthor_uid=16904006) [Bere E](http://www.ncbi.nlm.nih.gov/pubmed?term=Bere%20E%5BAuthor%5D&cauthor=true&cauthor_uid=16904006) et al.

Determinants of fruit and vegetable consumption among

children and adolescents: systematic review of the literature.

Part I: quantitative studies. [Int J Behav Nutr Phys Act](http://www.ncbi.nlm.nih.gov/pubmed/?term=97.%09Rasmussen+M%2C+Krolner+R%2C+Klepp+K-I%2C+et+al.+Determinants+of+fruit+and+vegetable+consumption+among+children+and+adolescents%3A+systematic+review+of+the+literature.+Part+I%3A+quantitative+studies.+Int+J+Behav+Nutr+Phys+Act+2006%3B+3%3A22.) 2006 Aug

1

1;3:22.

1

40

9

9

5. Blanchette L, Brug J. Determinants of fruit and vegetable

consumption among 6-12-year-old children and effective

interventions to increase consumption. J Hum Nutr Diet

2

005;18(6):431–443.

6. [Wind M,](http://www.ncbi.nlm.nih.gov/pubmed?term=Wind%20M%5BAuthor%5D&cauthor=true&cauthor_uid=16785090) [de Bourdeaudhuij I,](http://www.ncbi.nlm.nih.gov/pubmed?term=de%20Bourdeaudhuij%20I%5BAuthor%5D&cauthor=true&cauthor_uid=16785090) [te Velde SJ,](http://www.ncbi.nlm.nih.gov/pubmed?term=te%20Velde%20SJ%5BAuthor%5D&cauthor=true&cauthor_uid=16785090) [Sandvik C,](http://www.ncbi.nlm.nih.gov/pubmed?term=Sandvik%20C%5BAuthor%5D&cauthor=true&cauthor_uid=16785090) [Due P,](http://www.ncbi.nlm.nih.gov/pubmed?term=Due%20P%5BAuthor%5D&cauthor=true&cauthor_uid=16785090) [Klepp](http://www.ncbi.nlm.nih.gov/pubmed?term=Klepp%20KI%5BAuthor%5D&cauthor=true&cauthor_uid=16785090)

[KI](http://www.ncbi.nlm.nih.gov/pubmed?term=Klepp%20KI%5BAuthor%5D&cauthor=true&cauthor_uid=16785090) et al. Correlates of fruit and vegetable consumption among 11

year-old Belgian-Flemish and Dutch schoolchildren. [J Nutr Educ](http://www.ncbi.nlm.nih.gov/pubmed/?term=.+Correlates+of+fruit+and+vegetable+consumption+among+11+year-old+Belgian-Flemish+and+Dutch+schoolchildren.)

[Behav](http://www.ncbi.nlm.nih.gov/pubmed/?term=.+Correlates+of+fruit+and+vegetable+consumption+among+11+year-old+Belgian-Flemish+and+Dutch+schoolchildren.) 2006 Jul-Aug;38(4):211-21.

9

9

9

7. Patrick H, Nicklas TA. A review of family and social determinants

of children's eating patterns and diet quality. [J Am Coll Nutr](http://www.ncbi.nlm.nih.gov/pubmed/?term=A+review+of+family+and+social+determinants+of+children%27s+eating+patterns+and+diet+quality.) 2005

Apr;24(2):83-92.

8. Sorensen G, Quintiliani LM, Pereira L, Yang M, Stoddard AM.

Work Experiences and Tobacco Use: Findings from the Gear Up

for Health Study. J Occup Environ Med 2009; 51(1):87–94.

9. Janzon E, Engstrom G, Lindstrom M, Berglund G, Hedblad B,

Janzon L. Who are the “quitters”? a cross-sectional study of

circumstances associated with women giving up smoking. Scand

J Public Health 2005; 33(3):175–182.

1

1

00. Sanderson DM, Ekholm O, Hundrup YA, Rasmussen NK. Influence

of lifestyle, health, and work environment on smoking cessation

among Danish nurses followed over 6 years. Prev Med 2005;

4

1(3-4):757–760.

01. Schulte PA, Wagner GR, Downes A, Miller DB. A framework for

the concurrent consideration of occupational hazards and

obesity. Ann Occup Hyg 2008; 52(7):555–566.

1

41

1

1

1

02. [Schulte PA,](http://www.ncbi.nlm.nih.gov/pubmed?term=Schulte%20PA%5BAuthor%5D&cauthor=true&cauthor_uid=17267711) [Wagner GR,](http://www.ncbi.nlm.nih.gov/pubmed?term=Wagner%20GR%5BAuthor%5D&cauthor=true&cauthor_uid=17267711) [Ostry A,](http://www.ncbi.nlm.nih.gov/pubmed?term=Ostry%20A%5BAuthor%5D&cauthor=true&cauthor_uid=17267711) [Blanciforti LA,](http://www.ncbi.nlm.nih.gov/pubmed?term=Blanciforti%20LA%5BAuthor%5D&cauthor=true&cauthor_uid=17267711) [Cutlip RG,](http://www.ncbi.nlm.nih.gov/pubmed?term=Cutlip%20RG%5BAuthor%5D&cauthor=true&cauthor_uid=17267711) [Krajnak](http://www.ncbi.nlm.nih.gov/pubmed?term=Krajnak%20KM%5BAuthor%5D&cauthor=true&cauthor_uid=17267711)

[KM](http://www.ncbi.nlm.nih.gov/pubmed?term=Krajnak%20KM%5BAuthor%5D&cauthor=true&cauthor_uid=17267711) et al. Work, obesity, and occupational safety and health. Am

J Public Health 2007; 97(3):428–436.

03. World HealthOrganization. Expert Committee on Health

Promotion in the Work Setting. Health promotion for working

populations. Geneva, WHO, 1988.

04. [Pelletier KR.](http://www.ncbi.nlm.nih.gov/pubmed?term=Pelletier%20KR%5BAuthor%5D&cauthor=true&cauthor_uid=11727590) A review and analysis of the clinical and cost-

effectiveness studies of comprehensive health promotion and

disease management program at the worksite: 1998–2000

updat[e. Am J Health Promot](http://www.ncbi.nlm.nih.gov/pubmed/?term=A+review+and+analysis+of+the+clinical+and+cost-effectiveness+studies+of+comprehensive+health+promotion+and+disease+management+program+at+the+worksite%3A+1998%E2%80%932000+update.) 2001 Nov-Dec;16(2):107-16.

05. Aldana, S.G. Financial impact of health promotion programs: a

1

1

1

comprehensive

review

of

the

literature.

[Am](http://www.ncbi.nlm.nih.gov/pubmed/11502012)

[J Health Promot](http://www.ncbi.nlm.nih.gov/pubmed/11502012) 2001 May-Jun;15(5):296-320.

06. Harden A, Peersman G, Oliver S, MauthnerM, OakleyA. A

systematic review of the effectiveness of health promotion

interventions in the workp[lace. Occup Med](http://www.ncbi.nlm.nih.gov/pubmed/?term=109.%09A.+Harden%2C+G.+Peersman%2C+S.+Oliver%2C+M.+Mauthner+and+A.+Oakley.+A+systematic+review+of+the+effectiveness+of+health+promotion+interventions+in+the+workplace.+Occup.+Med.+VoL+49%2C+No.+8%2C+pp.+540-548%2C+1999) 1999 Nov;49:540-8.

07. [Sorensen G,](http://www.ncbi.nlm.nih.gov/pubmed?term=Sorensen%20G%5BAuthor%5D&cauthor=true&cauthor_uid=20725775) [Stoddard A,](http://www.ncbi.nlm.nih.gov/pubmed?term=Stoddard%20A%5BAuthor%5D&cauthor=true&cauthor_uid=20725775) [Quintiliani L,](http://www.ncbi.nlm.nih.gov/pubmed?term=Quintiliani%20L%5BAuthor%5D&cauthor=true&cauthor_uid=20725775) [Ebbeling C,](http://www.ncbi.nlm.nih.gov/pubmed?term=Ebbeling%20C%5BAuthor%5D&cauthor=true&cauthor_uid=20725775) [Nagler E,](http://www.ncbi.nlm.nih.gov/pubmed?term=Nagler%20E%5BAuthor%5D&cauthor=true&cauthor_uid=20725775) [Yang](http://www.ncbi.nlm.nih.gov/pubmed?term=Yang%20M%5BAuthor%5D&cauthor=true&cauthor_uid=20725775)

[M](http://www.ncbi.nlm.nih.gov/pubmed?term=Yang%20M%5BAuthor%5D&cauthor=true&cauthor_uid=20725775) et al. Tobacco use cessation and weight management among

motor freight workers: Results of the Gear Up for Health Study.

[Cancer Causes Control](http://www.ncbi.nlm.nih.gov/pubmed/?term=110.%09Sorensen+G%2C+Stoddard+A%2C+Quintiliani+L+et+al.+Tobacco+use+cessation+and+weight+management+among+motor+freight+workers%3A+Results+of+the+Gear+Up+for+Health+Study.+Cancer+Causes+Control.+2010+December+%3B+21(12)%3A+2113%E2%80%932122.) 2010 Dec;21(12):2113-22.

1

08. [Quintiliani L,](http://www.ncbi.nlm.nih.gov/pubmed?term=Quintiliani%20L%5BAuthor%5D&cauthor=true&cauthor_uid=22617415) [Stoddard A,](http://www.ncbi.nlm.nih.gov/pubmed?term=Stoddard%20A%5BAuthor%5D&cauthor=true&cauthor_uid=22617415) [Lederman R,](http://www.ncbi.nlm.nih.gov/pubmed?term=Lederman%20R%5BAuthor%5D&cauthor=true&cauthor_uid=22617415) [Harden E,](http://www.ncbi.nlm.nih.gov/pubmed?term=Harden%20E%5BAuthor%5D&cauthor=true&cauthor_uid=22617415) [Wallace L,](http://www.ncbi.nlm.nih.gov/pubmed?term=Wallace%20L%5BAuthor%5D&cauthor=true&cauthor_uid=22617415)

[Sorensen G.](http://www.ncbi.nlm.nih.gov/pubmed?term=Sorensen%20G%5BAuthor%5D&cauthor=true&cauthor_uid=22617415) Dissemination of a tobacco cessation program for

unionized workers. [Fam Community Health](http://www.ncbi.nlm.nih.gov/pubmed/?term=Dissemination+of+a+Tobacco+Cessation+Program+for+Unionized+Workers) 2012 Jul-

Sep;35(3):246-55.

1

42

1

1

09. Sorensen G, Hunt MK, Cohen N, Stoddard A, Stein E, Phillips J et

al. [Worksite and family education for dietary change: the](http://www.ncbi.nlm.nih.gov/pubmed/10345908)

[Treatwell 5-a-Day program.](http://www.ncbi.nlm.nih.gov/pubmed/10345908) Health Educ Res 1998 Dec; 577-91.

10. Parkinson DK, Bromet EJ, Dew MA, Dunn LO, Barkman M, Wright

M. Effectiveness of the united steel workers of America coke

oven intervention program. J Occup Med 1989; 31: 464-472.

11. Levin S. Pilot study of a cafeteria program relying primarily on

symbols to promote healthy choices. J Nutr Edu 1996; 282-285.

12. [Engbers LH,](http://www.ncbi.nlm.nih.gov/pubmed?term=Engbers%20LH%5BAuthor%5D&cauthor=true&cauthor_uid=15958254) [van Poppel MN,](http://www.ncbi.nlm.nih.gov/pubmed?term=van%20Poppel%20MN%5BAuthor%5D&cauthor=true&cauthor_uid=15958254) [Chin A Paw MJ,](http://www.ncbi.nlm.nih.gov/pubmed?term=Chin%20A%20Paw%20MJ%5BAuthor%5D&cauthor=true&cauthor_uid=15958254) [van Mechelen W.](http://www.ncbi.nlm.nih.gov/pubmed?term=van%20Mechelen%20W%5BAuthor%5D&cauthor=true&cauthor_uid=15958254)

Worksite health promotion programs with environmental

changes: a systematic review[. Am J Prev Med](http://www.ncbi.nlm.nih.gov/pubmed/15958254) 2005;29(1): 61-70.

13. Moskowitz JM, Lin Z, Hudes ES. The impact of workplace smoking

ordinances in California on smoking cessation. [Am J Public](http://www.ncbi.nlm.nih.gov/pubmed/?term=The+impact+of+workplace+smoking+ordinances+in+California+on+smoking+cessation.)

[Health](http://www.ncbi.nlm.nih.gov/pubmed/?term=The+impact+of+workplace+smoking+ordinances+in+California+on+smoking+cessation.) 2000 May;90(5):757-61.

1

1

1

1

14. [Hopkins DP,](http://www.ncbi.nlm.nih.gov/pubmed?term=Hopkins%20DP%5BAuthor%5D&cauthor=true&cauthor_uid=11173215) [Briss PA,](http://www.ncbi.nlm.nih.gov/pubmed?term=Briss%20PA%5BAuthor%5D&cauthor=true&cauthor_uid=11173215) [Ricard CJ,](http://www.ncbi.nlm.nih.gov/pubmed?term=Ricard%20CJ%5BAuthor%5D&cauthor=true&cauthor_uid=11173215) [Husten CG,](http://www.ncbi.nlm.nih.gov/pubmed?term=Husten%20CG%5BAuthor%5D&cauthor=true&cauthor_uid=11173215) [Carande-Kulis](http://www.ncbi.nlm.nih.gov/pubmed?term=Carande-Kulis%20VG%5BAuthor%5D&cauthor=true&cauthor_uid=11173215)

[VG,](http://www.ncbi.nlm.nih.gov/pubmed?term=Carande-Kulis%20VG%5BAuthor%5D&cauthor=true&cauthor_uid=11173215) [Fielding JE](http://www.ncbi.nlm.nih.gov/pubmed?term=Fielding%20JE%5BAuthor%5D&cauthor=true&cauthor_uid=11173215) et al. The Task Force on Community Preventive

Services. Reviews of evidence regarding interventions to reduce

tobacco use and exposure to environmental tobacco smok[e. Am](http://www.ncbi.nlm.nih.gov/pubmed/11173215)

[J Prev Med](http://www.ncbi.nlm.nih.gov/pubmed/11173215) 2001 Feb;20(2 Suppl):16-66.

1

1

15. Fichtenberg CM, Glantz SA. Effect of smoke-free workplaces on

smoking behaviour: systematic review. [BMJ](http://www.ncbi.nlm.nih.gov/pubmed/12142305) 2002 Jul

2

7;325(7357):188.

16. Borland R, Pierce JP, Burns DM, Gilpin E, Johnson M, Bal D.

Protection from environmental tobacco smoke in California: the

case for a smoke-free workplace. JAMA 1992;268(6):749–52.

1

43

1

1

17. Moskowitz JM, Lin Z, Hudes ES. The impact of California’s

smoking ordinances on worksite smoking policy and exposure to

environmental tobacco smoke. Am

J

Health Promot

1

999;13(5):278–81.

18. Groeneveld IF, Proper KI, van der Beek AJ, Hildebrandt VH, van

Mechelen W. Lifestyle-focused interventions at the workplace to

reduce the risk of cardiovascular disease – a systematic review.

Scand J Work Environ Health 2010;36(3):202-215

1

1

19. Wilson MG, Holman PB, Hammock A. A comprehensive review of

the effects of worksite health promotion on health related

outcomes. Am J Health Prom 1996; 10: 429-435.

20. Faculty of Public Health Medicine Committee on Health

Promotion. Health Promotion in the Workplace. Guidelines for

Health Promotion No. 40. London, UK: Royal College of

Physicians, 1995.

1

1

21. Rose G. Strategy of prevention: Lessons from cardiovascular

disease. BMJ 1981;282:1847-51.

22. Health and Welfare Canada. Heart health equality: mobilizing

communities for action. Ottawa, Ontario: Supply and Services

Canada, Cat, 1992. no H39-245/1992E.

1

1

23. Rose G, Day S. The population mean predicts the number of

deviant individuals. BMJ 1990;301:1031-4.

24. Puska P. Successful prevention of non-communicable diseases:

2

5 year experiences with North Karelia Project in Finland. Public

Health Medicine 2002; 4(1):5-7

1

44

1

1

25. Puska P. Health in all policies. [Eur J Public Health](http://www.ncbi.nlm.nih.gov/pubmed/?term=129.%09Puska+P.+Health+in+all+policies.+European+Journal+of+Public+Health+2007%3B+Vol.+17%2C+No.+4%2C+328.) 2007

Aug;17(4):328.

26. Sarrafzadegan N, Kelishadi R, Sadri G, Malekafzali H,

Pourmoghaddas M, Heidari

K

et al. Outcomes of

a

comprehensive healthy lifestyle program on cardiometabolic risk

factors in a developing country: the Isfahan Healthy Heart

Pro[gram. Arch Iran Med](http://www.ncbi.nlm.nih.gov/pubmed/?term=Outcomes+of+a+Comprehensive+Healthy+Lifestyle+Program+on+Cardiometabolic+Risk+Factors+in+a+Developing+Country%3A+The+Isfahan+Healthy+Heart+Program) 2013 Jan;16(1):4-11.

1

1

1

27. [Sarrafzadegan N,](http://www.ncbi.nlm.nih.gov/pubmed?term=Sarrafzadegan%20N%5BAuthor%5D&cauthor=true&cauthor_uid=19197403) [Kelishadi R,](http://www.ncbi.nlm.nih.gov/pubmed?term=Kelishadi%20R%5BAuthor%5D&cauthor=true&cauthor_uid=19197403) [Esmaillzadeh A,](http://www.ncbi.nlm.nih.gov/pubmed?term=Esmaillzadeh%20A%5BAuthor%5D&cauthor=true&cauthor_uid=19197403) [Mohammadifard](http://www.ncbi.nlm.nih.gov/pubmed?term=Mohammadifard%20N%5BAuthor%5D&cauthor=true&cauthor_uid=19197403)

[N,](http://www.ncbi.nlm.nih.gov/pubmed?term=Mohammadifard%20N%5BAuthor%5D&cauthor=true&cauthor_uid=19197403) [Rabiei K,](http://www.ncbi.nlm.nih.gov/pubmed?term=Rabiei%20K%5BAuthor%5D&cauthor=true&cauthor_uid=19197403) [Roohafza H](http://www.ncbi.nlm.nih.gov/pubmed?term=Roohafza%20H%5BAuthor%5D&cauthor=true&cauthor_uid=19197403) et al. Do lifestyle interventions work in

developing countries? Findings from the Isfahan Healthy Heart

Program in the Islamic Republic of Iran. [Bull World Health](http://www.ncbi.nlm.nih.gov/pubmed/?term=Do+lifestyle+interventions+work+in+developing+countries%3F+Findings+from+the+Isfahan+Healthy+Heart+Program+in+the+Islamic+Republic+of+Iran)

[Organ](http://www.ncbi.nlm.nih.gov/pubmed/?term=Do+lifestyle+interventions+work+in+developing+countries%3F+Findings+from+the+Isfahan+Healthy+Heart+Program+in+the+Islamic+Republic+of+Iran) 2009 Jan;87(1):39-50.

28. [Rabiei K,](http://www.ncbi.nlm.nih.gov/pubmed?term=Rabiei%20K%5BAuthor%5D&cauthor=true&cauthor_uid=19216762) [Kelishadi R,](http://www.ncbi.nlm.nih.gov/pubmed?term=Kelishadi%20R%5BAuthor%5D&cauthor=true&cauthor_uid=19216762) [Sarrafzadegan N,](http://www.ncbi.nlm.nih.gov/pubmed?term=Sarrafzadegan%20N%5BAuthor%5D&cauthor=true&cauthor_uid=19216762) [Abedi HA,](http://www.ncbi.nlm.nih.gov/pubmed?term=Abedi%20HA%5BAuthor%5D&cauthor=true&cauthor_uid=19216762) [Alavi M,](http://www.ncbi.nlm.nih.gov/pubmed?term=Alavi%20M%5BAuthor%5D&cauthor=true&cauthor_uid=19216762) [Heidari](http://www.ncbi.nlm.nih.gov/pubmed?term=Heidari%20K%5BAuthor%5D&cauthor=true&cauthor_uid=19216762)

[K](http://www.ncbi.nlm.nih.gov/pubmed?term=Heidari%20K%5BAuthor%5D&cauthor=true&cauthor_uid=19216762) et al. Process evaluation of a community-based program for

prevention and control of communicable disease in a developing

country: The Isfahan Healthy Heart Program. [BMC Public](http://www.ncbi.nlm.nih.gov/pubmed/?term=Process+evaluation+of+a+community-based+program+for+prevention+and+control+of+communicable+disease+in+a+developing+country%3A+The+Isfahan+Healthy+Heart+Program.)

[Health](http://www.ncbi.nlm.nih.gov/pubmed/?term=Process+evaluation+of+a+community-based+program+for+prevention+and+control+of+communicable+disease+in+a+developing+country%3A+The+Isfahan+Healthy+Heart+Program.) 2009 Feb 12;9:57.

29. Sarrafzadegan N, Rabiei K, Alavi M, Abedi HA, Zarfeshani S. How

can the results of a qualitative process evaluation be applied in

management, improvement and modification of a preventive

community trial? The IHHP Study. [Arch Public Health](http://www.ncbi.nlm.nih.gov/pubmed/?term=133.%09Sarrafzadegan+N%2C+Rabiei+K%2C+Alavi+M%2C+Abedi+HA%2C+Zarfeshani+S.+How+can+the+results+of+a+qualitative+process+evaluation+be+applied+in+management%2C+improvement+and+modi%F4%80%82%BFcation+of+a+preventive+community+trial%3F+The+IHHP+Study.+Arch+Pub+Health.+2011%3B+69(9).) 2011 Dec

5

;69(1):9.

1

45

1

1

1

30. [Robinson K,](http://www.ncbi.nlm.nih.gov/pubmed?term=Robinson%20K%5BAuthor%5D&cauthor=true&cauthor_uid=15613492) [Elliott SJ,](http://www.ncbi.nlm.nih.gov/pubmed?term=Elliott%20SJ%5BAuthor%5D&cauthor=true&cauthor_uid=15613492) [Driedger SM.](http://www.ncbi.nlm.nih.gov/pubmed?term=Driedger%20SM%5BAuthor%5D&cauthor=true&cauthor_uid=15613492) Using linking systems to build

capacity and enhance dissemination in heart health promotion: a

Canadian multiple-case study. [Health Educ Res](http://www.ncbi.nlm.nih.gov/pubmed/?term=Using+linking+systems+to+build+capacity+and+enhance+dissemination+in+heart+health+promotion%3A+a+Canadian+multiple-case+study) 2005

Oct;20(5):499-513.

31. Potvin L, Paradis G, Lessard R. le paradoxe de l’évaluation des

programmes communautaires multiples de promotion de la

santé. Ruptures, revue transdisciplinaire en santé 1994 ;1 (1) :

4

5-57.

32. [Nissinen A,](http://www.ncbi.nlm.nih.gov/pubmed?term=Nissinen%20A%5BAuthor%5D&cauthor=true&cauthor_uid=11693979) [Berrios X,](http://www.ncbi.nlm.nih.gov/pubmed?term=Berrios%20X%5BAuthor%5D&cauthor=true&cauthor_uid=11693979) [Puska P.](http://www.ncbi.nlm.nih.gov/pubmed?term=Puska%20P%5BAuthor%5D&cauthor=true&cauthor_uid=11693979) Community-based non

communicable disease interventions: lessons from developed

countries for developing ones. [Bull World Health Organ](http://www.ncbi.nlm.nih.gov/pubmed/?term=Community-based+noncommunicable+disease+interventions%3A+lessons+from+developed+countries+for+developing+ones)

2

001;79(10): 963-70.

1

1

1

1

33. World Health Organization. Scaling up action against non

communicable diseases: How much will it cost?. Geneva, 2011.

34. World Health Organization. Global strategy on diet, physical

activity and health. Geneva, 2004.

35. World Health Organization. Global strategy to reduce the

harmful use of alcohol. Geneva, 2010.

36. World Economic Forum. From Burden to “Best Buys”: Reducing

the Economic Impact of Non-Communicable Diseases in Low-

and Middle-Income Countries. Geneva, Switzerland; 2011

37. World Health Organization. Global action plan for the prevention

and control of non communicable diseases 2013-2020. Geneva,

WHO, 2013.

1

1

46

**7**

**. Acknowledgement**

We would like to thank all the staff of the Department of Epidemiology, the General Direction of

the University Hospital Farhat Hached of Sousse and all our partners:

1.

At the National level:







Departments of Cardiology, Endocrinology, Occupational Health, Pediatrics of the

University Hospital Farhat Hached of Sousse and the Department of Pediatrics of

the University Hospital Sahloul & the Department of Biochemistry of the Faculty

of Medicine of Sousse.

Regional Direction of Health and Regional Direction of Education of Sousse, the

Regional Commissioner for Youth, Sports and Physical Education of Sousse, the

Regional Service of Children of Sousse and the Group of Occupational Medicine of

Sousse

Associations: Tunisian Heart Foundation (THF) and the Association of Professors

of life science and earth.

2.

At the International level:









The National Institute of Health and Welfare (THL) of Helsinki, Finland

Duluth Medical Research Institute, University of Minnesota, USA

Matrix Public Solutions

The Department of Community Medicine, Primary Care and Emergency,

University Hospitals of Geneva



WHO office in Tunis

The project «Together in Health » was funded by ‘UnitedHealth Group’

and by the Research Unit ‘Santé UR12SP28’: Epidemiologic transition and prevention of chronic

disease of the Ministry of Higher Education (Tunisia)

1

47
