# Supplementary material for: Intervention effectiveness in reducing the clustering of non-communicable disease risk factors in the workplace: A quasi-experimental study
Source: PLoS One. 2025 Feb 6;20(2):e0317460. doi: 10.1371/journal.pone.0317460 (PMC11801702; doi:10.1371/journal.pone.0317460)
Supplement: Attachment — Submitted filename: Manuscript dds.docx [file pone.0317460.s004.docx]

**Full title: Intervention effectiveness in reducing the clustering of non-communicable disease risk factors in workplace: a quasi-experimental study**

**Short title: Prevention of chronic disease risk factors**

Nahla Guesmi^1^, Sihem Ben Fredj^1,2^, Nawel Zammit^1,2^, Rim Ghammam^1,2^, Imed Harrabi^1,2^, Firas Chouikha^1^, Maher Maoua^2,3^, Jihen Maatoug^1,2^, Hassen Ghannem^1,2^

^1^ Department of Epidemiology, «LR19SP03», University Hospital Farhat Hached, Sousse, Tunisia.

^2^ Faculty of Medicine of Sousse, University of Sousse, Tunisia.

^3^ Department of Occupational Medicine, «LR19SP03», University Hospital Farhat Hached, Sousse, Tunisia.

***Corresponding author:** Nahla Guesmi

**E-mail:** [nahlagasmi@gmail.com](mailto:nahlagasmi@gmail.com) (NG)

**Abstract**

Understanding the clustering patterns of non-communicable disease risk factors is important to address chronic diseases effectively, thus minimizing their onset and enhancing overall health. This study aimed to assess the feasibility and efficacy of a three-year workplace intervention in decreasing clustering of non-communicable disease risk factors in employees. A quasi-experimental study, including six companies, was conducted in the governorate of Sousse between 2010 and 2014. It involved an intervention group (Sousse-Jawhara and Sousse-Erriadh) and a control group (M’saken). The sample of participants in both groups was representative. The intervention included actions promoting physical activity, healthy diet, and smoking cessation in the workplace. A pre-tested questionnaire collected the participants’ socio-demographic characteristics and data concerning the risk factors through interviews. Clustering of tobacco use, physical inactivity, unhealthy diet, obesity, and high blood pressure was examined pre- and post-intervention. The mean risk factors per employee in the intervention group decreased significantly from 1.99±1.00 to 1.81±1.05 (p<10-3). A minor non-significant increase, from 1.72±0.97 to 1.78±1.11, was noted in the control group. The prevalence of two risk factor clusters dropped significantly from 40% to 34.4% pre- and post-intervention in the intervention group (p=0.014). However, an insignificant decline was noted in the control group. Combinations like obesity/hypertension and unhealthy diet/physical inactivity tended to aggregate in both groups. Overall, the intervention program demonstrated significant protective effects in reducing the co-occurrence of multiple risk factors in the intervention group, with an adjusted OR of 0.81; CI95% [0.68 – 0.97]. Along with the existing literature, the present study confirms the feasibility and effectiveness of health promotion programs in reducing non-communicable disease risk factors and their clustering. Integrating this intervention program into a national health policy could potentially generalize its positive impact.

**Introduction:**

Non-communicable diseases (NCDs) are a field of public health involving a very broad range of health issue categories, cardiovascular diseases, cancer, mental disorders, diabetes etc. The prevalence of NCDs, previously called “diseases of affluence”, has sharply risen worldwide in the last decades. This rise is ascribable to the economic progress, urban development, industrialism, and globalization, leading to changes in lifestyle worldwide and nationwide. [1] NCDs have a major impact on individual lives as well as public health costs. In terms of individual lives, they can lead to disability, which in turn affects the quality of life and the ability to work. They can also lead to premature death. With regard to public health costs, they represent a major burden on healthcare systems as they often require ongoing care and treatment, accounting for a large proportion of global healthcare spending [2]. It is crucial that public health initiatives focus on the prevention and management of NCDs to improve health outcomes and alleviate their economic impact, which is considered a challenging task mainly in low- and middle-income Countries (LMICs) where health systems are marked by fragmented services [3]. The risk of premature death by NCDs is actually the highest in LMICs [4]. In Tunisia, NCDs are responsible for 86% of deaths in 2016 (49% of deaths are caused by cardiovascular diseases) and 16% of premature deaths in the age category 30-70 years in the same year. Overall, five main risk factors can be ~~incriminated~~, including tobacco and alcohol consumption, physical inactivity, unhealthy diet, hypertension, and obesity [5]. The situation in Tunisia is alarming. The World Health Organization (WHO) has reported Tunisia as the first Arab country in using tobacco among men since one out of two men is a smoker [6]. In 2001, a study was conducted by the Tunisian National Institute of Public Health on a representative sample of the Tunisian population, involving 8576 subjects aged 15 years or more revealed that the prevalence of overweight is 12.2% (6.1% in males and 18.3% in females p<10-2) [7]. A key approach to address NCDs, especially in LMICs with limited clinical capacity, is to prevent them through policies that reduce unhealthy behaviors and the risk factors [8]. Yet, many policymakers are unsure about where to focus their efforts. These risk factors can often co-occur [9,10]. There is evidence that the risk factors have synergistic effects and that their combination is more harmful to health than their cumulative individual effects [11,12]. Consequently, the WHO has recently recommended an approach to chronic disease prevention focusing on how to address multiple modifiable risk factors [13]. Furthermore, the workplace has been proposed by the WHO as a priority environment for health promotion in the 21^st^ century: The workplace directly affects the workers’ physical, psychological, economic, and social well-being, which in turn affect the health of their families, communities, and societies. It provides an ideal framework and infrastructure to support health promotion for a broad audience [14]. Given the lack of interventions investigating the ecological approaches to prevention programs of NCDs in the workplace in LMICs, Sousse Chronic Disease Prevention Research Center (CDPRC) initiated a large-scale quasi-experimental workplace study in 2009 as community-based research being a part of non-communicable disease (NCD) prevention programs. The aim of the present study was to investigate the effectiveness of early intervention on the NCDs risk factor cluster and to examine predictors of the cumulative NCD risk factors in a Tunisian workforce.

**Methods**

**Study design and sample**

A quasi-experimental study involving two groups was carried out in ~~the governorate~~ of Sousse between 2010 and 2014. It was performed within the framework of a community intervention program aiming at the prevention of the major risk factors for non-communicable diseases, known as the Common Health Project. The project was launched in 2009 by the Chronic Disease Prevention Research Center (CDPRC) in Sousse in collaboration with other departments at Farhat Hached University Hospital. A total of three companies (Epi d'Or, Texmed, and UATS) in Sousse were randomly selected to be part of the intervention group, and three others constituted the control group. The latter included (STIP, Texmed, UATS, AAF, and Fitlec) in M'saken and they were relatively similar to the intervention group in size and gender composition. They involved at least 1 000 employees per group. All the employees in the chosen companies were enrolled in data acquisition. The pre- and post-intervention assessments were conducted on the two independent samples in both groups. The intervention group included companies in Sousse Jawhara and Sousse Erriadh. The control group included companies in M'saken. The estimated distance between Sousse Riadh-Jawhara and M'saken is about 20 km. The distance was taken into account to avoid contamination bias.

**Data collection and measurements**

Pre-assessment was conducted before the 2009-2010 intervention. It involved the participants' attitudes and behaviors with respect to the different lifestyle risk factors studied, including the diet, physical activity, smoking, as well as the biological risk factors (obesity and hypertension) in the two groups. Reassessment of these parameters took place at the end of the intervention in both groups in 2013-2014. Standardized pretests and questionnaires translated into Arabic were used. They were developed for the Community Health Intervention Project in collaboration with the Department of Epidemiology at Farhat Hached University Hospital. This questionnaire was distributed among the participants, allowing us to collect information on the socio-demographic characteristics, occupational characteristics, dietary habits, physical activity habits, and smoking habits. Biometric data, such as height and weight were also collected. A portable electronic scale was used to measure weight to the nearest 0.1 kg. The participants’ height was measured standing and barefoot, with an accuracy of 0.5 cm. Blood pressure at rest was measured twice using an electronic arm sphygmomanometer. The same questionnaire was distributed at the pre- and post-intervention assessments. The participants were interviewed by doctors trained at the site.

**Intervention**

The intervention strategies are based on employee engagement [15] and a social contextual framework for multi-level behavioral influence [16]. Focus was therefore put on three main points. The first involves effective communication to motivate managers and other stakeholders to participate in promoting healthy lifestyles in the workplace [17]. The second is based on creating a work environment that supports healthy eating, physical activity, and tobacco control. The third point consists in raising workers’ awareness of healthy lifestyles and risk factors for cardiovascular diseases. Therefore, a workplace intervention program was started in September 2010 and it lasted three years. The purpose of this intervention was to promote healthy diet and physical activity, and to encourage smoking cessation. To emphasize the importance of the intervention, training workshops for doctors, managers, and staff were organized in the Department of Occupational Medicine at Farhat Hached University Hospital and the Occupational Medicine Group in Sousse. The intervention strategy involved training of occupational health professionals and standardization of educational information to promote healthy lifestyles. Open awareness days, educational sessions, workshops, screenings of educational films, and interactive discussions were organized. Posters, streamers, and flyers in the cafeteria were also provided. In order to promote smoking cessation, smoking was prohibited in the company and smoking rooms were provided for smokers. In addition, free smoking cessation counseling was offered within the company. To promote healthy eating, healthy food was provided in the company cafeterias after evaluating the needs of the kitchen staff and the characteristics of the customers’ consumption. With regard to physical activities, free exercise activities were organized for employees and alternative transportation was encouraged (walking, riding a bike).

**Variables definitions**

The recommended level of physical activity was used as defined by the WHO. Physical inactivity is defined when adults aged 18–64 do less than 150 minutes of moderate intensity aerobic physical activity throughout the week or they do less than 75 minutes of vigorous intensity aerobic physical activity throughout the week or an equivalent combination of both moderate and vigorous intensity activities. An unhealthy dietary behavior was defined as a diet containing less than five servings of fruits and vegetables per day. With regard to the definition of smokers, the participants were asked the following question, “Do you currently smoke any tobacco products, such as cigarettes, cigars, or pipes?” Those who responded “yes” were defined as smokers. To define overweight and obesity, Body Mass Index (BMI) in kg/m2 was calculated by the ratio of weight to the square of the height and the international BMI cutoff values were used according to the WHO. Blood pressure at rest was measured twice by medical doctors using a digital monitor. The mean value of the 2 measurements was used to calculate systolic and diastolic blood pressure and to define high blood pressure on the day of the measurement. Adults were considered to have hypertension if they had systolic blood pressure of 140 mm Hg or more and/or diastolic blood pressure of 90 mm Hg or more on the day of the visit [21].

**Data analyses**

Statistical analysis was performed using the SPSS 20.0 software for windows. Continuous variable was presented as Mean (standard deviations) and categorical ones as percentages (%). Chi square test was used to compare percentages and Student t test to compare means in independent groups. The level of statistical significance was set at p <0.05.

The baseline characteristics of the study population were evaluated for the intervention group and the control group separately in pre- and post-assessment. Prevalence of lifestyle risk factors was investigated in the study population. The observed frequency of single risk factors coded as a binary variable were described (presence = 1; absence = 0). The prevalence of multiple risk factors was estimated from the sum of risk factors, being ranked from 0 to 5 (0 = no risk factor, 5 = all risk factors), based on the distribution observed in the sample at pre- and post-assessment in both groups. First, the clustering of the five lifestyle risk factors was examined. Clustering of two or more risk factors was studied on the basis of ratios of the observed and expected prevalence of one, two, three, four, and five simultaneously occurring risk habits. The expected proportion was calculated by multiplying the individual probabilities of each risk factor based on its occurrence in the study population for the intervention group and the control group separately in pre-and post-assessment. The difference between the observed and the expected proportion (O/E) was calculated. Aggregation exists when the observed combination (O) exceeded the expected prevalence factors (E) of the combination. If the result of the ratio of observed-to-expected (O/E) was greater than 1, it indicated the existence of aggregation between the risk factors. Secondly, the prevalence odds ratio (POR) was used to calculate clustering of two risk factors, regardless of the exposure to the other three risk factors. The POR represents the estimation of the relative odds of a risk factor in relation to another risk factor, which is calculated using the following equation:

$$\frac{\boldsymbol{N}_{\mathbf{11}}\times\boldsymbol{N}_{\mathbf{00}}}{\boldsymbol{N}_{\mathbf{10}} \times\boldsymbol{N}_{\mathbf{01}}}$$

Where $\boldsymbol{N}_{\mathbf{11}}$ is the number of individuals exposed to two risk factors, $\boldsymbol{N}_{\mathbf{00}}$ is the number of individuals without any risk factor, $\boldsymbol{N}_{\mathbf{10}}$ is the number of respondents showing only one risk factor, and $\boldsymbol{N}_{\mathbf{01}}$ representing those showing the other risk factors. For instance, a POR of 1.5 indicates that subjects exhibiting one risk factor (e.g., physical inactivity) are 1.5 times more likely to show the other risk factor (e.g., unhealthy diet) when compared to the subjects not exposed to the risk factor (e.g., physical inactivity). If 1 is not included in the 95% confidence interval, the two risk factors cluster. We evaluated if clustering of risk factors was differential between the intervention group and the control group in pre-and post-assessment. The final analysis was multivariate. For the selection of explanatory variables, univariate analysis was applied to examine the association between each independent variable and clustered risk factors. Then, Stepwise multinomial logistic regression analysis was used in order to seek the independent effects of the variables showing significant univariate associations or with p-value ≤ 0.2. These variables were included as potential confounding variables. The adjusted odds ratios (OR) and 95% confidence intervals were calculated. The three-level variable, i.e., clusters of risk factors, was set as the dependent variable in the regression model. The model estimated the probability that a respondent had a certain number of lifestyle risk factors compared to the reference group having no or one risk factor, with p < 0.05 being adopted for all analyses as the threshold for statistical significance. All analyses were conducted for the intervention group and the control group separately at pre-and post-assessment.

**Ethical considerations**

The protocol, data collection forms, and the questionnaire were approved by the ethics committee at Farhat Hached University Hospital, Sousse. Authorizations from the Ministry of Health, the governor of Sousse, and the Occupational Medicine Group in Sousse were obtained. Informed consent was obtained from the participants before the beginning of the intervention program. The latter consisted of interactive education actions having no damage on the participants’ integrity. At the end of the program and after the completion of post-intervention assessment, the same health education program was started for the control group as a delayed intervention

**Results**

**Characteristics of the study population**

Before the start of the intervention program, a total of 1775 employees were included in the study, involving 914 in the intervention group and 861 in the control group, with an overall response rate of 74.6%. After the intervention, a total of 2113 employees participated in the assessment, involving 1098 in the intervention group and 1015 in the control group, with a global response rate reaching 71.9%. The sociodemographic characteristics are presented in table 1.

**Table 1: The socio-demographic characteristics of the employees in the intervention and control groups pre- and post-intervention (Sousse, Tunisia. 2009-2014).**

| **Characteristics** | **Intervention group** | | | **Control group** | | |
| --- | --- | --- | --- | --- | --- | --- |
|  | Pre | Post | **p** | Pre | Post | **p** |
| **Age** means (SD) | 32.25 (8.11) | 33.86 (8.10) | **<10^-3^** | 35.40 (8.79) | 38.90 (8.77) | **<10^-3^** |
| **Age group** n (%) |  |  |  |  |  |  |
| **[12-29]** | 384 (42.5) | 373 (34.0) | **0.001** | 242 (28.7) | 158 (15.6) | **<10^-3^** |
| **[30-39]** | 357 (39.5) | 489 (44.5) |  | 284 (33.7) | 387 (38.1) |  |
| **[40-49]** | 117 (13.0) | 178 (16.2) |  | 283 (33.6) | 339 (33.4) |  |
| **[50-67]** | 45 (5.0) | 58 (5.3) |  | 33 (3.9) | 131 (12.9) |  |
| **Gender** n (%) |  |  |  |  |  |  |
| Male | 591 (64.7) | 719 (65.5) | 0.70 | 508(59.0) | 623 (61.4) | 0.29 |
| Female | 323 (35.3) | 379 (34.5) |  | 353 (41.0) | 392 (38.6) |  |
| **Marital status** n (%) |  |  |  |  |  |  |
| Single | 438 (48.5) | 387 (35.3) | **<10^-3^** | 238 (33.5) | 252 (25.1) | **<10^-3^** |
| Divorced or widower | 18 (2.0) | 11 (1.0) |  | 9 (1.1) | 16 (1.6) |  |
| Married | 447 (49.5) | 698 (63.7) |  | 553 (65.4) | 741 (73.4) |  |
| **Educational level** n (%) |  |  |  |  |  |  |
| Illiterate or Primary | 290 (32.2) | 316 (28.8) | 0.37 | 100 (12.0) | 144 (14.2) | 0.13 |
| College or Secondary | 447 (49.6) | 592 (54.0) |  | 646 (77.6) | 764 (75.3) |  |
| University | 165 (18.2) | 189 (17.2) |  | 87 (10.4) | 106 (10.5) |  |
| **Occupation** n (%) |  |  |  |  |  |  |
| Blue collar | 669 (74.6) | 818 (75.3) | 0.69 | 698 (84.9) | 760 (80.6) | **0.016** |
| White collar | 228 (25.4) | 269 (24.7) |  | 124 (15.1) | 183 (19.4) |  |

Table 2 shows the observed and expected prevalence for all the 32 possible combinations of the five chronic disease risk factors. At baseline, in the intervention group, the expected prevalence of the five risk factors was 0.4%; however, the observed prevalence of the five risk factors was 0.7% at pre-assessment, representing a 75% increase from expectation (O/E = 1.75). This ratio increased after the assessment in both the intervention and control groups. With regard to the simultaneity of the four risk factors, the combination of “lack of exercise, unhealthy diet, obesity, hypertension” was relevant and it was 300% higher than expected for independent factors (O/E = 3) in the intervention group. However, it slightly fell in hindsight. As for the simultaneous occurrence of three risk factors, the most noteworthy was the combinations “Physical inactivity + obesity + high blood pressure” (O/E = 2.17) and “smoking + obesity + high blood pressure” (O/E = 2). The first cluster rose to 3.5 after the intervention while the second fell to 1.66. In the control group, the two clusters increased. In the aggregation of two risk factors, obesity and hypertension were the highest in the intervention and control groups (O/E = 2), and they both decreased in the intervention and control groups after assessment.

**Table 2: Observed and expected prevalence of NCDs risk factors and their cluster at pre-and post-assessment in the intervention and control groups among employees, Tunisia (2009-2014)**

|  | **S** | **PI** | **UD** | **O** | **HBP** | **Intervention group** | | | | | | **Control group** | | | | | |
| --- | --- | --- | --- | --- | --- | --- | --- | --- | --- | --- | --- | --- | --- | --- | --- | --- | --- |
|  |  |  |  |  |  | **Pre** | | | **Post** | | | **Pre** | | | **Post** | | |
|  |  |  |  |  |  | **O (%)** | **E (%)** | **O/E** | **O (%)** | **E (%)** | **O/E** | **O (%)** | **E (%)** | **O/E** | **O (%)** | **E (%)** | **O/E** |
| **0** | - | - | - | - | - | 5.9 | 5.6 | 1.05 | 10.3 | 8.4 | 1.27 | 9.5 | 8.8 | 1.07 | 12.5 | 9.9 | 1.26 |
| **1** | + | - | - | - | - | 4.7 | 3.7 | 1.27 | 8.7 | 5.0 | 1.74 | 5.4 | 4.1 | 1.31 | 5.9 | 4.4 | 1.34 |
|  | - | + | - | - | - | 14.0 | 14.4 | 0.97 | 11.2 | 13.7 | 0.82 | 16.5 | 19.5 | 0.84 | 11.9 | 13.2 | 0.90 |
|  | - | - | + | - | - | 4.3 | 6.2 | 0.69 | 4.8 | 7.7 | 0.62 | 5.9 | 5.7 | 1.03 | 4.4 | 6.0 | 0.73 |
|  | - | - | - | + | - | 0.8 | 1.0 | 0.80 | 2.9 | 2.1 | 1.38 | 2.8 | 2.1 | 1.33 | 3.8 | 3.7 | 1.03 |
|  | - | - | - | - | + | 0.9 | 1.2 | 0.75 | 1.4 | 1.4 | 1.00 | 1.4 | 1.6 | 0.87 | 2.7 | 3.5 | 0.77 |
| **2** | + | + | - | - | - | 8.6 | 9.3 | 0.92 | 6.6 | 8.2 | 0.80 | 10.5 | 9.0 | 1.16 | 4.7 | 5.8 | 0.81 |
|  | + | - | + | - | - | 5.1 | 4.0 | 1.27 | 3.8 | 4.6 | 0.82 | 0.9 | 2.6 | 0.35 | 2.1 | 2.7 | 0.78 |
|  | + | - | - | + | - | 0.3 | 0.6 | 0.50 | 0.2 | 1.3 | 0.15 | 0.8 | 1.0 | 0.80 | 1.2 | 1.6 | 0.75 |
|  | + | - | - | - | + | 0.9 | 0.8 | 1.12 | 0.7 | 0.8 | 0.87 | 0.7 | 0.7 | 1.00 | 2.5 | 1.5 | 1.67 |
|  | - | + | + | - | - | 17.0 | 16.0 | 1.06 | 14.9 | 12.7 | 1.17 | 16.1 | 5.7 | 2.82 | 10.7 | 8.0 | 1.34 |
|  | - | + | - | + | - | 3.0 | 2.5 | 1.20 | 4.0 | 3.6 | 1.11 | 3.2 | 4.8 | 0.67 | 4.8 | 5.0 | 0.96 |
|  | - | + | - | - | + | 2.6 | 3.1 | 0.83 | 1.5 | 2.3 | 0.65 | 2.3 | 3.4 | 0.68 | 2.1 | 4.6 | 0.45 |
|  | - | - | + | + | - | 0.3 | 1.1 | 0.27 | 0.7 | 2.0 | 0.35 | 1.1 | 1.4 | 0.78 | 1.6 | 2.3 | 0.69 |
|  | - | - | + | - | + | 1.4 | 1.4 | 1.00 | 1.0 | 1.3 | 0.77 | 0.7 | 1.0 | 0.70 | 1.3 | 2.1 | 0.62 |
|  | - | - | - | + | + | 0.4 | 0.2 | 2.00 | 0.6 | 0.4 | 1.50 | 0.8 | 0.4 | 2.00 | 1.9 | 1.3 | 1.46 |
| **3** | + | + | + | - | - | 11.5 | 10.2 | 1.13 | 10.2 | 7.6 | 1.34 | 4.7 | 5.8 | 2.00 | 4 | 3.5 | 1.14 |
|  | - | + | + | + | - | 2.6 | 2.8 | 0.93 | 3.9 | 3.3 | 1.18 | 3.3 | 3.0 | 1.10 | 3.3 | 3.0 | 1.10 |
|  | - | - | + | + | + | 0.3 | 0.2 | 1.50 | 0.4 | 0.3 | 1.33 | 0.4 | 0.2 | 0.20 | 0.3 | 0.8 | 0.37 |
|  | + | - | + | - | + | 0.6 | 0.9 | 0.67 | 0.4 | 0.8 | 0.50 | 0.4 | 0.5 | 0.80 | 1.0 | 0.9 | 1.11 |
|  | + | - | - | + | + | 0.2 | 0.1 | 2.00 | 0.7 | 0.2 | 3.50 | 0.1 | 0.2 | 0.50 | 1.0 | 0.5 | 2.00 |
|  | - | + | - | + | + | 1.3 | 0.6 | 2.17 | 1.0 | 0.6 | 1.66 | 1.2 | 0.8 | 1.50 | 3.6 | 1.7 | 2.12 |
|  | + | - | + | + | - | 0.6 | 0.7 | 0.86 | 0.7 | 1.2 | 0.58 | 0.4 | 0.6 | 0.67 | 0.1 | 1.0 | 0.10 |
|  | + | + | - | + | - | 0.8 | 1.6 | 0.50 | 0.7 | 2.1 | 0.33 | 2.9 | 2.2 | 1.32 | 0.8 | 2.2 | 0.36 |
|  | - | + | + | - | + | 2.5 | 3.5 | 0.71 | 1.9 | 2.1 | 0.90 | 1.9 | 2.2 | 0.86 | 2.1 | 2.8 | 0.75 |
|  | + | + | - | - | + | 2.0 | 2.0 | 1.00 | 0.4 | 1.3 | 0.30 | 1.5 | 1.6 | 0.94 | 1.7 | 2.0 | 0.85 |
| **4** | - | + | + | + | + | 1.8 | 0.6 | 3.00 | 1.0 | 0.5 | 2.00 | 0.5 | 0.5 | 1.00 | 1.6 | 1.1 | 1.45 |
|  | + | - | + | + | + | 0.2 | 0.1 | 2.00 | 0.1 | 0.2 | 0.50 | 0.0 | 0.1 | 0.00 | 0.3 | 0.3 | 1.00 |
|  | + | + | - | + | + | 0.1 | 0.3 | 0.33 | 0.5 | 0.3 | 1.66 | 1.1 | 0.4 | 2.75 | 1.1 | 0.8 | 1.37 |
|  | + | + | + | - | + | 2.2 | 2.3 | 0.96 | 1.0 | 1.2 | 0.83 | 0.8 | 5.8 | 0.14 | 1.9 | 1.2 | 1.58 |
|  | + | + | + | + | - | 1.0 | 1.8 | 0.55 | 1.5 | 2.0 | 0.75 | 0.7 | 1.4 | 0.50 | 0.9 | 1.3 | 0.69 |
| **5** | + | + | + | + | + | 0.7 | 0.4 | 1.75 | 0.7 | 0.3 | 2.33 | 0.1 | 0.2 | 0.50 | 0.8 | 0.5 | 1.60 |

S: Smoking; PI: Physical Inactivity; UD: Unhealthy Diet; Ob: Obesity; HBP: High Blood Pressure

Table 3 presents the prevalence and the prevalence odds ratios (POR) of combinations of two risk factors regardless of the exposure to the other three risk factors in the intervention group and the control group at pre-and post-assessment. At baseline, obesity also clustered with high blood pressure among employees in both the intervention group and the control group. The calculated PORs, 2.67 (CI95%:1.72 – 4.09) and 2.02 (CI95%:1.31 – 3.12), were the highest, indicating that the presence of these behaviors significantly increased the occurrence of the other behaviors by 2.67 and 2.02, respectively in the intervention group and the control group. However, smoking was inversely clustered with obesity and lack of physical activity at pre- and post-assessment. Indeed, employees who did not smoke were more likely to be obese and physically inactive. Post-assessment evaluation indicated that the clustering of obesity and hypertension increased from POR=2.67 (CI95%:1.72 – 4.09) to POR=3.01 (CI95%:2.04 – 4.39) at pre-and post-intervention, respectively in the intervention group. The same trend was observed for the clustering of unhealthy diet and physical inactivity, increasing from POR=1.29 (CI95%:0.96 – 1.74) to POR=2.78 (CI95%:2.15 – 3.60), at pre-and post-intervention, respectively.

**Table 3: Prevalence (P) and Prevalence Odds Ratios (PORs) of Simultaneous Occurrence of Two NCDs Risk Factors at pre-and post-assessment in the intervention and control groups in Sousse, Tunisia (2009-2014)**

|  | **Intervention group** | | | | **Control group** | | | |
| --- | --- | --- | --- | --- | --- | --- | --- | --- |
|  | **Pre-assessment** | | **Post-assessment** | | **Pre-assessment** | | **Post-assessment** | |
|  | **P %** | **POR (CI_95%_)** | **P %** | **POR (CI_95%_)** | **P %** | **POR (CI_95%_)** | **P %** | **POR (CI_95%_)** |
| **Obesity and hypertension** | 4.9 | 2.67 (1.72 – 4.09) | 5.5 | 3.01 (2.04 – 4.39) | 4.6 | 2.02 (1.31 – 3.12) | 10.7 | 2.41 (1.78 – 3.26) |
| **Obesity and smoking** | 4.1 | 0.53 (0.34 – 0.81) | 5.6 | 0.55 (0.39 – 0.76) | 6.4 | 1.03 (0.70 – 1.52) | 6.4 | 0.63 (0.46 – 0.86) |
| **Obesity and unhealthy diet** | 8.0 | 0.99 (0.68 – 1.44) | 9.5 | 0.92 (0.68 – 1.24) | 6.8 | 0.76 (0.53 – 1.10) | 9.1 | 0.51 (0.38 – 0.68) |
| **Obesity and physical inactivity** | 11.7 | 1.42 (0.92 – 2.20) | 14.0 | 1.34 (0.98 – 1.83) | 13.4 | 0.94 (0.65 – 1.36) | 17.3 | 1.39 (1.04 – 1.85) |
| **Hypertension and smoking** | 6.8 | 0.88 (0.62 – 1.26) | 4.9 | 0.83 (0.58 – 1.19) | 5.1 | 1.21 (0.80 – 1.85) | 10.3 | 1.72 (1.28 – 2.32) |
| **Hypertension and unhealthy diet** | 9.6 | 0.98 (0.69 – 1.38) | 7.0 | 1.07 (0.76 – 1.51) | 5.2 | 0.80 (0.53 – 1.19) | 9.7 | 0.94 (0.70 – 1.26) |
| **Hypertension and physical inactivity** | 12.7 | 0.94 (0.64 – 1.37) | 8.6 | 0.93 (0.65 – 1.31) | 10.7 | 1.09 (0.72 – 1.65) | 14.8 | 0.99 (0.74 – 1.31) |
| **Smoking and physical inactivity** | 26.5 | 0.68 (0.50 – 0.92) | 22.0 | 0.78 (0.61 – 1.01) | 22.8 | 1.19 (0.86 – 1.66) | 16.2 | 0.81 (0.62 – 1.07) |
| **Smoking and unhealthy diet** | 21.8 | 1.18 (0.90 – 1.55) | 18.4 | 1.09 (0.85 – 1.39) | 8.2 | 0.44 (0.31 – 0.61) | 11.4 | 0.99 (0.75 – 1.31) |
| **Unhealthy diet and physical inactivity** | 39.3 | 1.29 (0.96 – 1.74) | 35.4 | 2.78 (2.15 – 3.60) | 29.2 | 1.69 (1.23 – 2.32) | 26.0 | 2.35 (1.79 – 3.10) |

The changes in employees’ prevalence of risk factors clusters for chronic diseases in relation to the socio-demographic characteristics are presented in Table 4. For the entire sample, the prevalence of employees having the aggregation of two risk factors decreased significantly from 40.1% at pre-assessment to 34.5% at post-assessment in the intervention group. However, the prevalence of no and one risk factor increased from 30.8% to 39.7% (p=0.001). In the control group, the prevalence of employees having two risk factors slightly decreased from 37.6% at pre-assessment to 33.4% at post-assessment, and for those having more than three risk factors, the prevalence increased from 20.7% to 25.2% (p=0.2). After stratification according to age, the prevalence of employees having the two-risk-factors cluster and more than three risk factors decreased in the two age groups involving participants over 30 years and those less than 30 years. This decrease was more pronounced for employees aged more than 30 years, from 37.0% and 44.9% for the two-risk-factors cluster and more than three risk factors, respectively at pre-assessment to 31.3% and 26.9%, respectively at post-assessment (p≤10-3). In the control group, the rate of the two-risk-factors cluster decreased but not significantly in both two age groups. With regard to gender, a sharp decrease was noted in the two-risk-factors cluster, from 37.8% to 30.2% (p≤10-3) in the intervention group. For females, a non-significant decrease was noted for the two-risk-factors cluster. In the control group, the prevalence of employees having the aggregation of two risk factors decreased, but not significantly. After stratification based on education, the prevalence of employees having more than three risk factors decreased in all the categories in the intervention group. However, this decrease was significant only among employees who did not finish their primary school and the rate dropped from 31.2% at pre-assessment to 21.1% at post-assessment (p≤10-3). Yet, in the control group, the prevalence of employees having more than three risk factors increased significantly, from 19.4% to 25.8% (p=0.02) at pre- and post-assessment, respectively in those having an intermediate or secondary school level. In the intervention group, the prevalence of employees having the two-risk-factors cluster and more than three risk factors significantly decreased among married employees, from 39% at pre-assessment to 33.2% at post-assessment (p=0.005). In the control group, the rate decreased among employees with and without partners, but not significantly. With regard to the work type, the prevalence of the two-risk-factors cluster and more than three risk factors significantly decreased in blue collars both in the intervention and control group, from 41.5% at pre-assessment to 35.1% at post-assessment and from 28.2% at pre-assessment to 25.3% at post-assessment. Stratification according to the occupational seniority showed a decrease in the prevalence of the two-risk-factors cluster and more than three risk factors in all the categories in the intervention group. The decrease was significant only in employees having job experience between three and ten years. In the control group, no significant changes were noted in the prevalence of the two-risk-factors cluster and more than three risk factors with regard to the occupational seniority. Concerning perceived health, a significant decrease was noted in the prevalence of the two-risk-factors cluster among employees who rated their health as good or very good, from 41.3% at pre-assessment to 29% at post-assessment (p<10-3) in the intervention group. However, the decrease was more pronounced in the prevalence of more than three risk factors among employees who reported an intermediate health status, from 34.8% at pre-assessment to 26.1% at post-assessment (p=0.01). In the control group, no significant variations were noted in the prevalence of the two-risk-factors cluster and more than three risk factors with respect to perceived health status. In the intervention group, the prevalence of the two-risk-factors cluster significantly decreased in the participants believing in health improvement, from 40.7% at pre-assessment to 34.4% at post-assessment (p<10-3). No significant changes were noted in the control group. In the intervention group, the prevalence of the two-risk-factors cluster significantly decreased in only the participants who were not diagnosed with at least one chronic disease, including cardiovascular diseases, diabetes, colorectal cancer, breast cancer, lung cancer, and ear-nose-throat cancer, from 41% at pre-assessment to 35.3% at post- assessment (p=0.001). In the control group, the decrease was not significant. In the intervention group, the prevalence of the two-risk-factors cluster decreased in both employees with and without health insurance. The change was significant only among insured employees, from 38.1% at pre-assessment to 34.7% at post-assessment (p=0.002). In addition, a non-significant increase was noted in the prevalence of more than three risk factors among uninsured employees, from 24.3% at pre-assessment to 25.6% at post-assessment.

**Table 4: Evolution of the prevalence of risk factors clusters for NCDs before and after the intervention according to the employees’ socio-demographic characteristics in the intervention and control groups in Sousse, Tunisia (2009-2014)**

|  |  | **Intervention group** | | | | | | | **Control group** | | | | | | |
| --- | --- | --- | --- | --- | --- | --- | --- | --- | --- | --- | --- | --- | --- | --- | --- |
| **Variable** |  | **Pre**  **n**  **(%)** | | | **Post**  **n**  **(%)** | | | **p** | **Pre**  **n**  **(%)** | | | **Post**  **n**  **(%)** | | | **p** |
|  |  | **0-1** | **2** | **≥3** | **0-1** | **2** | **≥3** |  | **0-1** | **2** | **≥3** | **0-1** | **2** | **≥3** |  |
| **Total** |  | 255 (30.8) | 332 (40.1) | 241 (29.1) | 416 (39.7) | 362 (34.5) | 271 (25.8) | **0.001** | 297 (41.8) | 267 (37.6) | 147 (20.7) | 392 (41.4) | 316 (33.4) | 239 (25.2) | 0.20 |
| **Gender** | Male | 150 (27.1) | 208 (37.6) | 195 (35.3) | 261 (38.0) | 207 (30.2) | 218 (31.8) | **<10^-3^** | 165 (38.4) | 149 (34.7) | 116 (27.0) | 212 (36.0) | 193 (32.8) | 184 (31.2) | 0.33 |
|  | Female | 105 (38.2) | 124 (45.1) | 46 (16.7) | 155 (42.7) | 155 (42.7) | 53 (14.6) | 0.48 | 132 (47.0) | 118 (42.0) | 31 (11.0) | 180 (50.3) | 123 (34.4) | 55  (15.4) | 0.08 |
| **Age (years)** | ≤30 | 229 (33.1) | 284 (41.0) | 179 (25.9) | 332 (39.2) | 299 (35.3) | 217 (25.6) | **0.027** | 195 (42.5) | 178 (38.8) | 86 (18.7) | 241 (44.4) | 180 (33.1) | 122 (22.5) | 0.13 |
|  | >30 | 23 (18.1) | 47 (37.0) | 57 (44.9) | 84 (41.8) | 63 (31.3) | 54 (26.9) | **<10^-3^** | 95 (39.1) | 87  (35.8) | 61 (25.1) | 151 (37.4) | 136 (33.7) | 117 (29.0) | 0.56 |
| **Education level** | Illiterate /Primary school | 66 (24.8) | 117 (44.0) | 83 (31.2) | 142 (46.9) | 97 (32.0) | 64 (21.1) | **<10^-3^** | 26 (32.1) | 32 (39.5) | 23 (28.4) | 60 (42.9) | 50 (35.7) | 30  (21.4) | 0.25 |
|  | Intermediate /Secondary school | 139 (34.2) | 165 (40.6) | 102 (25.1) | 216 (38.3) | 201 (35.6) | 147 (26.1) | 0.25 | 235 (43.4) | 201 (37.2) | 105 (19.4) | 293 (41.5) | 231 (32.7) | 182 (25.8) | 0.02 |
|  | University/master degree | 46 (31.3) | 47 (32.0) | 54 (36.7) | 58 (32.0) | 64 (35.4) | 59 (32.6) | 0.70 | 32 (43.8) | 24  (32.9) | 17 (23.3) | 39 (38.6) | 35 (34.7) | 27  (26.7) | 0.77 |
| **Marital status** | Single/ Widower/divorced | 131 (31.3) | 174 (41.5) | 114 (27.2) | 150 (39.3) | 141 (36.9) | 91 (23.8) | 0.06 | 109 (44.1) | 95 (38.5) | 43  (17.4) | 110 (45.1) | 82 (33.6) | 52  (21.3) | 0.20 |
|  | Married | 120 (30.0) | 156 (39.0) | 124 (31.0) | 265 (39.8) | 221 (33.2) | 179 (26.9) | **0.005** | 183 (40.0) | 171 (37.4) | 103 (22.5) | 279 (40.0) | 233 (33.4) | 186 (26.6) | 0.27 |
| **Occupation** | White collars | 65  (32.7) | 69  (34.7) | 65 (32.7) | 95 (38.8) | 81  (33.1) | 69  (28.2) | 0.37 | 40  (40.4) | 28 (38.3) | 31 (31.3) | 69  (41.8) | 48  (29.1) | 48  (29.1) | 0.93 |
|  | Blue collars | 187 (30.3) | 256 (41.5) | 174 (28.2) | 315 (39.6) | 279 (35.1) | 201 (25.3) | **0.001** | 250 (43.0) | 222 (38.2) | 109 (18.8) | 283 (40.0) | 241 (34.1) | 183 (25.9) | 0.009 |
| **Occupational seniority (year)** | ≤2 | 98 (29.3) | 145 (43.4) | 91 (27.2) | 75 (34.7) | 72 (33.3) | 69 (31.9) | 0.06 | 76 (44.7) | 69 (40.6) | 25 (14.7) | 34 (37.8) | 34 (37.8) | 22 (24.4) | 0.14 |
|  | 3-10 | 103 (31.8) | 119 (36.7) | 102 (31.5) | 206 (40.2) | 177 (34.6) | 129 (25.2) | **0.03** | 71 (40.3) | 67 (38.1) | 38 (21.6) | 98 (48.8) | 61 (30.3) | 42 (20.9) | 0.20 |
|  | >10 | 52 (33.3) | 62 (39.7) | 42 (26.9) | 135 (42.1) | 113 (35.2) | 73 (22.7) | 0.18 | 139 (41.6) | 117 (35.0) | 78 (23.4) | 260 (39.6) | 221 (33.7) | 175 (26.7) | 0.52 |

Table 5 presents the chronic disease cluster "changes in the prevalence of risk factors in the employees" according to health characteristics.

**Table 5: Evolution of the prevalence of risk factors clusters for NCDs before and after the intervention according to the employees’ health characteristics in the intervention and control groups in Sousse, Tunisia (2009-2014)**

| **Variable** |  | **Intervention group** | | | | | | | **Control group** | | | | | | |
| --- | --- | --- | --- | --- | --- | --- | --- | --- | --- | --- | --- | --- | --- | --- | --- |
|  |  | **Pre-assessment**  **n**  **(%)** | | | **Post-assessment**  **n**  **(%)** | | | **p** | **Pre-assessment**  **n**  **(%)** | | | **Post-assessment**  **n**  **(%)** | | | **p** |
|  |  | **0-1** | **2** | **≥3** | **0-1** | **2** | **≥3** |  | **0-1** | **2** | **≥3** | **0-1** | **2** | **≥3** |  |
| **Self-Perceived health** | Very bad /Bad | 18 (34.0) | 23 (43.4) | 12 (22.6) | 42 (40.0) | 39 (37.1) | 24 (22.9) | 0.70 | 12 (37.5) | 9 (28.1) | 11 (34.4) | 23 (32.9) | 19 (27.1) | 28 (40.0) | 0.84 |
|  | Intermediate | 80 (26.8) | 115 (38.5) | 104 (34.8) | 158 (35.6) | 170 (38.3) | 116 (26.1) | **0.01** | 78 (40.6) | 70 (36.5) | 44 (22.9) | 146 (39.2) | 131 (35.2) | 95 (25.5) | 0.79 |
|  | Good /Very good | 134 (31.1) | 178 (41.3) | 119 (27.6) | 184 (42.3) | 126 (29.0) | 125 (28.7) | **<10^-3^** | 180 (41.4) | 168 (38.6) | 87 (20.0) | 205 (44.6) | 149 (32.4) | 106 (23.0) | 0.14 |
|  | Excellent | 22 (61.1) | 9  (25.0) | 5 (13.9) | 28 (47.5) | 27 (45.8) | 4 (6.8) | 0.10 | 24 (55.8) | 16 (37.2) | 3 (7.0) | 18 (43.9) | 15 (36.6) | 8 (19.5) | 0.21 |
| **Believe in health improvement** | Disagree | 24 (30.4) | 30 (38.0) | 25 (31.6) | 11 (34.4) | 11 (34.4) | 10 (31.3) | 0.90 | 10 (31.3) | 17 (53.1) | 5  (15.6) | 17 (35.4) | 14 (29.2) | 17 (35.4) | 0.06 |
|  | Agree | 225 (30.5) | 300 (40.7) | 213 (28.9) | 402 (39.8) | 347 (34.4) | 261 (25.8) | **<10^-3^** | 280 (42.4) | 242 (36.6) | 139 (21.0) | 368 (41.6) | 296 (33.4) | 221 (25.0) | 0.16 |
| **Chronic diseases*** | No | 240 (31.8) | 309 (41.0) | 205 (27.2) | 391 (40.4) | 342 (35.3) | 236 (24.4) | **0.001** | 281 (42.8) | 247 (37.7) | 128 (19.5) | 359 (43.4) | 283 (34.2) | 186 (22.5) | 0.25 |
|  | Yes | 15 (20.3) | 23 (31.1) | 36 (48.6) | 25 (31.3) | 20 (25.0) | 35 (43.8) | 0.28 | 16 (29.1) | 20 (36.4) | 19 (34.5) | 33 (27.7) | 33 (27.7) | 53 (44.5) | 0.39 |
| **Health insurance** | Yes | 195 (31.0) | 240 (38.1) | 195 (31.0) | 380 (39.5) | 333 (34.7) | 248 (25.8) | **0.002** | 232 (41.5) | 203 (36.3) | 124 (22.2 | 361 (40.6) | 299 (33.6) | 229 (25.8) | 0.27 |
|  | No | 58 (31.4) | 82 (44.3) | 45 (24.3) | 32 (39.0) | 29 (35.4) | 21 (25.6) | 0.34 | 61 (42.7) | 60  (42.0) | 22 (15.4) | 27 (54.0) | 14 (28.0) | 9 (18.0) | 0.21 |

*The included chronic diseases were as follows: cardiovascular diseases, diabetes, colorectal cancer, breast cancer, lung cancer, and ear-nose-throat cancer.

Table 6 presents the changes in the employees’ prevalence of risk factors clusters for chronic diseases with respect to the health characteristics. In the intervention group, multiple logistic regression analysis revealed that men were more likely to have a risk score of three or more (OR=2.38, p<10-3) than women. Employees aged more than 30 years were more likely to have a risk score of three or more versus zero or one (OR=1.39, p=0.019) compared to younger employees. The same was noted in employees with less than two years of work experience compared to those having more than 10 years of work experience (OR=1.71, p=0.006) and those diagnosed with at least one chronic disease (OR=2.44, p<10-3). Employees who reported an intermediate perceived health were considerably more likely to have a risk score of three or more compared to zero or one risk factor (OR=5.16, p= p<10-3) than those who reported an excellent perceived health. Overall, the odds ratios for the comparisons of two versus zero or one risk factors were lower compared to those of three or four versus zero or one risk factors. The odds ratios for the two factor comparisons revealed that employees working for less than two years were more likely to have two risk factors compared to zero or one risk factor than those working for over than 10 years (OR=1.53, p=0.015). Employees who reported an intermediate perceived health were modestly more likely to have a risk score of two compared to zero or one risk factor than those who reported an excellent perceived health (OR=1.70, p=0.026). The intervention had a significant protective effect in the intervention group in reducing the likelihood of the multiple risk factors occurrence with OR=0.70 (p=0.018) and OR=0.74 (p=0.002) for the risk scores two and three or more, respectively. In the control group, the intervention had no significant effect in the stepwise method for the two-risk factor aggregation (OR=0.87, p=0.255). Furthermore, the time factor increased the likelihood of the risk score three or more, but not significantly (OR=1.12, p=0.378).

**Table 6: Ordinal regression analysis of multiple risk factors among employees in the intervention and control groups in Sousse, Tunisia, 2009-2014, with 0 or 1 risk factor as the reference category.**

| **Variables** | **Intervention group** | | **Control group** | |
| --- | --- | --- | --- | --- |
|  | **OR (CI_95%_)** | **P** | **OR (CI_95%_)** | **P** |
| **Intervention effect** | 0.81 (0.68-0.97) | 0.025 | 1.01 (0.84-1.21) | 0.937 |
| **Gender** | 1.62 (1.35-1.96) | <10^-3^ | 1.96 (1.62-2.37) | <10^-3^ |
| **Age (years)** **>30** (≤30) | 1.28 (1.05-1.55) | 0.016 | - | - |
| **Occupational seniority (year)** (>10) |  |  |  |  |
| ≤2 | 1.51 (1.15-1.98) | 0.003 | - | - |
| 3-10 | 1.25 (0.99-1.57) | 0.051 | - | - |
| **Chronic diseases* yes** (no) | 2.10 (1.52-2.90) | <10^-3^ | 2.05 (1.52-2.77) | <10^-3^ |
| **Perceived health** (excellent) |  |  |  |  |
| Very bad /Bad | 2.07 (1.37-3.13) | 0.008 | 2.45 (1.41-4.26) | 0.001 |
| Intermediate | 2.59 (1.71-3.93) | <10^-3^ | 1.67 (1.07-2.60) | 0.023 |
| Good /Very good | 1.95 (1.19-3.20) | 0.001 | 1.39 (0.90-2.14) | 0.131 |

*The included chronic diseases were as follows: cardiovascular diseases, diabetes, colorectal cancer, breast cancer, lung cancer, and ear-nose-throat cancer.

**Discussion**

One of our main findings in the present study was the decrease in the rate of employees having two or more risk factors in the intervention group, as opposed to the increase in the rate of employees in the control group. These results clearly illustrate the need to adopt a new paradigm of research in order to produce greater effect on public health with reduced health care costs. A growing body of literature has outlined several attempts to modify multiple lifestyle-related risk factors on a population scale, and it has demonstrated the feasibility and potential effectiveness of this approach. [22]. In addition, multiple unhealthy behaviors are often present simultaneously. It is therefore important to study the clustering of lifestyle risk factors because of the possible synergistic effects on health. Indeed, knowing the clustering tendency of risk factors and targeting change in multiple health behaviors are the key approaches to an effective prevention of chronic diseases. [23,24]. There is some evidence that combinations of lifestyle risk factors are more detrimental to people's health than what can be expected from the added individual effects alone [15,20], suggesting that the health effects of lifestyle risk factors are multiplicative rather than cumulative. The fact that multiple risk factors are the norm in the adult population argues strongly in favor of multiple-behavior interventions rather than single-behavior interventions. [16,21]. Multiple-behavior interventions may not only have a much greater impact on public health compared to single-behavior interventions [24], but they may also be more effective and efficient in achieving these goals [17]. Additionally, the workplace presents a suitable environment to prevent multiple risk factors, where it could be advantageous for both employees and employers [4].

When adjusting for changes in the number of NCD risk factors according to the participating employees’ characteristics, improvements in adherence to the five recommendations were found to increase, particularly among male participants, those over 30 years, and among workers. Thus, the determining factors of the employees’ response to the intervention were questioned. This trend could partly be explained by the perception of being at risk among the older employees and the close association between the adherence rate and the type of job. According to Hanlon et al. [25], the perception of being at risk improves the responsiveness to such workplace interventions. Thus, it would be logical to target those who perceive themselves to be in need of healthy change and who worry about the information given to them. The positive effect among males rather than females prompts us to wonder about the mechanisms responsible for the disproportionate change according to gender. Further research is needed to identify this pattern. Indeed, the POR values from the cluster analysis indicate that unhealthy diet is associated with physical inactivity and that obesity is associated with hypertension, independently of the exposure to other risk factors, both before and after assessment in the intervention and control groups. However, the combination “physical inactivity, unhealthy diet, obesity, high blood pressure” was 300% higher than what would be expected if the factors were independent (O/E=3) at baseline and it decreased slightly at post-assessment. However, it increased significantly in the control group. Although the improvement in the clusters of NCD risk factors is unlikely to be statistically significant, we believe that the results reported in the present study are important because this workplace health promotion program was able to identify at-risk employees and to provide access to health promotion activities to improve health. These features imply the need for deeper structural changes of environmental dietary and physical activity to tackle the obesogenic and hypertensive environment. Indeed, combining environmental interventions in the worksites [26] with a multi-sectoral approach [27] would be the cornerstone of an efficient and cost-effective action for NCD risk factors prevention, where, for instance, food industry may play a key role in addressing NCDs pattern through food reformulation, consumer information, responsible marketing, and accepting public private partnership [27]. The North Karelia Project, raising the slogan of ‘Health in All Policies’ [28], is a benchmarking example of a successful multi-sectoral prevention project of non-communicable diseases. It evolved from a demonstration project to a national policy, involving health and other services, schools, non-governmental organizations (NGOs), innovative media campaigns, local media, supermarkets, food industry, agriculture, etc. Indeed, a strategy to sustain an economic environment that can offer citizens a better quality of life is an absolute need since many interventions addressing poverty and development proved to have great potential on NCDs prevalence and risk [29]. Nevertheless, our attempts to make structural and environmental changes were limited by the political instability following the political and social events of 2010-2011 [30]. Due to the socio-political conditions after the revolution, the authorities and policy makers at the Ministry of Health and other sectors had other priorities and challenges than the fight against non-communicable diseases and their risk factors [31].

**Implications for clustering of risk factors for NCDs**

This study suggests a novel approach that takes into account the interplay of risk variables in analyzing the impact of interventions on a variety of health behaviors rather than examining the effectiveness of intervention programs by reducing individual risk factors. Clusters or patterns of harmful behaviors in adult populations and the associated socio-demographic characteristics have been highlighted in numerous earlier studies with regard to a variety of health behaviors. [32] The influence of intervention studies on these clusters and the compliance with healthy lifestyles and health standards, however, have received less attention in the public health literature. [33].

**Predictors for change in clustering of NCD risk factors**

The multinomial logistic model suggested five independent variables determining the clustering of risk habits in the intervention group involving male gender, more than 30 years of age, less than two years work experience, lower self-perceived health, and having at least one chronic disease. In a large study population derived from the 2003 Health Survey for England, Poortinga [11] reported that men, belonging to lower social class households, being single, and being economically inactive are more likely to have a higher number of lifestyle risk factors. However, older age groups and homeowners are less likely to have a higher number of lifestyle risk factors. Pronk et al. [34] reported the prevalence and cluster patterns of multiple healthy lifestyle factors among a random sample from a large Midwestern health plan. The assessed lifestyle-related health factors were physical activity, nonsmoking, high-quality diet, healthy weight for all subjects, and alcohol consumption. Only 10.8% of adults were found to meet all the five behavior-related factors. For adults, being in the 50- to 64-year-old cohort (OR1.46, p0.05), having a college degree (OR1.65; p 0.05), and having no chronic disease (OR1.92; p 0.05) were all associated with an increased likelihood to be in adherence to multiple healthy lifestyle factors. Raitakari et al. [32] studied the occurrence of common lifestyle risk factors, such as unhealthy diet, smoking, physical inactivity, and alcohol use in a cohort of 484 young adults aged between 18 and 24 years. The authors investigated the predictive factors for risk habit clustering, including male sex, aggressiveness, and past unemployment. Paying a lot of attention to health habits, higher education (being a student), good self-perceived health, and a high sense of responsibility seem to be protective factors against risk habit clustering. The 2007 China Chronic Disease and Risk Factor Surveillance provided data relating to NCD risk factors in a nationally representative sample of 49 247 Chinese aged 15 to 69 years. The risk factors were as follows: tobacco use, excessive alcohol drinking, insufficient intake of vegetables and fruits, physical inactivity, and overweight or obesity. In their study, Yichong Li et al. [35] reported that 57 % of a Chinese population have at least two lifestyle risk factors and the mean number of NCDs risk factors is 1.80 (95% CI: 1.78–1.83). Chinese of older age, being a male, living in rural areas, having lower education level and lower yearly household income were found to increase the likelihood of having higher NCD risk factors. Although the study design used was a quasi-experimental one, as in several other international interventions [36,37], it is possible to affirm that the risk factors change and evolution was due to the intervention because the workplace is generally a stable environment. Indeed, the same participants were assessed at the start and at the end of the intervention. However, the results from the multinomial multilevel regression model demonstrated the advantage of the present intervention that was more intensive and interactive. It proved to have a significant protective effect in the intervention group in reducing the likelihood of the multiple risk factors occurrence with ORa=0.70 (C.I95%: [0.56-0.88]; p=0.018) and ORa=0.74 (C.I95%: [0.57-0.95]; p=0.002) for the risk scores two and three or more, respectively. In the control group, adjusted ORs were not significant, which demonstrated the effect of the present intervention.

The results of the present study highlighted that the three-year workplace-based intervention was more effective than usual actions in reducing the co-occurrence and aggregation of multiple NCD risk factors. The project in its current form can therefore be recommended for implementation in worksites while taking in consideration all the cultural and individual needs that affect the clustering pattern of multiple NCD risk factors. Furthermore, this study can be considered a leading one in terms of describing the clustering phenomenon in a representative sample that takes into account the interaction between risk factors to investigate the effectiveness of the intervention on the cluster of NCD risk factors, and to explore predictors of the risk factors associated with NCDs in Tunisian working population.

To the best of the authors’ knowledge, this research is one of the fewest studies using an intervention scheme and it is the first to offer multi-component interventions targeting multiple NCD risk factors in a workplace setting in Tunisia. Although the co-occurrence of these risk factors is associated with further increased risk of morbidity and mortality [38], the majority of health promotion interventions typically address them as separate entities [39,40].

Workplaces have the potential for creating in-built social support, i.e., active collaboration of employees in making sustainable changes to attain a healthy lifestyle [41], thus probably reducing the degree of individual effort and motivation needed to make behavioral changes. Therefore, the changes in lifestyle achieved at work are thought to be sustainable in the long term. Most lifestyle programs often focus on short-term behavioral change. Indeed, only few studies have a follow-up assessment beyond one-year post-baseline. Indeed, only longer-term behavioral lifestyle changes can have a lasting impact on individual health [42]. The existence of a comparison group was another strength in the present study. Comparing results with those of a control group would further strengthen the assumption that the intervention program was effective in reducing cardiovascular diseases risk [43]. Despite the innovative approach of this intervention program in Tunisia, this study has some limitations that need to be considered.

For the purpose of feasibility and convenience, a quasi-experimental, non-randomized design was adopted with an added control group to strengthen the findings. Nevertheless, using a quasi-experimental nonequivalent control group design usually creates less compelling support for counterfactual interferences compared to a randomized controlled trial, which might limit the ability to illuminate causal inference [44]. Moreover, it does not provide certitude that the improvement in the targeted risk factors is the result of the intervention. Independent groups were compared at pre-and post-assessment and some socio-demographic characteristics were different. Individual-natural changes in NCD risk factors could therefore have been missed in the present approach. A multivariate analysis was therefore conducted to control this statistically. In addition, the sample was not truly population-based but rather a convenience sample. Although this may limit generalizability to the general population, the results reported in the present study would be broadly applicable to worksite intervention programs involving middle-aged professionals. However, the findings could not be generalized to all worksites in Sousse as employees outside the industrial sector were not taken into account. Moreover, reliance on self-report is another limitation, which may lead to social desirability bias. Self-assessment of the studied behaviors could also have led to recall bias. Although we attempted to mitigate potential measurement error by incorporating widely used standardized measures, it was most problematic for the assessment of physical activity and dietary intake, and it may provide over or under-estimates for some individuals. To manage this risk, standardization of data collection was performed by trained physician interviewers. Additionally, the present study could have been subject to selection bias related to the level of motivation with regard to willingness to adopt a healthier lifestyle similar to other healthy-lifestyle promotion programs. Individuals choosing to participate may have been motivated by factors unrelated to this specific program. Furthermore, certain environmental changes did not occur until the last part of the intervention period, which may represent another limitation. Changes that were easy to make (workshops and educational sessions for promoting healthy lifestyle) took place quickly and they were performed directly and entirely by research staff. However, the interventions requiring employer resources (providing healthy food in the companies’ canteens and cafeterias) tended to be delayed, probably due to the cost and the level of administrative approval needed.

**Conclusion**

A critical approach for addressing NCDs, particularly with the limited clinical capacity, is prevention through policies that reduce unhealthy behaviors and risk factors. The workplace is internationally recognized as a crucial area to tackle these health issues; however, it is often marginalized at national level. The results in the present study together with the data reported in the literature demonstrate the feasibility and effectiveness of these health promotion programs in preventing particular cluster patterns. Generalization of these programs is possible by their integration into a national health policy. Health care systems alone are not effective in the fight against chronic diseases and their risk factors. Thus, a holistic approach based on scientific evidence supplemented by partnering across a variety of sectors is strongly needed. Strategies for the prevention and control of NCDs should be implemented at a multi-sectoral level with the implication of stakeholders and policy makers to undertake legislative, environmental, and structural changes in addition to the community involvement. This could facilitate the collaboration of other sectors that are indirectly involved in health, such as transport, agriculture, education, trade, finance, and social affairs. Meanwhile, the Ministry of Health and the Ministry of labor should collaborate to promote research on workers’ health needs, particularly by framing special research agendas, giving them priority in national research programs and grant schemes, and fostering practical and participatory research. Making a difference to workplace health requires that employers, government, and health-care professionals operate in harmony with each other. Finally, civil society and the private sector should share responsibility for health and wellbeing through innovative partnerships, exchanging expertise and knowledge, and mobilizing resources.

**Acknowledgements.**

We would like to thank all the participants and those who contributed in different settings during this 4-year project. The authors would also like to thank Prof. Samir Boukattaya for proofreading and editing the manuscript.

**References**

1.	Le HT, Le TA, Mac TD, Nguyen DN, Vu HN, Truong ATM, et al. Non-communicable diseases prevention in remote areas of Vietnam: Limited roles of health education and community workers. Angkurawaranon C, éditeur. PLoS ONE. 26 sept 2022;17(9):e0273047.

2.	Irani F, Coquoz E, von Wolff M, Bitterlich N, Stute P. Awareness of non-communicable diseases in women: a cross-sectional study. Arch Gynecol Obstet. 14 avr 2022;306(3):801‑10.

3.	Bytyci-Katanolli A, Merten S, Kwiatkowski M, Obas K, Gerold J, Zahorka M, et al. Non-communicable disease prevention in Kosovo: quantitative and qualitative assessment of uptake and barriers of an intervention for healthier lifestyles in primary healthcare. BMC Health Serv Res. déc 2022;22(1):647.

4.	Allen L, Cobiac L, Townsend N. Quantifying the global distribution of premature mortality from non-communicable diseases. J Public Health (Oxf). 1 déc 2017;39(4):698‑703.

5.	Goldstein MG, Whitlock EP, DePue J, Planning Committee of the Addressing Multiple Behavioral Risk Factors in Primary Care Project. Multiple behavioral risk factor interventions in primary care. Summary of research evidence. Am J Prev Med. Août 2004;27(2 Suppl):61‑79.

6.	Ben Ayed H, Ben Hmida M, Ben Jemaa M, Trigui M, Jedidi J, Karray R,et al. Le tabagisme actif : un facteur de risque majeur des maladies non transmissibles humaines dans une enquête hospitalière. Revue des Maladies Respiratoires. 1 févr 2019;36(2):171‑8.

7.	Khelil M, Ben Abdelaziz A, Zanina Y, Yahia F, Ben Hassine D, Ben Rejeb N,et al. Epidemiology of obesity in Tunisia: HSHS4 study (Hammam Sousse Sahloul Heart Study). Tunis Med. juill 2022;100(7):551‑60.

8.	Lim SS, Vos T, Flaxman AD, Danaei G, Shibuya K, et al. A comparative risk assessment of burden of disease and injury attributable to 67 risk factors and risk factor clusters in 21 regions, 1990-2010: a systematic analysis for the Global Burden of Disease Study 2010. Lancet. 15 déc 2012;380(9859):2224‑60.

9.	McAloney K, Graham H, Law C, Platt L. A scoping review of statistical approaches to the analysis of multiple health-related behaviours. Prev Med. juin 2013;56(6):365‑71.

10.	Baruth M, Wilcox S, Egan BM, Dowda M, Laken M, Warren TY. Cardiovascular Disease Risk Factor Clustering among African American Adults. Ethn Dis. 2011;21(2):129‑34.

11.	Poortinga W. The prevalence and clustering of four major lifestyle risk factors in an English adult population. Prev Med. févr 2007;44(2):124‑8.

12.	Steyn K, Damasceno A. Lifestyle and Related Risk Factors for Chronic Diseases. In: Jamison DT, Feachem RG, Makgoba MW, Bos ER, Baingana FK, Hofman KJ, Rogo KO, éditeurs. Disease and Mortality in Sub-Saharan Africa [Internet]. 2nd éd. Washington (DC): The International Bank for Reconstruction and Development / The World Bank; 2006 [cité 8 mars 2023]. Disponible sur: http://www.ncbi.nlm.nih.gov/books/NBK2290/

13.	Global action plan for the prevention and control of NCDs 2013–2020 [Internet]. [cité 8 mars 2023]. Disponible sur: https://www.who.int/publications-detail-redirect/9789241506236

14.	Occupational health [Internet]. [cité 8 mars 2023]. Disponible sur: https://www.who.int/health-topics/occupational-health

15.	Community Organizing and Community Building for Health and Welfare [Internet]. Rutgers University Press. [cité 8 mars 2023]. Disponible sur: https://www.rutgersuniversitypress.org/community-organizing-and-community-building-for-health-and-welfare/9780813553009/

16.	Sorensen G, Emmons K, Hunt MK, Barbeau E, Goldman R, Peterson K, et al. Model for incorporating social context in health behavior interventions: applications for cancer prevention for working-class, multiethnic populations. Prev Med. sept 2003;37(3):188‑97.

17.	Fernstrom MH, Reed KA, Rahavi EB, Dooher CC. Communication strategies to help reduce the prevalence of non-communicable diseases: proceedings from the inaugural IFIC Foundation Global Diet and Physical Activity Communications Summit. Nutr Rev. mai 2012;70(5):301‑10.

18.	Physical activity [Internet]. [cité 8 mars 2023]. Disponible sur: https://www.who.int/news-room/fact-sheets/detail/physical-activity

19.	Healthy diet [Internet]. [cité 8 mars 2023]. Disponible sur: https://www.who.int/news-room/fact-sheets/detail/healthy-diet

20.	Obesity and overweight [Internet]. [cité 8 mars 2023]. Disponible sur: https://www.who.int/news-room/fact-sheets/detail/obesity-and-overweight

21.	Mancia G, Fagard R, Narkiewicz K,et al. 2013 ESH/ESC Guidelines for the management of arterial hypertension: the Task Force for the management of arterial hypertension of the European Society of Hypertension (ESH) and of the European Society of Cardiology (ESC). J Hypertens. juill 2013;31(7):1281‑357.

22.	World Health Organization, Burton J. WHO healthy workplace framework and model: background and supporting literature and practices [Internet]. World Health Organization; 2010 [cité 16 mars 2023]. 93 p. Disponible sur: https://apps.who.int/iris/handle/10665/113144

23.	Bhiri S, Maatoug J, Zammit N, Msakni Z, Harrabi I, Amimi S, et al. A 3-Year Workplace-Based Intervention Program to Control Noncommunicable Disease Risk Factors in Sousse, Tunisia. J Occup Environ Med. juill 2015;57(7):e72-77.

24.	Renggli A, Beer M, Mühlemann P, Stoffel-Kurt N. Sixième rapport sur la nutrition en Suisse et Stratégie alimentaire suisse 2013–2016. Bulletin des médecins suisses. 30 janv 2013;94(05):173‑5.

25.	Hanlon P, Carey L, Tannahill C, Kelly M, Gilmour H, Tannahill A, et al. Behaviour Change Following a Workplace Health Check: How Much Change Occurs and Who Changes? Health Promotion International. 1 janv 1998;13(2):131‑9.

26.	Global Burden of Disease (GBD) [Internet]. Institute for Health Metrics and Evaluation. 2014 [cité 16 mars 2023]. Disponible sur: https://www.healthdata.org/gbd

27.	Philip PM, Kannan S, Parambil NA. Community-based interventions for health promotion and disease prevention in noncommunicable diseases: A narrative review. J Educ Health Promot. 27 nov 2018;7:141.

28.	Puska P, Jaini P. The North Karelia Project: Prevention of Cardiovascular Disease in Finland Through Population-Based Lifestyle Interventions. Am J Lifestyle Med. 19 mars 2020;14(5):495‑9.

29.	Manderson L, Jewett S. Risk, lifestyle and non-communicable diseases of poverty. Global Health. 2 mars 2023;19(1):13.

30.	Saleh SS, Alameddine MS, Natafgi NM, Mataria A, Sabri B, Nasher J, et al. The path towards universal health coverage in the Arab uprising countries Tunisia, Egypt, Libya, and Yemen. Lancet. 25 janv 2014;383(9914):368‑81.

31.	Jabbour S. Health and contemporary change in the Arab world. Lancet. 1 févr 2014;383(9915):477‑9.

32.	Raitakari OT, Leino M, Räkkönen K, Porkka KV, Taimela S, Räsänen L, et al. Clustering of risk habits in young adults. The Cardiovascular Risk in Young Finns Study. Am J Epidemiol. 1 juill 1995;142(1):36‑44.

33.	Engbers LH, van Poppel MNM, Chin A Paw MJM, van Mechelen W. Worksite health promotion programs with environmental changes: a systematic review. Am J Prev Med. juill 2005;29(1):61‑70.

34.	Pronk NP, Anderson LH, Crain AL, Martinson BC, O’Connor PJ, Sherwood NE, et al. Meeting recommendations for multiple healthy lifestyle factors. Prevalence, clustering, and predictors among adolescent, adult, and senior health plan members. Am J Prev Med. août 2004;27(2 Suppl):25‑33.

35.	Li Y, Feng X, Zhang M, Zhou M, Wang N, Wang L. Clustering of cardiovascular behavioral risk factors and blood pressure among people diagnosed with hypertension: a nationally representative survey in China. Sci Rep. 9 juin 2016;6:27627.

36.	Schouw D, Mash R, Kolbe-Alexander T. Transforming the workplace environment to prevent non-communicable chronic diseases: participatory action research in a South African power plant. Glob Health Action. 26 nov 2018;11(1):1544336.

37.	Sorensen G, Landsbergis P, Hammer L, Amick BC, Linnan L, Yancey A, et al. Preventing Chronic Disease in the Workplace: A Workshop Report and Recommendations. Am J Public Health. déc 2011;101(Suppl 1):S196‑207.

38.	Weycker D, Nichols GA, O’Keeffe-Rosetti M, Edelsberg J, Khan ZM, Kaura S, et al. Risk-factor clustering and cardiovascular disease risk in hypertensive patients. Am J Hypertens. juin 2007;20(6):599‑607.

39.	Allan J, Querstret D, Banas K, de Bruin M. Environmental interventions for altering eating behaviours of employees in the workplace: a systematic review. Obes Rev. févr 2017;18(2):214‑26.

40.	Reed JL, Prince SA, Elliott CG, Mullen KA, Tulloch HE, Hiremath S, et al. Impact of Workplace Physical Activity Interventions on Physical Activity and Cardiometabolic Health Among Working-Age Women: A Systematic Review and Meta-Analysis. Circ Cardiovasc Qual Outcomes. févr 2017;10(2):e003516.

41.	Plotnikoff R, Karunamuni N. Reducing sitting time: the new workplace health priority. Arch Environ Occup Health. 2012;67(3):125‑7.

42.	Coffeng JK, van der Ploeg HP, Castellano JM, Fernández-Alvira JM, Ibáñez B, García-Lunar I, et al. A 30-month worksite-based lifestyle program to promote cardiovascular health in middle-aged bank employees: Design of the TANSNIP-PESA randomized controlled trial. Am Heart J. févr 2017;184:121‑32.

43.	White K, Jacques PH. Combined diet and exercise intervention in the workplace: effect on cardiovascular disease risk factors. AAOHN J. mars 2007;55(3):109‑14.

44.	MacCoun R. Experimental and Quasi-Experimental Designs for Generalized Causal Inference, by William R. Shadish, Thomas D. Cook, and Donald T. Campbell. Boston: Houghton Mifflin, 2001, 623 pp., $65.56. Journal of Policy Analysis and Management. 2003;22(2):330‑2.
