# Supplementary material for: Intervention effectiveness in reducing the clustering of non-communicable disease risk factors in the workplace: A quasi-experimental study
Source: PLoS One. 2025 Feb 6;20(2):e0317460. doi: 10.1371/journal.pone.0317460 (PMC11801702; doi:10.1371/journal.pone.0317460)
Supplement: Peer review history

# Author comment: Author response to Decision Letter 0

**DOI:** 10.1371/journal.pone.0317460.r001

10 Oct 2023

# Peer review: Decision Letter 0

**DOI:** 10.1371/journal.pone.0317460.r002

**Authors:** Johanna Pruller

11 Mar 2024

PONE-D-23-30907Intervention effectiveness in reducing the clustering of non-communicable disease risk factors in workplace: a quasi-experimental studyPLOS ONE

Dear Dr. Guesmi,

Thank you for submitting your manuscript to PLOS ONE. After careful consideration, we feel that it has merit but does not fully meet PLOS ONE’s publication criteria as it currently stands. Therefore, we invite you to submit a revised version of the manuscript that addresses the points raised during the review process.

==============================

Please note that we have only been able to secure a single reviewer to assess your manuscript. We are issuing a decision on your manuscript at this point to prevent further delays in the evaluation of your manuscript. Please be aware that the editor who handles your revised manuscript might find it necessary to invite additional reviewers to assess this work once the revised manuscript is submitted. However, we will aim to proceed on the basis of this single review if possible. The reviewer has raised multiple major concerns, specifically they recommend that you improve the details of the methods section and carefully proof read the entire manuscript. Could you please carefully revise the manuscript to address all comments raised?

We also note that the reviewer has recommended that you cite specific previously published works. As always, we recommend that you please review and evaluate the requested works to determine whether they are relevant and should be cited. It is not a requirement to cite these works. We appreciate your attention to this request.

==============================

Please submit your revised manuscript by Apr 25 2024 11:59PM. If you will need more time than this to complete your revisions, please reply to this message or contact the journal office at plosone@plos.org . When you're ready to submit your revision, log on to https://www.editorialmanager.com/pone/ and select the 'Submissions Needing Revision' folder to locate your manuscript file.

Please include the following items when submitting your revised manuscript:

- A rebuttal letter that responds to each point raised by the academic editor and reviewer(s). You should upload this letter as a separate file labeled 'Response to Reviewers'.
- A marked-up copy of your manuscript that highlights changes made to the original version. You should upload this as a separate file labeled 'Revised Manuscript with Track Changes'.
- An unmarked version of your revised paper without tracked changes. You should upload this as a separate file labeled 'Manuscript'.

If you would like to make changes to your financial disclosure, please include your updated statement in your cover letter. Guidelines for resubmitting your figure files are available below the reviewer comments at the end of this letter.

If applicable, we recommend that you deposit your laboratory protocols in protocols.io to enhance the reproducibility of your results. Protocols.io assigns your protocol its own identifier (DOI) so that it can be cited independently in the future. For instructions see: https://journals.plos.org/plosone/s/submission-guidelines#loc-laboratory-protocols . Additionally, PLOS ONE offers an option for publishing peer-reviewed Lab Protocol articles, which describe protocols hosted on protocols.io. Read more information on sharing protocols at https://plos.org/protocols?utm_medium=editorial-email&utm_source=authorletters&utm_campaign=protocols .

We look forward to receiving your revised manuscript.

Kind regards,

Johanna Pruller, Ph.D.

Staff Editor

PLOS ONE

Journal Requirements:

When submitting your revision, we need you to address these additional requirements.

1. Please ensure that your manuscript meets PLOS ONE's style requirements, including those for file naming. The PLOS ONE style templates can be found at 

https://journals.plos.org/plosone/s/file?id=wjVg/PLOSOne_formatting_sample_main_body.pdf and 

https://journals.plos.org/plosone/s/file?id=ba62/PLOSOne_formatting_sample_title_authors_affiliations.pdf

2. Thank you for stating the following financial disclosure: 

"This article was based on a project funded by the "UnitedHealth Group" and by the Research Unit "Santé" UR12SP28". "

Please state what role the funders took in the study.  If the funders had no role, please state: ""The funders had no role in study design, data collection and analysis, decision to publish, or preparation of the manuscript."" 

If this statement is not correct you must amend it as needed. 

Please include this amended Role of Funder statement in your cover letter; we will change the online submission form on your behalf.

3. In the online submission form, you indicated that [The data underlying the results presented in the study are available from the corresponding author upon request.]. 

All PLOS journals now require all data underlying the findings described in their manuscript to be freely available to other researchers, either 1. In a public repository, 2. Within the manuscript itself, or 3. Uploaded as supplementary information.

This policy applies to all data except where public deposition would breach compliance with the protocol approved by your research ethics board. If your data cannot be made publicly available for ethical or legal reasons (e.g., public availability would compromise patient privacy), please explain your reasons on resubmission and your exemption request will be escalated for approval. 

4. Please include your full ethics statement in the ‘Methods’ section of your manuscript file. In your statement, please include the full name of the IRB or ethics committee who approved or waived your study, as well as whether or not you obtained informed written or verbal consent. If consent was waived for your study, please include this information in your statement as well. 

[Note: HTML markup is below. Please do not edit.]

Reviewers' comments:

Reviewer's Responses to Questions

**Comments to the Author**

1. Is the manuscript technically sound, and do the data support the conclusions?

The manuscript must describe a technically sound piece of scientific research with data that supports the conclusions. Experiments must have been conducted rigorously, with appropriate controls, replication, and sample sizes. The conclusions must be drawn appropriately based on the data presented. 

Reviewer #1: Partly

**********

2. Has the statistical analysis been performed appropriately and rigorously? 

Reviewer #1: Yes

**********

3. Have the authors made all data underlying the findings in their manuscript fully available?

The PLOS Data policy requires authors to make all data underlying the findings described in their manuscript fully available without restriction, with rare exception (please refer to the Data Availability Statement in the manuscript PDF file). The data should be provided as part of the manuscript or its supporting information, or deposited to a public repository. For example, in addition to summary statistics, the data points behind means, medians and variance measures should be available. If there are restrictions on publicly sharing data—e.g. participant privacy or use of data from a third party—those must be specified.

Reviewer #1: Yes

**********

4. Is the manuscript presented in an intelligible fashion and written in standard English?

PLOS ONE does not copyedit accepted manuscripts, so the language in submitted articles must be clear, correct, and unambiguous. Any typographical or grammatical errors should be corrected at revision, so please note any specific errors here.

Reviewer #1: No

**********

5. Review Comments to the Author

Please use the space provided to explain your answers to the questions above. You may also include additional comments for the author, including concerns about dual publication, research ethics, or publication ethics. (Please upload your review as an attachment if it exceeds 20,000 characters)

Reviewer #1: Dear Author.

The work in this study relevant. However, there are several important gaps. What frameworks were used to derive at the programs to be implemented? How would another organisation be able to replicate what was done in this study? You omit details in the methodology which you only mention in the discussion for the first time. Please let your methodology speak to your results and discussion. There are examples of studies in the workplace that focus on prevention of NCDs in LMIC - I have made a comment in the manuscript for you to look at. Please make use of an editor as there are words that are ambiguous and makes it difficult to read. There are however certain places in the manuscript where the English is written better than at other places.

**********

6. PLOS authors have the option to publish the peer review history of their article (what does this mean? ). If published, this will include your full peer review and any attached files.

If you choose “no”, your identity will remain anonymous but your review may still be made public.

**Do you want your identity to be public for this peer review?** For information about this choice, including consent withdrawal, please see our Privacy Policy .

Reviewer #1: **Yes: ** Dr. Darcelle Schouw

**********

[NOTE: If reviewer comments were submitted as an attachment file, they will be attached to this email and accessible via the submission site. Please log into your account, locate the manuscript record, and check for the action link "View Attachments". If this link does not appear, there are no attachment files.]

While revising your submission, please upload your figure files to the Preflight Analysis and Conversion Engine (PACE) digital diagnostic tool, https://pacev2.apexcovantage.com/ . PACE helps ensure that figures meet PLOS requirements. To use PACE, you must first register as a user. Registration is free. Then, login and navigate to the UPLOAD tab, where you will find detailed instructions on how to use the tool. If you encounter any issues or have any questions when using PACE, please email PLOS at figures@plos.org . Please note that Supporting Information files do not need this step.

Attachment Submitted filename: Manuscript dds.docx

# Author comment: Author response to Decision Letter 1

**DOI:** 10.1371/journal.pone.0317460.r003

15 Sep 2024

Dear Dr. Darcelle Schouw and editorial office,

We are delighted to submit the revised version of our manuscript titled

"Intervention effectiveness in reducing the clustering of non-communicable disease risk factors in the workplace: a quasi-experimental study". Following the valuable feedback from both editors and the reviewer, we have carefully revised the manuscript. In this revised version, we have considered the suggestions provided by the editor and

reviewer. We have adjusted the text and added new paragraphs as necessary. Additionally, we have refined the program intervention frameworks based on the insightful remarks provided by the reviewer. Throughout the rest of this letter, we address and respond to each point raised by the reviewer. The comments from the reviewer are indicated in

red text, while our responses are provided in normal text underneath each

comment.

Authors

The funders had no role in study design, data collection and analysis, decision to publish, or preparation of the manuscript.

Question 1 What frameworks were used to derive at the programs to be

implemented?4

We have carefully considered the reviewer's comment and made several

modifications to the methodology section to provide greater clarity on the

research framework of our study. Additionally, we have included more

details regarding our methodology. Furthermore, we have provided as

supporting information, the overall project report, which includes this

study, containing all the necessary details about the general framework of

our research. We are open to any further remarks or suggestions aimed at

improving the quality of our work.

2- How would another organization be able to replicate what was done in

this study?

We have carefully revised the methodology section of our manuscript to

include additional details and ensure the continuity of ideas. We have also

enhanced the clarity of the procedures followed during the interventions,

as well as provided a comprehensive overview of the overall intervention

program to ensure the reproducibility of our work.

Additionally, we have included all necessary documents as supporting

information to supplement our manuscript.

We believe that these revisions significantly improve the quality and

transparency of our work, and we appreciate your valuable input. Please

let us know if there are any further suggestions or comments you may

have.

3- You omit details in the methodology which you only mention in the

discussion for the first time. Please let your methodology speak to your

results and discussion.

We have carefully considered your suggestion and have made revisions

accordingly. We have removed the unnecessary details from the discussion

section as you suggested, and we have added more extensive details to

ensure clarity and completeness in Methods section. We believe that these

changes have strengthened our manuscript, and we appreciate your

guidance in improving the quality of our work.

4-There are examples of studies in the workplace that focus on prevention

of NCDs in LMIC - I have made a comment in the manuscript for you to

look at.

We have carefully reviewed the articles you suggested, and we found

them to be incredibly useful. As per your recommendation, we have

incorporated these articles into our manuscript as references.

Please make use of an editor as there are words that are ambiguous and

makes it difficult to read. There are however certain places in the

manuscript where the English is written better than at other places.

We have carefully reviewed the entire manuscript and made the necessary

revisions to standardize the language, correct errors, and ensure clarity

and consistency throughout. We enlisted the help of a language editor and

a university-level English professor to assist us in this process.

We have revised the entire manuscript, adding the necessary references

throughout, and addressing all the points mentioned by both the editor

and the reviewer. We appreciate your efforts and remain open to any

further comments or suggestions you may have.

Authors

# Peer review: Decision Letter 1

**DOI:** 10.1371/journal.pone.0317460.r004

**Authors:** Barry Kweh

29 Dec 2024

Intervention effectiveness in reducing the clustering of non-communicable disease risk factors in workplace: a quasi-experimental study

PONE-D-23-30907R1

Dear Dr. Guesmi

We’re pleased to inform you that your manuscript has been judged scientifically suitable for publication and will be formally accepted for publication once it meets all outstanding technical requirements.

Within one week, you’ll receive an e-mail detailing the required amendments. When these have been addressed, you’ll receive a formal acceptance letter and your manuscript will be scheduled for publication.

An invoice will be generated when your article is formally accepted. Please note, if your institution has a publishing partnership with PLOS and your article meets the relevant criteria, all or part of your publication costs will be covered. Please make sure your user information is up-to-date by logging into Editorial Manager at Editorial Manager®  and clicking the ‘Update My Information' link at the top of the page. If you have any questions relating to publication charges, please contact our Author Billing department directly at authorbilling@plos.org.

If your institution or institutions have a press office, please notify them about your upcoming paper to help maximize its impact. If they’ll be preparing press materials, please inform our press team as soon as possible -- no later than 48 hours after receiving the formal acceptance. Your manuscript will remain under strict press embargo until 2 pm Eastern Time on the date of publication. For more information, please contact onepress@plos.org.

Kind regards,

Barry Kweh

Academic Editor

PLOS ONE

Additional Editor Comments (optional):

The authors have improved their manuscript by documenting the methodology more clearly as well as broadening the scope and nuances of their discussion as the reviewers' have noted.

Reviewers' comments:

Reviewer's Responses to Questions

**Comments to the Author**

1. If the authors have adequately addressed your comments raised in a previous round of review and you feel that this manuscript is now acceptable for publication, you may indicate that here to bypass the “Comments to the Author” section, enter your conflict of interest statement in the “Confidential to Editor” section, and submit your "Accept" recommendation.

Reviewer #2: All comments have been addressed

Reviewer #3: All comments have been addressed

**********

2. Is the manuscript technically sound, and do the data support the conclusions?

The manuscript must describe a technically sound piece of scientific research with data that supports the conclusions. Experiments must have been conducted rigorously, with appropriate controls, replication, and sample sizes. The conclusions must be drawn appropriately based on the data presented. 

Reviewer #2: Yes

Reviewer #3: Yes

**********

3. Has the statistical analysis been performed appropriately and rigorously? 

Reviewer #2: Yes

Reviewer #3: I Don't Know

**********

4. Have the authors made all data underlying the findings in their manuscript fully available?

The PLOS Data policy requires authors to make all data underlying the findings described in their manuscript fully available without restriction, with rare exception (please refer to the Data Availability Statement in the manuscript PDF file). The data should be provided as part of the manuscript or its supporting information, or deposited to a public repository. For example, in addition to summary statistics, the data points behind means, medians and variance measures should be available. If there are restrictions on publicly sharing data—e.g. participant privacy or use of data from a third party—those must be specified.

Reviewer #2: Yes

Reviewer #3: Yes

**********

5. Is the manuscript presented in an intelligible fashion and written in standard English?

PLOS ONE does not copyedit accepted manuscripts, so the language in submitted articles must be clear, correct, and unambiguous. Any typographical or grammatical errors should be corrected at revision, so please note any specific errors here.

Reviewer #2: Yes

Reviewer #3: Yes

**********

6. Review Comments to the Author

Please use the space provided to explain your answers to the questions above. You may also include additional comments for the author, including concerns about dual publication, research ethics, or publication ethics. (Please upload your review as an attachment if it exceeds 20,000 characters)

Reviewer #2: (No Response)

Reviewer #3: - The authors have adequately addressed the comments raised.

- The manuscript described a technically sound piece of scientific research with data that supports the conclusions

- The authors made all data underlying the findings in their manuscript fully available

- The manuscript presented in an intelligible fashion and written in standard English

**********

7. PLOS authors have the option to publish the peer review history of their article (what does this mean? ). If published, this will include your full peer review and any attached files.

If you choose “no”, your identity will remain anonymous but your review may still be made public.

**Do you want your identity to be public for this peer review?** For information about this choice, including consent withdrawal, please see our Privacy Policy .

Reviewer #2: No

Reviewer #3: **Yes: ** Prof. Mohamed Abd Ellatif

**********

# Editor report: Acceptance letter

**DOI:** 10.1371/journal.pone.0317460.r005

**Authors:** Barry Kweh

PONE-D-23-30907R1

PLOS ONE

Dear Dr. Guesmi,

I'm pleased to inform you that your manuscript has been deemed suitable for publication in PLOS ONE. Congratulations! Your manuscript is now being handed over to our production team.

At this stage, our production department will prepare your paper for publication. This includes ensuring the following:

* All references, tables, and figures are properly cited

* All relevant supporting information is included in the manuscript submission,

* There are no issues that prevent the paper from being properly typeset

If revisions are needed, the production department will contact you directly to resolve them. If no revisions are needed, you will receive an email when the publication date has been set. At this time, we do not offer pre-publication proofs to authors during production of the accepted work. Please keep in mind that we are working through a large volume of accepted articles, so please give us a few weeks to review your paper and let you know the next and final steps.

Lastly, if your institution or institutions have a press office, please let them know about your upcoming paper now to help maximize its impact. If they'll be preparing press materials, please inform our press team within the next 48 hours. Your manuscript will remain under strict press embargo until 2 pm Eastern Time on the date of publication. For more information, please contact onepress@plos.org.

If we can help with anything else, please email us at customercare@plos.org.

Thank you for submitting your work to PLOS ONE and supporting open access.

Kind regards,

PLOS ONE Editorial Office Staff

on behalf of

Dr. Barry Kweh

Academic Editor

PLOS ONE
